# Supplementary material for: A Reanalysis of the FDA’s Benefit–Risk Assessment of Moderna’s mRNA-1273 COVID Vaccine Based on a Model Incorporating Benefits Derived from Prior COVID Infection
Source: Vaccines (Basel). 2026 Feb 10;14(2):165. doi: 10.3390/vaccines14020165 (PMC12945011; doi:10.3390/vaccines14020165)
Supplement: Supplementary file 1 [file vaccines-14-00165-s001.zip › Supplement_S1_4071938.pdf]

Supplement S1: Using Data Available Before 22 January 2022 to Estimate Inputs for Hospitalizations Modeling[1]

.

Recall the input variables for our hospitalizations modeling:

Variables for U and V	Range of Values	Scenario A Values
I_r: Baseline-infection rate—fraction of an unvaccinated, infection-naïve test population of 18–25-year-old males expected to contract an infection over the 5-month evaluation period. Infections need not accumulate at a constant rate	$0 \le I_r \le 1$	1
H_r: Baseline infection-hospitalization rate for infection-naïve unvaccinated males 18–25	$0 \le H_r \le 0.01$	0.0014
F_{pi}: Fraction of a test population of 1 million unvaccinated males 18–25 having had a COVID-19 infection before the start of the 5-month evaluation period	$0 \le F_{pi} \le 1$	0.62
E_{pi}: Effectiveness of prior infection protection against COVID-19 infection	$0 \le E_{pi} \le 1$	0.45
E_v: Effectiveness of vaccination against COVID-19 infection	$0 \le E_v \le 1$	0.30
E_h: Effectiveness of hybrid protection against COVID-19 infection	$0 \le E_h \le 1$	0.57
HRR_{pi}: Hospitalization risk reduction for those reinfected and unvaccinated	$0 \le HRR_{pi} \le 1$	0.79
HRR_v: Hospitalization risk reduction for those fully vaccinated and experiencing their first COVID-19 infection	$0 \le HRR_v \le 1$	0.67
HRR_h: Hospitalization risk reduction for those reinfected and fully vaccinated	$0 \le HRR_h \le 1$	0.88
$H_{\mathrm{VAM/P}}$: Projected number of VAM/P hospitalizations occurring in the course of attaining a test population of 1 million fully mRNA-1273 vaccinated 18–25-year-old males	$H_{\mathrm{VAM/P}} \approx 268$	268

Also recall that the FDA used end-of-December 2021 case and hospitalization data from the CDC to project cases and hospitalizations prevented over the hypothetical 5-month evaluation period by mRNA-1273 vaccinating 1 million representative males 18–25, where we have defined the evaluation period to be 1/1/22 – 5/31/22. Our modeling of COVID hospitalizations makes projections that account for age-adjusted infection-hospitalization risk, the level of infection anticipated over the evaluation period, and the effectiveness of protection against infection and hospitalization provided by prior infection alone, vaccination alone, and hybrid protection provided by both vaccination and prior infection. Here, we use data available before 1/22/2022 to derive estimates for all the variables needed in our infections-based modeling, such as those for Scenario A appearing the table above.

S1. Estimating I_r

Overview. In our Scenarios A–C, which correspond, respectively, to the FDA's Scenarios 1–3, the values of I_r are computed, respectively, from the FDA's estimates of cases prevented in its Scenarios 1–3 [1, Table 2]. In our Scenarios D and E, I_r is derived from the assumption that over the evaluation period the increase in COVID-infection level among 18–25-year-old males is twice that of the general population during the second COVID wave in the U.S. (10/1/20--2/28/21).

Detailed Discussion. As we discussed in Section 1.3 of the main exposition, in its Scenario 1, the FDA assumed that via vaccination 82,484 cases would be prevented over 5 months with a vaccine effectiveness of 30%; thus, the FDA assumed 82,484/0.3 ≈ 274,947 cases would be reported over 5 months among 1 million representative unvaccinated males 18–25. Assuming the CDC's case-to-infections multiplier of 4 from [2] continues to be valid over the evaluation period, the FDA's case projection would correspond to over 1 million infections, so that the FDA essentially assumed that all unprotected (i.e., unvaccinated) 18–25-year-old males would become infected over the 5-month evaluation period. Thus, in Scenario A, our reanalysis of Scenario 1, we too assume all unprotected persons in the test population become infected. However, in our modeling, the unprotected are the infection-naïve unvaccinated. Assuming all such persons become infected over 5 months is equivalent to setting $I_r = 1$, our Scenario-A value for I_r.

In its Scenario 2, the FDA assumed a more modest number of cases would accumulate over 5 months (corresponding to the average weekly COVID-19 case-incidence rate in 2021); specifically, the agency assumed 26,705 cases would be prevented, corresponding to roughly $4 \cdot 26{,}705/.3 \approx 356{,}067$ infections, yielding I_r= 0.356 in our Scenario B. Finally, for its Scenario 3, the FDA assumed the lowest COVID-19

[1] Supplement to "A reanalysis of the FDA's benefit-risk assessment of Moderna's mRNA-1273 COVID vaccine based on a model incorporating benefits derived from prior COVID infection" by P.S. Bourdon, R. Duriseti, H.C. Gromoll, D.K. Dalton, K. Bardosh, and A.E. Krug.

weekly case-incidence rate over 2021 would apply over its 5-month evaluation period yielding 3,903 cases prevented, corresponding to roughly 4 · 3903/.3 ≈ 52,040 infections. Thus, I_r= 0.052 in our Scenario C.

We derived our Scenario D estimate $I_r \approx 0.456$ as follows. Based on the figure below, produced using CDC case [3] and seroprevalence [4] data, we estimate that during the second COVID wave in the U.S., the COVID-19 infection level in the U.S. increased by 14%, where we have assumed the wave took place over the 5-month period, 1 October 2020 through 28 February 2021.

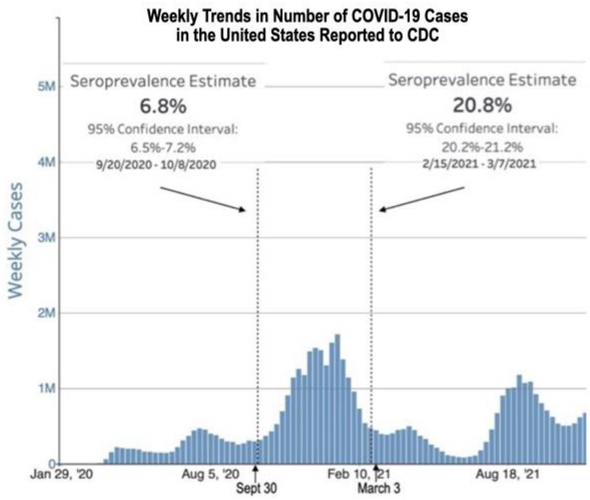

Suppose that on 1 January 2022 we project an infection-level jump of twice that of the second wave: 28% over 5 months among males 18–25. We estimate the corresponding value of I_r below.

According to the CDC [5], as of 12/31/21, 60.6% of those 18–24 had been fully vaccinated. Assume the following effectiveness-of-protection levels: 30% for infection-naive fully vaccinated individuals, 47% for those having prior infection, and 57% for those that are vaccinated and have had a prior infection. (The 47% and 57% effectiveness estimates are derived in Section S4 below.) Consider a large population having N members with the following mix of protection: 60.6% have been vaccinated (primary series only) and among the unvaccinated 25% are infection-naive while among the vaccinated 60% are infection naive.[2] If the level of infection of this population increases 28% over 5 months and I_r represents the fraction of the infection-naive unvaccinated who become infected, then we would expect

$$(0.394 \cdot N \cdot 0.25)I_r + (0.394 \cdot N \cdot 0.75)I_r(1-0.47) + (0.606 \cdot N \cdot 0.6)I_r(1-0.3) + (0.606 \cdot N \cdot 0.4)I_r(1-0.57) = 0.28 \cdot N,$$

where the first summand on the left represents the number of infections among the infection-naïve unvaccinated, the second, the number of infections among the unvaccinated having prior infection, the third, the number of infections among the infection-naïve vaccinated, and the fourth, the number of infections among the vaccinated having prior infection. The displayed equation of the preceding sentence yields $I_r \approx 0.456$, which is our value for Scenarios D and E. In early January of 2021 it would have been difficult to characterize as a likely underestimate our assumption of Scenarios D and E that the rise in infection level in the general-population over 1/1/22 – 5/31/22 would be twice that of the second wave because there was no vaccine protection of the U.S. population during the first half of the second wave, very little vaccine protection of young persons during the whole wave, and there were considerably more COVID-naïve persons during the second wave than at the end of 2021.

S2. Estimating H_r (Baseline Infection-Hospitalization Rate)

Overview. We estimate H_r using an IHR model [6] developed via a seroprevalence-based meta-analysis anchored to the ancestral strain of SARS-CoV-2.[3] Applying the model, we obtain a "with COVID" IHR

---

[2] The assumption that 40% of the vaccinated have experienced a COVID-19 infection is a crude estimate intended to account for waning of vaccine effectiveness against the Delta and Omicron variants as well as for the months the vaccinated were unvaccinated (e.g., [5] indicates that through 2 April 2021, well over a year after the first COVID-19 infections were reported in the U.S., only 18.2% of those 18–24 had been vaccinated).

[3] The meta-analysis preprint [6] appeared 10/29/21 (an earlier version appeared 7/31/21); [7] is the published version of [6].

of 0.37% for the infection-naïve unvaccinated of ages 18–25 and reduce this rate assuming 40% of hospitalizations attributed to COVID are incidental, arriving at an IHR of 0.22% ($\approx 0.6 \cdot 0.37\%$) for infection-naïve unvaccinated 18–25 year-olds). The 0.22% rate for the general population of 18–25 year-olds in the U.S. (males and females) is consistent with U.S. military COVID-19 hospitalization data from the period 1 February 2020 through 4 January 2021 [8].

In its benefit-risk assessments [9] and [1] for, respectively, Pfizer's and Moderna's mRNA vaccines, the FDA relied on sex-specific CDC hospitalizations data. The case and hospitalization rates per 100,000 persons in Table 2 of [9] suggests that for the 18–24 age range, females are 2.53 times as likely as males to be hospitalized with COVID-19 and 1.21 times as likely to have a case of COVID-19.[4] We use hospitalization and case data from Table 2 of [9] to estimate the IHR for unvaccinated, infection-naïve males 18–25 to be 0.14%.

Detailed Discussion. D. Herrera-Esposito and G. de los Campos provide a meta-analysis [6] based on data from thirteen different countries (including four different locations in the U.S.) that yields age-stratified

- infection-hospitalization rates for COVID-19 (a measure of disease severity and denoted "ISR"),
- infection-ICU-admissions rates (indicating critical disease and denoted "ICR"), and
- infection-fatality rates (denoted "IFR").

As we discuss below, the application of Herrera-Esposito and de los Campos's model to the age range of greatest interest 18–25 yields an infection-hospitalization rate consistent with that suggested by U.S. Military COVID-hospitalization data for the period ending 1/4/21 [8]. In [6], ISR, "infection-severe rate," is defined as the rate of "infections resulting in hospitalization or out-of-hospital death." Because the number of out-of-hospital COVID-19 deaths is quite small relative to the number of COVID-associated hospitalizations, we assume ISR well approximates IHR, infection-hospitalization rate.

Infections contributing to the IHR modeling of [6] were principally ancestral strain. By early January 2022, several studies (described at the end of this section) comparing the severity of Omicron to Delta as well as to the ancestral strain were available, with all the studies suggesting that the IHR for Omicron would likely be less than or equal to that of the ancestral strain. In our modeling of COVID-hospitalizations of 18–25 year-olds, we approximate the IHR for Omicron using the IHR suggested by [6], with an incidental-hospitalizations adjustment.

Our goal is to use Herrera-Esposito and de los Campos's ISR-IHR model to derive an infection-hospitalization rate IHR for unvaccinated 18–25 year-olds that the FDA might have used in its benefit-risk modeling of mRNA-1273. The estimated age-stratified IHRs of [6] appear in its Table 1,[5] which we have copied below:

Age	ISR % (CrI)	ICR % (CrI)	IFR % (CrI)
0-9	0.103 (0.063-0.162) (*)	0.0088 (0.0053-0.0139) (*)	0.00050 (0.00025-0.00087) (*)
10-19	0.22 (0.13-0.35)	0.024 (0.014-0.037)	0.0019 (0.0010-0.0033)
20-29	0.47 (0.28-0.74)	0.063 (0.038-0.10)	0.0072 (0.0037-0.0126)
30-39	0.99 (0.57-1.61)	0.17 (0.10-0.28)	0.027 (0.014-0.049)
40-49	2.1 (1.2-3.5)	0.46 (0.26-0.77)	0.10 (0.05-0.19)
50-59	4.4 (2.4-7.4)	1.2 (0.6-2.1)	0.40 (0.18-0.77)
60-69	8.9 (4.6-15.2)	3.3 (1.6-5.9)	1.5 (0.6-3.0)
70-79	17.1 (8.9-28.8)	8.3 (3.9-15.5)	5.5 (2.3-11.3)
80+	30.3 (16.4-47.7)	19.4 (9.2-34.7)	18 (7.5-34.3)

Consistent with earlier findings that the COVID-19 IFR increases exponentially with age, Herrera-Esposito and de los Campos show that the risk of severe COVID-19 also increases exponentially with age (but at a lower rate than that of fatal illness). We fit an exponential modeling function $M(t) = ae^{bt}$ to

[4] Moreover, these relationships are consistent with those of [10, Table 1] based on data from 3/1/20 to 3/1/22.

[5] Values in Table 1 of [6] are identical to those of Table 1 of [7], the published version of [6].

the first four data points in the second column of the table above via least-squares minimization, over the variables a and b, of the sum

$$S = (A_M[0,10] - 0.103)^2 + (A_M[10,20] - 0.22)^2 + (A_M[20,30] - 0.47)^2 + (A_M[30,40] - 0.99)^2,$$

where

$$A_M[\alpha, \beta] = \frac{1}{\beta - \alpha}\int_{\alpha}^{\beta} M(t)dt = \frac{1}{\beta - \alpha}\int_{\alpha}^{\beta} a\, e^{bt} dt$$

is the average value of M over $[\alpha, \beta]$. Values of a and b that minimize S are $a \approx 0.07014$ and $b \approx 0.0750,$ and when $a = 0.07014$ and $b = 0.0750$, the square root of the sum S above is less than 0.003. Thus, our modeling function is $M(t) = 0.07014\, e^{0.0750t}$.

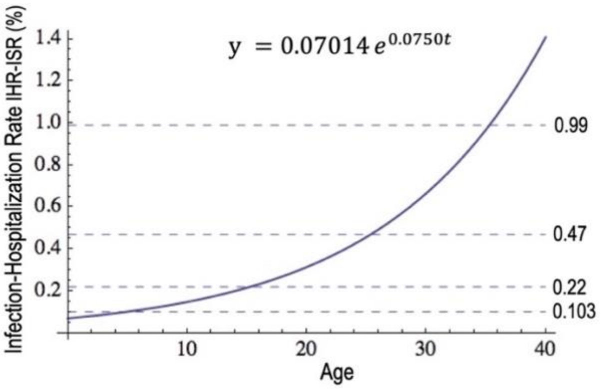

The reader may check that the average value of the function $M(t) = 0.07014\, e^{0.0750t}$ over the intervals [0, 10], [10, 20], [20, 30], and [30, 40], rounded to two decimal places, are, respectively, 0.10, 0.22, 0.47, and 0.99, in agreement with values in the table above for these age intervals. The average value of M over [18, 26],

$$\frac{1}{8}\int_{18}^{26} M(t)\ dt \approx 0.37\%,$$

is thus the infection-hospitalization-rate estimate for the age range 18–25 suggested by Herrera-Esposito's model, a value in good agreement with the finer age-range IHR (ISR) estimates provided in supplementary Table S1 of [6], reproduced below.[6]

Age	ISR % (CrI)	ICR % (CrI)	IFR % (CrI)
0-4	0.086 (0.052-0.13) (*)	0.0069 (0.0042-0.0109) (*)	0.00036 (0.00018-0.00062) (*)
5-9	0.13 (0.08-0.20) (*)	0.011 (0.007-0.018) (*)	0.00070 (0.00035-0.00121) (*)
10-14	0.18 (0.11-0.29)	0.019 (0.011-0.029)	0.0014 (0.0007-0.0024)
15-19	0.27 (0.16-0.42)	0.030 (0.018-0.047)	0.0026 (0.0014-0.0046)
20-24	0.39 (0.23-0.61)	0.050 (0.030-0.078)	0.0051 (0.0027-0.0090)
25-29	0.56 (0.33-0.89)	0.081 (0.049-0.129)	0.010 (0.005-0.018)
30-34	0.82 (0.48-1.33)	0.13 (0.08-0.22)	0.020 (0.010-0.035)
35-39	1.2 (0.7-2.0)	0.22 (0.13-0.36)	0.038 (0.019-0.069)
40-44	1.7 (1.0-2.9)	0.36 (0.20-0.60)	0.075 (0.036-0.138)
45-49	2.5 (1.4-4.2)	0.59 (0.32-0.99)	0.15 (0.07-0.27)
50-54	3.7 (2.0-6.2)	0.96 (0.51-1.66)	0.29 (0.13-0.55)
55-59	5.2 (2.8-8.9)	1.6 (0.8-2.8)	0.56 (0.25-1.09)
60-64	7.5 (3.9-12.8)	2.6 (1.3-4.6)	1.1 (0.5-2.2)

All the preceding infection-hospitalization rates are computed based on the assumption that a "COVID hospitalization" is any hospitalization of a person who tests positive for COVID, even if the person is hospitalized for treatment of a disorder or injury totally unrelated to COVID; i.e., the preceding rates include incidental COVID hospitalizations.

A person contracting a COVID infection may test positive for an extended period of time. According to one study [11], "[T]he average time to transition from RT-PCR positive to negative was 24 days after symptom onset and 10% remained positive even 33 days after symptom onset." Thus, it is not surprising

[6] Values in Table S1 of [6] are identical to those of Table S1 of [7].

that a person's positive PCR test upon hospital admission is often incidental to the true reason for admission.

A natural choice for the Omicron incidental-hospitalization rate that the FDA might have made for its mRNA-1273 assessment [1] is 40%, given CDC director Walensky's interview comment 1/9/22 "[U]p to 40 percent of the patients who are coming in with COVID-19 are coming in … with something else" [12], and Canada's province of Ontario published incidental rates for January 8–15 exceeding 45% [13].[7,8] In addition, there is evidence COVID incidental-hospitalization rates are higher for younger age ranges.[9] For example, a major study [16] based on data from Germany from the pre-vaccination period (for children) March 2020 to May 2021 found, "As of May 2021, the cumulative rate for hospitalization associated with SARS-CoV-2 infection was 35.9 per 10,000 children" and "When limiting the analysis to include only patients with COVID-19 who required therapeutic interventions, the hospitalization rate decreased 5.5-fold to 6.5 per 10,000 children," which suggests an incidental rate of over 80% "for children" (defined to be persons 0-17-years-old).[10]

Further evidence that a significant percentage of hospitalizations attributed to COVID are not severe or perhaps are incidental derives from the following data provided by a report [18] of the Imperial College London COVID-19 Response Team:

Table 1. Delta and Omicron cases and hospitalisations for cases with specimen dates in the period 1-14 December 2021. Results are shown for all cases, Pillar 2 cases only and Pillar 2 symptomatic cases only. For all Pillar 2 cases, we also show hospitalisations involving at least 1 night's stay. Numbers between 1 and 5 shown as ≤5.

	All cases (pillars 1 and 2)				Pillar 2 cases						Pillar 2 symptomatic cases			
Last PCR test specimen date (December 2021)	Delta cases	Omicron Cases	Delta hospital-isations	Omicron hospital-isations	Delta cases	Omicron Cases	Delta hospital-isations	Omicron hospital-isations	Delta hospital-isations (>0 days)	Omicron hospital-isations (>0 days)	Delta cases	Omicron Cases	Delta hospital-isations	Omicron hospital-isations
1	25098	164	586	≤5	24615	156	465	≤5	122	0	10548	76	174	≤5
2	23212	226	493	≤5	22783	208	368	≤5	109	0	9831	110	125	≤5
3	21111	252	449	7	20704	241	313	≤5	78	0	8635	122	99	≤5
4	17235	303	372	≤5	16924	291	256	0	73	0	7258	144	86	0
5	16164	434	337	20	15868	418	214	12	52	≤5	6650	205	65	≤5
6	20745	977	397	14	20288	927	291	≤5	64	≤5	8769	453	89	≤5
7	21058	1916	395	24	20609	1869	265	16	66	≤5	8588	848	79	8
8	20551	2956	353	25	20133	2886	223	18	65	≤5	8398	1243	66	8
9	21639	4047	316	29	21302	3991	205	15	43	≤5	9075	1725	64	6
10	21869	5214	255	33	21567	5153	156	19	43	≤5	8921	2294	51	9
11	15581	4807	177	30	15392	4767	114	21	28	≤5	6463	2116	32	7
12	13746	6550	123	40	13639	6519	81	26	20	≤5	5667	3003	23	13
13	17489	11933	109	26	17391	11908	88	25	22	7	7573	5801	28	14
14	13819	15804	52	36	13805	15802	52	36	12	6	6240	7579	22	7
						Column totals:	3091	200*	797	37*				

* assuming ≤ 5 = 3

Note that only about 25.8% (≈ 797/3091×100%) of the Pillar-2 hospitalizations with Delta lasted one day or more, and, assuming "≤5" is 3, only about 18.5% (≈ 37/200×100%) of the hospitalizations with Omicron lasted one day or more. Remarks: (i) "The mean ages of Omicron and Delta cases attending hospital were 30.8 (95%CI:28.6-33.0) years and 38.3 (95%CI:37.5-39.1) years, respectively" [18, p. 6]. (ii) Pillar 2 hospitalizations are of those testing positive through "routine community testing" while Pillar 1 test positive through hospital-based testing done for all admitted to hospital. The Imperial College COVID-Response Team deemed Pillar 1 hospitalizations more likely to be incidental [18, p. 10].

We believe the discussion above justifies an assumption that incidental hospitalizations account for at least 40% to 50% of the hospitalizations contributing to our IHR estimate of 0.37% derived from the

---

[7] The average of daily rates during 1/8/22 – 5/31/22 is 52.8% [13].

[8] Consistent with the 40% and 45% incidental-rate estimates, McAlister et al find, "The proportion of incidental SARS-CoV-2 infections rose from 10% in Wave *1 to 41% during the Omicron wave* [14]." Moreover, McAlister et al. underestimate the incidental rate because they consider only hospital admissions through emergency departments. Thus, they ignore obvious sources of incidental COVID hospitalizations: scheduled surgeries, labor and delivery, and doctor referrals.

[9] U.S. data contributing to the findings of [6] comes from a period during which the incidental-hospitalization rate is estimated to be between 70% and 75% for those in the age range 0 to 18 and between 30% and 40% for those 19 to 65 (according to a large study [15]). See the conclusion of Appendix S1 to this supplement for a discussion, based on the model of [6] and data from Connecticut and the CDC suggesting an incidental rate 70% or higher for 15–24 year-olds in the U.S. for the year 2020. A direct comparison of Connecticut and CDC data in Table S1.5 of Appendix S1 suggests an incidental rate 80% or higher for 15–24 year-olds in the U.S. for the year 2020. We note that Figure 2 of [15] indicates the incidental rate for 0–18 year-olds was typically higher before the COVID vaccination campaign began.

[10] [16] was later published [17] (but without the comparison of reported "associated rates" to rates for treatment).

model of [6] for the age range 18–25. In other words, the preceding suggests the following range for a valid IHR, which we denote by H_r, for unvaccinated 18–25 year-olds (males and females combined):

$$H_r \text{ lies in the interval between } 0.5 \cdot 0.37\% \approx 0.185\% \text{ and } 0.6 \cdot 0.37\% \approx 0.22\%.$$

As a plausibility check on the IHR range $0.00185 \leq H_r \leq 0.0022$, consider the following data from [9] on COVID-19 hospitalizations, from 1 February 2020 through 4 January 2021, among U.S. Military service members:

Number of COVID-19 cases in DOD, as of 6 a.m. EST on Jan. 4:

	Cumulative Cases	Cumulative Hospitalized	Cumulative Recovered	Cumulative Deaths
**Military**	110,018 (+12,973)	945 (+64)	72,647 (+13,179)	14
**Civilian**	31,301 (+5,063)	895 (+96)	17,053 (+3,327)	118 (+19)
**Dependent**	17,862 (+2,500)	215 (+21)	11,188 (+1,961)	9
**Contractor**	10,733 (+1,618)	326 (+39)	6,397 (+1,144)	42 (+4)
**Total**	169,914 (+22,154)	2,381 (+220)	107,285 (+19,611)	183 (+23)

The first row of tabulated data above provides a case hospitalization rate of

$$\frac{945}{110108} = 0.00859, 0.859\%.$$

Note that the preceding case-hospitalization rate is not influenced by vaccination, given the data cut-off of 4 January 2021. Also, it is not influenced by reinfections: according to the UK Coronavirus Dashboard [19] as of 4 January 2021, England reported 2,533,577 COVID cases, but only 4359 COVID reinfections, a case reinfection rate of approximately 0.172%. Thus, it's safe to assume that the U.S. Military 2020 case hospitalization rate we computed above is not meaningfully affected by cases resulting from reinfections.

If we use the CDC case to infection multiplier of 4 (computed for February 2020–September 2021) [2], we get an ancestral-COVID-variant IHR for the U.S. military of approximately

$$\frac{0.00859}{4} \approx 0.002148,$$

which lies in our IHR interval $0.00185 \leq H_r \leq 0.0022$. This completes our plausibility check.
Remarks: Note that we have not adjusted the preceding U.S. Military IHR for incidental hospitalizations and that the median age of active duty service members is over 25 according to a 2020 DoD report [20, Section 2.41]; thus, there is reason to believe the actual IHR is lower.[11] However, the percentage of service members having comorbidities is likely lower than that of the general population. Also, in 2020 the DoD reported the percentage of male active-duty service members to be approximately 82.8% [20, Section 2.14].

We have provided evidence that the IHR for the ancestral strain of COVID-19 lies between 0.00185 and 0.0022 for U.S. residents (males and females) 18–25 years old. By the end of December of 2021, there were already reports and data indicating that Omicron is milder than Delta as well as the ancestral strain.

In terms of early comparisons of the risk of hospitalization for Omicron versus for Delta, a UK Health Security Agency technical briefing [21] states, "The risk of hospital admission alone with Omicron was approximately one-third of that for Delta (Hazard Ratio 0.33, 95% CI: 0.30 to 0.37)." A preprint [22] appearing 21 December 2021[12] found "Compared to earlier Delta infections, after controlling for factors

[11] Evidence the Omicron IHR for 18–25 year-olds is perhaps significantly lower than even 0.185% is provided by 2020 hospitalization data for the State of Connecticut and the CDC. See Appendix S1 to this supplement.

[12] and later published in the Lancet [23]

associated with severe disease, SGTF-infected [Omicron] individuals had a lower odds of severe disease (aOR 0.3, 95% CI 0.2-0.5)." A study [24] e-published 28 December 2021 found, "Fewer than half (45%) of patients in COVID-19 wards [with Omicron infections] required oxygen supplementation compared to 99.5% in the first [ancestral-strain] wave."

The preceding (early) comparisons of the severity of Omicron variant relative to the Delta variant as well as the ancestral strain suggests that it would have been reasonable to assume, 1 January 2022, that Omicron's IHR should be no higher than that of the ancestral strain.[13] Thus, we will take H_r = 0.0022 as an estimate of IHR for U.S. residents 18–25 years old (general-population mix of males and females).

IHR for Males Lower than that for Females. In its benefit-risk assessments [9] and [1] for, respectively, Pfizer's and Moderna's mRNA COVID vaccines, the FDA relied on sex-specific CDC hospitalizations data. For the FDA's Moderna assessment, which did not include an analysis of vaccine benefits and risks for females, hospitalization rates are provided only for males. However, because the FDA's Pfizer study [9] considered benefits and risks for female-only as well as male-only age-groups, COVID hospitalization rates are provided based on age and sex. These rates appear in Table 2 of [9], reproduced below.

Table 2 of [20]
Vaccine coverage and COVID incidences by sex and age subgroups.

Sex	Age subgroup	Population[1]	Vaccinated population[2]	COVID-19 cases/100 k persons[2]	Hospitalizati ons/100 k persons[3]	Percent of hospitalized going to ICU[3]	Percent of hospitalized who die[3]
Female							
	16–17	4,119,686	1,985,672	47.9	1.593	19.5	0.7
	18–24	14,923,948	8,033,040	64.6	2.025	8.1	1
	25–29	11,428,122	5,918,524	68.6	2.45	5.9	0.3
Male							
	16–17	4,300,731	1,826,299	42.9	0.35	32.7	0.7
	18–24	15,633,953	7,217,945	53.3	0.8	22.2	0.6
	25–29	12,036,982	5,592,473	57.8	0.875	22.7	1.5

Source: 1-CDC Wonder, 2-COVID Data Tracker, 3-COVID NET.

The incidence data in the preceding table is for the week of 10 July 2021, while the hospitalization data is an average for the four-week period concluding 10 July 2021. Observe Table 2 of [9] indicates that for the 18–24 age range, the with-COVID-19 hospitalization rate for females is 2.53 times that for males; also observe that Table 2 of [9] suggests that females are more likely to have a COVID case, with female to male case-rate ratio of 1.21.[14] We assume that the with-COVID hospitalization-rate ratio 2.53 also reflects the for-COVID hospitalization-rate ratio; equivalently, incidental-hospitalization rates are similar for males and females in the 18–24 age bracket.

Note that the lower case and hospitalization rates among males provided in the table above cannot be attributed to greater COVID-vaccine uptake among males vs. females. In fact, the opposite is likely true; e.g., the spreadsheet of data for Figure 3 at the Government of Canada website [27] indicates that in Canada 35.5% of females and 25.9% of males in the age range 18–29 had been fully vaccinated as of 10 July 2021 (with corresponding percentages as of 8/7/21 being 60.2% and 51.1%). The CDC spreadsheet [28], which unfortunately does not give male/female vaccine-uptake by age, indicates that in the United States 51.9% of females and 47.5% of males had been fully vaccinated as of 10 July 2021.

We assume that the case rates of Table 2 of [9] mirror infection rates. Focus on the data for 18–24 year-olds in Table 2 of [9]: If N infections among a population of 100,000 males yield 0.8 hospitalizations we expect 1.21 N infections among a population of 100,000 females to yield 2.025 hospitalizations. We therefore estimate the ratio of female IHR to male IHR to be

$$\frac{2.025/(1.21N)}{0.8/N} \approx 2.09.$$

---

[13] A Johns Hopkins study [25] found a hospitalization hazard ratio for Omicron versus the ancestral strain of 0.94 while another study [26] (based on data from the state of Washington) found the hazard ratio to be 0.92.

[14] Similar differences in hospitalization and case rates are reported in Table 1 of [10], which provides "Descriptive statistics of COVID-19 cases [in Israel] from 1 March 2020 until 1 March 2022, (n = 3,605,400)" indicating that

- for the 10–19 age range, the percentage of confirmed COVID cases among males that result in hospitalization is 0.241% and among females is 0.297%, while
- for the 20-29 age range, the percentage of confirmed COVID cases among males that result in hospitalization is 0.681% and among females is 1.577%,

Note the preceding percentages are case hospitalization rates; we expect these rates to be somewhat lower if restricted to Omicron cases, and we expect corresponding infection-hospitalization rates to be much lower. Moreover, Table 1 of [10] reports positivity rates for COVID tests among young females exceed those among young males, e.g., 8.6% vs. 6.2% for 20-29 year-olds.

We assume this IHR ratio continues to be valid for 18–25 year-olds and that the ratio of female-to-male infections continues to be 1.21. Now consider a representative sample of 100,000 infected persons 18–25 years old consistent with the data in Table 2 of [9]. Assuming that the ratio of infected females to infected males in this sample is 1.21, we have 45,249 infected males in the sample and 54,751 infected females. Assuming an IHR of 0.22% applies to the sample as a whole and r is the IHR for males in the sample while $2.09 \cdot r$ is the IHR for females in the sample, we have

$$45{,}249 \cdot r \; + \; 54{,}751 \cdot 2.09 \cdot r \; = \; 0.0022 \cdot 100{,}000,$$

so that $r \; \approx \; 0.0014$. Thus, based on data from Table 2 of [9] we assume an IHR of 0.14% for males in the age range 18–25 (and the corresponding rate for females is about 0.29%).

In Section S2 of Supplement S2, we complete a sensitivity analysis, in which we examine how the hospitalizations-prevented benefit of mRNA-1273 vaccinating 1 million males 18–25 changes based on the assumption that there are no sex-based differences in IHR and infection rate (so that, in particular, the IHR for males 18–25 is assumed to be 0.22%).

S3. Estimating F_{pi}

Overview. For our Scenarios A--C we use the value $F_{pi} = 0.62$, which is the average of (a) the CDC's estimate that 54.9% of those 18–49 years old had been infected by COVID-19 before 10/1/21 [2] with (b) an estimate derived below that 69% of unvaccinated male 18–25 year-olds had been infected by 1/1/2022. For Scenarios D and E, we use $F_{pi} = 0.69$.

Detailed Discussion. Because test populations are "representative," F_{pi} should equal the fraction of the unvaccinated males 18–25 in the general U.S. population estimated to have had a COVID-19 infection by 1 January 2022. We will assume initially that infection rates in males and females are comparable and that the fraction of those 18–29 infected by 1/1/22 well approximates the fraction of those 18–25 infected by 1/1/22.

As a first step in estimating F_{pi}, we will use COVID-case data by age and CDC infection-incidence data from [2] to derive an estimate for the fraction of the population of ages 18–29 having been COVID-19 infected by 10/1/21. In Table 2 of [2], we find the CDC estimate that 54,860 is the "Infected rate per 100,000" for those 18–49 years old as of the end of September 2021; that is, COVID-infection incidence for the 18–49 age group as of the end of September 2021 stands at approximately 0.5486. We expect the infection incidence to be higher for 18–29 year-olds. Consider, e.g., the following cumulative case counts by age from [29] as of 9/29/21:

Total number of cases of COVID-19 in the United States as of September 29, 2021, by age group [29]

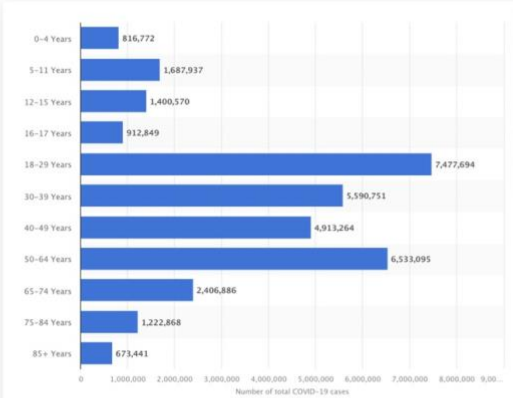

According to the U.S. Census Bureau estimates [30], population totals in the U.S. for the age ranges 18–29, 30–39, 40–49, as of 7/1/21, are, respectively, 53,571,758, 45,241,992, and 40,786,132. Thus, using the case totals from the preceding chart, we find the following COVID-case incidences as of 9/29/21:

18–29 year-olds: 0.1396, 30–39 year-olds: 0.1236, and 40–49 year-olds: 0.1205.

Thus, if r is case incidence for 18–29 year-olds, then $0.8854r$ is case incidence for 30–39 year-olds, and $0.8632r$ is case incidence for 40–49 year-olds. If we assume these age-group case-incidence relations well approximate infection-incidence relations, then we can arrive at an estimate for infection incidence

among 18–29 year-olds in the U.S. as of the end of September 2021 as follows. Let r now represent COVID-infection incidence for the age group 18–29 as of the end of September 2021 and recall that, according to the CDC [2], infection incidence for the 18–49 age group stands at 0.5486 as of the end of September 2021. We have

$$53{,}571{,}758 \cdot r + 45{,}241{,}992 \cdot 0.8854r + 40{,}786{,}132 \cdot 0.8632r = 0.5486 \cdot 139{,}599{,}882,$$

where 139,599,882 is the total (2021) population of 18–49 year-olds in the U.S. Solving the preceding equation for r, yields $r \approx 0.594$. Thus, we estimate that by 10/1/21, 59.4% of those 18-29 in the U.S. had been infected by COVID-19. We assert that 0.594 is a conservative estimate of the fraction of the *unvaccinated* 18–29 having experienced a prior infection as of the end of September 2021. There is certainly reason to expect a larger percentage of the unvaccinated to have been infected by the end of September of 2021 than of the vaccinated: vaccination does provide some protection from infection and those having had an infection might be more reluctant to vaccinate—they might assume they have natural immunity; also, they might be aware of studies (such as [31]) suggesting that those who are not infection naïve have a greater likelihood of experiencing a severe adverse event after vaccination.

Recall from the main exposition that our benefit-risk analysis is based on a hypothetical test population of 1 million unvaccinated males 18–25, a percentage of whom have had a COVID infection prior to 1/1/22. Our aim is to approximate that percentage in two steps with the first step being to approximate the percentage having prior infection on 1/1/22 in a test population consisting of 1 million unvaccinated 18–25 year-olds (*general-population mix of males and females*). Taking this test population back to 10/1/21 and assuming infected rates for those 18–25 well approximate those for 18–29, we assume 59.4% of this test population had a COVID infection prior to 10/1/21. We seek to determine how much the infected percentage increases over the period 10/1/21 through 12/31/21.

To do this, we rely on average weekly COVID-case incidence data for the period 10/3/21–12/25/21 provided in a CDC Morbidity and Mortality Weekly Report (MMWR) [32] that was published online 1/21/2022. We assume that if the FDA had wished to consider the protection that prior infection provides against reinfection and hospitalization, the agency could have obtained the incidence data in [32] directly from the CDC in early January of 2022.[15] The incidence data from [32] is for the age group 18–49 and is expressed in terms of average weekly cases per 100,000. We'll assume case incidence per 100,000 for the 18–29 range is well approximated by that for the 18–49 range. (Case incidence computations above suggest the incidence rate for the range 18–29 might be higher, but our computations above suggest a greater percentage of the 18–29 age range vs. the 18–49 range have prior-infection protection from reinfection.)

Table 2 of [32] indicates the average weekly incidence of COVID-19 among the unvaccinated to be 330.3 cases per 100,000 for 18–49 year-olds during the months of October and November of 2021, and 745.6 cases per 100,000 for 18–49 year-olds during the month of December of 2021. Thus, for the 8 + 5/7 weeks of October and November of 2021, total COVID incidence among the unvaccinated was approximately $8.71 \cdot 330.3 \approx 2876.9$ cases per 100,000 and approximately $4.43 \cdot 745.6 \approx 3303.0$ for the 4 + 3/7 weeks of December 2021.

Let's focus on estimating how many infection-naïve unvaccinated 18–29 year-olds in our test population of 1 million became infected during October and November of 2021. We are assuming that at the beginning of October, this group of 1 million unvaccinated 18–29-year-olds would comprise 406,000 that are infection-naïve and 594,000 that have had a previous infection. Based on the CDC data discussed in the preceding paragraph, we assume that by the end of November 28,770 COVID cases would be reported for this group. We seek to estimate how many of these cases occurred among the infection naïve. We rely on data from another CDC MMWR [33] published online 1/19/2022 and presenting data from the period May–November 2021. Again, we assume that had FDA wished to consider the protection that prior infection provides against reinfection (and hospitalization), the agency could have obtained the following data from [33] directly from the CDC by early January of 2021: "By the week beginning October 3, compared with COVID-19 cases rates among unvaccinated persons without a previous COVID-19 diagnosis, case rates among vaccinated persons without a previous COVID-19 diagnosis were

[15] We also note that in the third paragraph of Section 4 of [1], the FDA cites data ("23% VE") from a preprint by Tseng et al. posted at medRxiv 1/21/2022 (https://www.medrxiv.org/content/10.1101/2022.01.07.22268919v2). This 23% VE estimate did not appear in the 1st version of the preprint posted at medRxiv.

6.2-fold (California) and 4.5-fold (New York) lower; *rates were substantially lower among both groups with previous COVID-19 diagnoses, including 29.0-fold (California) and 14.7-fold lower (New York) among unvaccinated persons with a previous diagnosis*, and 32.5-fold (California) and 19.8-fold lower (New York) among vaccinated persons with a previous diagnosis of COVID-19."

Let r be the COVID-case rate among the infection-naïve unvaccinated of ages 18–29 during October–November 2021. Using the data in the preceding paragraph, in particular assuming among the unvaccinated having prior infection a 21.8-fold lower case rate than the infection-naïve unvaccinated, (where 21.8 is approximately the average of 29 and 14.7) we have

$$r \cdot 406{,}000 \; + \; r \cdot 594{,}000/22.8 \; = \; 28{,}770,$$

which yields $r \approx 0.0666$. Thus, during October–November 2021, we estimate that $0.0666 \cdot 406{,}000 \approx 27{,}040$ cases of COVID-19 were reported for infection-naïve unvaccinated persons in our test population of 100,000.

Assuming the CDC's case-to-infections multiplier of 4 from [2] is still valid through November of 2021, 27,040 cases would correspond to 108,160 infections among the infection-naïve unvaccinated. Thus, at the end of November, our test population of 1 million unvaccinated persons would have only 406,000-108,160= 297,840 that are infection-naïve. In other words, as of the end of November 2021, the infected fraction of our test population is 0.70216.

We now estimate how many infection-naïve persons in our test population became infected during December 2021. On 12/1/2021, we are assuming the 1 million members of the test population would comprise 297,840 that are infection naïve and 702,160 that have had a prior COVID infection. We assume based on data from [32] discussed above that 33,030 cases of COVID-19 would occur in this group in the month of December. We seek to estimate how many of the 33,030 cases occur among the infection-naïve unvaccinated.

Let r be the COVID-case rate among the infection-naïve unvaccinated in our test population during December of 2021. Using the data in the preceding paragraph, relying on our work of the next section estimating that prior infection provides protection against Omicron reinfection with 47% effectiveness, and assuming protection against reinfection well approximates protection against cases among the prior infected, we have

$$r \cdot 297{,}840 \; + r \cdot 702{,}160 \cdot (1 - 0.47) = 33{,}030,$$

from which we obtain $r \approx 0.0493$. Thus, during December 2021, we estimate that $0.0493 \cdot 297{,}840 \approx 14{,}680$ cases of COVID-19 were reported for infection-naïve unvaccinated persons in our test population.

Continuing to assume the CDC's case-to-infections multiplier of 4 is still valid through December of 2021, 14,680 cases would correspond to 58,720 infections among the infection-naïve in our (unvaccinated) test population. Thus, at the end of December, our test population of 1 million unvaccinated persons would have only 297,840-58,720 = 239,120 persons that are infection-naïve. In other words, as of the end of December 2021, we estimate that in our test population of 1 million 18–29 year-olds the fraction having had a COVID-19 infection is 0.76088, which we round to 0.76 and we assume this would well approximate the infected fraction for a test population of 1 million 18–25 year-olds (general-population mix of males and females) as of 1/1/22.

If we assume there is no difference in infection rates between males and females, then we have $F_{pi} = 0.76$ as an estimate of the fraction of males, 18–25, having been infected with COVID-19 before 1 January 2021, and we use this value of F_{pi} in a sensitivity analysis (Supplement S2, Section S2.3). Here, we derive a value of F_{pi} assuming females are 1.21 times as likely as males to be COVID infected.

According to U.S, Census data [30], in 2021 males constituted about 51.1% of the U.S. population of ages 18–25 while females constituted 48.9%. Thus, in 2021 a representative group of 1 million U.S. residents 18–25 years old would comprise 511,000 males and 489,000 females. Assuming a male prior-infected fraction of x and a female prior infection factor of $1.21x$ and that 760,000 have had a prior infection, we have

$$511{,}000 \cdot x \; + \; 489{,}000 \cdot 1.21 \cdot x \; = \; 760{,}000,$$

So that $x \approx 0.69$. Thus, in Scenario D, our most likely scenario, as well as in Scenario E, we assume $F_{pi} = 0.69$. For Scenarios A–C, we use a more conservative estimate—the average of the end-of-September CDC estimate of 0.549 (males and females, vaccinated and unvaccinated, ages 18–49) and our end-of-December estimate of 0.69 for unvaccinated males 18–25 $(0.549 + 0.69)/2 \approx 0.62$.

We remark that seroprevalence data cannot be used to obtain a reasonable estimate for F_{pi}. Consider, e.g., the following from [34]:

> It is particularly challenging to determine the true proportion of the population that has been previously exposed to SARS-CoV-2. … [S]erological studies will inevitably under-estimate the number of people exposed, since some will have a lower antibody count when the study is conducted and test negative.

We also note that studies, such as [33], [35], and [36], quantifying the protection of prior infection against reinfection are based on a history of prior infection rather than on persons having detectable levels of antibodies against the SARS-CoV-2 nucleocapsid protein.

S4. Estimating E_{pi}, E_v, and E_h

Overview. Recall that the FDA used VE = 0.30 and VEH = 0.72 for all its Omicron-based scenarios [1, Table 1]. For reasons discussed in the next section of this supplement ("Estimating HRR_{pi}, HRR_v, and HRR_h"), for all our scenarios, we use $E_v = 0.3$ and $HRR_v = 0.67$. Our values $E_{pi} = 0.45$ and $E_h = 0.57$ are derived from a December 2021 study [37] based on data from South Africa.[16]

Detailed Discussion. In the main exposition, we cited studies (e.g., [33], [35], [40]) as well as a CDC science brief [41] indicating that prior infection provides protection roughly equivalent to or superior to vaccination and asserted immunological considerations suggest that prior infection would likely provide greater protection against Omicron than vaccination among the COVID-naïve.[17] We focus on identifying values for E_{pi} and E_h based on data available by the end of December 2021.

We rely on data from South Africa available 14 December 2021, appearing in a press release [37] headlined as follows: *Discovery Health, South Africa's largest private health insurance administrator, releases at-scale, real-world analysis of Omicron outbreak based on 211 000 COVID-19 test results in South Africa, including collaboration with the South* African Medical Research Council (SAMRC) *on vaccine effectiveness.*

Discovery Health's analysis found that "vaccinated individuals who received two doses of the Pfizer-BioNTech vaccine had 33% protection against infection, relative to the unvaccinated, in the first weeks of South Africa's Omicron-driven fourth wave" and that "vaccinated individuals who received two doses of the Pfizer-BioNTech vaccine had 70% protection against hospital admission in this same time period." Note that the 70% VEH is consistent with the 72% VEH found in Table 6 of the UK Health Security Agency technical briefing [21], released 31 December 2021, based on data from England that combined effectiveness estimates of all three vaccines used there (AstraZeneca's, Pfizer's, and Moderna's).

Discovery Health's analysis also found

> People who were infected with COVID-19 in South Africa's third (Delta) wave face a 40% relative risk of reinfection with Omicron. People who were infected with COVID-19 in South Africa's second (Beta) wave face a 60% relative risk of reinfection with Omicron. While individuals who had a documented infection in South Africa's first wave, and therefore were likely to have been infected with the SARS CoV-2 virus carrying the D614G mutation, face a 73% risk of reinfection relative to those without prior documented infection.

The preceding estimates of prior-infection protection derive from testing performed over the period from 11/15/21 to 12/7/21 "during which Omicron infections were increasing dramatically" [37]. Observe that protection conferred by an infection wanes as the time from the infection to the testing period increases. To account for waning of protection in the analysis to follow, we will assume that the relative-risk reductions described in the preceding paragraph are valid as of 11/15/21, the first day of the testing period, which is a choice that is both convenient and conservative. It is convenient in that South Africa's third (Delta) wave began 6 months before 11/15/21 while its second (Beta) began 12 moths before. It is conservative because choosing a later date, say, 12/7/2021, would suggest slightly more durable protection conferred by prior infection, e.g., with the choice 12/7/2021, the level of "third wave protection" against reinfection (60%

[16] Our effectiveness estimates involving prior infection are consistent with those of meta-analyses [38] and [39].

[17] In Section S7, we present strong evidence, based on meta-analyses and systematic reviews incorporating evaluation-period data, that prior-infection protection was superior to two-dose vaccination protection among the COVID-naïve during the evaluation period

effective) would extend to about 6.5 months beyond the wave rather than to about 6 months (corresponding to our choice of 11/15/21).

Below is a depiction of the first three waves of COVID-19 infection in South Africa from a 12/2/21 preprint [42].[18]

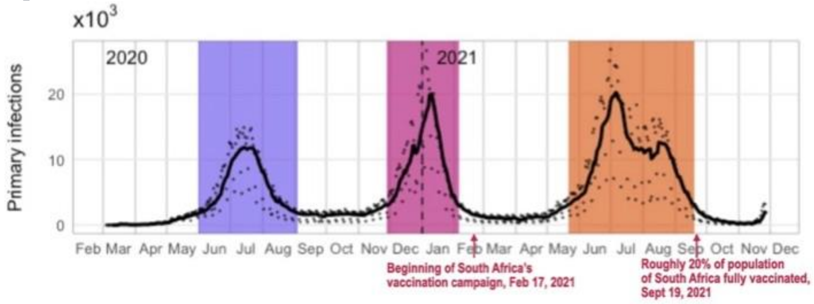

First Three Waves of SARS-CoV-2 Infection in South Africa

Worldometer [44] reports (based on data from South Africa's National Institute for Communicable Diseases) 603,252 cases during South Africa's first wave (which we approximate, based on the preceding figure, as the period 5/31/20 – 9/2/20); 679,394 cases during its second wave (11/30/20 – 2/3/21); and 1,255,386 cases during its third wave (5/20/21 through 9/15/21). This suggests that the following weighted average of relative risks captures the overall risk of reinfection among the prior infected relative "to those without prior documented infection":

$$\frac{603252}{2538032} \cdot 0.73 + \frac{679394}{2538032} \cdot 0.60 + \frac{1255386}{2538032} \cdot 0.40 \approx 0.53.$$

The preceding corresponds to an effectiveness level of 47% for prior infection's preventing Omicron reinfection.[19] As discussed above, we assume 47% measures overall effectiveness as of 11/15/21. We might have directly computed the effectiveness estimate as a weighted average of effectiveness levels corresponding to each wave:

$$(\dagger) \quad \frac{603252}{2538032} \cdot 0.27 + \frac{679394}{2538032} \cdot 0.40 + \frac{1255386}{2538032} \cdot 0.60 \approx 0.47.$$

We remark that in obtaining the preceding section's estimate of $\boldsymbol{F_{pi}}$**,** we used this 47% effectiveness estimate to approximate the additional number of infection-naïve unvaccinated 18–29 year-olds that became infected during December of 2021 in the U.S. Our analysis (discussed below) suggests 47% may be a slight underestimate for the overall level of prior-infection protection in the U.S. in December 2021 (and had we used a higher estimate, then our $\boldsymbol{F_{pi}}$ estimate would have been larger as well, lowering the benefits of vaccination for the general population of 18-25-year-old males in the U.S.).

In order to use the data from South Africa discussed above to account for the waning of prior-infection protection over our evaluation period, we must provide estimates of effectiveness of protection that vary continuously with time since infection. We do this by assuming protection declines linearly over time, which yields, in a natural way, the polygonal curve plotted below, providing estimates of the effectiveness of protection conferred by prior infection based on the time since infection. For each triple of points on the curve, the left and right points correspond to a wave's approximate beginning and ending points, respectively, and the point between the left and right locates the "half-case point" of the corresponding wave—the approximate date for which half of the cases of the wave occurred on or before the date. In the

[18] [42] was later published in *Science* [43].

[19] The 47% effectiveness estimate is roughly consistent with estimates of protection against any Omicron infection from the meta-analyses [38] and [39] (above 50% through 6 months in [38] and through 25 weeks in [39] and increasing toward protection at a level of 75% or above within the first few weeks after an infection). The Discovery-Health report [37] does not indicate whether the analysis of prior-infection protection is restricted to only unvaccinated individuals. Disregarding vaccination status might result in an overestimation of prior-infection protection (among the unvaccinated). However, in the relevant period vaccination coverage in South Africa was limited. According to Table 3 of [45], at the end of December of 2021 only 26.3% of South Africa's population had been fully vaccinated against COVID.

plot, the slope of the segment to the left of the half-case point for the Second Wave is $\frac{13}{172}$ while that to the right is $\frac{2}{19}$. Note that at end of the third wave (9/15/21) the effectiveness level reaches 66.6% and we, conservatively, cap effectiveness at this level and assume it holds until the beginning of the testing period 11/15/2021. Had we continued the line with slope 2/19 over the interval from 9/15 to 11/15, then the implied effectiveness for an infection occurring 11/15/21 would have been 73%.[20]

A polygonal curve providing an extension of effectiveness estimates for prior-infection protection against omicron infection in South Africa where effectiveness is assumed to be as of 11/15/21*

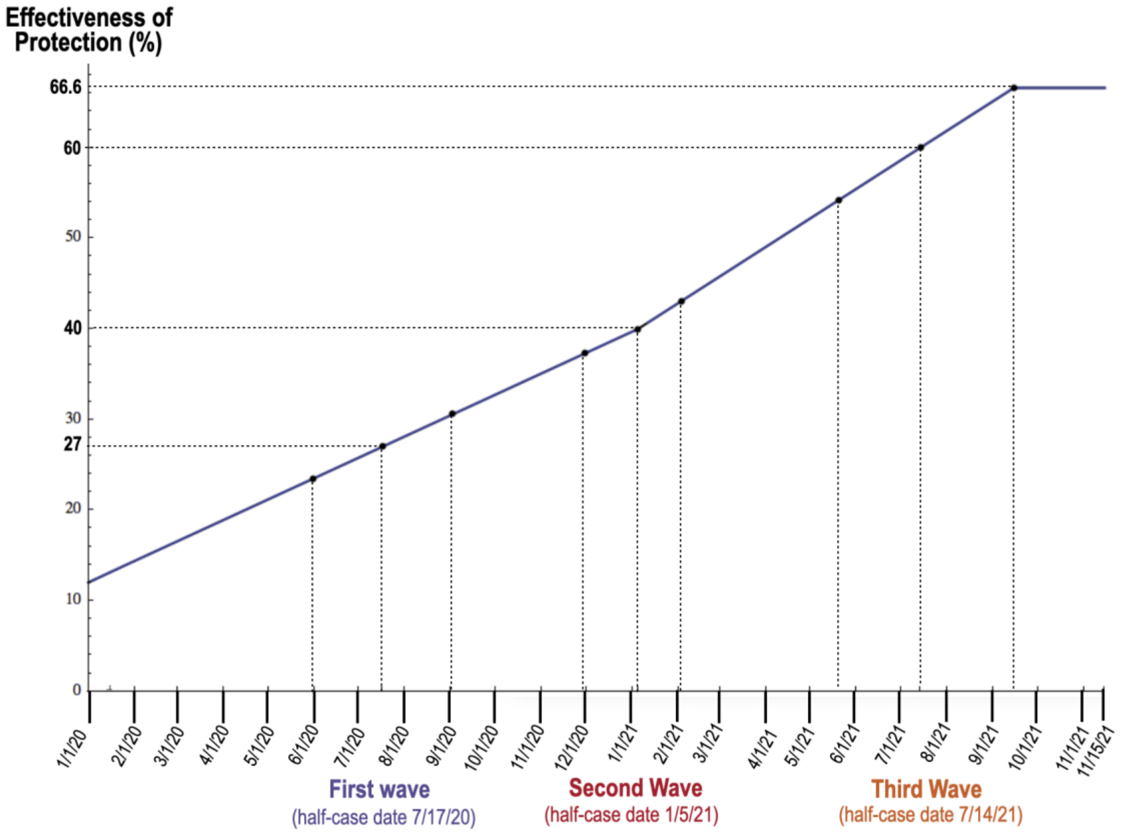

* Examples: For a person infected by COVID on the Third-Wave half-case date 7/14/21 who does not experience or reinfection after that point, the preceding plot indicates the person has 60%-effective prior-infection protection as of 11/15/21. Suppose a person is infected on 9/1/21 (tests positive that day); because 9/1 is 49 days after the Third Wave case half-point, 7/14/21, then the level of protection we assume the person has as of 11/15/21 is $60 + (2/19)49 \approx 65.2\%$.

For future reference, we provide a formula for the function PD whose graph is the blue polygonal curve in the preceding plot. Enumerating the days from January 1, 2020 (day 0 of a leap year) to December 31,2025 (day 730) and noting that day 370 corresponds to the Second Wave half-case point 1/5/21 while day 560 corresponds to the Third Wave half-case point 7/14/21, we have

$$PD(t) = \begin{cases} (13/172)(t-370) + 40, & t \leq 370 \\ (2/19)(t-560) + 60, & 370 < t < 623 \\ 66.6 \quad , & t \geq 623. \end{cases}$$

For example, the effectiveness of prior-infection protection, as of 11/15/21, for someone infected on day $t = 152$, 1 June 2020, and not reinfected, is

$$PD(152) = \frac{-13 \cdot 218}{172} + 40 \approx 23.5\%$$

---

[20] The estimate that a recovery from a very recent infection provides 73% effective protection against re-infection for a short period of time is consistent with estimates of meta-analyses [38] and [39] (in which estimates of effectiveness increase toward protection at a level of 75% or above within the first few weeks after an infection). .

Let G denote a representative group of 1 million unvaccinated 18–25-year-old persons in the United States formed at the beginning of the evaluation period 1/1/22. In the preceding section, we estimated that about 76% of such a group would have been infected by COVID before 1/1/22. We now turn to estimating the level of prior-infection protection against Omicron we would expect members of G to have both at the beginning of the evaluation period and at the end. Members of G constitute a mix of males and females. We assume that the estimate of prior-infection protection we arrive at applies to males 18–25.

Below is a depiction of daily COVID case totals during the pre-evaluation period (from the Johns Hopkins Coronavirus Resource Center [46]) together with the polygonal curve we will use to estimate prior-infection protection against Omicron in the U.S. as of 1/1/21 and as of 5/31/2021 for our representative group G of 1 million unvaccinated persons.

Estimated prior-infection effectiveness against Omicron infection based on time from infection to 1/1/21,* together with a plot of daily COVID case totals during the pre-evaluation period

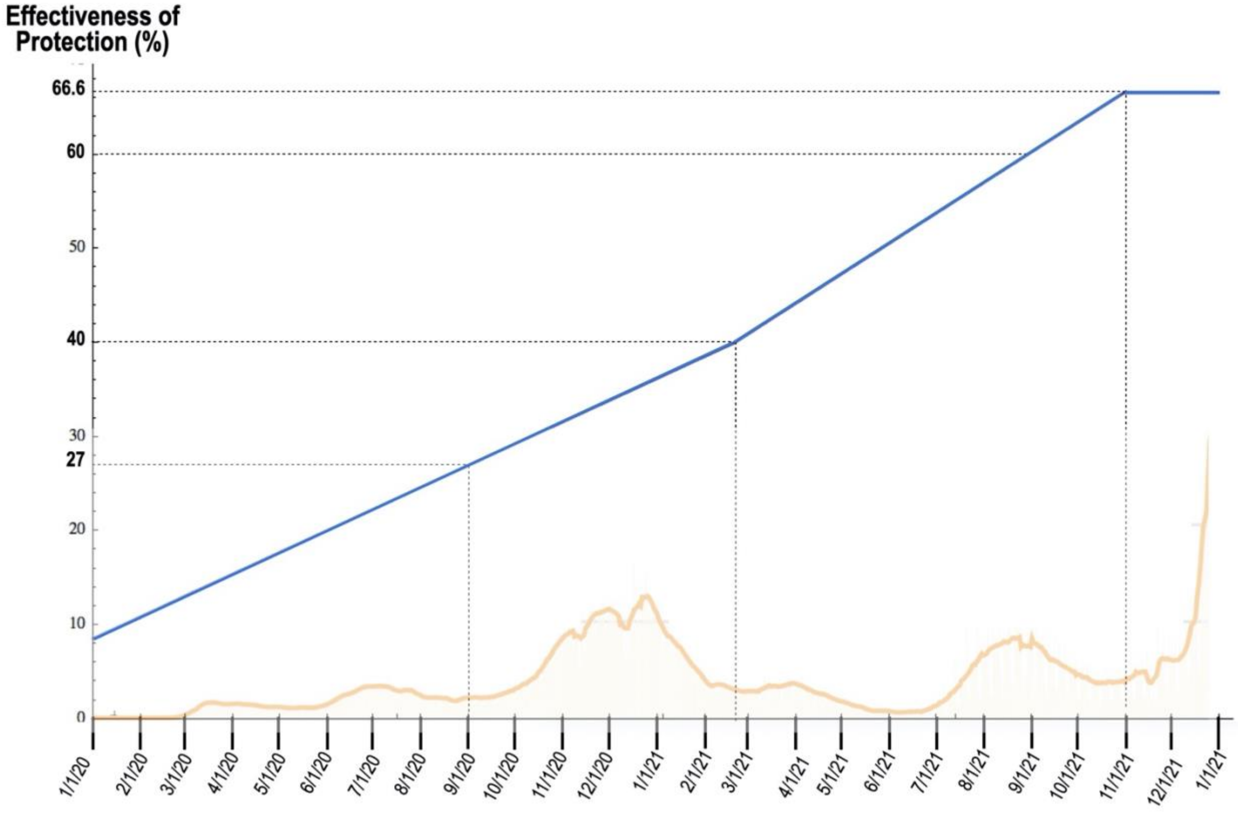

*For example, according to the blue polynomial curve providing effectiveness estimates, a person infected 11/1/21 (and not reinfected after that date) will have approximately 66.6% effective protection against reinfection (by Omicron), as of 1/1/21.

The function whose graph is the blue polygonal curve in the plot above is given by $USPD(t) = PD(t-47)$, where PD is defined on the preceding page.

The figure above shows there were many new cases in U.S. during the three months leading up to the beginning of the evaluation period, especially in late December 2021 This is quite unlike the corresponding situation in South Africa, where we have estimated prior-infection protection as of 11/15/21, which marks the end of a roughly two-month period of relatively low infection prevalence. With so many of its members having highly effective protection as of 1/1/22, resulting from infections occurring close to this date, we anticipate that the overall level of protection infected members of G will have as of 1/1/22 will be higher than that, as of 11/15/21, of a similarly formed group in South Africa. We assume the South African group would have approximately 47% effective protection, as of 11/15/21 based on the data of [37].

Applying the effectiveness-levels suggested by the curve of the figure above, we estimate in Appendix S4 of this supplement that the average level of protection infected members of G have is approximately 52% at the start of the evaluation period and approximately 39% at its end, which yields an average level of evaluation-period protection of 45.5%. We, conservatively, round down, so that for Scenarios A–D, we use $E_{pi} = 0.45$, and for Scenario E, $E_{pi} = 0.30$.

We turn now to estimating the effectiveness of hybrid protection from infection E_h. It is natural to assume that $E_h \geq E_{pi}$ and that an upper bound for E_h would be obtained by assuming that vaccination and prior infection independently provide protection (over the evaluation period) with effectiveness 30% and 47%, respectively (use of 45% yields a slightly lower estimate of hybrid protection), so that fully vaccinated persons with prior infection would have a 37% ($\approx 0.7 \cdot 0.53$) risk of reinfection relative to the infection-naïve unvaccinated. With these assumptions, we have $0.47 \leq E_h \leq 0.63$, and averaging the bounds, we'd have $E_h \approx 0.55$.

Alternatively, there is evidence in references such as [33], based on CDC data from New York and California, May–November 2021, that hybrid protection reduces the risk of Delta infection relative to the unvaccinated having prior infection. Using Table 2 from [33], one can average for the period July 4-November 20 the hazard ratios for Delta infection for an unvaccinated, infection-naïve person relative to an unvaccinated person having prior infection, yielding 30.22 for CA and 18.18 for NY. The same hazard-ratio averages relative to a person with hybrid protection are 38.76 for CA and 21.38 for NY. Thus, in CA, the risk of infection for a person having hybrid protection relative to an unvaccinated person with prior infection would be approximately $30.22/38.76 \approx 0.78$; in NY, the relative risk would be $18.18/21.38 \approx 0.85$. The average of these relative risks is 0.815.

We have estimated, based on data from South Africa, the risk of infection for an unvaccinated person with prior infection relative to an infection-naïve unvaccinated person to be 0.53. Thus, if we assume that hybrid protection against Omicron carries the same relative-risk benefit as that against Delta, we then obtain the following estimate for the risk of an Omicron infection for a person with hybrid protection relative to an infection-naïve unvaccinated person: $0.815 \cdot 0.53 \approx 0.43$. Thus, in our modeling, we will take $E_h = 1 - 0.43 = 0.57$.[21]

S5. Estimating HRR_{pi}, HRR_v and HRR_h

Overview. Our estimate $HRR_v = 0.67$ derives from a UK HSA Technical Briefing [21, Table 6 (Dose 2, 2–24 weeks)] and we show below that this estimate agrees with one based on data from [18] based on Omicron cases in England for the period 12/1/21–12/14/21. We also use data from [18] to estimate $HRR_{pi} = 0.79$ and $HRR_h = 0.88$.[22]

Detailed Discussion. We derive possible values of hospitalization-risk reduction among the Omicron infected based on data from Report 50 of the Imperial College COVID-19 response team [18]. This report, released 22 December 2021, is based on all PCR-confirmed SARS-CoV-2 cases in England with last test specimen dates between the 1st and 14th December 2021 inclusive. Here's a portion of Table 3 of [18] providing raw data in columns 4 and 5, with estimates in columns 6, 7, 8, adjusted to correct for under-ascertainment of reinfections (annotations in blue added):

[21] This choice is roughly consistent with [38, Table 2], which reports the effectiveness of hybrid immunity against reinfection to be 69.0% (58.9 to 77.5) at 3 months, dropping to 60.4% (49.6 to 70.3) at 6 months, to 51.1% (40.2 to 61.9) at 9 months and to 41.8% (31.5 to 52.8) at 12 months.

[22] As noted in the main manuscript, the estimates $HRR_{pi} = 0.79$ and $HRR_h = 0.88$ combined with our estimates $E_{pi} = 0.45$ and $E_h = 0.57$ yield overall estimates for protection against hospitalization—88.5% for prior infection and 94% for hybrid protection---consistent with those of meta-analyses [38] and [39].

Table 3. Estimates of the hazard ratio (HR) for hospital attendance for Omicron vs Delta cases and for reinfections vs primary infections, stratified by vaccination status. The percentage of cases and hospitalisations that were reinfections is also shown. Uncorrected estimates are generated via conditional Poisson regression. Corrected estimates (only the mean estimates for Omicron are shown) adjust for under ascertainment of reinfection, assuming 1/3 of all infections are detected through community surveillance. 95% confidence intervals are shown in parentheses for the uncorrected estimates. D1, D2 and D3 categories are post-dose 1, 2 and 3, respectively. D3 categories all received a mRNA booster and are distinguished by the dose 1/2 vaccine used. Numbers in category names (14, 21) refer to days since last dose.

	Variant	Vaccination or reinfection category	Cases	Hospital-isations	% Reinfections (cases)	% Reinfections (hosp)	HR relative to primary Delta infection in unvaccinated	p-value
Corrected	All	Not reinfection	277847	3164	-	-	-	-
	All	Reinfection	42309	127	15.2%	3.9%	0.42	-
	Omicron	Unvaccinated	9585	56	56.4%	21.1%	0.76	-
	Omicron	AZ:D1:<21	0	0	-	-	Uncorrected: 18.6% & 8.9%,	
	Omicron	AZ:D1:21+	257	≤5	70.7%	0.0%	respectively	
	Omicron	AZ:D2:<14	29	0	10.4%	-	-	-
	Omicron	AZ:D2:14+	11440	46	42.5%	11.1%	0.37	-
	Omicron	AZ:D3:<14	2877	≤5	27.3%	0.0%	0.07	-
	Omicron	AZ:D3:14+	2384	8	21.1%	0.0%	0.21	-
	Omicron	PF/MD:D1:<21	293	0	43.4%	-	-	-
	Omicron	PF/MD:D1:21+	2526	10	39.1%	52.0%	0.66	-
	Omicron	PF/MD:D2:<14	249	≤5	62.1%	0.0%	0.59	-
	Omicron	PF/MD:D2:14+	22249	60	36.1%	17.6%	0.26	-
	Omicron	PF/MD:D3:<14	780	≤5	29.5%	67.9%	Uncorrected: 11.9% & 6.7%,	
	Omicron	PF/MD:D3:14+	2467	11	24.3%	25.3%	respectively	

Data in the preceding table for the unvaccinated constitutes a small sample. It is chosen for analysis here because it was available for the FDA to consider for its mRNA-1273 benefit-risk assessment.[23] The data provides the following case hospitalization rate CHR for the unvaccinated not having had prior COVID-19 infection:

$$\mathrm{CHR} = \frac{56(1-0.221)}{9585(1-0.564)} \approx 0.01057.^{24}$$

It's important to note that the authors of Report 50 did try to limit the number of incidental hospitalizations counted as COVID-19 hospitalizations:

> [A] substantial proportion of hospitalisations attributed to COVID-19 may be incidental; all persons admitted to UK hospitals are currently routinely tested for SARS-Cov-2 infection and testing positive may be unrelated to the clinical reason for admission. We mitigated this potential issue by restricting our analysis to individuals with last specimen dates on or before the day of hospital attendance, and for our primary analysis to cases who had a positive test via the UK "Pillar 2" community surveillance programme, which is distinct from the "Pillar 1" hospital testing system.

Thus, we will not adjust the CHR computed above for incidental hospitalizations.

The CHR for the unvaccinated having had a prior infection is

$$\frac{56 \cdot 0.211}{9585 \cdot 0.564} \approx 0.00219.$$

Thus, the risk of hospitalization of the unvaccinated with prior infection relative to those without is given by

$$\frac{0.00219}{0.01057} \approx 0.207,$$

so that the hospitalization-risk reduction provided prior infection is about 79.3%; that is, we have some evidence supporting scenarios for which $HRR_{pi} = 0.793$. Unfortunately, the hospitalization risk ratio of 0.207 yielding an estimate of HRR_{pi} derives from a small sample. Report 50 mentions that the mean age of those hospitalized for Omicron is 30.8 years; so, some data from Report 50 is relevant to risk in younger persons. The question here is: does prior infection provide a fairly uniform hospitalization-risk reduction across age groups? If so, the estimate $HRR_{pi} = 0.793$ is more likely to be accurate. We use

[23] We also assume that the FDA could have sought additional data like that of [18, Table 3] from the UK Health Security Agency.

[24] We remark this CHR is consistent with our estimate of R = 0.0022 for the *infection* hospitalization rate for those 18–25: Report 50 [18, p. 10] states, "Across the whole epidemic, it is likely that fewer than a third of infected individuals were tested through the country's `Pillar 2' community surveillance system." Thus, we assume that every case represents at least 3 infections and obtain an upper IHR estimate of 0.01057/3 = 0.0035 and would expect a significantly lower rate with a restriction to the age range 18–25.

$HRR_{pi} = 0.79$ in our hospitalizations modeling (except for Scenario E, for which we assume prior-infection protection is equivalent to two-dose vaccine protection among the COVID-naïve).

Data from Report 50's Table 3 (above) also allows a computation of hospitalization risk reduction provided by vaccination alone (no prior infection) and by hybrid protection. The CHR for the vaccinated (PF/MD:D2:14+) without prior infection is

$$\text{CHR} = \frac{60(1-0.176)}{22249(1-0.361)} \approx 0.00348.$$

Thus, the risk of hospitalization for the vaccinated without prior infection relative to the unvaccinated without prior infection is

$$\frac{0.00348}{0.01057} \approx 0.329,$$

so that the hospitalization-risk reduction provided by vaccination is about 67.1% In our modeling, we will assume

$$HRR_v = 0.67.$$

It is interesting to note that this risk reduction is the same as that found in the UK technical briefing [21] on which the FDA relied. In Section 3 of [21] "Vaccine Effectiveness" in the context of a Cox survival analysis "used to estimate the risk of hospital admission by vaccination status," the briefing states "Adjustments were made for age, gender, previous positive test, region, ethnicity, clinically extremely vulnerable status, risk group status and period." Note, an adjustment is made for "previous positive test," but the nature of the adjustment is not described and no separate effectiveness estimate is provided for hybrid protection against hospitalization. Even if hospitalizations are tracked among the vaccinated and unvaccinated without regard to prior-infection status, removing those with prior infection in both groups (to obtain an estimate of HRR_v) would mean increased case-hospitalization rates for both the vaccinated and unvaccinated. And both rates could increase in such a way that their ratio remains essentially unchanged.

The CHR for the vaccinated (PF/MD:D2:14+) with prior infection is

$$\text{CHR} = \frac{60 \cdot 0.176}{22249 \cdot 0.361} \approx 0.00131.$$

Thus, the risk of hospitalization for the vaccinated with prior infection relative to the unvaccinated without prior infection is

$$\frac{0.00131}{0.01057} \approx 0.124,$$

which yields a hospitalization-risk reduction provided by vaccination and prior infection of about 87.6%. Thus, in our modeling, we take $HRR_h = 0.88$.

The hospitalization-risk reductions estimated above apply to those who have contracted a COVID-19 infection and provide estimates of the risk of progression to severe (Omicron) disease (relative to the unvaccinated experiencing a primary infection). As discussed in the main exposition, to achieve overall estimates the hospitalization-risk reduction in the general population—e.g., vaccinated with prior infection vs. unvaccinated without prior infection—we must combine the risk-reduction of infection with the corresponding hospitalization-risk reduction among those infected. For example, the overall risk to those with hybrid immunity of both contracting an infection and having it lead to hospitalization relative to those who are unvaccinated without prior infection is $(1 - E_h)(1 - HRR_h) = 0.43 \cdot 0.12 \approx 0.052$, yielding an overall effectiveness against hospitalization of 94.8%.[25] The overall risk of hospitalizations for those who are unvaccinated with prior infection relative to the infection-naïve unvaccinated is $(1 - E_{pi})(1 - HRR_{pi}) = 0.55 \cdot 0.21 \approx 0.1155$, yielding an overall effectiveness against hospitalization of 88.5%.[26] As discussed in the main exposition, the assumptions that $E_v = 0.30$ and $HRR_v = 0.67$ yield an overall effectiveness against hospitalization for the COVID-naive fully vaccinated of about 77%. Thus, data available in December of 2021 suggests that for Omicron, just as for Delta (see [33] and [36]), prior

[25] In good agreement with data from [38, Table 2].

[26] In nearly perfect agreement with the estimate of 88.9% at 40 weeks against BA.1 found in [39] and a little higher than the estimates of [38, Table 2], e.g., 82.5% at 3 months, 80.1% at 6 months, and 74.6% at 12 months.

infection provides greater protection against hospitalization than does two-dose vaccination among the COVID-naïve.

We conclude this section by discussing further our choice of 0.30 for E_v In [1], the FDA assumed that primary-series mRNA-1273 vaccination would provide 30% effective protection against Omicron *cases*; however, the source to which the 30% estimate is attributed considers mRNA-1273 effectiveness against *symptomatic Omicron infection* [21, Figure 2C], and Figure 2C does suggest that the average effectiveness of mRNA-1273 against symptomatic infection is about 30% during the five month period beginning two weeks after dose 2. Thus, we interpret the FDA's assumption of 30% effective protection against cases to be equivalent to an assumption of 30% protection against symptomatic infection.

In our model, E_v represents the effectiveness of vaccination alone against *all infection* relative to the infection-naïve unvaccinated. Typically, vaccine effectiveness against symptomatic infection has been found to be higher than that against all infection. For example, Moderna's clinical-trial report of data up to the end of the blinded phase [47] found that mRNA-1273 provided 82% effective protection against any infection and 93.2% effective protection against symptomatic infection, while [48] found that mRNA-1273 provided 56.6% effective protection against all infection and 84.2% effective protection against symptomatic infection.[27]

We will not make any reduction to $E_v = 0.30$ based on its measuring protection against all infection. We also will not make adjustments to $E_v = 0.30$ relating to its measuring the protection from vaccination alone (no prior infection) *relative to the infection-naïve unvaccinated*. The FDA assumed, based on [21], that mRNA-1273 vaccination would reduce the risk of symptomatic infection by 30% relative to the unvaccinated, without regard to prior-infection status among the vaccinated or unvaccinated. Removing those with prior infection from among the vaccinated and unvaccinated would increase infection incidence in both groups, potentially having little effect on the effectiveness estimate. Further justification for not increasing $E_v = 0.30$ comes from studies [50] and [51] of vaccine protection against Omicron whose findings were announced 12/14/21 and 1/1/22, respectively, with the former's conclusion being "Primary immunisation with two BNT162b2 or ChAdOx1 doses provided no or limited protection against symptomatic disease with the Omicron variant" and the latter's, "Two doses of COVID-19 vaccines are unlikely to protect against infection by Omicron."[28]

Yet another early study [55] of vaccine effectiveness against Omicron, released 12/16/21, reports in Table 2 that 14+ days after dose 2, BNT162b2 vaccination (among the COVID naïve) provides protection from Omicron symptomatic infection with negative effectiveness -18% (-26, -11). However, [55] attempts to estimate vaccine effectiveness against Omicron relative to vaccine effectiveness against Delta and the method is extremely sensitive to effectiveness-against-Delta estimates. For example, Table 3 of [55] reports 19% effective protection for BNT162b2 against Omicron symptomatic infection if vaccine effectiveness against Delta is assumed to be 69.8% (instead 55.9%, as assumed in Table 2 of [55], which yields -18% effectiveness against Omicron). These estimates of protection against Omicron depend on the

---

[27] Relative to the Omicron variant, estimates of Table 3 of [49] suggest that mRNA-1273 vaccination had 55% effectiveness against symptomatic infection during the 3-month period following dose 2, but only 30% effectiveness against all infection during the same period.

[28] The studies [50] and [51] were later modified ([52] and [53], respectively) with different study periods providing larger numbers of Omicron cases. Modifications led to new findings consistent with the FDA's assumption that mRNA-1273 would provide 30% protection against *symptomatic* Omicron infection during the evaluation period. However, the studies [51] and [53] had an additional significant difference: the original study [51] tested effectiveness of vaccination against infection, regardless of symptoms, *finding vaccination had statistically significant negative effectiveness against Omicron infection 120–238 days postdose 2* while [53] found effectiveness against *symptomatic infection* to be 15% (8%, 27%) 120–179 days postdose 2 and 1% (-8%, 10%) 180–239 days postdose 2. On the other hand, the published version [52] of [50] found that the effectiveness of two doses of mRNA-1273 *against symptomatic infection* declined from 75.1% (70.8, 78.7) from 2 to 4 weeks after dose 2 to 14.9% (3.9 to 24.7) after 25 or more weeks, with data from Table 3 of [52] suggesting, for the 5-month period starting two weeks after dose 2, mRNA-1273 vaccination provides an average effectiveness against symptomatic Omicron infection of approximately 36% while BNT162b2 vaccination provides approximately 30% average effectiveness. Another study (based on data not available in early January 2022) suggests that $E_v = 0.30$ may be an overestimate: the study [54] provides the following estimates of protection provided by full vaccination (2 doses of BNT162b2) and full vaccination plus a booster (3 doses of BNT162b2) *relative to infection-naïve unvaccinated persons*: "Assuming VE took full effect 7 days after vaccination, we estimated: (1) VE for the **second**, third or fourth doses of BNT162b2 **were 13% (95% credible interval: 2–39%)**, 48% (34–64%) and 69% (46–98%) 7 days following immunization, respectively, **waning to 7% (1–21%)**, 26% (7–41%) and 35% (10–71%) 100 days after immunization." The preceding are estimates of vaccine effectiveness against all infection, not just symptomatic infection.

hazard ratio 2.68 (2.54, 2.83) for Omicron infection relative to Delta infection reported in Table 2 of [56] for those ≥ 14 days past dose 2 of BNT162b2.

The study [55, p. 7] also reports a hazard ratio of 6.36 (5.32, 7.74) for Omicron reinfection relative to Delta reinfection among the unvaccinated. We use this ratio of 6.36 along with the ratio 2.68 relating to BNT162b2 vaccination (among the COVID naïve) to illustrate further the sensitivity of the Omicron-protection estimates of [55] to variation in Delta-protection estimates. Averaging hazard-ratio estimates of Table 2 of [33] from 9/26/21 through the end of the study period 11/20/21, we obtain hazard ratios of Delta infection among the COVID-naïve unvaccinated vs. the unvaccinated with prior-infection protection: 29.7 for California and 19.4 for New York. Corresponding average hazard ratios of Delta infection among the COVID-naïve unvaccinated vs. COVID-naïve mRNA fully vaccinated persons are 7.2 and 5.2. Combining the preceding hazard ratios with the ratios 6.36 and 2.68 ([55, p. 7 & Table 2]) and assuming that vaccination means mRNA-vaccination (as does [33]), we arrive at the following

- Vaccine effectiveness (among the COVID naïve) against Omicron infection 14+ days past dose 2 is 48.5% (NY data) and 62.8% (CA data).[29]
- Prior-infection effectiveness against Omicron infection is 67.2% (NY data) and 78.6% (CA data).[30]

S6. Estimating the VAM/P Rate for mRNA-1273

Overview. In this section, we provide data and analysis supporting our VAM/P-rate estimates of Table 2 and Section 1.4 of the main exposition.

Detailed Discussion. We begin by discussing VAM/P rates for dose 2 of mRNA-1273. We continue to assume that VAM/P rates for males 18–24 well approximate those for males 18–25.

S6.1 Approximation of Second-Dose Rate Using the FDA's Estimate of VAM/P Risk for BNT162b2 and VSD-based Risk Ratios, mRNA-1273 vs. BNT162b2. The FDA suggests in its mRNA-1273 assessment [1] that for 18–25-year-old males VAM/P risk for mRNA-1273 is comparable to that for BNT162b2: 128 cases per million 2nd doses of mRNA-1273 [1, Table 4]} vs. 131 cases per million full vaccinations of BNT162b2 (among 18–24-year-old males [9, Table 3], with corresponding hospitalization projections of 110 per million 2nd doses of mRNA-1273 and 131 per million full BNT162b2 vaccinations.

Adjusted-rate ratios for 18–39 year-olds reported in the table on page 25 of [56] suggest that the 2nd-dose VAM/P rate for Moderna's mRNA-1273 is 2.72 times that of Pfizer's BNT162b2 and that the corresponding ratio for the 1st dose is 2.63. For 18–39-year-old *males*, the tabulated data on page 27 of [56] suggests a 2nd-dose rate ratio of 2.26, and, if we assume the same percentage decrease for the 1st dose, then that rate ratio would be 2.19 instead of 2.63. As indicated in the main exposition, data from other sources suggests higher rate ratios (at least for dose 2).

Based on data from the preceding two paragraphs, we now compute a VAM/P-rate estimate for mRNA-1273 using the FDA's corresponding estimate for BNT162b2 from [9] and the VSD VAM/P rate ratios from [56]. FDA data available in October 2021 reports 5 myo/pericarditis events postdose 1 of BNT162b2 and 36 postdose 2 [57, p. 14]. Assuming the same ratios of dose 1 and dose 2 events are reflected in the FDA's projected 131 VAM/P cases per million Pfizer's BNT162b2 vaccinations [9, Table 3], we find the corresponding estimate for the VAM/P rate for mRNA-1273 among males 18–25 to be

$$\frac{5}{41}\cdot 131\cdot 2.19+\frac{36}{41}\cdot 131\cdot 2.26\approx 295\quad \text{VAM/P cases per million full mRNA} - 1273\ \text{vaccinations},$$

where we have applied the VSD-based comparison of VAM/P rates, mRNA-1273 vs. BNT162b2, discussed above. The first summand on the left of the preceding equation represents a VAM/P incidence rate of ≈35 cases per million 1st doses of mRNA-1273 among males 18–25, while the second represents ≈260 cases per million 2nd doses. Using the FDA's VAM/P case-hospitalization rate of 86% [1], we find the overall case rate of 295 per million full vaccinations corresponds to a rate of approximately 254 VAM/P hospitalizations per million full vaccinations and 224 per million 2nd doses. Thus, the FDA's estimate of 110 VAM/P hospitalizations per million 2nd doses of mRNA-1273 (used in Scenarios 1–4) is

---

[29] Lower estimate $(1-2.68/5.2)\times 100\%\approx 48.5\%$; upper $(1-2.68/7.2)\times 100\%\approx 62.8\%$

[30] Lower estimate $(1-6.36/19.4)\times 100\%\approx 67.2\%$; upper $(1-6.36/29.7)\times 100\%\approx 78.6\%$;

less than half the estimate of 224 per million 2nd doses derived from the FDA's BNT162b2 estimate and VSD data.

S6.2 Approximation of Second-Dose Rate Using Data from Patone et al. as well as Buchan et al. Patone et al. [58] analyze the incidence of myocarditis cases requiring hospitalization occurring within 28-days of a COVID-vaccine dose through a study population of over 42 million residents of England with a study period from 12/1/20 to 11/15/21. During the study period mRNA-1273 was approved for use in the United Kingdom for people 18 and older [59], so that among of the 813,524 2nd doses of mRNA-1273 administered to those 13–39 in the study population only 248 (0.03%) were administered to those 13–17 [58, Supplementary Table 1]. Thus, we assume that Patone et al.'s mRNA-1273 findings for males under 40 are for males 18–39. Patone et al. found among males 18–39 a rate of 101 (95%CI: 95, 104) excess myocarditis hospitalizations per million 2nd doses of mRNA-1273 (and approximately 12 (95%CI: 1, 13) excess myocarditis hospitalizations per 1 million 1st doses) [58, p. 4].[31] We expect a much higher rate of such hospitalizations among 18–25-year-old males than among males 18–39. To arrive at a plausible approximation of the rate among 18–25-year-old males, we rely on data from Public Health Ontario reported by Buchan et al. [61].

Supplementary Table 3 of [61] provides the following crude reporting rates of *myocarditis* per million second doses of mRNA-1273 administered: 299.5 (95%CI: 171.2, 486.4) for males 18–24 and 72.1 (95%CI: 31.1, 142.0) for males 25–39, with all cases of myocarditis contributing to the analysis meeting the requirements of level 1 or 2 of the Brighton Collaboration's myocarditis-case definitions. Note the ratio of these rates, the former divided by the latter is approximately 4.15.[32] Thus, if r denotes the myocarditis hospitalization rate among 25–39-year-old males for the 2nd dose of mRNA-1273, we take $4.15\ r$ to be the corresponding rate among 18–24-year-old males.

In Supplementary Table 1 of [58] we find the following:[33]

Numbers of 2nd doses of mRNA-1273 administered to members of Patone et al.'s study population

Women	Men	Sex not Recorded	18–29 year-olds	30–39 year-olds
405,748 (39.0%)	434.478 (41.8%)	199, 693 (19.2%)	431,451	381,825

Because the age range 18–24 constitutes 7 of the 12 years of the range 18–29, a natural approximation of the number of 2nd doses received by 18–24 year-olds is $251{,}680 \approx 7/12 \cdot 431{,}451$ based on the preceding table. If we assume that 41.8% of these doses are received by males, then we would have 105,202 2nd doses received by 18–24-year-old males out of a total of 339,949 ($\approx 0.418 \cdot (431{,}451 + 381{,}825)$) received by males 18–39. Thus, approximately 30.9% of the doses received by males 18–39 were received by those in the 18–24 range with about 69.1% received by males 25–39.

Recalling that r is the myocarditis hospitalization rate among 25–39-year-old males for the 2nd dose of mRNA-1273, and $4.15\ r$ is the corresponding rate among 18–24-year-old males, and the excess myocarditis hospitalization incidence is 101 per million 2nd doses among males 18–39, we assume

$$r \cdot 0.691 \cdot (1\ \text{millon}) + 4.15r \cdot 0.309 \cdot (1\ \text{million}) = 101.$$

Solving the preceding equation for r yields $r \approx 0.0000512$, so that $212 \approx 4.15r \cdot 1{,}000{,}000$ is a natural estimate for the number of excess myocarditis hospitalizations per million 2nd doses of mRNA-1273 among males 18–24.

If we assume that the VAM/P case-hospitalization rate for the 2nd dose of mRNA-1273 among 18–24 males is 86% (the rate FDA assumed in [1] for 18–25-year-old males), then we anticipate the 212 hospitalizations discussed in the preceding paragraph resulted from 247 excess myocarditis cases (after dose 2 of mRNA-1273). Supplementary Table 2 of [61] suggests that 83.33% of myo/pericarditis cases

---

[31] The published version [60] of the preprint [58] incorporates an additional month of data and suggests for males 18–39 a rate of 97 excess myocarditis hospitalizations per million second-doses of mRNA-1273 and a rate of 14 per million first doses [60, Table 4].

[32] The rate ratio of 4.15 is consistent with the following results reported by Oster et al. in [62, Table 2] based on passive-surveillance data from the CDC's VAERS system: a myocarditis incidence rate among males 18–24 of 56.31 per million 2nd doses of mRNA-1273 (7-day risk interval postvaccination) and a weighted average of corresponding rates for males 25–29 and 30–39 of 13.35 ($\approx 1/3 \cdot 24.18 + 2/3 \cdot 7.93$), yielding the rate ratio 4.22 ≈ 56.31/13.35.

[33] Husby et al. [63] provide no data for sub-ranges of the age range 12–39 precluding an analysis like the one completed in this subsection based on Patone et al.'s data.

contributing to Buchan et al.'s study are either for myocarditis or for myocarditis with pericarditis. It follows that we would anticipate the 247 myocarditis cases per million 2nd doses of mRNA-1273 corresponds to a VAM/P case rate of $296 \approx 247/0.8333$ per million 2nd doses of mRNA-1273, with $296 - 247 = 49$ of the VAM/P cases being for pericarditis alone. Supplementary Table 2 of [61] provides an estimate (age independent) that 38.8% of vaccine attributable pericarditis cases result in hospitalizations. Using this estimate, we anticipate that of the 49 pericarditis cases contributing to the total of 296 VAM/P cases discussed above $19 \approx 0.388 \cdot 49$ are hospitalized.[34] Adding these projected pericarditis hospitalizations to the 212 myocarditis hospitalizations discussed earlier, we obtain a rate of 231 VAM/P hospitalizations per million 2nd doses of mRNA-1273 among males 18–24, which is the VAM/P hospitalization rate reported in Column 4 of Table 2 of the main exposition, with the corresponding reported VAM/P case rate being 269 per million 2nd doses assuming an 86% VAM/P case-hospitalization rate.

Recall that in Table 2 of the main exposition, we combine VAM/P case and hospitalization-rate estimates from four different sources to obtain summary estimates, with column 4 of Table 2 presenting data from the source Patone et al. (with adjustments based on [61]) . The summary estimates are weighted averages with weights determined by the number of VAM/P events yielding each of the four contributing estimates. Patone et al.'s findings for males 18–39 are based on 36 hospitalized myocarditis cases ≤ 28 days from mRNA-1273 dose 2 (and 8 cases ≤ 28 days from mRNA-1273 dose 1). Seeking to apportion the 36 postdose 2 cases among 18–24-year-old males and 25–39-year-old males, use the rate ratio 4.15 as above:

$$234{,}747 \cdot r + 105{,}202 \cdot 4.15 \cdot r = 36,$$

where 234,747 is our estimate for the number of males 25–39 receiving mRNA-1273 dose 2 and 105,202 is the corresponding estimate for males 18–24; solving the equation for r yields $r \approx 0.00005362$, suggesting $105{,}202 \cdot 4.15 \cdot 0.00005362 \approx 23$ cases occurred among males 18–24 and the remaining 13 cases among males 25–39. Thus, we choose 23 as the number-of-events estimate appearing in column 4 of Table 2 of the main exposition.

S6.3 A Background Rate for Myo/pericarditis Among Young Males. Expected rates of myo/pericarditis displayed in Table 4 of [61] suggest an expected rate (or "background rate") of myo/pericarditis for 18–24-year-old males between 3.34 and 3.89 cases per million per week—see the next subsection for further discussion. This expected rate is consistent with data, described below, from the U.S.

Before describing this U.S. data, recall we use myo/pericarditis as shorthand for myocarditis/pericarditis, i.e., myocarditis or pericarditis or both (and many would use "myopericarditis" for "both," i.e., for myocarditis with pericarditis). However, the CDC sources [65] and [66] (both of which are slides of ACIP meeting presentations in, respectively, August 2021 and October 2021) use myopericarditis for myocarditis or myocarditis with pericarditis ([65, p. 11], [66, p. 11]).[35] The table on page 13 of [65] provides an incidence rate (passive surveillance through VAERS) of 37.7 myopericarditis cases per million 2nd doses of mRNA-1273 among 18–24-year-old males (7 day-risk period). The table on page 9 of [65] indicates that this incidence rate results from 79 myopericarditis cases; thus, the approximate number of second doses from which the 79 cases were identified is 79/37.7 ≈2.096 million. The table on page 9 of [65] indicates a background rate of 0 to 4 cases per week arising among such a group of 2.096 million males, 18–24 years old—hence, a background rate of up to 4/2.096 ≈1.91 cases per million per week. This background rate is consistent with the range "0.2 to 1.9 per 1 million person 7-day risk period" reported in a footnote on page 8 of [66].[36]

To get a background rate for myo/pericarditis based on U.S. data we need to add to the incidence rate of 1.91 myocarditis/myocarditis-with-pericarditis cases per million per week among 18–24 year-old males, an appropriate incidence rate for pericarditis. We depend upon a study "Myopericarditis and Pericarditis

---

[34] In a later study, Le Vu et al. [64] estimate approximately 33 pericarditis hospitalizations per million 2nd doses of mRNA-1273 among males 18–24, where a "pericarditis hospitalization" is one whose primary discharge diagnosis is pericarditis.

[35] Consistent with this definition, myopericarditis is defined as "acute myocarditis with or without pericarditis" in [67].

[36] Page 8 of [66] provides reporting rates of "myocarditis"; however, these rates are very similar to the "myopericarditis" rates reported on page 13 of [65]. Thus, we assume that "myocarditis" means myocarditis with or without pericarditis (a natural assumption).

in the Deployed Military Member: A Retrospective Series" [68] of cardiology patients from the U.S. Military receiving care at the cardiology clinic of the U.S. Military Hospital in Kuwait between 2004 and 2008. This Kuwaiti clinic was the primary evacuation node for U.S. Military personnel stationed in the Middle East during this period, and we assume most military service members deployed to the Middle East during this period were relatively young males. We note that this study uses the expected definitions: "Pericarditis is an inflammation of the fibrous pericardial sac surrounding the heart. Myopericarditis occurs when there is additional involvement of the myocardium." The incidence of hospitalized pericarditis cases found by the study is 7.4 per 100,000 person-years, or 7.4 cases over 5.2 million person-weeks, or ≈1.42 pericarditis cases per million per week. Thus, our background-rate estimate for myo/pericarditis is 1.91 + 1.42 = 3.33 cases per million per week, but we will assume this is the background rate of hospitalized cases. Assuming an 86% hospitalization rate for myo/pericarditis among males 18–24, a background rate of 3.33 hospitalized cases per million per week corresponds to a background rate of 3.33/0.86 ≈3.87 cases per million per week, consistent with Buchan et al.'s rate. *We apply a background rate myo/pericarditis rate of 4 cases per million per week among males 18–24 to determine VAM/P rates based on incidence rates.*

S6.4 Approximation of Second-Dose Rate Using Data from Sharff et al. As noted in the main exposition, Sharff et al. [69] found a myo/pericarditis incidence rate of 537.1 (95% CI: 215, 1100) per million second doses of an mRNA-vaccine for males ages 18 to 24 years. All cases presented in a 21-day interval post-vaccine administration with 6 cases following BNT162b2 and 1 following mRNA-1273 and with 6 of the 7 cases hospitalized (including the case following mRNA-1273). Given the strong evidence presented in the main exposition that dose 2 of mRNA-1273 carries significantly greater myo/pericarditis risk than dose 2 of BNT162b2, it's reasonable to use the incidence rate of 537.1 per million as an approximation of incidence rate for dose 2 of mRNA-1273 (and to assume the rate might be an underestimate).

Subtracting the expected rate (derived in Section S6.3) of 4 per million per week for the 3-week observation period for Sharff et al.'s study, we arrive at a VAM/P-rate estimate of 525 cases per million 2nd doses of mRNA-1273. Observe that the hospitalization rate for the 7 myo/pericarditis cases among males 18–24 in Sharff et al.'s study is $86\% \approx 6/7 \times 100\%$, in agreement with the FDA's rate for 18–25-year-old males used in [1]. Applying this 86% rate, we obtain a VAM/P-hospitalization rate estimate of 452 per million 2nd doses of mRNA-1273. The case and hospitalization rates derived above appear in column 5 of Table 2 of the main exposition, along with 7 events. Recall Sharff et al. offer an explanation of why their VAM/P rates are significantly higher than those "reported to US advisory committees":

> We identified additional valid cases of myopericarditis following an mRNA vaccination that would be missed by the VSD's search algorithm, which depends on select hospital discharge diagnosis codes. The true incidence of myopericarditis is markedly higher than the incidence reported to US advisory committees in the fall of 2021. The VSD should validate its search algorithm to improve its sensitivity for myopericarditis. [Here, myopericarditis is myocarditis or pericarditis (myo/pericarditis).]

S6.5 Approximation of Second-Dose Rate Using Data from Buchan et al. As noted in the main exposition, data from Table 4 of [61] suggests for males 18–24 a VAM/P case rate of 302 per million second doses of mRNA-1273 (7-day risk interval).[37] Here is the source data:

Table 4. Observed vs. expected episodes of myocarditis/pericarditis using a 7-day risk window following dose 2 of COVID-19 mRNA vaccines among individuals receiving dose 2 on or after June 1, 2021, by age group, sex, and vaccine product

Age group (years)	Females			Males		
	Individuals with 2 doses	Expected*	Observed	Individuals with 2 doses	Expected*	Observed
			mRNA-1273 – Dose 2			
18-24	170,317	0.2-0.2	**7**	179,866	0.6-0.7	**55**

Remarks: The myo/pericarditis cases tallied in the preceding table were detected during an "enhanced surveillance" period 6/1/21–9/4/21 [61, p. 3], and all cases contributing to Buchan et al.'s study were judged to meet the Brighton

[37] The data for dose 2 from Table 2 of the main exposition is independent of the type of dose 1. If we restrict to homologous dosing (1st dose also mRNA-1273), then the rate rises to at least 320 per million when the 2nd dose is taken less than 8 weeks after the first (with 4--6 weeks being the recommended interval). For further discussion of differences in VAM/P rates based the gap between doses 1 and 2 and on homologous vs. heterologous primary-series schedules, see Appendix S2 of this supplement, where we derive the 320 cases per million 2nd doses estimate for homologous dosing.

Collaboration's case definitions (levels 1–3). In fact, applying the Collaboration's requirements reduced the reported 417 post-mRNA-vaccination study-period cases of myo/pericarditis to 297 [61, p. 5].

Note that for males the ratio of observed events to vaccine doses yields an incidence rate of $55/0.179866 \approx 305.78$ cases per million 2nd doses of mRNA-1273. With an expected rate of between $0.6/0.179866 \approx 3.34$ cases per million and $0.7/0.179866 \approx 3.89$ cases per million (over 1 week), we obtain a VAM/P rate between $305.78 - 3.89 = 301.89$ and $305.78 - 3.34 = 302.44$, so that we take 302 per million to be the VAM/P case rate for the 2nd dose of mRNA-1273 (for 18–24-year-old male Ontarians).

For its BNT162b2 assessment, the FDA assumed all VAM/P cases were hospitalized [9, Section 2.3.2.2]. For its mRNA-1273 assessment, the FDA assumed VAM/P case-hospitalization rates decrease with age, starting with 86% for males 18–25 and ending with 77% for males 36 and over [1, Section 2.3.2.2]. Buchan et al. provide an overall case-hospitalization rate associated with the 2nd dose of an mRNA vaccine (all ages, males & females) of 79.2% ($\approx 164/207 \times 100\%$, with 10 of the 164 hospital admissions including ICU care) ([61, Table 1]. We expect the rate to be higher for the age range 18–24, but, conservatively, choose a hospitalization rate of 80%, obtaining an estimated VAM/P hospitalization rate of 242 ($\approx 0.80 \cdot 302$) per million 2nd doses of mRNA-1273 among males 18–24.

The case and hospitalization rates derived in the preceding two paragraphs appear in column 3 of Table 2 of the main exposition, along with 55 events.

We note that for a primary-series comprising two doses of mRNA-1273 among males 18–24, Table 3 of [61] provides, with wide confidence intervals, a reporting rate of 376.5 myo/pericarditis cases per million second doses when the second dose is administered ≤ 30 days after the first dose (as recommended in the U.S.[38]) and a reporting rate of 331.4 per million second doses when the second dose is administered 31 to 55 days after the first. Table 2 of [61] provides a reporting rate of 37.2 myo/pericarditis cases per million first doses of mRNA-1273.

S6.6 Computation of Summary Rates as a Weighted Average. The table below summarizes the VAM/P rate estimates for mRNA-1237 dose 2 among males 18–24/18–25 obtained in Sections S6.1, S6.2, S6.4, and S6.5 above:

Description	Buchan et al. [59]	Patone et al. [56, 59]	Sharff et al. [67]	Data From FDA & VSD [25, 54, 55]	Weighted Average of Highlighted Data
Cases per millon	302	269	525	260	301
Hospitalizations per million	242	231	452	224	250
Number of events on which estimate is based	55	23	7	22	Total Events 107

* See Sections 6.1, 6.2, 6.4, 6.5 above.

The weighted average determining the summary case-rate estimate in the final column is

$$302 \cdot \frac{55}{107}. + 269 \cdot \frac{23}{107} + 525 \cdot \frac{7}{107} + 260 \cdot \frac{22}{107} \approx 301,$$

and the weighted average determining the summary hospitalization-rate estimate is

$$242 \cdot \frac{55}{107}. + 231 \cdot \frac{23}{107} + 452 \cdot \frac{7}{107} + 224 \cdot \frac{22}{107} \approx 250.$$

S6.7 Approximation of First-Dose Rate. In its BLA-Memo Scenario [72], the FDA estimated VAM/P-hospitalization risk for mRNA-1273 at 128 per million *full vaccinations*, yielding an implied rate of 18 (= 128 – 110) hospitalizations per million 1st doses of mRNA-1273 (where 110 is the 2nd dose rate of the FDA's Scenarios 1-4 of [1]).

As discussed in Subsection S6.1 above, the FDA's assumption of BNT162b2 VAM/P risk from [9, Table 3] and the VSD risk-ratio data from [56, pp. 25, 27] suggest a VAM/P incidence-rate estimate of 35

---

[38] The initial CDC recommendations 12/20/2020 for COVID vaccination included , "The second dose should be administered as close to the recommended interval [4 weeks for mRNA-1273] as possible" [70]. However, on 1/21/2021, the CDC introduced more dosing-interval flexibility as follows: "The second dose should be administered as close to the recommended interval as possible. However, if it is not feasible to adhere to the recommended interval, the second dose of Pfizer-BioNTech and Moderna COVID-19 vaccines may be scheduled for administration up to 6 weeks (42 days) after the first dose" [71].

cases per million 1st doses of mRNA-1273 among males 18–25.[39] Using the FDA's assumed VAM/P hospitalization rate of 86%, we arrive at an estimate of 30 ($\approx 0.86 \cdot 35$) VAM/P hospitalizations per million 1st doses of mRNA-1273.

Recall from the main exposition as well as from Section S6.2 above that Patone et al. found an excess myocarditis hospitalization risk of 12 per million 1st doses of mRNA-1273 among males < 40 [58, p. 4]. We expect a higher rate among males 18–25. Based on Patone's findings it seems reasonable to take 12 per million 1st doses of mRNA-1273 as an estimate (highly likely an underestimate) of 1st dose VAM/P hospitalization risk.

Of the three estimates of VAM/P rates per million 1st doses of mRNA-1273 discussed above, 12, 18, and 30, we use the middle estimate 18.[40]

S6.8 Exclusion of VAM/P Rates Based on Strictly Passive Surveillance. We have relied heavily on VAM/P data collected by Public Health Ontario during a period of "enhanced passive surveillance" 6/1/21–9/4/21 [61, p. 3]. We have not included in our VAM/P-rate analysis sources depending on strictly passive surveillance; e.g., data from a study by Oster et al. [62] whose postvaccination myocarditis incidence rates are based on the U.S. CDC's passive adverse event reporting system VAERS.[41] Typically, incidence rates based on passive reporting are substantially below actual incidence rates. Here are some additional reasons why Oster et al.'s rates are substantially below actual VAM/P rates:

1. *MedDRA Code Limitation*: Oster et al. relied solely on MedDRA codes to pick up myocarditis cases in VAERS. These MedDRA codes miss cases that do not have a full diagnosis yet. In other words, in Oster et al., the authors found that 89% of all cases had chest pain and 98% of all confirmed myocarditis cases had elevated troponin documented in the notes. If the authors had searched for chest pain + troponin (two key components of the CDC's probable case requirements) and then reviewed all the clinical information they would have increased their detection substantially.
2. *Exclusion of Cases*: Oster et al. omitted ≈15% of VAERS cases lacking dose information, and ≈80% are likely dose 2-related [74].
3. *Omission of Pericarditis*: Oster et al. excluded pericarditis, which may account for up to ≈50% of VAM/P cases according to [75].

S7. Prior-infection protection against Omicron

We present evidence that during the evaluation period 1/1/22 – 5/31/22 prior infection provided greater protection against Omicron infection and hospitalization than did two-dose vaccination (among the COVID-naïve). In this section, we rely on results from some studies appearing after 1/21/22 and do not restrict discussions of such studies to footnotes.

As noted in the main exposition, CDC data [33] from the period May – November 2021 suggests that during the Delta-dominant period, prior infection provided greater protection against infection and hospitalization than did two-dose vaccination. Moreover, we provided immunological reasons to expect prior infection's greater protection to continue into the Omicron era and included, in a footnote, data from one reference supporting this assertion [76], which is a reference included in two meta-analyses of prior-infection protection [38] and [39] whose findings are reviewed below.

The meta-analysis [39], based on data published up to 31 September 2022, found "Although protection from re-infection from all variants wanes over time, our analysis of the available data [for all variants] suggests that the level of protection afforded by previous infection is at least as high, if not higher than that provided by two-dose vaccination using high-quality mRNA vaccines (Moderna and Pfizer-BioNTech)." The preceding finding of [39] is consistent with that of a pre-Omicron meta-analysis [35] based on studies published December 2020 through August 2021: "[O]ur review demonstrates that natural

---

[39] In an article by Wong et al. [73], the FDA published its VAM/P data (with later data cutoff points from its data partners than in [1]). The meta-analysis of Table 2 of [73] suggests a 1st dose VAM/P case rate among males 18–25 of 35 cases per million 1st doses of mRNA-1273 in agreement with our estimate obtained in Section 6.1. See Appendix S2 for details.

[40] In Appendix S2 of this supplement, we pool results from three sources appearing after 1/22/22 to obtain an estimate of 24 VAM\P hospitalizations per million 1st doses of mRNA-1273 administered to males 18–25.

[41] We note that it is not clear if [62] was available in some form before 1/22/22; [62] was published online 1/25/22.

immunity in COVID-recovered individuals is, at least, equivalent to the protection afforded by complete vaccination of COVID-naïve populations."

In the meta-analysis [39], the following three plots (B, D, & F of Figure 3) illustrate prior-infection's protection against Omicron, where "severe disease" is that resulting in hospitalization and/or death:

Figure S1.1: Plots from the meta-analysis [39] with highlighted protection levels from Table S2 of [3] added

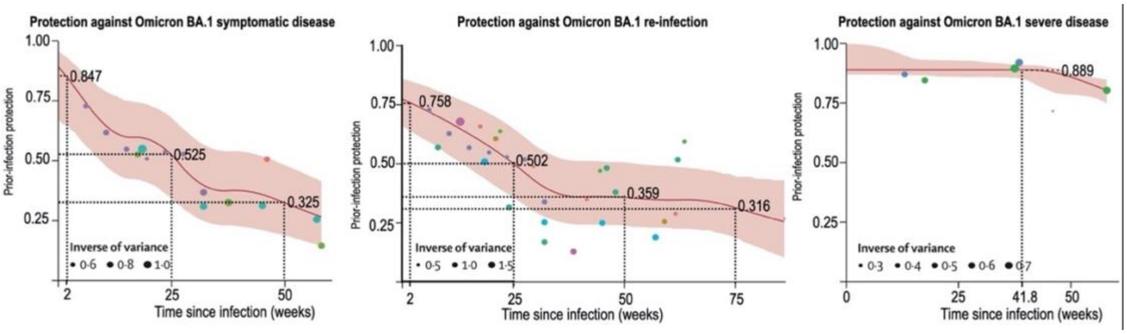

Similar findings are reported in the meta-analysis [38] based on studies from the period 1 January 2020 to 1 June 2022. Here are plots from Figure 2A of [38], where "any infection" encompasses both symptomatic and asymptomatic cases and "severe disease" is "a combination of the WHO definitions of severe, critical, or fatal COVID-19":

Figure S1.2: Plots from [38] displaying protection against Omicron variant conferred by previous infection, with highlighted protection levels from Table 2 of [38] added

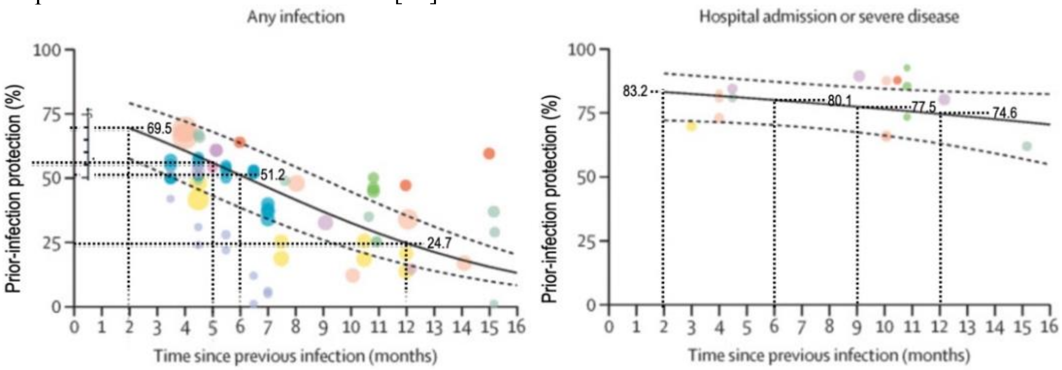

Let's turn our attention to the protection from Omicron that two doses of mRNA-1273 or two-doses of BNT152b2 provide. The study [52] (discussed in Section S5) was based on data collected in England during the period 11/27/21 – 1/12/22 and includes a total of 886,774 eligible persons infected with the Omicron variant. Below are plots, adapted from Figure 1 of [52], illustrating two-dose vaccine effectiveness against Omicron.

Figure S1.3: Plots adapted from [52] *displaying protection against symptomatic Omicron infection via labeled disks* (squares apply to the Delta variant). Data from Table 3 and Supplementary Table 2 of [52] have been added.

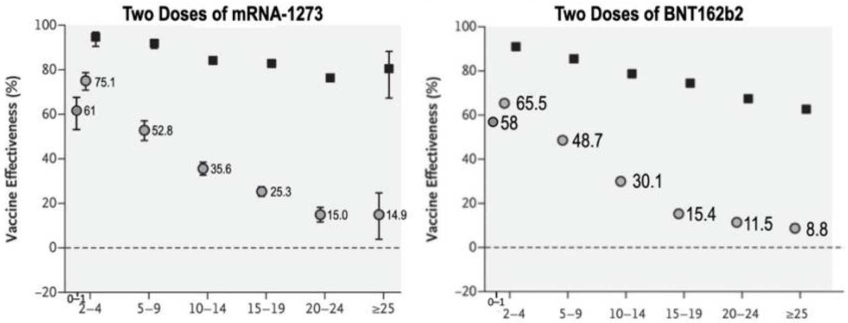

Findings from the study [52] contribute to a meta-analysis [77] of studies estimating COVID-vaccine effectiveness against Omicron infection, with included studies described in papers published from 26 November 2021 to 27 June 2022. The meta-analysis [77] characterizes the effectiveness data displayed in Figure S1.3 above as follows:

- two-doses of mRNA-1273 provide short-term protection against symptomatic infection (all ages) at an effectiveness level of 42.3% while the corresponding protection provided by two doses of BNT162b2 is 41.0% [77, Supplementary Figure 1], and
- two-doses of mRNA-1273 provide long-term protection against symptomatic infection (all ages) at an effectiveness level of 15.0% while the corresponding protection provided by two doses of BNT162b2 is 12.6% [77, Supplementary Figure 2].

In the meta-analysis [77], the duration of the "short-term" and "long-term" protection-periods varies somewhat, with the short-term period for the 13 contributing studies ending between 69 and 149 days postdose 2 (median: 104 days) and the long-term period for the 11 contributing studies beginning between 70 and 181 days postdose 2 (median: 120 days) [77, Supplementary Figures 1 & 2]. For those studies included in the long-term analysis that specified a maximum time since dose 2, the maximum was less than 15 months (and for any study whose study period ended on or before 3/1/22, this 15 month maximum would be valid). For all but 2 studies contributing to the short- and long-term effectiveness analysis, a homologous two-dose series of BNT162b2 or mRNA-1273 vaccine was considered while for remaining 2 studies a heterologous series was considered consisting of BNT162b2 and mRNA-1273. For all but 2 studies contributing to the short- and long-term effectiveness analysis, protection against *symptomatic* Omicron was assessed (with the remaining two studies assessing protection against all infection).

For the pooled analyses of [77], Supplementary Figures 1 and 2, respectively, indicate that the short-term effectiveness against Omicron infection (symptomatic or all) of two-dose vaccination to be 43.5% (all ages) and long-term to be 16.4% (all ages). Observe that the pooled prior-infection effectiveness estimates of the meta-analyses [39] and [38] illustrated, respectively, in Figure S1.1 & S1.2 are clearly above the vaccine-effectiveness estimates of [77], for both the short and long terms. For instance, for [38] the average effectiveness of prior-infection protection over the 149-day period after infection is about 63% while for [39] it's above 67% for symptomatic infection and above 66% for all infection.[42] Compare the short-term vaccine-effectiveness estimate of 43.5% from [77] to these short-term protection estimates of 63%, 66%, and 67% for prior infection, and note the latter estimates would be even higher had we made a more neutral choice of endpoint for prior-infection's short-term interval, e.g., having the interval end at 104 days rather than 149 days post infection.

Turning to the long-term: for prior-infection protection corresponding to the plot on the left of Figure S1.2 above, an underestimate of the average protection level beyond 181 days after infection (through 15 months after) is obtained by averaging the three protection estimates for months 9, 12, and 15 from Table 2 of [38]: $(37.0 + 24.7 + 15.5)/3 \approx 25.7$. We dramatically underestimate long-term protection corresponding to the leftmost plot Figure S1.1 by using the prior-infection effectiveness estimate 26.8% provided in Table S2 of [39] at week 58, which is the last data point in Table S2 for the plot of Figure S1.1. Similarly, for the middle plot of Figure S1.1, estimated protection is 25.6% 86 weeks (nearly 20 months) after infection. Observe that our underestimates of long-term prior-infection protection 25.7%, 25.6%, and 26.8% are considerably above the pooled long-term vaccine protection estimate 16.4% from [77, Supplementary Figure 2].

As for vaccination protection from severe Omicron disease, a systematic review [78] involving 21 vaccine effectiveness studies from the period 3 December 2021 – 7 April 2022 reports,

> In contrast to vaccine effectiveness against delta severe disease, the majority of vaccine effectiveness estimates for omicron severe disease were below 75%; for example, thirteen (81%) of sixteen vaccine

[42] We compute the average effectiveness corresponding to Figure S1.2 of [38] for the 149-day period immediately following infection as the average value of the effectiveness function over the interval from 2 months (about 61 days after infection) to 149 days past infection. Because the effectiveness curve is close to being a straight line over this interval, we can simply average the effectiveness estimates at 2 months (69.5%) and at 149 days ($\approx$ 56%) to approximate average effectiveness up to 149 days after infection $(69.5 + 56)/2 \approx 63$. We underestimate the average effectiveness corresponding to the leftmost plot Figure S1.1 of [39] for the 149-day period immediately following infection as the average ($\approx$ 67.4) of the effective estimates for weeks 1 through 22 from Table S2 of [39] and the corresponding estimate for the middle plot is 66.2.

> effectiveness estimates within three months of vaccination with the primary series were below 75% (Figure). Moreover, 13 (42%) vaccine-specific estimates fell below 50% at some point in time after vaccination.

The authors of [78] also report,

> There is a suggestion that the vaccine effectiveness after the primary series is lower when severe disease is defined as hospitalization without requirement for clinical criteria of Covid-19 than hospitalization with clinical criteria, particularly after 3 months since vaccination, although too few studies (n = 3) are available to make a definitive comparison.

For example, a study [79] based on data from England found 2-dose vaccination for 18–64 year-olds to be less than 40% effective against Omicron hospitalization 25+ weeks after the second dose of BNT162b2[43] [79, Figure 2b] while the corresponding effectiveness level rises to 65.1% if hospitalization is primary respiratory coded and lasts 2 or more days [79, Table S13]. Note that for the study [79], "The data was restricted to tests up to 23 February 2022 to account for delays in the SUS data recording" [79, Supplement, p. 2]. Because the vaccination program began in England 8 December 2020, 25+ weeks for the study [79] means 25 to 63 weeks (about 14.5 months). Thus, according to [79. Table S13], with the definition of COVID hospitalization meaning "primary respiratory coded and lasts 2 or more days," the effectiveness of 2 doses of BNT162b2 vaccination against hospitalization among 18–64 year-olds is 65.1% during the 25–63 week interval and 73.8% from 2 to 25 weeks.

Comparing effectiveness estimates of the preceding paragraph to corresponding estimates illustrated in Figures S1.1 and S1.2,[44] we appear to have clear and convincing evidence that prior-infection provides significantly more protection against severe Omicron than does 2-dose vaccination. We have already pointed out how prior-infection protection against Omicron infection appears to exceed substantially that conferred by 2-dose vaccination both in the short and long terms. However, there is an important issue to consider. Protection from prior infection is measured with respect to the infection-naïve unvaccinated and vaccine-associated protection against Omicron is typically measured in studies that include those with prior infection in both the vaccinated and unvaccinated groups. What happens to the vaccine-effectiveness estimates discussed above relative to Omicron if we remove those having prior COVID infection from both the vaccinated and unvaccinated groups? We provide below strong evidence that the removal of those with prior infection does not increase vaccine-effectiveness estimates—in fact, the evidence suggests the removal likely decreases vaccine-effectiveness estimates. Thus, the comparisons provided at the beginning of this paragraph are apt—during the evaluation period 1/1/22 – 5/31/22 prior-infection provided more protection against Omicron than did two-dose mRNA vaccination alone (non-hybrid protection).

First, we consider a study [80] of Omicron disease during the period 1 February 2022 through 31 May 2022 *in the essentially COVID-infection naïve population of Western Australia*. In Australia, "Soon into the rollout, the Pfizer vaccine became the 'preferred' vaccine for people aged <60 years in non-outbreak settings due to an increased risk of thrombosis with thrombocytopenia syndrome following AstraZeneca vaccination in younger cohorts" [80]. According to an Australian-government report [81], as of 8 January 2023: over 44 million Pfizer doses had been administered, over 5 million Moderna doses, and over 237,600 Novavax. The report adds, "The Vaxzevria (AstraZeneca) vaccine is provisionally approved for adults. To 8 January 2023, just under 14 million doses of Vaxzevria (AstraZeneca) have been administered in Australia. Very few doses of this vaccine have been used since 2021."

The study [80] has a restrictive definition of COVID hospitalization:

> We defined a SARS-CoV-2 hospitalization as >1 inpatient admissions 0–7 days after the date of a positive PCR result. To reduce potential bias that might be introduced through routine preadmission SARS-CoV-2 screening, we excluded admissions for patients indicated by the specialty of the admitting

---

[43] 2nd dose effectiveness for mRNA-1273 is not provided.

[44] Both indicating the effectiveness of prior-infection protection against hospitalization is over 80% for at least 26 weeks after infection, and from 25 to 69 weeks over 70% for Figure S1.2 and from 25 to 58 weeks over 80% for Figure S1.1.

clinician to be unlikely to have been hospitalized for treatment of COVID-19 and for patients admitted for boarding purposes (Table 1).

Nevertheless, the study found "Overall, VE against severe disease for 2 doses of vaccine was 41.9% (95% CI 4.8%–64.5%)," where "severe disease" is defined as "SARS-CoV-2 hospitalization, an associated death, or both." To compare the effectiveness estimate of 41.9% to those reviewed in [78], we choose estimates from [78] applying to the period "6+ months" postdose 2 with severe disease defined as "hospitalization with requirement of clinical criteria." The 11 corresponding severe-Omicron effectiveness estimates from [78] (see Table 1 and the associated figure) are 72%, 68%, 65.4%, 65.1%, 61%, 57%, 57%, 53.1%, 53%, 43%, and 38%. Thus, only one of the estimates falls below the vaccine effectiveness estimate of 41.9% (relative to the infection-naïve unvaccinated), and the one estimate 38% lying below 41.9% applies to a study population with ages between 12 and 18. Moreover, we expect the overall-effectiveness estimate of 41.9% of [80] would fall were it an estimate for protection 6+ months after vaccination. Finally, note that the meta-analysis [77] found "The overall VE of the full doses against Omicron-associated severe events was estimated to be 57.3% (95% CI: 48.5–64.7%, 24 studies) for all ages," and VE estimates of pure mRNA vaccines were 60.9% (95% CI: 50.7–68.9%, 18 studies) for all ages, Thus, the effectiveness-against-severe disease estimate 41.9% of [80] suggests that measuring the protection of vaccination against severe Omicron relative to the infection-naïve unvaccinated will not result in increased estimates of effectiveness, in fact the estimate of [80], compared to those of [77] and [78], suggests the opposite is more likely.

The study [80] also found "[I]n adjusted analyses comparing those who were unvaccinated with those who had received 2 vaccine doses, VE for preventing PCR-confirmed infection of any severity was 24.9% (95% CI 21.2%–28.4%)." The study's findings also suggest *that even three-dose vaccination* may not protect against Omicron as well as prior-infection when both vaccination effectiveness and prior-infection effectiveness are measured with respect to the infection-naïve unvaccinated. For instance, Figure 5 of [80] suggests the effectiveness of three homologous doses is below that of prior-infection effectiveness and falls below 60% by one month after boosting and below 10% by 4 months after boosting. Figure 5 of [80] also suggests that three heterologous doses provide effectiveness roughly comparable to that indicated by the leftmost two plots of Figure S1.1 for up to 4 months but falling below 50% by 4 months after boosting and below 20% by 5 months after boosting. Definitions of homologous and heterologous from [80] are as follows: a homologous 3-dose vaccination schedule consists of 2 doses of an mRNA vaccine and an mRNA booster regardless of brand/manufacturer, and a heterologous 3-dose vaccination schedule consists of 2 doses of ChAdOx1 followed by an mRNA booster dose, regardless of brand/manufacturer.

Like Western Australia, the population of Hong Kong was essentially COVID-naïve 1 January 2022 but experienced a significant wave of Omicron infection 1/1/22 – 7/31/22. Based on data collected 4/28/22 – 7/30/22, authors of the study [54] found that "Assuming VE took full effect 7 days after vaccination, we estimated: (1) VE for the second, third or fourth doses of BNT162b2 were 13% (95% credible interval: 2–39%**)**, 48% (34–64%) and 69% (46–98%) 7 days following immunization, respectively, waning to 7% (1–21%), 26% (7–41%) and 35% (10–71%) 100 days after immunization." Thus, the findings of [54] suggest that even 4 doses of BNT162b provided less-effective protection against Omicron infection relative to the COVID-naïve unvaccinated than does prior infection (alone).

Finally, consider the following excerpt from Figure 3 of [82], reporting some of the findings of a study of mRNA COVID vaccines' effectiveness against Omicron infection (based on data collected 11/1/2021 – 4/30/2022 in the Yale New Haven Health System):

Vaccine effectiveness among people without a prior infection*	Adjusted Vaccine effectiveness (95% CI)	Adjusted P-value	Unadjusted Vaccine effectiveness (95% CI)	Unadjusted P-value
Primary vaccination: <14 days after 2nd dose	21.5% (14.0, 28.3%)	<0.001	13.5% (6.1, 20.3%)	0.001
Primary vaccination: 14-149 days after 2nd dose	27.1% (18.7, 34.6%)	<0.001	-28.8% (-42.3, -16.6%)	<0.001
Primary vaccination: ≥150 days after 2nd dose (pre-booster)	13.6% (8.7, 18.2%)	<0.001	5.8% (1.5, 9.9%)	0.009

* Prior infection = documented prior infection

Note the adjusted vaccine-effectiveness estimates (e.g., 27.1%, 14–149 days after 2nd dose) are consistent with those of [54] and [80] reporting on studies conducted in essentially infection-naïve populations.

Moreover, restricting to those *having prior documented infections*, the study [82] found two-dose vaccine effectiveness to be higher (e.g., 41% 14–149 days after dose 2). Considering the data collection-period 11/1/2021 – 4/30/2022 for the study [82], we believe one possible reason why restricting to those with prior documented infection increases the apparent effectiveness of vaccination is that infections among the vaccinated might tend to be more recent than those among the unvaccinated (because vaccination was much more effective against pre-Delta variants than against Delta and more effective against Delta, than against Omicron).

Depletion-of-susceptibles bias: Consider a cohort study with one cohort being a large group of individuals vaccinated against disease D each matched with a member of a corresponding cohort of unvaccinated individuals who remain unvaccinated, with members of both cohorts being disease-D naïve. Suppose the cohort study commences 1 March 2021, the day each member of the vaccinated cohort is vaccinated against disease D. Defining the susceptibles in each cohort to be the disease-D naïve, we expect a "differential depletion of susceptibles" (cf. [83]): a more rapid decrease in the susceptible population of the unvaccinated cohort.

We measure vaccine effectiveness by comparing disease-incidence rate in the vaccinated cohort to that in the unvaccinated cohort. Suppose that the vaccine against disease D has high effectiveness that does not wane and that the same is true of the protection derived from a prior disease-D infection; then, owing to a more rapid of depletion of susceptibles over time in the unvaccinated cohort, we would expect the incidence rate in the unvaccinated cohort to decrease over time while that in the vaccinated cohort remains relatively stable (or slowly decreases if hybrid protection is greater than vaccine-only protection). Comparing the two incidence rates would thus yield, over time, an increasing ratio of the vaccinated-incidence rate to the unvaccinated-incidence rate falsely suggesting waning vaccine effectiveness.[45]

However, if vaccine protection against disease D does wane significantly (say, after 5 or 6 months), so that the effect of vaccination is principally to delay infection in the vaccinated cohort, then in a study of infections in the vaccinated and unvaccinated cohorts with study period beginning 9 or 10 months after 1 March 2021, we might find many of the vaccinated and unvaccinated have had a prior infection and would find that infections in the vaccinated population would tend be more recent than those in the unvaccinated population. If prior-infection protection also wanes, then those among the vaccinated with more recent prior infections would have higher levels of protection than the unvaccinated having earlier prior infections, which might lead to the study's overestimating vaccine effectiveness (relative to a study of vaccination effectiveness with members of the vaccinated and unvaccinated groups being disease-D naïve). *Comparing the COVID vaccine-effectiveness estimates from studies [54] and [80] computed within populations that were initially essentially COVID naïve to corresponding estimates from meta-analyses [77] and [78] for which contributing study populations were not initially COVID naïve suggests that many studies of COVID-vaccine effectiveness based on data from the early Omicron period exaggerated vaccine (and perhaps even booster) effectiveness because of vaccination's delaying but not preventing depletion of susceptibles in the vaccinated population.*

References

1. Yogurtcu, O.N., Funk, P.R., Forshee, R.A., Anderson, S.A., Marks, P.W., Yang, H. Benefit-risk assessment of COVID-19 vaccine mRNA (mRNA-1273) for males age 18--64 years. Vaccin*e* 2023; 14: 100325. doi: https://doi.org/10.1016/j.jvacx.2023.100325

2. Estimated COVID-19 Burden. Updated 8 November 2021. United States Centers for Disease Control and Prevention. Available online: https://web.archive.org/web/20211110062857/https://www.cdc.gov/coronavirus/2019-ncov/cases-updates/burden.html

[45] Under the assumption that vaccine protection does not wane, we expect that comparisons of incidence rates over time restricted to only the susceptibles in each cohort would not suggest waning.

3. Trends in Number of COVID-19 Cases and Deaths in the US Reported to CDC, by State/Territory, United States Centers for Disease Control and Prevention. Available online: https://web.archive.org/web/20211216002330/https://covid.cdc.gov/covid-data-tracker/#trends_dailycases

4. Nationwide COVID-19 Infection-Induced Antibody Seroprevalence (Commercial laboratories), United States Centers for Disease Control and Prevention. Available online https://covid.cdc.gov/covid-data-tracker/#national-lab

5. Trends in Demographic Characteristics of People Receiving COVID-19 Vaccinations in the United States. United States Centers for Disease Control and Prevention. Available online: https://covid.cdc.gov/covid-data-tracker/#vaccination-demographics-trends

6. Herrera-Esposito, D., de los Campos, G. Age-specific rate of severe and critical SARS-CoV-2 infections estimated with multi-country seroprevalence studies. Preprint. medRxiv 2021. 07.29.21261282. doi: https://doi.org/10.1101/2021.07.29.21261282

7. Herrera-Esposito, D., de los Campos, G. Age-specific rate of severe and critical SARS-CoV-2 infections estimated with multi-country seroprevalence studies. BMC Infect. Dis. 2022; 22:311-325. https://doi.org/10.1186/s12879-022-07262-0

8. Oprihory, J. Snapshot: DOD and COVID-19. Air & Space Forces Magazine. 2021: Jan. 4. Available online: https://www.airandspaceforces.com/snapshot-dod-and-covid-19/

9. Funk, P.R., Yogurtcu, O.N., Forshee, R.A., Anderson, S.A., Marks, P.W., Yang, H. Benefit-risk assessment of COVID-19 vaccine, mRNA (Comirnaty) for age 16–29 years. Vaccine 2022; 40: 2781–2789. doi: 10.1016/j.vaccine.2022.03.030

10. Kaim, A., Shetrit, S.B., Saban M. Women Are More Infected and Seek Care Faster but Are Less Severely Ill: Gender Gaps in COVID-19 Morbidity and Mortality during Two Years of a Pandemic in Israel. Healthcare (Basel) 2022; 10:2355. doi: 10.3390/healthcare10122355

11. Gombar, S., Chang, M., Hogan, C., Zehnder, J., Boyd, S., et al. Persistent detection of SARS-CoV-2 RNA in patients and healthcare workers with COVID-19. J. Clin. Vir. 2020; 129: 104477. doi: https://doi.org/10.1016/j.jcv.2020.104477

12. Transcript of Interview with CDC Director Rochelle Walensky]. Fox News Sunday. 9 January 2022. Available online: https://www.foxnews.com/transcript/fox-news-sunday-on-january-9-2022

13. Breakdown of COVID-19 positive hospital admissions. Government of Ontario. Available online: https://data.ontario.ca/dataset/breakdown-of-covid-19-positive-hospital-admissions.

14. McAlister, F.A., Hau, J.P., Atzema, C., McRae, A.D., Morrison, L.J., Grant, L., et al. The burden of incidental SARS-CoV-2 infections in hospitalized patients across pandemic waves in Canada. Sci Rep. 2023;24:6635. doi: 10.1038/s41598-023-33569-2

15. Thayer, J., Miller, A., Hirz, K., Poxner, X., Sandberg, N., Vander Berg, D., et al., A Bright Side: Hospitalizations for COVID-19 Might Be Overcounted, Especially Among Kids, Epic Research 2022. Available online: https://epicresearch.org/articles/a-bright-side-hospitalizations-for-covid-19-might-be-overcounted-especially-among-kids

16. Sorg, AL., Hufnagel, M., Doenhardt, M., et al. Risk of Hospitalization, severe disease, and mortality due to COVID-19 and PIMS-TS in children with SARS-CoV-2 infection in Germany. Preprint. medRxiv 2021.11.30.21267048; doi: https://doi.org/2021.11.30.21267048

17. Sorg, AL., Hufnagel, M., Doenhardt, M., et al. Risk for severe outcomes of COVID-19 and PIMS-TS in children with SARS-CoV-2 infection in Germany. Eur J Pediatr 2022; 181: 3635–3643. doi: https://doi.org/10.1007/s00431-022-04587-5

18. Ferguson, N., Ghani, A., Hinsley, W., Volz, E. Report 50: Hospitalisation risk for Omicron cases in England. Imperial College London (12/22/2021). Available online: https://www.imperial.ac.uk/media/imperial-college/medicine/mrc-gida/2021-12-22-COVID19-Report-50.pdf

19. COVID-19 Archive data download. UKHSA data dashboard, Cases. cumCasesBySpecimenDate_nation_2021.csv, cumReinfectionsBySpecimenDate_nation_2021.csv. Available online: https://ukhsa-dashboard.data.gov.uk/covid-19-archive-data-download

20. 2020 Demographics Profile of the Military Community. Department of Defense. United States of America. Available Online: https://download.militaryonesource.mil/12038/MOS/Reports/2020-demographics-report.pdf

21. UK Health Security Agency. Technical briefing: Update on hospitalisation and vaccine effectiveness for Omicron VOC-21NOV-01 (B.1.1.529). 2021; Dec 31. https://assets.publishing.service.gov.uk/government/uploads/system/uploads/attachment_data/file/1045619/Technical-Briefing-31-Dec-2021-Omicron_severity_update.pdfss

22. Wolter, N., Jassat, W., Walaza, S., Welch, R., Moultrie, H., et al. Early assessment of the clinical severity of the SARS-CoV-2 Omicron variant in South Africa. Preprint. medRxiv 2021.12.21.21268116; doi: https://doi.org/10.1101/2021.12.21.21268116

23. Wolter, N., Jassat, W., Walaza, S., Welch, R., Moultrie, H., et al. Early assessment of the clinical severity of the SARS-CoV-2 omicron variant in South Africa: a data linkage study. Lancet 2022; 399: 437–446. https://doi.org/10.1016/S0140-6736(22)00017-4

24. Abdullah, F., Myers, J., Basu, J., Tintinger, G., Ueckermann, V., et al. Decreased severity of disease during the first global omicron variant covid-19 outbreak in a large hospital in tshwane, south africa. Int. J. Infect. Dis. 2022 Mar;116:38-42. doi: doi: https://doi.org/10.1016/j.ijid.2021.12.357. Epub 2021 Dec 28.

25. Robinson, M.L., Morris, C.P., Betz, J.F., Zhang, Y., Bollinger, R., Wang, N., Thiemann, D.R., et al. Impact of Severe Acute Respiratory Syndrome Coronavirus 2 (SARS-CoV-2) Variants on Inpatient Clinical Outcome, Clin. Inf. Dis., 2022; ciac957, https://doi.org/10.1093/cid/ciac957

26. Paredes, M.I., Lunn, S.M., Famulare, M., Frisbie, L.A., Painter, I., Burstein, R., Roychoudhury, P., Xie, H., Mohamed Bakhash, S.A., Perez, R., et al. Associations Between Severe Acute Respiratory Syndrome Coronavirus 2 (SARS-CoV-2) Variants and Risk of Coronavirus Disease 2019 (COVID-19) Hospitalization Among Confirmed Cases in Washington State: A Retrospective Cohort Study. Clin. Infect. Dis. 2022; 75:e536–e544. doi: 10.1093/cid/ciac279

27. COVID-19 vaccination: Vaccination coverage. Number and percentage of people that have received a COVID-19 vaccine in Canada by province or territory, vaccination status, sex, age group, and last booster dose by vaccine. Government of Canada. Available online: https://web.archive.org/web/20211019014456/https://health-infobase.canada.ca/covid-19/vaccination-coverage/?wbdisable=true

28. COVID-19 Vaccination Demographics in the United States, National. United States Centers for Disease Control and Prevention. Available online: https://data.cdc.gov/Vaccinations/COVID-19-Vaccination-Demographics-in-the-United-St/km4m-vcsb

29. Total number of cases of COVID-19 in the United States as of December 30, 2021, by age group. Statista. Available online: https://web.archive.org/web/20211006060703/https://www.statista.com/statistics/1254271/us-total-number-of-covid-cases-by-age-group/

30. National Population by Characteristics: 2020-2022. Vintage 2022. Median Age and Age by Sex. Spreadsheet: Annual Estimates of the Resident Population by Single Year of Age and Sex for the United States: April 1, 2020 to July 1, 2022 (NC-EST2022-SYASEX). United States Census Bureau. Available online: https://www.census.gov/data/tables/time-series/demo/popest/2020s-national-detail.html#v2021

31. Mathioudakis, A., Ghrew, M., Ustianowski, A., Ahmad, S., Borrow, R., et al. Self-Reported Real-World Safety and Reactogenicity of COVID-19 Vaccines: A Vaccine Recipient Survey. Life 2021;11: 249. doi: https://doi.org/10.3390/life11030249

32. Johnson A.G., Amin A.B., Ali A.R., et al. COVID-19 Incidence and Death Rates Among Unvaccinated and Fully Vaccinated Adults with and Without Booster Doses During Periods of Delta and Omicron Variant Emergence — 25 U.S. Jurisdictions, April 4–December 25, 2021. MMWR Morb Mortal Wkly Rep 2022;71:132–138. DOI: http://dx.doi.org/10.15585/mmwr.mm7104e2

33. León, T.M., Dorabawila, V., Nelson, L., Lutterloh, E., Bauer, U., et al. COVID-19 Cases and Hospitalizations by COVID-19 Vaccination Status and Previous COVID-19 Diagnosis — California and New York, May–November 2021. MMWR Morb Mortal Wkly Rep 2022;71:125–131. DOI: http://dx.doi.org/10.15585/mmwr.mm7104e1

34. Chen, S., Flegg, J.A., White, L.J., Aguas, R. Levels of SARS-CoV-2 population exposure are considerably higher than suggested by seroprevalence surveys. PLoS Comput Biol. 2021; 17:e1009436. doi: https://doi.org/10.1371/journal.pcbi.1009436

35. Shenai, M.B., Rahme, R., Noorchashm, H. Equivalency of Protection From Natural Immunity in COVID-19 Recovered Versus Fully Vaccinated Persons: A Systematic Review and Pooled Analysis. Cureus. 2021;13: e19102. doi: 10.7759/cureus.19102.

36. Gazit, S., Shlezinger, R., Perez, G., Lotan, R., Peretz, A., et al. Comparing SARS-CoV-2 natural immunity to vaccine-induced immunity: reinfections versus breakthrough infections. Preprint. medRxiv 2021.08.24.21262415; doi: https://doi.org/10.1101/2021.08.24.21262415

37. Press Release. Discovery Health, South Africa's largest private health insurance administrator, releases at-scale, real-world analysis of Omicron outbreak based on 211 000 COVID-19 test results in South Africa, including collaboration with the South African Medical Research Council (SAMRC) on vaccine effectiveness. Available online: https://www.discovery.co.za/corporate/health-insights-omicron-outbreak-analysis

38. Bobrovitz, N., Ware, H., Ma, X., Li, Z., Hosseini, R., Cao, C., et al. Protective effectiveness of previous SARS-CoV-2 infection and hybrid immunity against the omicron variant and severe disease: a systematic review and meta-regression. Lancet Infect. Dis. 2023; 23:556--567 doi: https://doi.org/10.1016/S1473-3099(22)00801-5

39. COVID-19 Forecasting Team. Past SARS-CoV-2 infection protection against re-infection: a systematic review and meta-analysis. Lancet. 2023 Mar 11;401(10379):833-842. doi: https://doi.org/10.1016/S0140-6736(22)02465-5.

40. Cohen, K.W.; Linderman, S.L.; Moodie, Z.; Czartoski, J.; Lai , L.; et al. Longitudinal analysis shows durable and broad immune memory after SARS-CoV-2 infection with persisting antibody responses and memory B and T cells. Cell. Rep. Med. 2021;2:100354. doi: 10.1016/j.xcrm.2021.100354

41. Science Brief: SARS-CoV-2 Infection-induced and Vaccine-induced Immunity. Updated Oct. 29, 2021. Available online: https://stacks.cdc.gov/view/cdc/111167

42. Pulliam, J.R.C., van Schalkwyk, C., Govender, N., von Gottberg, A., Cohenm C., et al. Increased risk of SARS-CoV-2 reinfection associated with emergence of the Omicron variant in South Africa. Preprint. medRxiv 2021.11.11.21266068; doi: https://doi.org/10.1101/2021.11.11.21266068

43. Pulliam, J.R.C., van Schalkwyk, C., Govender, N., von Gottberg, A., Cohenm C., et al. Increased risk of SARS-CoV-2 reinfection associated with emergence of the Omicron variant in South Africa. Science. 2022; 376. doi: https://www.science.org/doi/abs/10.1126/science.abn4947

44. Total Coronavirus Cases in South Africa. Worldometer. Available online: https://www.worldometers.info/coronavirus/country/south-africa/.

45. Masresha, B., Poy, A., Weldegebriel, G., Mbuyita, S., Fussum, D., Bwaka, A., Paluku, G., Atuhebwe, P., Mihigo, R., Impouma, B. Progress with COVID-19 vaccination in the WHO African Region in 2021. Pan Afr. Med. J. 2022;41:8. doi: 10.11604/pamj.supp.2022.41.2.34102

46. Johns Hopkins Coronavirus Resource Center. United States. Data Timeline. Number of Daily Cases. Available online: https://coronavirus.jhu.edu/region/united-states

47. El Sahly, H.M., Baden, L.R., Essink, B., Doblecki-Lewis, S., Martin, J.M., et al. Efficacy of the mRNA-1273 SARS-CoV-2 Vaccine at Completion of Blinded Phase. N. Engl. J. Med 2021;385:1774–1785, doi: 10.1056/NEJMoa2113017.

48. Chin, E.T., Leidner, D., Zhang, Y., Long, E., Prince, L., Li, Y., Andrews, J.R., et al. Effectiveness of the mRNA-1273 vaccine during a SARS-CoV-2 delta outbreak in a prison. N. Engl. J. Med. 2021;385:2300–2301. doi: https://doi.org/10.1056/NEJMc2114089

49. UK Health Security Agency. COVID-19 vaccine surveillance report: Week 13. 2022; March 31. Available online: https://assets.publishing.service.gov.uk/government/uploads/system/uploads/attachment_data/file/1066759/Vaccine-surveillance-report-week-13.pdf

50. Andrews, N., Stowe, J., Kirsebom, F., Toffa, S., Rickeard, T., et al.Effectiveness of COVID-19 vaccines against the Omicron (B.1.1.529) variant of concern. Preprint. medRxiv. 14 December 2021 doi: 10.1101/2021.12.14.21267615.

51. Buchan, S.A., Chung, H., Brown, K.A., Austin, P.C., Fell, D.B., et al. Effectiveness of COVID-19 vaccines against Omicron or Delta infection. Preprint. medRxiv. 1 January 2022. https://www.medrxiv.org/content/10.1101/2021.12.30.21268565v1.

52. Andrews, N., Stowe, J., Kirsebom, F., Toffa, S., Rickeard, T., Gallagher, E., Gower, C., et al. Covid-19 Vaccine Effectiveness against the Omicron (B.1.1.529) Variant. N. Engl. J. Med. 2022;21:1532-1546. doi: https://doi.org/10.1056/NEJMoa2119451

53. Buchan, S.A., Chung, H., Brown, K.A., Austin, P.C., Fell, D.B., et al. Estimated Effectiveness of COVID-19 Vaccines Against Omicron or Delta Symptomatic Infection and Severe Outcomes. JAMA Netw. Open. 2022;5:e2232760. doi: https://doi.org/10.1001/jamanetworkopen.2022.32760

54. Lau, J.J., Cheng, S.M.S., Leung, K. et al. Real-world COVID-19 vaccine effectiveness against the Omicron BA.2 variant in a SARS-CoV-2 infection-naive population. Nat. Med. 2023;29:348–357. https://doi.org/10.1038/s41591-023-02219-5

55. Ferguson, N., Ghani, A., Cori, A., Hogain, A., Hinsley, W., Volz, E. Report 49: Growth, population distribution and immune escape of Omicron in England. Imperial College London (12/16/2021). Available online: https://www.imperial.ac.uk/media/imperial-college/medicine/mrc-gida/2021-12-16-COVID19-Report-49.pdf

56. Klein, N. Myocarditis analyses in the vaccine safety datalink: rapid cycle analyses and “Head-to-Head” product comparisons. CDC ACIP meeting on COVID-19 vaccines, October 2021. Available online https://www.cdc.gov/vaccines/acip/meetings/downloads/slides-2021-10-20-21/08-COVID-Klein-508.pdf

57. Wong, H.L. Surveillance Updates of Myocarditis/Pericarditis and mRNA COVID-19 Vaccination in the FDA BEST System. Vaccines and Related Biological Products Advisory Committee, October 2021. Available online: https://www.fda.gov/media/153090/download

58. Patone, M., Mei, X.W., Handunnetthi, L., Dixon, S., Zaccardi, F., Shankar-Hari, M., Watkinson, P., Khunti, K., Harnden, A., Coupland, C.A.C., Channon, K.M., Mills, N.L., Sheikh, A., Hippisley-Cox, J. Risk of Myocarditis After Sequential Doses of COVID-19 Vaccine and SARS-CoV-2 Infection by Age and Sex. Preprint. medRxiv 2021.12.23.21268276; doi: 10.1101/2021.12.23.21268276.

59. Moderna vaccine becomes third COVID-19 vaccine approved by UK regulator. Press Release. Medicines and Healthcare products Regulatory Agency. 8 January 2021. Available online: https://www.gov.uk/government/news/moderna-vaccine-becomes-third-covid-19-vaccine-approved-by-uk-regulator

60. Patone, M., Mei, X.W., Handunnetthi, L., Dixon, S., Zaccardi, F., Shankar-Hari, M., Watkinson, P., Khunti, K., Harnden, A., Coupland, C.A.C., Channon, K.M., Mills, N.L., Sheikh, A., Hippisley-Cox, J. Risk of Myocarditis After Sequential Doses of COVID-19 Vaccine and SARS-CoV-2 Infection by Age and Sex. Circulation 2022;145: 743–754. doi: 10.1161/CIRCULATIONAHA.122.059970.

61. Buchan, S.A., Seo, C.Y., Johnson, C., et al. Epidemiology of myocarditis and pericarditis following mRNA vaccines in Ontario, Canada: by vaccine product, schedule and interval. Preprint. medRxiv 2021.12.02.21267156; doi: 10.1101/2021.12.02.21267156.

62. Oster, M.E., Shay D.K., Su ,J.R., Gee, J., Creech, C.B., Broder, K.R., et al. Myocarditis Cases Reported After mRNA-Based COVID-19 Vaccination in the US From December 2020 to August 2021. JAMA 2022;327:331–340. doi: 10.1001/jama.2021.24110

63. Husby, A., Hansen, J.V., Fosbøl, E., Thiesson, E.M., Madsen, M., Thomsen, R.W., et al. SARS-CoV-2 vaccination and myocarditis or myopericarditis: population based cohort study. BMJ 2021 375; e068665. doi: 10.1136/bmj-2021-068665

64. Le Vu, S., Bertrand, M., Jabagi, M.J., Botton, J., Drouin, J., Baricault, B., et al. Age and sex-specific risks of myocarditis and pericarditis following Covid-19 messenger RNA vaccines. Nat. Commun. 2022 13;3633. doi: 10.1038/s41467-022-31401-5

65. Su, J.R. Myopericarditis following COVID-19 vaccination: Updates from the Vaccine Adverse Event Reporting System (VAERS). CDC ACIP meeting August 2021. Available online: https://stacks.cdc.gov/view/cdc/109492

66. Su, J.R. Myopericarditis following COVID-19 vaccination : updates from the Vaccine Adverse Event Reporting System (VAERS). CDC ACIP meeting October 2021. Available online: https://www.cdc.gov/vaccines/acip/meetings/downloads/slides-2021-10-20-21/07-COVID-Su-508.pdf . https://web.archive.org/web/20211109123108/https://www.cdc.gov/vaccines/acip/meetings/downloads/slides-2021-10-20-21/07-COVID-Su-508.pdf

67. Halsell, J.S., Riddle, J.R., Atwood, J.E., Gardner, P., Shope, R., et al. Myopericarditis following smallpox vaccination among vaccinia-naive US military personnel. JAMA. 2003;289:3283-9. doi: 10.1001/jama.289.24.3283

68. Lin, A.H., Phan, H.A., Barthel, R.V., Maisel, A.S., Crum-Cianflone, N.F., et al. Myopericarditis and pericarditis in the deployed military member: a retrospective series. Mil Med. 2013;178:18–20. doi: 10.7205/milmed-d-12-00226

69. Sharff, K.A., Dancoes, D.D., Longueil, J.L., Johnson, E.S., Lewis, P.F. Risk of myopericarditis following COVID-19 mRNA vaccination in a large integrated health system: A comparison of completeness and timeliness of two methods. Preprint. medRxiv 2021.12.21.21268209; doi: 10.1101/2021.12.21.21268209.

70. Interim Clinical Considerations for Use of mRNA COVID-19 Vaccines Currently Authorized in the United States. United States Centers for Disease Control and Prevention. Page last reviewed: December 20, 2020. Available online: https://web.archive.org/web/20201230005657/https://www.cdc.gov/vaccines/covid-19/info-by-product/clinical-considerations.html

71. Interim Clinical Considerations for Use of mRNA COVID-19 Vaccines Currently Authorized in the United States. United States Centers for Disease Control and Prevention. Page last reviewed: January 21, 2021. https://web.archive.org/web/20210131024205/https://www.cdc.gov/vaccines/covid-19/info-by-product/clinical-considerations.html

72. Zhang, R., Goswami, J. BLA Clinical Review Memorandum. FDA Office of Vaccines Research and Review. Review Completion Date 28 January 2022. Available online: https://www.fda.gov/media/156342/download.

73. Wong, H.L., Hu, M., Zhou C.K., Lloyd, P.C., Amend, K.L., Beachler, D.C., et al. Risk of myocarditis and pericarditis after the COVID-19 mRNA vaccination in the USA: a cohort study in claims databases. Lancet. 2022;399:2191–2199. doi: 10.1016/S0140-6736(22)00791-7

74. Krug, A., Stevenson, J., Høeg, T.B. BNT162b2 Vaccine-Associated Myo/Pericarditis in Adolescents: A Stratified Risk-Benefit Analysis. Eur J Clin Invest. 2022; 52:e13759. doi:10.1111/eci.13759

75. Comirnaty and Spikevax: possible link to very rare cases of myocarditis and pericarditis. European Medicines Agency. 9 July 2021. Available online: https://www.ema.europa.eu/en/news/comirnaty-spikevax-possible-link-very-rare-cases-myocarditis-pericarditis

76. Altarawneh, H. Chemaitelly, H. Ayoub, H. Tang, P. Hasan, M., et al. Effects of Previous Infection and Vaccination on Symptomatic Omicron Infections. N. Engl. J. Med. 2022;387:21–34. doi: https://doi.org/10.1056/NEJMoa2203965

77. Song, S., Madewell, Z.J., Liu, M., Longini, I.M., Yang, Y. Effectiveness of SARS-CoV-2 vaccines against Omicron infection and severe events: a systematic review and meta-analysis of test-negative design studies. Front. Public Health. 2023;11:1195908. doi: https://doi.org/10.3389/fpubh.2023.1195908

78. Feikin, D.R., Abu-Raddad, L.J., Andrews, N., Davies, M., Higdon, M.M., Orenstein, W.A., Patel, M.K. Assessing vaccine effectiveness against severe COVID-19 disease caused by omicron variant. Report from a meeting of the World Health Organization, Vaccine. 2022;40:3516–3527. doi: https://doi.org/10.1016/j.vaccine.2022.04.069

79. Stowe, J., Andrews, N., Kirsebom, F., et al. Effectiveness of COVID-19 vaccines against Omicron and Delta hospitalisation, a test negative case-control study. Nat. Commun. 2022;13:5736. doi: https://doi.org/10.1038/s41467-022-33378-7

80. Bloomfield, L.E., Ngeh, S., Cadby, G., Hutcheon, K., Effler, P.V. SARS-CoV-2 Vaccine Effectiveness against Omicron Variant in Infection- Naïve Population, Australia, 2022. Emerg. Infect. Dis. 2023;29:1162–1172. doi: https://doi.org/10.3201/eid2906.230130

81. COVID-19 vaccine safety report – 12-01-2023. Government of Australia 12 January 2023. Available online: https://www.tga.gov.au/news/covid-19-vaccine-safety-reports/covid-19-vaccine-safety-report-12-01-23#comirnaty-pfizer-mrna-vaccines

82. Lind, M.L., Robertson, A.J., Silva, J., Warner, F., Coppi, A.C., et al. Association between primary or booster COVID-19 mRNA vaccination and Omicron lineage BA.1 SARS-CoV-2 infection in people with a prior SARS-CoV-2 infection: A test-negative case-control analysis. PLoS Med. 2022;19:e1004136. doi: https://doi.org/10.1371/journal.pmed.1004136

83. Lipsitch, M., Goldstein, E,, Ray, G.T., Fireman, B. Depletion-of-susceptibles bias in influenza vaccine waning studies: how to ensure robust results. Epidemiol Infect. 2019;147:e306. doi: https://doi.org/10.1017/S095026881900196160

Appendix S1: An Analysis of Calendar Year 2020 COVID Incidental-Hospitalization Rates and Ancestral-Strain IHR Based on Data from Connecticut and the CDC

In this appendix, we show how data from Connecticut and the CDC provides evidence that incidental COVID-hospitalization rates were substantial for calendar year 2020 and were especially high for the young. We also show this data suggests that the ancestral-strain IHR for 15–24 year-olds in Connecticut was likely less than 0.0895%, which via the Herrera-Esposito IHR model, suggests a corresponding IHR for 18–25 year-olds in Connecticut was likely less than 0.103%. These IHR estimates are roughly consistent with our modeling based on COVID hospitalization and infection data from Ontario over the period 1/1/22–5/31/22, which suggests 0.100% is a good estimate for the Omicron IHR for unvaccinated 18–29 year-olds in Ontario (see Section S8 of Supplement S2) All these estimates are for a general-population mix of males and females. Thus, the Omicron IHR of 0.14% we use in our modeling for 18–25-year-olds males in the U.S. is likely a reasonable estimate of the Omicron IHR for a general-population mix of males and females in the U.S. of ages 18–25.

Hospitalization Table H-1 from reference [A1.1] provides the following COVID-19 hospitalizations data for Connecticut by age group (*without inclusion of incidental hospitalizations*).

Table S1.4: Connecticut COVID Hospitalization Rates by Age, Calendar Year 2020

Age Group	2020 COVID Hospitalizations	Population*	2020 COVID-Hospitalization Rate per 100,000 Population
0–4	36	180,713	19.92
5–14	31	417,411	7.43
15–24	102	480,874	21.21
25–44	951	890,203	106.83
45–64	3351	996,424	336.30
65+	6129	634,635	1831.55

*From [A1.2], Year 2020. Hospitalization rates per 100,000 population are provided in [A1.1] based on population in the year 2000.

Now consider the CDC data from Table 2 of [A1.3]:

Table 2: Estimated rates of COVID-19 disease outcomes, per 100,000, by age group — United States, February-December 2020

	Infection rate per 100,000		Symptomatic Illness rate per 100,000		Hospitalization rate per 100,000	
Age group	Estimate	95% UI*	Estimate	95% UI*	Estimate	95% UI*
0-4 yrs	15,333	12,880 – 18,408	13,068	11,637 – 14,847	190	157 – 228
5-17 yrs	27,218	22,837 – 32,644	23,201	20,640 – 26,406	187	148 – 233
18-49 yrs	30,602	25,595 – 36,888	26,112	23,007 – 29,776	776	657 – 928
50-64 yrs	22,966	19,218 – 27,634	19,588	17,300 – 22,343	1,619	1,425 – 1,848
65+ yrs	16,722	13,460 – 21,087	13,554	12,031 – 15,398	3,485	3,065 – 4,003
All ages	25,412	21,905 – 29,726	21,532	19,305 – 24,180	1,254	1,118 – 1,423

* Adjusted estimates and rates are presented in two parts: an uncertainty interval [UI] and a point estimate. The uncertainty interval provides a range in which the true number or rate of COVID-19 infections, symptomatic illnesses, or hospitalization would be expected to fall if the same study was repeated many times, and it gives an idea of the precision of the point estimate. A 95% uncertainty interval means that if the study were repeated 100 times, then 95 out of 100 times the uncertainty interval would contain the true point estimate. Conversely, in only 5 times out of a 100 would the uncertainty interval not contain the true point estimate.

The large difference in the Connecticut and CDC 2020 COVID-hospitalization rates (per 100,000 population) is likely due to three factors: the CDC's inclusion of incidental COVID hospitalizations in its accounting, Connecticut's population being healthier than that of many other states (e.g., in its 2020 state-rankings, Sharecare ranks Connecticut 7th in terms of well-being [A1.4]), and overall COVID infection rate in CT for 2020 being about 87% of that in the United States as a whole [A1.5]. It's also possible that

the large difference in Connecticut and CDC 2020 COVID-hospitalization rates is partially due to flaws in CDC modeling.

In the table below, we present a comparison of 2020 COVID-hospitalization data from Connecticut versus nationwide data from the CDC. In the third column, we provide parenthetically adjustments to CDC rates based on (i) the assumption that the overall COVID infection rate in CT for 2020 was about 87% of that in the United States as a whole, as well as (ii) the assumptions that the overall health of those in Connecticut is better than average for the U.S. The specific adjustment for better health, multiplying the CDC rates by 0.77, results from the assumption that the health of those in Connecticut is comparable to the heath of Canadians—see Section S4 of Supplement S2. The total parenthetical adjustment in the third column results from multiplying the CDC rate by the product $0.87 \cdot 0.77 \approx 0.67$.

Table S1.5: Comparing 2020 COVID Hospitalization Rates: Connecticut vs. Nationwide Rates Provided by the CDC

Age Group	2020 COVID-Hospitalization Rate per 100,000 Connecticut	2020 CDC COVID-Hospitalization Rate per 100,000*	Percentage by which the Connecticut's rate is lower than the CDC's
0–4	19.92	190 (127)	89.5 (84.3)
5–14	7.43	148–187 (99–125)	95.0–96.0 (92.5–94.0)
15–24	21.2	187–233 (125–156)	88.7–90.9 (83.0–86.4)
25–44	106.83	657–776 (440–520)	83.7–86.2 (75.7–79.5)
45–64	336.30	776–1425 (520-955)	56.7–76.4 (35.3–64.8)
65+	1831.55	3485 (2335)	47.4 (21.6)

* For age ranges for which a direct comparison is not possible, we make choices designed to reduce the difference in rates, comparing: CT's rate for 5–14 with the range extending from the lower bound of the CDC's UI for 5–17 to the rate for 5–17, CT's rate for 15–24 with the range extending from CDC's rate for 5–17 to the upper bound of the UI for 5–17, CT's rate for 25–44 with the range extending from the lower bound for the CDC's UI for 18–49 to the rate for 18–49, and CT's rate for 45–64 with the range extending from the CDC's rate for 18–49 to the lower bound of the UI for 50–64.

Observe, in particular, that the percentages in the final column of the preceding table are consistent with data discussed in Section S2 of this supplement suggesting incidental-COVID hospitalization rates are higher for younger age groups.

Rather than reducing CDC rates to approximate Connecticut rates and attempting to estimate CDC rates with a finer age stratification, we now take the opposite approach—increasing Connecticut rates to approximate national, CDC rates. To obtain approximate 2020 COVID hospitalization rates in Connecticut for the CDC age ranges 5–17 and18–49, we use weighted averages of Connecticut's rates for the ranges 5–14, 15–24, 25–44, and 45–64. Because the range 5–14 constitutes 10 of the 13 years of the range 5–17, we estimate the Connecticut COVID hospitalization rate for 5–17 as

$$\frac{10}{13} \cdot 7.43 + \frac{3}{13} \cdot 21.2 \approx 10.61 \ (per \ 100{,}000).$$

Similarly, for the 18–49 range, we estimate the Connecticut rate to be

$$\frac{7}{32} \cdot 21.21 + \frac{20}{32} \cdot 106.83 + \frac{5}{32} \cdot 336.30 \approx 123.96 \ (per \ 100{,}000).$$

We obtain similar rates if we fit an exponential model $M(t) = ae^{bt}$ to Connecticut's data for ranges 5–14, 15–24, and 25–44 (using the same method we applied in Section S2 of this supplement relative to IHR data from [A1.6]).[46]

To obtain a COVID-hospitalization rate estimate for the CDC age range 50–64, we will fit an exponential model $M(t) = ae^{bt}$ to Connecticut data for the age ranges 5–14,15–24, 25–44, and 45–64. The

[46] $M(t) = 2.72e^{0.10t}$ well fits the data, and its average value over [5,18] is approximately 9.21 while our weighted average estimate for the age range 5–17 is 10.61; the average of $M(t)$ over [18,50] is 121.0 while our weighted average estimate for the age range 18–49 is 123.6.

function $M(t) = 9.264e^{0.0642t}$ is an (approximate) least-squares best-fitting model, but the sum of squares of errors is rather large (about 450). However, the average value of $M(t)$ over [45, 65] is approximately 338.6, which is fairly close to the Connecticut rate 336.3 for the age range 45–64. The average value of $M(t)$ over [50,65] is 386.06, which we take as an estimate for Connecticut COVID-hospitalization rate per 100,000 population for the age range 50–64.

To estimate national COVID-hospitalization rates from Connecticut rates, we divide Connecticut rates by 0.67, to adjust the rates upward to account for, as above, a lower COVID infection rate in Connecticut and better than average health of Connecticut residents.

Tabulating our estimates based on Connecticut data as well as CDC data, we have the following.

Table S1.6: Comparing 2020 COVID Hospitalization Rates: Nationwide Rate Suggested by the CDC vs. Nationwide Rate Suggested by Data from Connecticut.

Age Group	2020 CDC National COVID-Hospitalization Rate per 100,000*	National 2020 COVID-Hospitalization Rate per 100,000 Suggested by Connecticut Data**	Possible Percentage Overestimate By the CDC
0–4	190	29.7 (≈19.92/0.67)	540%
5–17	187	15.8 (≈10.61/0.67)	1084%
18–49	776	185.0 (≈123.96/0.67)	319%
50–64	1619	576.2 (≈386.06/0.67)	181%
65+	3485	2733.7 (≈1831.55/0.67)	27%

*From Table 2 of [A1.3] (reproduced on page 1 of this appendix)
**Derived from Connecticut rates of Table S1.4 as described in the four paragraphs preceding this table

We now obtain an approximation of the IHR for the ancestral strain of COVID-19 for the age range 15–24 in Connecticut using data from Table S1.4 as well as the CDC's Table 2 from [A1.3] . From Table S1.4, we know there were 102 COVID hospitalization among 480,874 persons 15–24 years old in Connecticut during calendar year 2020. We need to estimate the number of COVID-19 infections that occurred among the 480,873 persons in this age range. In order to more likely underestimate the number of infections than to overestimate the number, we will use the 27.218% infection-rate estimate for the 5–17 age range in the CDC's Table 2 (reproduced above) rather than the higher estimate for the 18–49 range. Moreover, we will reduce the 27.218% estimate using additional CDC data. The CDC COVID Data Tracker, updated 31 December 2020, reports the nationwide COVID-case rate to be 5,923 cases per 100,000 population and a corresponding rate for Connecticut of 5,151 cases per 100,000, a case rate approximately 87% of the national rate [A1.5]. Let's assume the 2020 infection rate among those 15–24 in Connecticut is also 87% of our estimated national rate for this age group 27.218%, so that we arrive at the estimated infection rate of about 23.7% for 15–24 year-olds in Connecticut during 2020. This yields an approximate IHR for this age range of

$$\frac{102}{0.237 \times 480{,}874} \approx 0.0895\%.$$

The ratio of IHRs for the ranges 18–25 and 15–24, according to the Herrera-Esposito model [A1.6], is

$$\frac{\int_{18}^{26} 0.0714\, e^{0.0750t} dt/8}{\int_{15}^{25} 0.0714\, e^{0.0750t} dt/10} \approx 1.152,$$

which suggests a COVID-19 IHR for 18-25 year-olds in Connecticut based on calendar year 2020 data of approximately 0.103% ($\approx 0.0895\% \cdot 1.152$). If we assume the health of those in Connecticut is comparable to the heath of Canadians, then we might adjust this IHR of 0.103% upward through division by 0.77 to approximate a nationwide ancestral-strain IHR for the U.S. based on the 2020 data discussed above: $0.103\%/0.77 \approx 0.134\%$. This suggests that the Omicron IHR of 0.14% we use in our modeling for 18–25-year-olds males in the U.S. is a reasonable estimate of the Omicron IHR for a general-population mix of males and females in the U.S. We remark that in contrast to data from Table 2 of [A1.7] and Table 1 of [A1.8], Connecticut's data for the 15–24 age group shows males and females having nearly identical hospitalization rates per 100,000 population 21.19 males and 21.24 females.

The Herrera-Esposito model [A1.6] suggests an IHR for 15–24 year-olds of about 0.322%:

$$\frac{1}{10}\int_{15}^{25} 0.07014\, e^{0.0750t} dt \approx 0.322$$

Assuming incidental hospitalizations account for the difference between the 0.322% ancestral-strain IHR from the modeling of [A1.6] and the corresponding IHR of 0.0895% derived above from Connecticut and CDC data, the incidental rate for 2020 among 15–24 year-olds would be about 72.2%. This is consistent with Figure 2 of [A1.9], which indicates an incidental COVID-hospitalization rate of 70% or higher among 0–18 year-olds from about 1 May 2020 through December of 2020.

References:

[A1.1] Hospitalization Statistics. Hospitalization Tables for 2020. Connecticut State Department of Health. Available online: https://portal.ct.gov/dph/health-information-systems--reporting/hisrhome/hospitalization-statistics
[A1.2] Annual State & County Population with Demographics. State-level ASRH estimate tables in Excel By 18 Age Groups: 2020–2022. Connecticut State Department of Health. Available online: https://portal.ct.gov/dph/health-information-systems--reporting/population/annual-state-county-population-estimates
[A1.3] Estimated Disease Burden of COVID-19. Updated 19 January 2021. United States Centers for Disease Control and Prevention. Available online: https://web.archive.org/web/20210408012037/https://www.cdc.gov/coronavirus/2019-ncov/cases-updates/burden.html
[A1.4] Sharecare's Community Well-Being Index 2020 State Rankings Report. Available online: https://wellbeingindex.sharecare.com/wp-content/uploads/2021/05/Sharecare-Community-Well-Being-Index_2020-State-Rankings-vFINAL.pdf
[A1.5] CDC COVID Data Tracker. United States COVID-19 Cases and Deaths by State, Updated 31 December 2020. United States Centers for Disease Control and Prevention. Available online: https://web.archive.org/web/20210102050424/https://covid.cdc.gov/covid-data-tracker/#cases_casesper100k
[A1.6] Herrera-Esposito, D., de los Campos, G. Age-specific rate of severe and critical SARS-CoV-2 infections estimated with multi-country seroprevalence studies. BMC Infect. Dis. 2022; 22:311-325. https://doi.org/10.1186/s12879-022-07262-0
[A1.7] Funk, P.R., Yogurtcu, O.N., Forshee, R.A., Anderson, S.A., Marks, P.W., Yang, H. Benefit-risk assessment of COVID-19 vaccine, mRNA (Comirnaty) for age 16–29 years. Vaccine 2022; 40: 2781–2789. doi: 10.1016/j.vaccine.2022.03.030
[A1.8] Kaim, A., Shetrit, S.B., Saban M. Women Are More Infected and Seek Care Faster but Are Less Severely Ill: Gender Gaps in COVID-19 Morbidity and Mortality during Two Years of a Pandemic in Israel. Healthcare (Basel) 2022; 10:2355. doi: 10.3390/healthcare10122355
[A1.9] Thayer, J., Miller, A., Hirz, K., Poxner, X., Sandberg, N., Vander Berg, D., et al., A Bright Side: Hospitalizations for COVID-19 Might Be Overcounted, Especially Among Kids, Epic Research 2022. Available online: https://www.epicresearch.org/articles/a-bright-side-hospitalizations-for-covid-19-might-be-overcounted-especially-among-kids

Appendix S2: Further Analysis of VAM/P Risk for mRNA-1273, Including, for Dose 2, Consideration of Risk Variation Based on Dosing Interval as well as Homologous vs. Heterologous Primary-Series Schedule

In this appendix, we discuss VAM/P data from the United States, Canada, England, France, and Scandinavia. Because of variations in public-health guidance during the year 2021, heterologous primary-series dosing (for which the 1st dose manufacturer differs from that of the 2nd) was rare in the U.S., England, and France, but fairly common in Canada and Scandinavia. Also, public-health guidance on the *dosing interval*, the time between the 1st and 2nd doses of an individual's primary series COVID-vaccination, varied by country; however, the manufacturer's dosing-interval guidance was as follows: 3 weeks for Pfizer's BNT162b2 and 4 weeks for Moderna's mRNA-1273. See Section S2.5 of this appendix for a discussion of 2021 public-health directives concerning primary-series COVID vaccination in the United States, Canada, England, France, and Scandinavia.

In evaluating the VAM/P risk of primary series mRNA-1273 vaccination, we should focus on the homologous series for which both the 1st and 2nd doses are mRNA-1273.

S2.1. VAM/P-rate estimates for dose 2 of the mRNA-1273 homologous primary series, based on data available before 1/1/22

Based on data available before 1/1/22, we estimated in Section 1.4 of the main exposition that the completion of 1 million mRNA-1273 primary series among males 18–25 carried a risk of 268 VAM/P hospitalizations (18 following dose 1 and 250 following dose 2). We chose our dose 1 estimate from the FDA's BLA-Memo Scenario (see, e.g., Section S6.7 of this supplement). Our dose 2 estimate of Table 2 of the main exposition was derived from the following four sources: Buchan et al. [A2.1, A2.2], Patone et al. [A2.3,A2.4], (iii) Sharff et al. [A2.5,A2.6], and the FDA (with VSD-based adjustments) [A2.7, A2.8].

In addition to providing incidence-rate estimates for myocarditis/pericarditis following mRNA-1273 vaccination, Buchan et al. [A2.1, A2.2] also found that the incidence rate for dose 2 of mRNA-1273 depends on both the type of the corresponding dose 1 and the dosing interval. These findings are described in Table 3 of [A2.1] or eTable 5 of [A2.2]:

eTable 5. Rates[a] of reported myocarditis and pericarditis per million doses administered and 95% CI among males aged 18-24 years by vaccine product and interdose interval with dose 2 on or after June 1, 2021 **[to the end of the study period Sept 4, 2021]**

Vaccine schedule	Reports (N)	Doses administered (N)	Reported rate (95% CI) per million doses
People with two doses			
Homologous schedule			
BNT162b2-BNT162b2	11	235,819	46.6 (23.3 – 83.5)
Interval ≤30 days	2	21,160	94.5 (11.4 – 341.4)
Interval 31-55 days	8	124,235	64.4 (27.8 – 126.9)
Interval ≥56 days	1	90,424	11.1 (0.3 – 61.6)
mRNA-1273-mRNA-1273	27	93,616	288.4 (190.1 – 419.6)
Interval ≤30 days	4	10,623	376.5 (102.6 – 964.1)
Interval 31-55 days	20	60,352	331.4 (202.4 – 511.8)
Interval ≥56 days	3	22,641	132.5 (27.3 – 387.2)
Heterologous schedule			
mRNA-1273-BNT162b2	0	8,853	NR
Interval ≤30 days	0	1,058	NR
Interval 31-55 days	0	5,402	NR
Interval ≥56 days	0	2,393	NR
BNT162b2-mRNA-1273	29	85,893	337.6 (226.1 - 484.9)
Interval ≤30 days	6	7,720	777.2 (285.2 - 1691.6)
Interval 31-55 days	20	62,717	318.9 (194.8 - 492.5)
Interval ≥56 days	3	15,456	194.1 (40.0 - 567.2)

[a] Estimates were not provided for strata with 0 reported events. NR, not reported.

The data in the preceding table suggests that among males 18–24 the heterologous series BNT162b2-mRNA-1273 carries more VAM/P risk than does the homologous series mRNA-1273-mRNA-1273 and that VAM/P risk decreases as dosing-interval increases. Also observe from the table that of the primary series having 2nd dose mRNA-1273, 48.8% were heterologous (BNT162b2-mRNA-1273) and 52% were homologous (mRNA-1273-mRNA-1273); moreover, a strong majority of mRNA-1273 2nd doses were delivered with an extended dosing interval between 31 and 55 days (inclusive).[47] That a significant number of heterologous series with 2nd dose being mRNA-1273 were administered in Ontario during the summer of 2021 is also clear from the Ontario vaccine uptake report [A2.9] presenting data trough 9/4/2021: among 18–29 year-olds, Table 2 reports 444,609 1st doses of mRNA-1273 completed and 653,110 2nd doses completed.

At the time of the FDA's mRNA-1273 assessment (3rd week of January 2022), the recommended dosing interval for BNT162b2 was 3 weeks and that for mRNA-1273 was 4 weeks However, near the end of January 2021 [A2.10] the CDC introduced more flexibility in dosing-interval length as follows:

[47] Based on the row for 18–29 year-olds of Table 2 of the Ontario vaccine uptake report [A2.9], we see that during the period 6/1/21–9/4/2021 at most 255 (= 68 + 451 - 264) primary series with first dose ChAdOx1 (AstraZeneca) were administered to 18–29 year-old Ontarians, males & females.

> The second dose should be administered as close to the recommended interval as possible. However, if it is not feasible to adhere to the recommended interval, the second dose of Pfizer-BioNTech and Moderna COVID-19 vaccines may be scheduled for administration up to 6 weeks (42 days) after the first dose. … Both doses of the series should be completed with the same product. … In exceptional situations in which the first-dose vaccine product cannot be determined or is no longer available, any available mRNA COVID-19 vaccine may be administered at a minimum interval of 28 days between doses to complete the mRNA COVID-19 vaccination series.

Note also the preceding's directive "Both doses of the series should be completed with the same product." As we have already mentioned, owing to public-health agency recommendations, during 2021 the heterologous primary-series schedule BNT162b2, then mRNA-1273 was rare in U.S., England, and France.

Given the limited amount of data provided in eTable 5 above for the homologous schedule mRNA-1273-mRNA-1273 with dosing interval < 30 days, we will obtain from eTable 5 a VAM/P-risk estimate for dose 2 of the homologous mRNA-1273 series assuming a dosing interval < 56 days (longer than the maximum recommended interval according to the CDC guidance quoted above of 42 days). In Table 4 of [A1.1], Buchan et al. indicate that during the period 1 June 2021 through 4 September 2024 among males 18–24 receiving dose 2 of mRNA-1273, there were 55 cases of myocarditis/pericarditis *occurring in a 7-day risk window following dose 2.* Note that in eTable 5 a total of 56 such cases (but not necessarily occurring during the 7-day risk window postdose 2) are identified with 29 associated with dose 2 of the heterologous schedule BNT-162b2-mRNA-1273 and 27 with the homologous mRNA-1273 schedule. Thus, we know from Table 4 of [A1.1] that 55 of these 56 cases occurred within 7 days after dose 2. Thus, for the homologous schedule at least 26 of the 27 cases occurred within 7 days of dose 2, so that at least 23 of the 24 cases with dosing gap < 56 days also occurred during the 7-day risk window after dose 2. Thus, based on Buchan et al.'s eTable 5, we have a myocarditis/pericarditis incidence rate of at least $23/0.070975 \approx 324.1$ cases per million 2nd doses of mRNA-1273, assuming a dosing interval < 56 days, a homologous schedule, and a 7 day-risk window postvaccination. Applying our background rate of 4 cases per million per week (see Section S6.3 of this supplement), we obtain a VAM/P-rate estimate of 320 cases per million 2nd doses of mRNA-1273 among males 18–24, assuming a dosing interval < 56 days and a homologous schedule. Observe this VAM/P-rate estimate of 320 per million exceeds the estimate we used of 302 per million in Table 3 of the main exposition. Also observe that the other VAM/P-rates contributing to Table 3 derive from data from the U.S. and England. Thus, the following modified version of Table 3 provides an approximation of VAM/P risk associated with the homologous mRNA-1273 primary series with a dosing-interval restriction < 56 days for column-3 data.

Estimated VAM/P events per million 2nd Doses of the homologous mRNA-1273 primary series administered to males 18–24/18–25 based on data available before 1 January 2022. (For the data of column 3, dosing interval restricted to < 56 days; unrestricted for columns 4–6.)

Description	FDA Scenarios 1–4 (Scenarios 5 & 6) [1, Table 2]	Buchan et al. [59]	Patone et al. [56,59]	Sharff et al. [67]	Data From FDA & VSD [25, 54, 55]	Weighted Average of Highlighted Data
Cases per millon	128 (68 & 241)	320*	269	525	260	306†
Hospitalizations per million	110 (58 & 207)	256	231	452	224	257‡
Number of events on which estimate is based	21	23	23	7	22	Total Events Columns 3–6 75

* Homologous mRNA-1273 schedule, dosing interval < 56 days (CDC recommendation "The second dose should be administered as close to the recommended interval as possible [28 days for mRNA-1273]. However, if it is not feasible to adhere to the recommended interval, the second dose of Pfizer-BioNTech and Moderna COVID-19 vaccines may be scheduled for administration up to 6 weeks (42 days) after the first dose.

† $306 \approx 320 \cdot \frac{23}{75}. + 269 \cdot \frac{23}{75} + 525 \cdot \frac{7}{75} + 260 \cdot \frac{22}{75}$ ‡ $257 \approx 256 \cdot \frac{23}{75}. + 231 \cdot \frac{23}{75} + 452 \cdot \frac{7}{75} + 224 \cdot \frac{22}{75}$

The maximum dosing-interval recommendation of 6 weeks was in place in the U.S. (until February of 2022). However, England recommended more flexibility in dosing-interval, "Operationally, it is recommended that the second dose of both vaccines should be routinely scheduled between four and 12

weeks after the first dose." See Section S2.5 of this appendix for a discussion of dosing-interval recommendations, with references provided.

S2.2 VAM/P-rate estimates for dose 2 of the mRNA-1273 homologous primary series, based on data available only after 1/22/22

On 7/27/2025, we executed a PubMed search without restrictions on "COVID AND vaccin* AND myocarditis AND meta*", and 93 results were returned, only one of which was a meta-analysis of COVID-vaccine related myocarditis/pericarditis providing estimates of risks associated with mRNA-1273 significantly stratified by age and sex: "Age- and sex-stratified risks of myocarditis and pericarditis attributable to COVID-19 vaccination: a systematic review and meta-analysis" by Kitano et al. [A2.11].

Kitano et al.'s analysis of attributable (excess) risk per 100,000 2nd doses of mRNA1273 among males 18–24 is presented in Table 2 of [A2.11] as follows:

Table 2. Summary of findings on the risk ratio and attributable risk of combined myocarditis and pericarditis after mRNA-1273 vaccine.[a]

Age group, years	Sex	Dose	Risk ratio (95% CI)	No. of studies for risk ratio	Attributable risk per 100 000 doses (95% CI)	No. needed to harm[b]	No. of studies for attributable risk
18-24	Male	First	2.61 (2.11-3.24)	4	2.84 (1.34-4.33)	35 211	3
		Second	14.14 (8.35-23.96)	4	20.02 (10.47-29.57)	4995	4
		Booster	–	1	–	–	1

Observe that the attributable-risk estimates of the preceding table are described as being for "combined myocarditis and pericarditis" (i.e., myocarditis or pericarditis (or both)); however, the details of the pooled analysis of risk over the 4 sources used, appearing in supplementary Figure S5 of [A2.11], reveal that for one of the 4 sources, separate estimates of myocarditis and pericarditis risks are included:

Figure S5. "Attributable risk of myocarditis, pericarditis, or combined myocarditis or pericarditis[a]

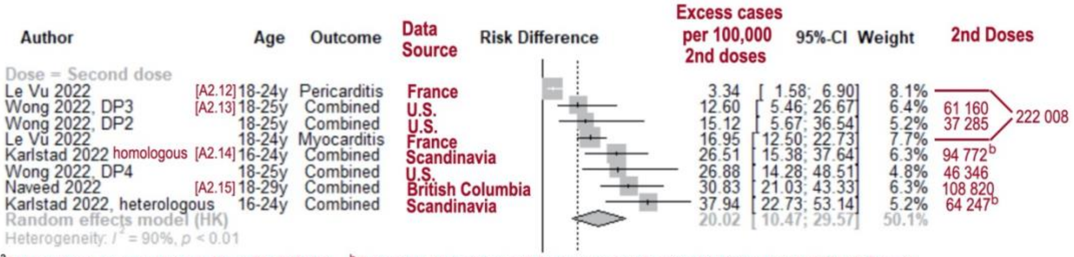

[a]Text and lines in magenta added (to Figure S5 of [A2.11]) [b]Estimated number of males 16–24 receiving dose 2 obtained by dividing total by 2 [A2.14, Table 1]

Not only are the risks of pericarditis and myocarditis found by Le Vu 2022 [A2.12] not combined in the preceding analysis of Kitano et al.'s Figure S5 but also note that, among the rates included, the risk for pericarditis alone is given the greatest weight 8.1%. We emphasize that the provided weights, which sum to 50% (0.50), are applied as expected to obtain the weighted average of approximately 20.02:

$$\frac{3.34 \cdot 0.081 + 12.60 \cdot 0.064 + 15.12 \cdot 0.052 + 16.95 \cdot 0.077 + 26.51 \cdot 0.063 + 26.88 \cdot 0.048 + 30.83 \cdot 0.063 + 37.94 \cdot 0.052}{0.50} \approx 20.09$$

We now observe that obtaining a combined-risk estimate (rather than separate ones for pericarditis and myocarditis) based on Le Vu et al.'s data [A2.12] is straightforward—we need only add the myocarditis and pericarditis risks together, obtaining 16.95 + 3.34 ≈ 20.29 attributable cases per 100,000 2nd doses of mRNA-1273. Moreover, all these cases are hospitalized, which is not true for the other included studies. To see that adding risk estimates for myocarditis and pericarditis from Le Vu et al.'s study yields combined risk, consider the following from "Characteristics of the study population" [A2.12, p.2]:

> Between May 12, 2021 and October 31, 2021, within a population of 32 million persons aged 12 to 50 years, 21.2 million first (19.3 million second) doses of the BNT162b2 vaccine and 2.86 million first (2.58 million second) doses of the mRNA-1273 vaccine were received (Table S1). In the same period, 1612 cases of myocarditis (of which 87 [5.4%] had also a pericarditis as associated diagnosis) and 1613 cases of pericarditis (37 [2.3%] with myocarditis as associated diagnosis) were recorded in France.

Thus, Le Vu et al.'s analysis of myocarditis risk is restricted to those myocarditis cases having a primary discharge diagnosis of myocarditis while their analysis of pericarditis risk is restricted to those having a primary discharge diagnosis of pericarditis. Thus, our adding attributable risks for each provides combined risk.

Our goal is to produce a new version of Figure S5 that corrects the incompatibilities in the data presented and applies a more reasonable weighting scheme. We will call the new version of Figure S5, "Figure S5m," where m = modified.

We have already expressed some concern about the high weighting given the lowest rate of Figure S5–-that for pericarditis alone from [A2.12]. Here is another weighting concern. "Wong 2022" [A2.13] presents the FDA's BEST-System data from its four data partners—the same partners that provided data for the FDA's mRNA-1273 benefit-risk assessment [A2.16] but with data collected over a longer time period.[48] Incidence rates from each of the 4 partners are reported in Table 2 of [A2.13]. However, Kitano et al. [A2.11] appear not to make use of the estimates from Table 2 of Wong 2022; rather, they appear to rely on odds-ratio estimates from the supplement of Wong et al.'s study [A2.13, Table S3], with the estimates presented only for three data partners DP2, DP3, and DP4. This means that Kitano et al. exclude data from DP1, which, based on Table 2 of [A2.13], reports for the 2nd dose of mRNA-1273 among males 18–25, the highest number of myo/pericarditis events (13) , highest number doses (94,629), and the 2nd highest incidence rate per 100,000 person-days (2.1). Equally concerning is that, according to Table 2 of [A2.13] the total number of 2nd doses of mRNA-1273 reported by DP2, DP3, and DP4 is 144,791; whereas, the number of 2nd doses of mRNA-1273 contributing to the analysis of hospitalized myo/pericarditis events by Le Vu et al. is 222,008 [A2.12, Table S1]. Thus, Kitano et al.'s attributing in their Figure S5 analysis a combined weight of 16.4% to DP2, DP3 and DP4 and of only 15.6% to Le Vu et al.'s estimates seems unreasonable. One would expect a weighting in which the combined weight of DP2, 3, and 4 would be roughly 65% ($\approx 144791/222008 \cdot 100\%$) of that of Le Vu 2022 [A2.12]. The most natural way to weight the findings of the various studies mentioned in Figure S5 is to use number of doses, which we have inserted on the right of Figure S5 above.

So far, we have addressed two concerns about Figure S5: by combining the separate pericarditis and myocarditis rates of Le Vu et al. [A2.12] and by using numbers of doses administered to determine weighting. There are additional issues relating to incompatibilities among the rates provided in Figure S5 that we also address to produce our modified Figure S5m. As already noted, the VAM/P rate of 20.29 per 100,000 based on Le Vu et al.'s data is 20.29 *VAM/P hospitalizations* per 100,000 2nd doses of mRNA-1273 while the other entries are VAM/P case rates including some non-hospitalized cases. The rate of Karlstad 2022 derives from "either inpatient or outpatient hospital care" [A2.14, p. 600]; the rate of Naveed 2022 derives from hospital admissions or emergency department visits [A2.15, pE1529], while the rate of Wong 2022 derives from "inpatient facilities or emergency department settings" [A2.13, p. 2193].

To correct, at least partially, this incompatibility of data from Le Vu 2022 vs. that of the other included studies, we will assume an 80% VAM/P case-hospitalization rate (as we did for Buchan et al.'s study [A2.1, A2.2]), which yields 25.36 ($\approx 20.29/0.80$) VAM/P cases per 100,000 2nd doses of mRNA-1273 among males 18–24, which we will use in Figure S5m as an approximation for a VAM/P case rate derived from Le Vu et al.'s study [A2.12]

Another incompatibility in the data of Figures S5 of [A2.11] are the age ranges: while it's not unreasonable to assume that rates for 18–24, and 16-24 well approximate rates for the range 18–25 used by FDA for its mRNA-1273 analysis, the rate for the range 18–29 of Naveed 2022 [A2.16] will produce an underestimate of that for the range 18–24/25; in fact, we, conservatively, estimate that the VAM/P case rate 30.83 per 100,000 2nd doses among 18–29-year-old males corresponds to a rate of at least 35.74 cases per 100,000 among 18–24-year-old males—see Section S2.6 of this appendix for the argument.

Yet another incompatibility in the rates displayed above in Figure S5 of [A2.11] is that some of the rates correspond to the homologous schedule mRNA-1273-mRNA-1273 (true of the first Karlstad et al. entry and essentially true for Le Vu 2022's rate based on data from France and Wong 2022's rate based

[48] Data cutoffs stated in Section 2.3.1 of [A2.16] being August 21, 2021 (DP1), July 10, 2021 (DP2), July 31, 2021 (DP3), and June 30, 2021 (DP4), and the cutoffs of [A2.13], stated on p. 2192, being September 30, 2021 (DP1), Oct 31, 2021 (DP2), Nov 4, 2021 (DP3), or Dec 25, 2021 (DP4)

on data from the U.S., while that for Naveed et al. is mixed but still mostly homologous). In Section S2.6 below, we estimate that the data of Naveed 2022 suggests a VAM/P case rate of at least 33.50 for the 2nd dose of the homologous mRNA-1273 primary series among males 18–24. *Rather than use this higher rate, we will simply use the rate of 30.83 per 100,000 found for males 18–29 as an underestimate of the rate for males 18–24/25 assuming homologous mRNA-1273 dosing*.[49]

Before presenting our Figure S5m incorporating all the compatibility adjustments discussed above, we need to discuss further the data in Figure S5 for "Wong 2022". Kitano et al. [A2.11] re-analyze the FDA's data from Wong 2022 [A2.13], mentioning in their supplementary material, "For the study by Wong et al., the sample size ratio of vaccinated vs control groups was estimated from the vaccine coverage rate by age groups and vaccine type in United States as of September 30, 2021 to implement treatment arm continuity correction for zero outcome cells." Wong 2022 provides a meta-analysis of the data from all four data partners through their Table 2, finding an incidence rate of 2.17 (1·55 to 3·04) per 100,000 person days of risk following dose 2 of mRNA-1273 [A2.13]. Using a weighted-average of the three background rates provided in Supplemental Table 3 of [A2.13] of 0.12 cases per 100,000 person days of risk, we obtain an excess myo/pericarditis case rate of 2.05 cases per 100,000 person days of risk. With a 7 day risk period following each 2nd dose, the number of doses corresponding to 100,000 person days of risk is 100000/7. Thus, the VAM/P case rate suggested by the data of Table 2 of [A2.13] is 143.5 (= 7· 2.05) cases per 100,000 2nd doses of mRNA-1273 among males 18–25. Assuming an 86% hospitalization rate, as did the FDA, we arrive at an estimated VAM/P hospitalization rate of about 124 cases per million 2nd doses of mRNA-1273 among males 18–25. Given the shortcomings of the FDA's BEST system described by the FDA itself (e.g., "small sample sizes and imperfect sensitivity of ICD-10 codes to identify these rare outcomes" [A2.7, Section 4]) and Sharff et al.'s finding announced in late December of 2021, "The true incidence of myopericarditis is markedly higher than the incidence reported to US advisory committees" [A2.6], we have disregarded Wong 2022's outlier rate in our Figure S5m below.

Figure S5m. Estimates of attributable risk for young males of combined myocarditis or pericarditis following dose 2 of mRNA-1273, assuming dose 1 is also mRNA-1273 and no restrictions on dosing interval

Author	Age	VAM/P rate per million 2nd doses	Number of 2nd doses
Le Vu 2022 [a]	18–24	253.6	222,008
Karlstad 2022[b]	16-24	265.1	94,772
Naveed 2022 [c]	18-29	308.3	108,820
Wong 2022	18–25	143.5	239,420
		Weighted Average of rates, rows 1–3 [d]: 270.1	Total (excluding Wong 2022): 425600

[a] Column-3 rate derived from Figure 3 of [A2.12], assuming an 80% VAM/P case hospitalization rate—see the discussion above between Figures S5 and Figure S5m; Column 4: [A2.12, Table S1]

[b] Column-3 rate is the mRNA-1273/mRNA-1273 rate from Table 3 of [A2.14]; Column 4: we have divided by 2, the number 189,545 of 2nd doses received by males and females 16–24 [A2.14, Table 1], where we assume the number of males roughly equals the number of females (e.g., [A2.14, eTable 8] indicates equivalent person-time at risk for males and females 6–24).

[c] Column-3 rate, that for 18–29 year-old males after dose 2 of mRNA-1273 in Naveed et al.'s study population [A2.15], is taken here as an underestimate of the rate for 18–24-year-old males with homologous dosing—see the discussion above between Figures S5 and Figure S5m as well as that in Section S2.6 of this appendix, Column 4: [A2.15, Table S9]

[c] $\mathbf{270.1 \approx 253.6 \cdot \frac{222{,}008}{425600} + 265.1 \cdot \frac{94772}{425600} + 308.3 \cdot \frac{108820}{425600}}$

Assuming an 80% VAMP case-hospitalization rate, the weighted-average rate of 270.1 per million 2nd doses of mRNA-1273 yields a VAM/P hospitalization rate of approximately 216 per million second doses

[49] In the context of correspondence on a larger study by Naveed and et al. [A2.17], we requested from the corresponding author of Naveed 2022 data like that of Buchan et al.'s eTable 5 above but did not receive a response.

of mRNA-1273 among males 18–24/18–25. For reasons explained above, we take this as a rate for the 2nd dose of the homologous mRNA-1273 primary series without any restriction on dosing interval.

For Buchan et al.'s study [A2.2], the 2nd dose incidence-rate ratio for the homologous mRNA-1273 primary series with dosing interval < 56 days vs the homologous series with no dosing-interval restriction is 338.1/288.4 ≈ 1.17.[50] For dosing intervals < 35 days, the study [A2.18] suggests an incidence-rate ratio of 1.32 or higher—see Section S2.7 below. Multiplying the lower incidence-rate ratio 1.17 and the VAM/P rate of 270.1 per million second doses of mRNA-1273 for homologous series without a dosing-interval restriction suggests 316 (≈1.17 · 270.1) per million as a VAM/P rate for the 2nd dose of the homologous mRNA-1273 primary series with a restriction on dosing-interval of < 56 days (because the ratio 1.17 derives from a dosing-interval length restriction of < 56 days). Continuing to assume a case hospitalization rate of 80% for VAM/P, we obtain an estimate of 253 (≈0.8 · 316) per million as a VAM/P hospitalization rate for the 2nd dose of the homologous mRNA-1273 primary series, assuming dosing-interval length < 56 days. Figure 1d of [A2.18] suggests that VAM/P risk steadily increases as the dosing-interval length decreases. Thus, to make our rate of 253 per million more conservative, we'll take it as an estimate of the VAM/P hospitalization rate for the 2nd dose of the homologous mRNA-1273 primary series among males 18–25, assuming a dosing-interval-length of ≤ 42 days (6 weeks).

S2.3 VAM/P estimates for dose 1 of the mRNA-1273

Figure S5 of Kitano et al.'s meta-analysis also provides a 1st-dose assessment:

Figure S5 (Dose 1). Attributable risk of combined myocarditis or pericarditis following mRN-1273

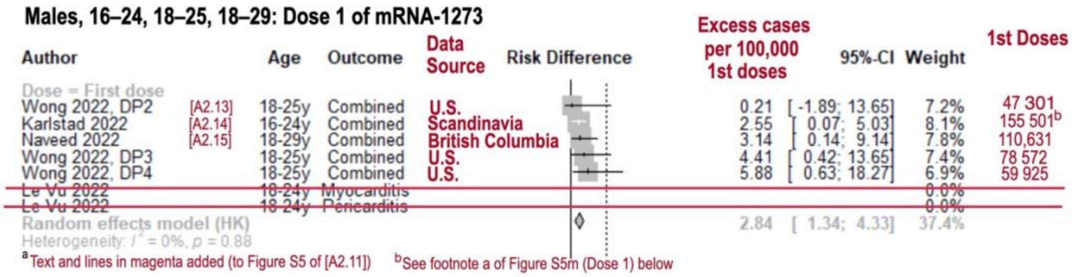

Once again Kitano at al. [A2.11] have disregarded data from data-partner DP1 of Wong 2022 [A2.13]. We again will use the meta-analysis of data from all four data partners DP1, 2, 3, and 4 from Table 2 of [A2.13], which reports an incidence rate of 0.62 per 100,000 person days of risk during a 7-day risk period after dose 1. Using a weighted-average of the three background rates provided in Supplemental Table 3 of [A2.13] of 0.12 cases per 100,000 person days of risk, we obtain an excess myo/pericarditis case rate of 0.50 cases per 100,000 person days of risk. Assuming each dose yields 7 days of risk, we obtain an incidence rate of 3.5 per 100,000 1st doses, which is consistent with Naveed et al.'s rate—especially so because the rate of Naveed 2022 is for 18–29-year-olds instead of 18–25 year-olds. Because we do not have separate assessments of dose-1 VAM/P risk for the ranges 18–24 and 25–29, we will not adjust Naveed et al.'s rate.

Figure S5m (Dose 1). Attributable risk of combined myocarditis or pericarditis following dose 1 of mRNA-1273

Author	Age	VAM/P rate per million 2nd doses	Number of 1st doses
Karlstad 2022[a]	16–24	25.5	155,501
Naveed 2022[b]	18–29	31.4	110,631
Wong 2022[c]	18-25	35.0	302,990

[50] Based on eTable 5 of [A2.2], the incidence rate for dose 2 of mRNA-1273 for homologous dosing among males 18–24 with dosing interval < 56 days is 388.1 (≈ $\mathbf{24/0.070975}$) per million.

Author	Age	VAM/P rate per million 2nd doses	Number of 1st doses
		Weighted Average of rates, rows 1–3[d]: 31.7	Total: 569,122

[a] Column-3 rate [A2.14, Table 3]; Column 4: Table 1 of [A2.14] reports 296,865 1st doses of mRNA-1273 received by males and females ages 16–24 while eTable 8 of [A2.14] provides a person-years at risk ratio of 3.2/2.9 ≈ $\mathbf{1.1}$ for males vs. females in the age range 16–24; thus, assuming that the number of males receiving dose 1 of mRNA-1273 is 1.1 times the corresponding number of females, we estimate that the number of males among the 296,865 dose-1 recipients is 155,501.
[b] Column-3 rate from Figure S5 (Dose 1) above, see also [A2.15, Table S6]; Column 4: [A2.15, Table S9]
[b] Column-3 rate [A2.13, Table 2] (see the discussion preceding this table); Column 4: [A213, Table 2]
[d] $31.7 \approx 25.5 \cdot \frac{155501}{569122} + 31.4 \cdot \frac{110631}{569122} + 35.0 \cdot \frac{302990}{569122}$

Applying an 80% case hospitalization rate, we might anticipate 0.8 · 31.7 ≈ 25 VAM/P hospitalizations per million 1st doses of mRNA-1273 among males 18–25. The 86% hospitalization rate used by FDA as a VAM/P case-hospitalization rate derives from an early VSD dataset [A2.19, p. 16] involving 1st and 2nd doses among 18–29 year-olds. Buchan et al. [A2.2, Table 1] report an *age-independent* case-hospitalization percentage for myo/pericarditis following dose 1 of any COVID vaccine of approximately 67% (≈ 60/90 x 100%) with 4 of the 60 hospitalized cases involving ICU admission.[51] We expect a higher hospitalization percentage among younger persons [A2.14, Section 2.3.2.2]. If we apply a 76% case-hospitalization rate (roughly the average of Buchan et al.'s 66% and the FDA's 86%) to the case rate of 31.7 per million, we obtain ≈ 24 VAM/P hospitalizations per million 1st doses of mRNA-1273.

S2.4 The VAM/P rate for the homologous mRNA-1273 primary series

Applying the pooled estimates of Figures S5m and S5m (Dose 1), we obtain for males 18–25 an estimate of 270.1 + 31.7 ≈ 302 *VAM/P cases per million homologous mRNA-1273 series completed with no restrictions on length of dosing interval and an estimate of 316 + 31.7 ≈348 VAM/P cases per million assuming a dosing interval of ≤ 6 weeks.*

Corresponding hospitalization rate estimates are as follows: 216 + 24 = 240 hospitalizations per million homologous mRNA-1273 series with no restrictions on length of dosing interval and of 253+24 = 277 hospitalizations per million homologous series, assuming a dosing interval ≤ 6 weeks.

Note that the interval of VAM/P hospitalization risk we have identified—from 240 to 277 per million full mRNA-1273 vaccinations—includes both our estimate of 268 VAM/P hospitalizations per million full mRNA-1273 vaccinations applied in the main exposition (and based on data available before 1/1/22) as well as the estimate 275.7 per million full vaccinations obtained in Section 1.4 of the main exposition from the VAM/P rate suggested by one of the FDA's data partners (assuming a 100% case hospitalization rate as did the FDA in its BNT162b2 assessment [A2.7]). Note also that we have made conservative choices throughout this appendix, e.g., not adjusting upward Naveed et al.'s VAM/P rate for 1st or 2nd doses of mRNA-1273 among males 18–29 [A2.15] to better approximate the rate among males 18–25 and using for males 18–24/25 an 80% VAM/P hospitalization rate for dose 2 and 76% for dose 1. Had we, e.g., used the FDA's VAM/P hospitalization rate of 86% for males 18–25, the corresponding hospitalization risk range would have been between 251 *hospitalizations per million homologous mRNA-1273 series with no restrictions on length of dosing interval and 289 hospitalizations per million homologous series, assuming a dosing interval ≤ 6 weeks*.

As we note in the next section, in February of 2022, the CDC [A2.20] updated dosing-interval guidance in the U.S.: "An 8-week interval may be optimal for some people ages 12 years and older, especially for males ages 12 to 39 years." We note that pooled data from British Columbia, Quebec, and Ontario [A2.17] suggests that even with a dosing interval ≥ 56 days, the level of VAM/P risk associated with dose 2 of mRNA-1273 remains concerning: 239.4 VAM/P cases per million 2nd doses among males 18–29 [A217, Table 2]. A conservative adjustment to estimate a corresponding rate for males 18–24 yields

[51] With the corresponding case-hospitalization-percentage for dose 2 being approximately 79.2% (≈164/207 x 100%) with 10 of the 164 hospitalizations involving ICU admission

approximately 278 VAM/P cases per million 2nd doses of mRNA-1273.[52] Assuming an 80% hospitalization rate for these 2nd-dose cases yields a rate of 222 VAM/P hospitalizations per million 2nd doses of mRNA-1273 among males 18–24/25. Table 3 of [A2.17], which provides data for the entire study population, indicates that with a dosing interval ≥ 56 days the VAM/P risk for dose 2 of the homologous mRNA-1273 primary series exceeds that of the series BNT162b2-mRNA-1273. Thus, the rate of 222 VAM/P hospitalizations per million 2nd doses of mRNA-1273 may approximate the rate corresponding to dose 2 of the homologous mRNA-1273 primary series for males 18–24.

S2.5 Heterologous primary-series dosing during calendar-year 2021: rare in the U.S., England, and France, common in Canada and Scandinavia

S2.5.1 United States. The initial CDC recommendations [A2.21, 12/20/20] for COVID vaccination included,

> The second dose should be administered as close to the recommended interval as possible. …. [T]hese mRNA COVID-19 vaccines are not interchangeable with each other or with other COVID-19 vaccine products. The safety and efficacy of a mixed-product series have not been evaluated. Both doses of the series should be completed with the same product.

The initial recommendations were modified by end of January 2021 [A2.22] as follows:

> The second dose should be administered as close to the recommended interval as possible. However, if it is not feasible to adhere to the recommended interval, the second dose of Pfizer-BioNTech and Moderna COVID-19 vaccines may be scheduled for administration up to 6 weeks (42 days) after the first dose. … Both doses of the series should be completed with the same product. … In exceptional situations in which the first-dose vaccine product cannot be determined or is no longer available, any available mRNA COVID-19 vaccine may be administered at a minimum interval of 28 days between doses to complete the mRNA COVID-19 vaccination series.

In February of 2022 the recommended dosing interval was extended to eight weeks [A2.20]: “An 8-week interval may be optimal for some people ages 12 years and older, especially for males ages 12 to 39 years”; but heterologous dosing was still not recommended for the primary series.

S2.5.2 England. Initial recommendations from Public Health England concerning COVID vaccination were similar to those in the United States concerning homologous dosing but provided more dosing-interval flexibility [A2.23, pp.7, 11]

> There is no evidence on the interchangeability of the mCOVID-19 vaccines although studies are underway. Therefore, every effort should be made to determine which vaccine the individual received and to complete with the same vaccine. For individuals who started the schedule and who attend for vaccination at a site where the same vaccine is not available, or if the first product received is unknown, it is reasonable to offer one dose of the locally available product to complete the schedule. … Operationally, it is recommended that the second dose of both vaccines should be routinely scheduled between four and 12 weeks after the first dose.

The recommendation that “every effort” should be made to complete the series with the same vaccine was left unchanged until mid-December 2021; however, by end of July 2021, the dosing-interval recommendation changed to the following [A2.24, p.10]: “JCVI [Joint Committee on Vaccination and Immunisation] is currently recommending a minimum interval of eight weeks between doses of all the available COVID-19 vaccines where a two-dose primary schedule is used.”

S2.5.3 Canada. Canada initially adopted the same policy as the U.S. and England on the interchangeability of primary series vaccines “NACI [National Advisory Committee on Immunization] recommends that the vaccine series be completed with the same COVID-19 vaccine product” [A2.25, 12/23/20], and recommended a schedule for Moderna’s vaccine as follows: minimum interval 21 days, authorized interval 28 days, alternative interval “None.” However, by early June, Canada had significantly altered its recommendations [A2.26, A2.27]:

[52] Apply Equation (2.6.1) below with 23.94 replacing 30.83.

> mRNA COVID-19 vaccine. NACI recommends that, if readily available, the same mRNA COVID-19 vaccine product should be offered for the subsequent dose in a vaccine series started with an mRNA COVID-19 vaccine. However, when the same mRNA COVID-19 vaccine product is not readily available, or is unknown, another mRNA COVID-19 vaccine product recommended for use in that age group can be considered interchangeable and should be offered to complete the vaccine series. … (Strong NACI Recommendation)
>
> AstraZeneca/COVISHIELD COVID-19 vaccine. NACI recommends that either AstraZeneca/COVISHIELD COVID-19 vaccine or an mRNA COVID-19 vaccine product may be offered for the subsequent dose in a vaccine series started with an AstraZeneca/COVISHIELD COVID-19 vaccine. … (Discretionary NACI Recommendation)
>
> Dosing schedule for Moderna's mRNA-1273: minimum interval 21 days, authorized interval 28 days, extended interval 16 weeks, with the extended interval given "in the context of limited COVID-19 vaccine supply and ongoing pandemic disease" and indicating that "jurisdictions should maximize the number of individuals benefiting from the first dose of vaccine by extending the second dose of COVID-19 vaccine up to four months.

Consistent with these altered recommendations, we see from Buchan et al.'s eTable 5 [A2.2], that of the primary series administered in Ontario to males 18–24 from 6/1/2021 through 9/4/2021 with 2nd dose mRNA-1273, 48.8% were heterologous (BNT162b2-mRNA-1273) and 52% were homologous (mRNA-1273/mRNA-1273); moreover, a strong majority of mRNA-1273 2nd doses were delivered with an extended dosing interval between 31 and 55 days (inclusive).[53] The Ontario vaccine uptake report [A2.9] is another source revealing that a significant number of heterologous series with 2nd dose being mRNA-1273 were administered during the period 12/14/20 – 9/4/2021: e.g., among 18–29 year-olds, Table 2 reports 444,609 1st doses of mRNA-1273 completed and 653,110 2nd doses. On the other hand, in the U.S. and England 1st and 2nd dose tallies are consistent with rare heterologous primary-series dosing. Among those being tracked by the CDC's VSD system through 10/9/2021, the number of 2nd doses received is about 96% of the number of 1st doses received for the mRNA-1273 vaccine and about 95% for the BNT162b2 vaccine [A2.8, p. 7]; among those in England belonging to the nearly 43 million-member study population of Patone et al. the number of 2nd doses received is about 89.7% of the number of 1st doses received for mRNA-1273 vaccine and about 85.6% for BNT162b2 [A2.4, Table 1].

S2.5.4. France. Data from two studies by Le Vu et al. [A2.12, A2.18] show that heterologous primary COVID-vaccine series having 2nd dose BNT162b2 or mRNA-1273 were rare in France through 30 November 2022.

The footnote to Table S1 of [A2.12] reads as follows:

> Study period from May 12, 2021, to October 31, 2021. a Among individuals that received the BNT162b2 vaccine as a second dose, 2.2% had received another vaccine for the first dose. b Among individuals receiving the mRNA-1273 vaccine as a second dose, 4.7% had received another vaccine for the first dose.

Table 1 of [A2.18] reports numbers of myocarditis cases occurring within 7 days of a 2nd dose of an mRNA vaccine during a study period of 12/27/2020 to 11/30/2022: 271 cases after BNT162b2 and 123 cases after mRNA-1273. Table S4 of [A2.18] reports numbers of myocarditis cases occurring within 7 days of a 2nd dose a homologous mRNA vaccine series (same study period): 263 cases after BNT162b2 and 119 cases after mRNA-1273, so that only 8 cases (≈ 2.95%) occurred after a heterologous primary series having BNT162b2 as dose 2 and only 4 cases after a heterologous series having mRNA-1273 as dose 2 (≈ 3.25%).

In terms of dosing interval, France's lengthened it to six weeks as of 4/14/2021 [A2.28] .

---

[53] Based on the row for 18–29 year-olds of Table 2 of the Ontario vaccine uptake report [A2.9], we see that during the period 6/1/22–9/4/2021 at most 255 (= 68 + 451 - 264) primary series administered to 18–29 year-olds had AstraZeneca's ChAdOx1as a first dose.

S2.5.5 Scandinavia. That the heterologous series BTN162b2-mRNA-1273 was not rare in Scandinavia is established by dosing data provided in Table 1 of Karlstad 2022 [A2.14], which reports the following numbers of those 16–24 (males and females) completing the given primary series:
BNT162b2-mRNA-1273: 128,495,
mRNA-1273-mRNA-1273: 189,545,
mRNA-1273-BNT162b2: 11,302,
BNT162b2-BNT162b2: 1,229,590.

In terms of dosing interval:

- In early January of 2021, Denmark health authorities recommended increasing the dosing interval to up to six weeks to allow more people to receive a first injection [A2.29].
- "On February 4, 2021, KRAR [Finland National Advisory Committee on Vaccines] recommended a 12-week interval between the corona vaccine doses. The longer interval was considered to enable a more rapid increase in the vaccine coverage in a serious epidemic situation, and coincided with a limited number of available vaccines, which that might result in better protection among the vaccinated. In May 2021, the recommendation for the interval between doses was shortened to 8-12 weeks for mRNA vaccines. In September 2021, THL [Finland Institute for Health and Welfare] again changed its recommendation on the intervals to the minimum of six weeks for both mRNA and adenovirus vaccines." [A2.30, p.2]
- In mid-March of 2021 Sweden extended the recommended dosing interval to six weeks for mRNA vaccines [A2.31]
- On 30 April 2021, Norway announced that "The interval between the first and second dose of the mRNA vaccines (Pfizer-BioNTech and Moderna) is being extended to up to 12 weeks for all age groups under 65 years following a recommendation from the Norwegian Institute of Public Health." [A2.32]

S2.6 Dose 2 mRNA-1273 VAM/P rate for 18–24 year-olds vs. 18–29 year-olds.

Naveed 2022 [A2.15] found a VAM/P case rate 30.83 per 100,000 2nd doses of mRNA-1273 among 18–29-year-old males. We argue here that this rate suggests a rate of at least 35.74 cases per 100,000 among 18–24-year-old males and that additional data provided in [A2.15] and other sources suggests a rate of at least 33.50 VAM/P cases per 100,000 2nd doses of the homologous mRNA-1273 primary series.

Le Vu et al. [A2.12] find an excess hospitalized myocarditis rate of approximately 16.95 per 100.000 2nd doses of mRNA-1273 among males 18–24, but for males 25–29, the corresponding rate is about 10.6 per 100,000 [A2.12, Figure 3]; moreover, for excess hospitalized pericarditis rates, Le Vu et al. find the rate to be approximately 3.34 per 100,000 2nd doses of mRNA-1273 among males 18–24, but for males 25–29, the corresponding rate is about 1.2 per 100,000 [A2.12, Figure 3]. Thus, the rates for the 25–29 range are, respectively, about 63% and 36% of those for the 18–24 range. Conservatively, we assume a uniform drop of only 33% from the range 18–24 to 25–29,[54] and also assume a fairly uniform uptake of dose 2 of mRNA-1273 for the range 18–29 so that, e.g., the number of 18-24 year-olds receiving the 2nd dose is 7/12's of the total number N for the range 18–29. If r is the excess postdose 2 myo/pericarditis rate for 18–24-year-old males, then we can express the number of excess myo/pericarditis cases for the 18–29 range as follows:

$$\frac{7}{12} \cdot N \cdot r + \frac{5}{12} \cdot N \cdot 0.67r = \frac{30.83}{100{,}000} \cdot N \qquad (2.6.1).$$

Solving for r yields r = 35.74 cases per 100,000 doses of mRNA-1273 as the rate for 18–24-year-old males.

We wish to adjust the estimated rate derived above from Naveed 2022 [A2.15] of 35.74 VAM/P cases per 100,000 doses of mRNA-1273 among 18–24-year-old males to make it more representative of a

---

[54] Consistent with our assertion that for males assuming a 33% drop in the VAM/P case rate from the age range 18–24 to 25–29 is conservative, Oster et al. in [A2.33, Table 2] found (based on passive-surveillance data from the CDC's VAERS system) a myocarditis incidence rate among males 18–24 of 56.31 per million 2nd doses of mRNA-1273 and among those 25–29 of 24.18 which represents a drop of approximately 57 %.

homologous-dosing rate. Evidence suggests that a strong majority of the primary series with 2nd dose mRNA-1273 contributing to this rate were homologous. Before presenting this evidence, we discuss the dependence of the VAM/P incidence rate for dose 2 of mRNA-1273 on the corresponding 1st dose type—mRNA-1273 or BNT162b2.

Buchan et al.'s data [A2.2, eTable 5] suggests that for 18–24-year-old males, dose 2 of mRNA-1273 carries more risk if the corresponding dose 1 is BNT162b2 instead of mRNA-1273, with the incidence-rate ratio for dose 2 of BNT162b2-mRNA-1273 vs. mRNA-1273-mRNA-1273 being 337.6/288.4 ≈1.17. However, note that if we restrict to those series having 2nd doses within 31 to 55 days of dose 1 (inclusive), the incidence rate for homologous dosing slightly exceeds that for heterologous dosing (331.4 per million 2nd doses vs. 318.9 per million). In a "multi-provincial" study, which we will call "Naveed 2024," Naveed et al. compare—for their entire study population—the myo/pericarditis incidence rate associated with the mRNA-1273-mRNA-1273 schedule to that of the BNT162b2-mRNA-1273 schedule [A2.17, Table 3]. According to Table 3 of [A2.17], the incidence rate for the homologous schedule exceeds that of the heterologous one only when the dosing interval is ≥ 56 days. However, because a majority of 2nd doses had a dosing interval beyond 56 days, the incidence rate for the homologous schedule mRNA-1273-mRNA-1273 exceeds that for the heterologous schedule BNT162b2-mRNA-1273 for the entire study population without any restriction on dosing interval. Here are the myo/pericarditis incidence-rate numbers:

mRNA-1273 - mRNA-1273: $\frac{193}{(25.18120 \cdot 100{,}000)} \approx 7.66$ per 100,000 2nd doses (entire study population),

BNT1612b2 - mRNA-1273: $\frac{91}{(16.52199 \cdot 100{,}000)} \approx 5.51$ per 100,000 2nd doses (entire study population),

where the numerators are myo/pericarditis cases reported in column 2 on the 2nd page of Table S4 of [A2.17], while the denominators are 1st-doses whose 2nd doses are mRNA-1273, which are reported, respectively, in the 3rd-from-last and 2nd-from-last rows of column 3 of Table S3 of [A2.17].

This finding—the overall homologous-case rate mRNA-1273-mRNA-1273 exceeds that for the heterologous schedule BNT162b2-mRNA-1273—differs from Buchan et al.'s corresponding finding for 18–24-year-old males [A2.2, eTable 5]. As we indicated in an earlier footnote, we requested from Naveed et al. data from their multi-provincial study Naveed 2024 [A2.17] for males 18–24 that would parallel the data in Buchan et al.'s eTable 5 [A2.2] but received no response.

If we wish to infer from Naveed 2022 [A2.15] a VAM/P case rate for 18–24-year-old males for the 2nd dose of the homologous mRNA-1273 primary series that is more likely to underestimate the rate than overestimate it, then we should assume that the heterologous schedule BNT1612b2-mRNA-1273 carries more VAM/P risk than the homologous mRNA-1273 schedule. We have noted that the incidence-rate ratio from Buchan et al.'s eTable 5, BNT1612b2-mRNA-1273 vs. mRNA-1273-mRNA-1273, is 1.17. Karlstad et al.'s corresponding ratio is 379.4/265.1 ≈ 1.43, which is a ratio of VAM/P rates from Table 3 of [A2.14]. To infer a homologous-schedule rate from the rate derived earlier of 35.74 VAM/P cases per 100,000 2nd doses of mRNA-1273 among 18–24-year-old males, we will assume that if r is the mRNA-1273 2nd dose VAM/P rate with homologous dosing (in cases per 100,000 2nd doses), then the corresponding VAM/P rate for BNT162b2-mRNA-1273 dosing is $1.3r$, where 1.3 is the average of incidence-rate ratios 1.17 and 1.43 discussed above.

To adjust the estimated rate derived above from Naveed 2022 [A2.15] of 35.74 VAM/P cases per 100,000 2nd doses of mRNA-1273 among 18–24-year-old males, to make it representative of a homologous-dosing rate, we need to estimate the percentage of primary series contributing to this rate that are homologous mRNA-1273-mRNA-1273. The rate 35.74 per 100,000 derives from 108,820 2nd doses of mRNA-1273 received by 18–29 year-old males in the study population of Naveed 2022 [A215., Table S9]. Table S9 indicates that 110,631 1st doses of mRNA-1273 were received by 18–29 year-olds in the study population; thus, in theory each of the 108,820 2nd doses could have had mRNA-1273 as the corresponding first dose, but this is surely not the case. Continuing with data from Table S9 of [A2.15], we note it is obvious there were significant numbers of non-homologous series with 2nd dose mRNA-1273 among males *30 and older*: for males 30+, 365,774 1st doses of mRNA-1273 were received but 423,764 2nd doses of mRNA-1273 were received. Similarly, in Ontario we know there were substantial numbers of heterologous series BNT162b2-mRNA-1273—as we noted earlier, Ontario vaccine-uptake data shows

through the final day of Buchan et al.'s study period 9/4/2021 that among 18–29 year-olds, 444,609 1st doses of mRNA-1273 had been administered while 653,110 2nd doses had been administered; in addition, 1,425,978 1st doses of BNT162b2 had been administered while only 962,678 2nd doses had been administered [A2.9]. In contrast, Table S9 of [A2.15] shows that for Naveed 2022, 274,238 1st doses of BNT162b2 and 256,767 2nd doses of BNT162b2 were received by 18–29-year-old males in the study population, and, as we have noted, 110,631 1st doses and 108,820 2nd doses of mRNA-1273 were received by 18–29 year-old males in the study population.

We now estimate the percentage of the 108,820 2nd doses of mRNA-1273 received by 18–29 year-old males in the study population of Naveed 2022 for which mRNA-1273 was also the corresponding dose 1.

According to Table S9 of [A2.15], a total of 274,238 + 110,631 = 384,869 1st doses of BNT162b2 or mRNA-1273 were administered to males 18–29 in Naveed 2022's study population from 12/15/2020 through 3/10/2022. According to the Government of Canada vaccine tracker [A2.34], 383,868 1st doses of a COVID vaccine were administered to males 18–29 in British Columbia through 10/13/2022. Comparing Table S9's tally of 1st doses 384,869 to that of the vaccine tracker 383,868, we assume that the vaccine tracker has slightly undercounted the number of 1st doses delivered. We have compared Naveed 2022's data to the vaccine tracker's to provide evidence that very few 1st doses of non mRNA vaccines (e.g., ChAdOx1) were administered to males 18–29 in Naveed 2022's study population, which also holds for Ontario based on its vaccine-uptake data [A2.9].

According to Table S9 of [A2.15], a total of 256,767 + 108,820 = 365,587 2nd doses of BNT162b2 or mRNA-1273 were administered to males 18–29 in Naveed 2022's study population from 12/15/2020 through 3/10/2022 while the Government of Canada vaccine tracker reports a slightly higher number 366,593. Subtracting the total number of mRNA-vaccine 2nd doses received by males 18–29 in Naveed 2022's study population from the corresponding 1st-dose total, we obtain 384,869 – 365,587 = 19,282, the number of 1st dose recipients who did not receive dose 2.

Because we seek to overestimate the number of heterologous series BNT162b2-mRNA-1273 received (which will result in an underestimate in the VAM/P risk of homologous-series dosing), we will assume that twice as many mRNA-1273 dose 1 recipients as BNT162b2 recipients chose not to take dose 2. If r is the fraction of BNT162b2 recipients who decline to take dose 2 (of any COVID vaccine) then $2r$ is the fraction of mRNA-1273 recipients who decline to take dose 2, and we have 274,238 r + 110,631 2 r = 19,282. Solving the preceding equation for r yields r = 0.0389 and thus the approximate number of BNT162b2 dose 1 recipients not taking dose 2 of any COVID vaccine is about 10,668 (and 8614 mRNA-1273 dose 1 recipients decided against taking dose 2).[55] Thus, assuming twice as many mRNA-1273 dose 1 recipients as BNT162b2 recipients chose not to take dose 2, we conclude that among males 18–29 in Naveed's 2022 study population 263,570 (≈274,238 – 10,668) recipients of dose 1 of BNT162b2 chose to take either BNT162b2 or mRNA-1273 as dose 2.

Buchan et al.'s eTable 5 [A2.2] shows that of the 244,672 18–24-year-old males receiving dose 2 of BNT162b2, 8,853 (≈3.6%) had mRNA-1273 as the corresponding 1st dose and the remainder (≈96.4%) had BNT162b2 as the 1st dose. Let's suppose that among the 256,767 18-29-year-old males in Naveed 2022's study population receiving dose 2 of BNT162b2, 18,487 (≈ 2·3.6%) had mRNA-1273 as the corresponding 1st dose and the remainder (≈92.8%) had BNT162b2 as the 1st dose. Assuming 92.8% of the 256,767 dose 2 BNT162b2 recipients had BNT162b2 as dose 1, we conclude that of the estimated 263,570 BNT162b2 dose 1 recipients who took dose 2 of an mRNA vaccine, 238,278 (≈0.928·256,767) received BNT162b2 as dose 2, which provides an estimate of 263,570-238,278 = 25,292 primary series consisting of BNT162b2 followed by mRNA-1273 received by 18–29 year-old males in Naveed 2022's study population. Thus, with our assumptions above we have an estimate that ≈23.2% of the 108,820 2nd doses of mRNA-1273 received by 18–29 year-old males followed BNT162b2 as dose 1 and 76.8% were 2nd doses of the homologous mRNA-1273 primary series.

Assume that (i) r is the 2nd-dose VAM/P rate (in cases per 100,000 2nd doses) associated with the homologous mRNA-1273 primary series among 18–24 year-old males, (ii) the VAM/P rate for BNT162b2-mRNA-1273 dosing is 1.3 r, where 1.3 is the average of 1.17 and 1.43, (iii) 23.2% of the

---

[55] Our assumption that twice as many mRNA-1273 dose 1 recipients as BNT162b2 recipients chose not to take dose 2 is contrary to the findings of the study [A2.35] (based on data from the U.S.) whose Table 2 reports that of 61,440,790 mRNA-1273 dose-1 recipients 9.08% missed dose 2 and of 94,406,661 BNT162b2 dose 1 recipients, 9.60% missed dose 2.

primary series having mRNA-1273 as dose 2 had BNT162b2 as dose 1 and the remaining 76.8% had mRNA_1273 as dose 1, and (iv) the overall VAM/P rate for is 35.74 per 100,000. We have

$$0.768\ r + 0.232 \cdot 1.3\ r = 35.74 \qquad (2.6.2)$$

so that r ≈ 33.50 cases per 100,000.[56]

We have completed our argument that Naveed 2022's VAM/P case rate 30.83 per 100,000 2nd doses of mRNA-1273 among 18–29-year-old males suggests a rate of at least 35.74 cases per 100,000 among 18–24-year-old males and that additional data provided in [A2.15] and other sources suggests a rate of at least 33.50 VAM/P cases per 100,000 2nd doses of the homologous mRNA-1273 primary series (among males 18–24).

S2.7 VAM/P incidence-rate variation for dose 2 of mRNA-1273 based on dosing interval, according to a study by Le Vu et al.

In [A2.18], which appeared in 2024, Le Vu et al. describe their matched, case-control study "Influence of mRNA Covid-19 vaccine dosing interval on the risk of myocarditis" having a study period 12/27/20–11/30/22 with a study population having ages 12 and up (stratified into 12–29, 30-49, 50+). "Through the same [study] period, 7911 cases of myocarditis were diagnosed in French hospitals. For each case, 10 control subjects of same sex, age and area of residency (Supplementary Table 1) were sampled and assigned the date of case diagnosis as index date."

Below is a copy of Le Vu et al.'s Supplementary Table 3 of [A2.18] providing an assessment of myocarditis risk for dose 2 of mRNA-1273 based on dosing interval. Unfortunately, the analysis is for males and females combined, ages 12-29 (rather than for males 18-24). We have highlighted adjusted odds ratios (aORs) for dosing intervals ≤ 35 days.

Supplementary Table 3. Association between myocarditis and exposure to mRNA vaccines within 7 days, by dosing interval and age category.

			Age											
			12-29				30-49				50+			
**Exposure**	**Dose**	**Interval**	**Case**	**Control**	**aOR**	**(95% CI)**	**Case**	**Control**	**aOR**	**(95% CI)**	**Case**	**Control**	**aOR**	**(95% CI)**
**Unexposed**	-	-	2774	30640	1.0	Reference	2080	21875	1.0	Reference	2068	20918	1.0	Reference
**BNT162b2**	**1**	-	40	286	2.0	(1.4-2.9)	25	156	2.5	(1.5-4.0)	10	137	1.4	(0.70-2.9)
	**2**	**<22**	93	73	22	(16-32)	21	28	11	(5.5-22)	0	23	-	-
		**22-28**	54	82	12	(8.1-18)	19	34	6.3	(3.1-13)	3	51	0.34	(0.046-2.4)
		**29-35**	17	36	8.3	(4.4-16)	0	14	-	-	2	6	4.6	(0.65-32)
		**>35**	39	122	4.5	(3.0-6.8)	15	70	3.3	(1.7-6.4)	8	60	1.3	(0.52-3.4)
		**All**	203	313	11	(8.6-13)	55	146	5.2	(3.5-7.8)	13	140	0.84	(0.40-1.8)
	**3**	**<153**	18	41	6.9	(3.7-13)	2	11	3.7	(0.76-18)	3	4	16	(2.3-110)
		**153-183**	36	85	6.0	(3.9-9.4)	10	39	3.1	(1.3-7.1)	3	37	2.4	(0.57-9.8)
		**184-213**	17	33	6.7	(3.4-13)	4	19	1.6	(0.42-6.3)	8	54	1.6	(0.61-4.3)
		**>213**	1	8	2.0	(0.24-16)	1	10	1.5	(0.19-12)	6	26	1.4	(0.39-4.8)
		**All**	72	167	6.2	(4.5-8.6)	17	79	2.5	(1.4-4.7)	20	121	2.0	(1.1-3.8)
**mRNA-1273**	**1**	-	3	31	1.3	(0.36-4.5)	7	32	3.6	(1.5-8.9)	3	21	1.0	(0.20-5.3)
	**2**	**<22**	19	4	73	(21-250)	16	7	30	(12-76)	1	3	1.8	(0.035-92)
		**22-28**	24	7	49	(20-120)	9	7	38	(12-120)	1	10	1.7	(0.17-16)
		**29-35**	8	4	30	(7.7-120)	5	4	16	(4.1-64)	1	1	1.9	(0.000045-81000)
		**>35**	27	13	25	(12-55)	11	14	9.5	(3.7-24)	1	9	2.5	(0.29-21)
		**All**	78	28	38	(23-64)	41	32	20	(12-34)	4	23	2.0	(0.46-8.7)
	**3**	**<153**	-	-	-	-	7	22	7.7	(3.0-20)	0	9	-	-
		**153-183**	-	-	-	-	8	44	2.5	(1.0-6.2)	6	19	6.9	(1.9-25)

[56] In order to produce a conservative estimate of the VAM/P rate for dose 2 of the homologous mRNA-1273 primary series among 18–24-year-old males, we have attempted to overestimate the percentage non-homologous primary series having 2nd dose mRNA-1273 received by males 18–29 in Naveed 2022's study population (i) by assuming recipients of dose 1 mRNA-1273 were twice as likely not to take dose 2 of either mRNA vaccine and (ii) by assuming 92.8% of the 2nd doses of BNT162b2 received were part of the homologous BNT162b2-BNT162b2 series. Naveed 2024 [A2.17], which analyzed VAM/P data from British Columbia, Quebec, and Ontario found that over their entire study population 95% of recipients of dose 2 of BNT161b2 had BNT162n2 as the corresponding dose 1 (of 13,106,508 2nd doses received, 12,456,645, 95.0%, also had BNT162b2 as dose 1 [A2.17, Table S3]). Note that had we assumed 95%, instead of 92.8% as the homologous-dosing percentage among recipients of BNT162b2 dose 2, our estimated percentage of heterologous series BNT162b2-mRNA-1273 would have dropped. On the other hand, Table S3 of [A2.17] indicates that of the 4,170,319 recipients of 2nd doses of mRNA-1273 in Naveed 2024's study population (all ages both sexes) who received an mRNA vaccine as dose 1, 1,652,199 (≈39.0%) had BNT162b2 as dose 1; so that the dosing percentage for the heterologous series BNT162b2-mRNA-1273 in Naveed 2024's study population appears to be considerably higher than that of Naveed 2022's percentage for 18–29 year-old males. Factors that may account for this are higher heterologous dosing percentages in Ontario vs. British Columbia suggested by Buchan et al.'s eTable 5 of [A2.2] and higher heterologous dosing among older age ranges. Note that if we transform equation (2.6.2) assuming 61% homologous dosing and 39% heterologous, we obtain $0.61\ r + 0.39 \cdot 1.3\ r = 35.74$, so that $r \approx 32.00$ cases per 100,000, which still exceeds the rate we have applied in Table S5m.

In an earlier study, Le Vu et al. analyzed risk for males and females separately with a finer age stratification [A2.12, Table S2]:

Table S2. Association between myocarditis and exposure to mRNA vaccines within 7 days, according to sex and age group

			Male				Female			
Age	Exposure	Dose	Cases	Controls	OR (95% CI)[a]	aOR (95% CI)[b]	Cases	Controls	OR (95% CI)[a]	aOR (95% CI)[b]
12-17	Unexposed		92	1,119	Reference	Reference	22	248	Reference	Reference
	BNT162b2	1	3	49	0.71 (0.21-2.3)	0.86 (0.26-2.9)	1	2	6.1 (0.54-70)	8.5 (0.69-110)
		2	30	37	14 (7.4-26)	18 (9-35)	4	13	4.7 (1.1-20)	7.1 (1.5-33)
	mRNA-1273	1	0	1	-	-	0	0	-	-
		2	2	0	-	-	0	0	-	-
18-24	Unexposed		290	3,940	Reference	Reference	67	863	Reference	Reference
	BNT162b2	1	16	113	2.2 (1.3-3.8)	2.1 (1.2-3.7)	2	29	0.9 (0.2-4)	0.9 (0.2-4.1)
		2	88	130	12 (8.5-17)	13 (9.2-19)	12	25	7.8 (3.5-18)	9.6 (4.3-22)
	mRNA-1273	1	2	12	2.6 (0.56-13)	2.6 (0.56-13)	1	2	6.5 (0.57-76)	15 (1.1-200)
		2	38	12	46 (23-91)	44 (22-88)	11	4	33 (10-100)	41 (12-140)
25-29	Unexposed		138	1,743	Reference	Reference	30	348	Reference	Reference
	BNT162b2	1	8	32	3.3 (1.5-7.4)	4 (1.7-9.3)	0	12	-	-
		2	28	61	6.3 (3.8-10)	7.1 (4.2-12)	4	7	7.5 (1.8-31)	10 (2.1-47)
	mRNA-1273	1	1	5	2.4 (0.27-20)	3.4 (0.37-31)	0	2	-	-
		2	17	13	20 (9-45)	19 (8.3-43)	2	1	20 (1.8-220)	23 (2.1-270)

We return our focus to Supplementary Table 3 of [A2.18]. Because the numbers of controls corresponding to a given dosing gap should roughly correlate with the number of doses administered to the general population having the given dosing gap, we'll compute an odds ratio for 2nd doses with dosing gap ≤ 35 days as the following weighted average:

$$\frac{4}{15}\cdot 73+\frac{7}{15}\cdot 49+\frac{4}{15}\cdot 30\approx 50.33.$$

Comparing the preceding aOR to that for "All" dosing-intervals, 38, reported in [A2.18, Table S3], we obtain an aOR ratio 50.33/38 ≈1.32, and take this as a rough estimate of the ratio of the incidence rate of myocarditis for dose 2 of mRNA-1273 when administered ≤ 35 days after dose 1 vs. the rate with no dosing-interval restriction.

References:

[A2.1] Buchan, S.A., Seo, C.Y., Johnson, C., et al. Epidemiology of myocarditis and pericarditis following mRNA vaccines in Ontario, Canada: by vaccine product, schedule and interval. Preprint. medRxiv 2021.12.02.21267156; doi: 10.1101/2021.12.02.21267156.

[A2.2] Buchan, S.A., Seo, C.Y., Johnson, C., Alley, S., Kwong, J.C., Nasreen, S., et al. Epidemiology of Myocarditis and Pericarditis Following mRNA Vaccination by Vaccine Product, Schedule, and Interdose Interval Among Adolescents and Adults in Ontario, Canada. JAMA Netw. Open 2022;5:e2218505. doi: 10.1001/jamanetworkopen.2022.18505

[A2.3] Patone, M., Mei X.W., Handunnetthi, L., Dixon, S., Zaccardi, F., Shankar-Hari, M., et al. Risk of Myocarditis After Sequential Doses of COVID-19 Vaccine and SARS-CoV-2 Infection by Age and Sex. medRxiv [Preprint] 2021. Available online: https://www.medrxiv.org/content/10.1101/2021.12.23.21268276v1

[A2.4] Patone, M., Mei X.W., Handunnetthi, L., Dixon, S., Zaccardi, F., Shankar-Hari, M., et al. Risk of Myocarditis After Sequential Doses of COVID-19 Vaccine and SARS-CoV-2 Infection by Age and Sex. Circ. 2022;146:743–754. doi: 10.1161/CIRCULATIONAHA.122.059970

[A2.5] Sharff, K.A., Dancoes, D.D., Longueil, J.L., Johnson, E.S., Lewis, P.F. Risk of myopericarditis following COVID-19 mRNA vaccination in a large integrated health system: A comparison of completeness and timeliness of two methods. medRxiv [Preprint] 2021. Available online: https://www.medrxiv.org/content/10.1101/2021.12.21.21268209v1

[A2.6] Sharff, K.A., Dancoes, D.D., Longueil, J.L., Johnson, E.S., Lewis, P.F. Risk of myopericarditis following COVID-19 mRNA vaccination in a large integrated health system: A comparison of completeness and timeliness of two methods. Pharmacoepidemiolgy Drug Saf. 2022;31: 921–925. doi: 10.1002/pds.5439

[A2.7] Funk, P.R., Yogurtcu, O.N., Forshee, R.A., Anderson, S.A., Marks, P.W., Yang, H. Benefit-risk assessment of COVID-19 vaccine, mRNA (Comirnaty) for age 16–29 years. Vaccine 2022;40: 2781–2789. doi: 10.1016/j.vaccine.2022.03.030

[A2.8] Klein, N. Myocarditis analyses in the vaccine safety datalink: rapid cycle analyses and "Head-to-Head" product comparisons. CDC ACIP meeting on COVID-19 vaccines, October 2021. Available online https://www.cdc.gov/vaccines/acip/meetings/downloads/slides-2021-10-20-21/08-COVID-Klein-508.pdf

[A2.9] COVID-19 Vaccine Uptake and Program Impact in Ontario: December 14, 2020 to September 4, 2021. Public Health Ontario. Surveillance Report. https://www.publichealthontario.ca/en/Data-and-Analysis/Infectious-Disease/COVID-19-Data-Surveillance/Archives/Vaccine-Uptake
[A2.10] Interim Clinical Considerations for Use of mRNA COVID-19 Vaccines Currently Authorized in the United States. United States Centers for Disease Control and Prevention. Page last reviewed: January 21, 2021. https://web.archive.org/web/20210131024205/https://www.cdc.gov/vaccines/covid-19/info-by-product/clinical-considerations.html
[A2.11] Kitano, T., Salmon, D.A., Dudley, M.Z., Saldanha, I.J., Thompson, D.A., Engineer, L. Age- and sex-stratified risks of myocarditis and pericarditis attributable to COVID-19 vaccination: a systematic review and meta-analysis. Epidemiol Rev. 2025;47:1–11. doi: 10.1093/epirev/mxae007
[A2.12] Le Vu, S., Bertrand, M., Jabagi, M.J., Botton, J., Drouin, J., Baricault, B., et al. Age and sex-specific risks of myocarditis and pericarditis following Covid-19 messenger RNA vaccines. Nat. Commun. 2022;13:3633. doi: 10.1038/s41467-022-31401-5
[A2.13] Wong, H.L., Hu, M., Zhou C.K., Lloyd, P.C., Amend, K.L., Beachler, D.C., et al. Risk of myocarditis and pericarditis after the COVID-19 mRNA vaccination in the USA: a cohort study in claims databases. Lancet. 2022;399:2191–2199. doi: 10.1016/S0140-6736(22)00791-7
[A2.14] Karlstad, Ø., Hovi, P., Husby, A., H\"ark\"anenet, T., Selmer, R.M., Pihlstr\"om, N., et al. Vaccination and Myocarditis in a Nordic Cohort Study of 23 Million Residents. JAMA Cardiol. 2022;7:600–612. doi: 10.1001/jamacardio.2022.0583
[A2.15] Naveed, Z., Li, J., Spencer, M., Wilton, J., Naus, M., García, H.A.V., Otterstatter, M., Janjua, N.Z. Observed versus expected rates of myocarditis after SARS-CoV-2 vaccination: a population-based cohort study. CMAJ 2022;194:E1529--E1536. doi: 10.1503/cmaj.220676
[A2.16] Yogurtcu, O.N., Funk, P.R., Forshee, R.A., Anderson, S.A., Marks, P.W., Yang, H. Benefit-risk assessment of COVID-19 vaccine mRNA (mRNA-1273) for males age 18--64 years. Vaccine 2023; 14: 100325. doi: https://doi.org/10.1016/j.jvacx.2023.100325
[A2.17] Naveed, Z., Chu, C., Tadrous, M., Veroniki, A.A., Li, J., Rouleau, I., et al. A multiprovincial retrospective analysis of the incidence of myocarditis or pericarditis after mRNA vaccination compared to the incidence after SARS-CoV-2 infection. Heliyon. 2024;10:e26551. doi: 10.1016/j.heliyon.2024.e26551
[A2,18] Le Vu, S., Bertrand, M., Semenzato, L., Jabagi, M.J., Botton, J., Drouin, J., et al. Influence of mRNA Covid-19 vaccine dosing interval on the risk of myocarditis. Nat. Commun. 2024;15:7745. doi: 10.1038/s41467-024-52038-6
[A2.19] Klein, N. Rapid Cycle Analysis to Monitor the Safety of COVID-19 Vaccines in Near Real-Time within the Vaccine Safety Datalink: Myocarditis and Anaphylaxis. CDC ACIP meeting August 2021. Available online: https://www.cdc.gov/vaccines/acip/meetings/downloads/slides-2021-08-30/04-COVID-Klein-508.pdf
[A2.20]. Interim Clinical Considerations for Use of mRNA COVID-19 Vaccines Currently Authorized in the United States. United States Centers for Disease Control and Prevention. Page last reviewed: February 22, 2022. https://web.archive.org/web/20220228144109/https://www.cdc.gov/vaccines/covid-19/clinical-considerations/covid-19-vaccines-us.html
[A2.21] Interim Clinical Considerations for Use of mRNA COVID-19 Vaccines Currently Authorized in the United States. United States Centers for Disease Control and Prevention. Page last reviewed: December 20, 2020. https://web.archive.org/web/20201230005657/https://www.cdc.gov/vaccines/covid-19/info-by-product/clinical-considerations.html
[A2.22] Interim Clinical Considerations for Use of mRNA COVID-19 Vaccines Currently Authorized in the United States. United States Centers for Disease Control and Prevention. Page last reviewed: January 21, 2021. https://web.archive.org/web/20210131024205/https://www.cdc.gov/vaccines/covid-19/info-by-product/clinical-considerations.html
[A2.23] COVID-19: The Green Book, Chapter 14a: Coronavirus (COVID-19) vaccination information for public health professionals. GOV.UK. Last updated 11 December 2020. https://web.archive.org/web/20201218122201/https://www.gov.uk/government/publications/covid-19-the-green-book-chapter-14a
[A2.24] COVID-19: The Green Book, Chapter 14a: Coronavirus (COVID-19) vaccination information for public health professionals. GOV.UK. Last updated 30 July 2021. https://www.gov.uk/government/publications/covid-19-the-green-book-chapter-14a
[A2.25]. Recommendations on the use of COVID-19 vaccines. Government of Canada. Publication date: December 23, 2020. https://web.archive.org/web/20210101233331/https://www.canada.ca/en/public-health/services/immunization/national-advisory-committee-on-immunization-naci/recommendations-use-covid-19-vaccines.html

[A2.26] Archived 12: NACI rapid response: Interchangeability of authorized COVID-19 vaccines [2021-06-01]. Government of Canada.6/1/21. https://web.archive.org/web/20210629224302/https://www.canada.ca/en/public-health/services/immunization/national-advisory-committee-on-immunization-naci/recommendations-use-covid-19-vaccines/rapid-response-interchangeability.html
[A2.27]. Recommendations on the use of COVID-19 vaccines. Government of Canada. Publication date: May 28, 2021. https://web.archive.org/web/20210630005938/https://www.canada.ca/en/public-health/services/immunization/national-advisory-committee-on-immunization-naci/recommendations-use-covid-19-vaccines.html
[A2.28] France extends gap between mRNA vaccine shots to ramp-up rollout. Reuters. 10 April 2021. https://www.reuters.com/business/healthcare-pharmaceuticals/france-extend-gap-between-mrna-vaccine-shots-minister-says-2021-04-10/
[A2.29] Denmark to expand COVID-19 vaccination program by delaying second dose. Medical X Press. 4 January 2021. Available online: https://medicalxpress.com/news/2021-01-denmark-covid-vaccination-dose.html#:~:text=Denmark%20to%20expand%20COVID%2D19,on%20documentation%20submitted%20by%20laboratories
[A2.30] Tiirinki H, Viita-Aho M, Tynkkynen LK, Sovala M, Jormanainen V, Keskimäki I. COVID-19 in Finland: Vaccination strategy as part of the wider governing of the pandemic. Health Policy Technol. 2022;11:100631. doi: 10.1016/j.hlpt.2022.100631
[A2.31] Sweden extends gap between vaccine doses to six weeks to speed up rollout. The Local. 19 March 2021. Available online:
https://www.thelocal.se/20210319/sweden-moves-to-stretch-gap-between-vaccine-doses-to-six-weeks#:~:text=Sweden's%20public%20health%20agency%20has%20recommended%20that,number%20of%20vulnerable%20people%20protected%20against%20coronavirus
[A2.32] Interval extended between the first and second dose of the vaccine. News story. Ministry of Health and Care Services. Government of Norway. 30 April 2021. Available online: https://www.regjeringen.no/en/historical-archive/solbergs-government/Ministries/hod/News/2021ny/interval-extended-between-the-first-and-second-dose-of-the-vaccine/id2846787/
[A2.33] Oster, M.E., Shay D.K., Su ,J.R., Gee, J., Creech, C.B., Broder, K.R., et al. Myocarditis Cases Reported After mRNA-Based COVID-19 Vaccination in the US From December 2020 to August 2021. JAMA 2022;327:331–340. doi: 10.1001/jama.2021.24110
[A2.34] COVID-19 vaccination in Canada. Vaccination coverage by age, sex, and province or territory. Spreadsheet for Figure 5 for report week, March 13, 2022. Government of Canada. Available online: https://web.archive.org/web/20220323053623/https://health-infobase.canada.ca/covid-19/vaccination-coverage/
[A2.35] Meng, L., Murthy, N.C., Murthy, B.P., Zell, E., Saelee, R., Irving, M., et al. Factors Associated with Delayed or Missed Second-Dose mRNA COVID-19 Vaccination among Persons >12 Years of Age, United States. Emerg. Infect. Dis. 2022;28:1633–1641. doi: 10.3201/eid2808.220557

Appendix S3: Subpopulation Analyses

S3.1 Subpopulation analyses based on prior-infection status.

As noted in the main exposition, for the subpopulation of 18–25-year-old unvaccinated males having a COVID-infection prior to the evaluation period 1/1/22 -- 5/31/22, we can use our model to compute the expected number of COVID hospitalizations in various scenarios simply by setting $F_{pi} = 1$ and leaving all other variables unchanged. For example, with $F_{pi} = 1$ in Scenario A, our model predicts $U \approx 161.70$ hospitalizations per million and $V \approx 72.24$, yielding a hospitalizations-prevented benefit of 89.46 per million vaccinated, far below VAM/P hospitalization risk (≈ 268 per million vaccinated).

The analysis of the preceding paragraph may be reinterpreted in terms of evaluation-period hospitalization risk for a typical unvaccinated 18–25-year-old male, COVID-infected prior to the evaluation period. For ease of exposition, we will call this male Bob.[57] If Bob chooses not to vaccinate, our model estimates the probability that Bob will be hospitalized for COVID during the evaluation period to be 161.70 in 1 million (assuming $I_r = 1$). Bob's total hospitalization risk associated with the choice to vaccinate is essentially the sum of his probability of VAM/P hospitalization and the probability of his

[57] If we were also considering COVID-hospitalization risk for a typical 18–25-year-old female, we would call her Alice, choosing "Alice" and "Bob" because these names are customarily used for "person A" and "person B" in the field of cryptography.

having a breakthrough-case COVID hospitalization during the evaluation period (assuming postvaccination VAM/P hospitalization is independent of postvaccination COVID-hospitalization).[58]

While there is some evidence that VAM/P risk increases with prior infection, we assume only that it does not decrease. Suppose Bob decides to take dose 1 and at the same time decides to take dose 2 if VAM/P does not occur after dose 1; then, if r_1, r_2 are, respectively, the VAM/P-incidence rates for doses 1 and 2 for males 18–25, then the probability Bob will experience VAM/P is well approximated by

$$r_1 + (1 - r_1)r_2.$$

Because r_1 is quite small, the preceding probability is essentially $r_1 + r_2$, which explains why, even on the individual level, the FDA should consider the sum of risks, postdose 1 and postdose 2 in assessing the VAM/P risk of full vaccination (as the agency did in its population-level analyses of BNT162b2 [A3.1, Section 2.3.1] and of mRNA-1273 in its BLA-Memo [A3.2, Section 4.7]). Therefore 268/1,000,000 approximates the probability that Bob experiences hospitalization owing to VAM/P during the course of mRNA-1273 vaccination. The probability that he experiences COVID hospitalization owing to a breakthrough infection is 72.24 in 1 million, according to our model. Thus, his total hospitalization risk from vaccination is approximately 340.24 in 1 million. This is under the Scenario-A assumption that $I_r = 1$, which we interpret to mean that evaluation-period infection is certain for an infection-naïve male 18–25, and Bob's probability of infection is $I_r(1 - E_{pi}) = 0.55$. Table 7 of the main exposition shows how Bob's hospitalization risk varies as his risk of infection varies.

S3.2 Subpopulation analyses based on prior-infection status and BMI.

We illustrate how comorbidities may impact a benefit-risk assessment, focusing on obesity. As we have noted, "Having obesity [BMI ≥ 30] may triple the risk of hospitalization due to a COVID-19 infection" [A3.3]. This is consistent with a finding that hospitalization risk for the obese is 3.2 times that for the non-obese for an adolescent study population (ages 12–17) [A3.4, Table IV]. Gao et al. [A3.5] provide additional support for this CDC estimate based on a cohort study of 6,910,695 residents of England (data from the period 1/24/20 – 4/30/20). Gao et al. found that for those 20–39 years old COVID hospitalization risk is minimized when BMI $\approx$ 23 and that for 23 $\leq$ BMI $\leq$ 44 risk is multiplied by a factor of approximately 1.09 (1.08, 1.10) for each unit increase in BMI. Gao et al. "found no difference in effect estimates by sex" [A3.5, Figure 2]. Applying Gao's model (see Section S3.3 below), we obtain the following:

Table S1.7: Estimated COVID hospitalization hazard ratio (HR) for 20–39 year-olds relative to those with BMI $\approx$ 23 based on a model by Gao et al. [A3.5]

BMI Range	COVID Hospitalization HR
23–24	ref.
25–29	1.49
30–34	2.29
35–39	3.52
40–44	5.41

Assuming that for 18–25-year-old males obesity triples COVID-hospitalization risk and that 20.7% of this population is obese [A3.6], we find not being obese drops baseline COVID hospitalization risk $H_r = 0.0014$ to approximately $0.71 \cdot H_r$ (see Section S3.3 below). We assume this same factor determines the drop in hospitalization risk for unvaccinated males 18–25 with prior infection (IHR $= 0.71 \cdot H_r \cdot (1 - E_{pi})(1 - HRR_{pi})$).

[58] Technically speaking, because there is a small chance—on the order of 0.02 in 1 million---that Bob might experience both a VAM/P hospitalization and a breakthrough-COVID hospitalization, Bob's total hospitalization risk from vaccination is the sum of his VAM/P-related risk and his COVID-related risk minus the product of these two risks (with the product being the small chance that Bob experiences both a VAM/P hospitalization and a COVID hospitalization by our assumption of independence of these events).

We assume that not being obese for an otherwise typical *vaccinated* 18–25-year-old male decreases IHR by a greater amount: IHR $= 0.36 \cdot H_r(1 - E_v)(1 - HRR_v)$ based on case-hospitalization rates computed from the raw data of Supplementary Table A2 of [A3.7].

We provide in Table S1.8 below (which reproduces Table 9 of the main exposition) a COVID hospitalization benefit-risk analysis derived from Gao et al.'s BMI-based model [A3.5] described above. We assume that the BMI range 23–24 for 18–25-year-old males provides minimum COVID-hospitalization risk, which we estimate by multiplying the hospitalization risks appearing in column 2 of Tables 7 and 8 of the main exposition by the factor 0.71 and those in column 3 by the factor of 0.36. For a BMI-based hazard ratio HR from Table S1.7 above, we compute the corresponding effect on the hospitalization risks in column 2 of Tables 7 and 8 of the main exposition via the factor $HR \cdot 0.71$ and for those in column 3 via the factor $HR \cdot 0.36$.

Table S1.8: Hospitalization-risk ratios $\left(\frac{\text{risk if vaccinated (VAM/P \& breakthrough)}}{\text{risk if unvaccinated}}\right)$ based on BMI for males 18–25. Ratios displayed as pairs: "with prior-infection protection, without prior-infection projection."

Probability of Infection I_r*	BMI 23–24	BMI 25–29	BMI 30–34	BMI 35–39	BMI 40–44
1	2.56, 0.39	1.79, 0.30	1.25, 0.23	0.89, 0.19	0.66, 0.17
0.5	4.90, 0.66	3.36, 0.48 **	2.26, 0.35	1.55, 0.27	1.09, 0.22
0.25	9.56 ,1.20	6.49 , 0.84	4.30, 0.59	2.85, 0.42	1.85, 0.32

*With prior-infection protection, $I_r(1 - E_{pi})$ is the probability of infection over the evaluation period.

**Sample computation: $\frac{268+36.12\cdot 1.49\cdot 0.36}{80.85\cdot 1.49\cdot 0.71}, \frac{268+161.7\cdot 1.49\cdot 0.36}{700\cdot 1.49\cdot 0.71} \approx 3.49, 0.48$

S3.3 Gao's Model.

Recall that Gao et al [A3.5] found that for those 20–39 years old the minimum COVID hospitalization risk was associated with BMI ≈ 23 and that for the range 23 ≤ BMI ≤ 44 risk is multiplied by factor of approximately 1.09 (1.08, 1.10) for each unit increase in BMI. In other words, Gao and al. found that for those 20–39 years old, with BMI x ≥ 23, hospitalization hazard ratio HR relative to those with BMI ≈ 23 is well modeled by

$$f(x) = 1.09^{x-23}.$$

For the data of Table S1.7 above, we approximated the COVID Hospitalization HR for the given ranges, staring with BMI 25–29, as the average value of the function $f(x) = 1.09^{x-23}$ over, respectively, the intervals 25 ≤ BMI ≤ 30, 30 ≤ BMI ≤ 35, 35 ≤ BMI ≤ 40, 40 ≤ BMI ≤ 45. For example, for the range 25–29, we obtain,
$$

$$\frac{1}{30-25}\int_{25}^{30} 1.09^{x-23}\, dx \approx 1.49.$$

.

We now derive an approximate COVID infection-hospitalization rate for non-obese 18–25-year-old males, based on the assumption that for males in this age group obesity triples COVID-hospitalization risk. Let r denote the approximate COVID infection-hospitalization rate for unvaccinated 18–25-year-old males with no prior-infection *who are not obese*, and recall our estimated baseline IHR for typical unvaccinated infection naïve males 18–25 is 0.0014. Assuming that for 18–25-year-old males obesity triples COVID-hospitalization risk and that 20.7% of this population is obese [A3.6], we find that $0.793\, r + 3r \cdot 0.207 = 0.0014$, so that $r \approx 0.00099$. Hence, not being obese multiplies baseline COVID hospitalization risk $H_r = 0.0014$ by approximately 0.71.

References:

[A3.1] Yogurtcu, O.N., Funk, P.R., Forshee, R.A., Anderson, S.A., Marks, P.W., Yang, H. Benefit-risk assessment of COVID-19 vaccine mRNA (mRNA-1273) for males age 18–64 years. Vaccine 2023; 14: 100325. doi: https://doi.org/10.1016/j.jvacx.2023.100325

[A3.1] Funk, P.R., Yogurtcu, O.N., Forshee, R.A., Anderson, S.A., Marks, P.W., Yang, H. Benefit-risk assessment of COVID-19 vaccine, mRNA (Comirnaty) for age 16–29 years. Vaccine 2022; 40: 2781–2789. doi: 10.1016/j.vaccine.2022.03.030
[A3.2] Zhang, R., Goswami, J. BLA Clinical Review Memorandum. FDA Office of Vaccines Research and Review. Review Completion Date 28 January 2022. Available online: https://www.fda.gov/media/156342/download
[A3.3] Obesity, Race/Ethnicity, and COVID-19. United States Centers for Disease Control and Prevention. January 20, 2022 [cited 2024 Apr 26]. Available online: https://web.archive.org/web/20220120135636/https://www.cdc.gov/obesity/data/obesity-and-covid-19.html
[A3.4] Campbell, J.I., Dubois, M.M., Savage, T.J., Hood-Pishchany, M.I., Sharma, T.S., Petty, C.R., et al. Pediatric COVID-19 US Registry. Comorbidities Associated with Hospitalization and Progression Among Adolescents with Symptomatic Coronavirus Disease 2019. J. Pediatr. 2022;245:102–10. doi: 10.1016/j.jpeds.2022.02.048
[A3.5] Gao, M., Piernas, C., Astbury, N.M., Hippisley-Cox, J., O'Rahilly, S., Aveyard, P., Jebb, S.A. Associations between body-mass index and COVID-19 severity in 6.9 million people in England: a prospective, community-based, cohort study. Lancet Diabetes Endocrinol. 2021;9:350–9. doi: 10.1016/S2213-8587(21)00089-9
[A3.6] Adult Obesity Prevalence Maps. United States Centers for Disease Control and Prevention. Updated September 27, 2022 [cited 2024 Apr 26]. Available online: https://web.archive.org/web/20230420140356/https://www.cdc.gov/obesity/data/prevalence-maps.html
[A3.7] Simard, M., Boiteau, V., FortinÉ, Jean, S., Rochette, L., Trépanier, P.L., et al. Impact of chronic comorbidities on hospitalization, intensive care unit admission and death among adult vaccinated and unvaccinated COVID-19 confirmed cases during the Omicron wave. J Multimorb Comorb. 2023;13:26335565231169567. doi: 10.1177/26335565231169567

Appendix S4: Approximating the level of prior-infection protection for a representative group G of 1 million unvaccinated 18–25-year-old persons in the United States formed at the beginning of the evaluation period 1 January 2022

We begin by estimating numbers of infections that have occurred in the group G, starting with those closest to the evaluation period, which are the infections among members of G yielding the most effective evaluation-period protection from reinfection. We rely on the same CDC data [32] used in Section S3 to obtain F_{pi}. Specifically, we rely on data from Tables 1 and 2 of [32]:

From Table 1

Event/Variant emergence/ Predominance period§	Unvaccinated persons	
	Total no.	Average weekly incidence*
**COVID-19 cases**		
Pre-Delta (April–May 2021)	**1,006,686**	163.8
Delta emergence (June 2021)	**196,988**	64.0
Delta predominance (July–November 2021)	**4,546,682**	460.1
Omicron emergence (December 2021)	**1,061,684**	725.6
**Total**	**6,812,040**	—

* Events per 100,000 population.

From Table 2

Event/Time/ Characteristic	Unvaccinated	
	Total no.	Average weekly incidence*
**COVID-19 cases**		
**October–November**		
Overall (age-standardized)	**1,108,298**	347.8
**Age group, yrs**		
18–49	**760,042**	330.3
50–64	**225,290**	355.3
≥65	**122,966**	403.6
**December**		
Overall (age-standardized)	**1,061,684**	725.6
**Age group, yrs**		
18–49	**781,969**	745.6
50–64	**189,789**	680.8
≥65	**89,926**	704.9

Observe from Table 2 (above right) that the average weekly incidence rates per 100,000 population for the age group 18–49 for October-November as well as December are similar to the overall rates. For October–December we will assume that rates for those 18–49 well approximate those for 18–25 year-olds, and, for April–September, we will assume overall rates well approximate those for 18–25 year-olds. Because our group G of one million unvaccinated 18–25 year-olds is "representative," we assume that the case rates for G over the period October–December match those from Table 2 above. Thus, we expect over the 61 days of October and November $(61/7) \times 3303$ cases per million ≈ 28783 cases in the group G, and we expect over the 31 days of December $(31/7) \times 7456$ cases per million = 33418 cases in the group G. We assume the CDC's case to infections multiplier of 4 from the period through 30 September 2021 continues to hold for October–December (recall that this likely a conservative assumption owing to increased home testing for COVID starting in August 2021[59]). Thus, we anticipate approximately $4 \times 28783 \approx 115{,}132$ infections within G over October and November and approximately $4 \times 33418 \approx 133672$ infections within G over December. Because recent infection provides high protection against reinfection, we estimate 248,804 ($\approx 115132 + 133672$) different members of G became infected with COVID during October–December 2021. In Section S3 of this supplement, we estimated that of these infections, about 80,000 were reinfections; we further assume that these reinfections are of persons in G previously infected during the period before 10/1/21–12/31/21.

Consider now our hypothetical group G at the end of September 2021. Note that it is still a representative group—even though we are assuming that 248,804 of its members are infected over October–December. and that about 80,000 of these infections are reinfections, this also would have been our prediction for infections had we started with a representative group of one million unvaccinated 18–

[59] See Rader, B., Gertz, A., Iuliano A.D., Gilmer, M., Wronski, L., Astley, C.M. Sewalk, K. Varrelman, T.J., Cohen, J., Parikh, R., Reese, H.E., et al. Use of At-Home COVID-19 Tests—United States, August 23, 2021–March 12, 2022. MMWR Morb. Mortal. Wkly. Rep. 2022;71:489–494. doi: http://dx.doi.org/10.15585/mmwr.mm7113e1.

25 year-olds at the end of September. Thus, we assume that infection rates for G over April-September are still those of the general unvaccinated population, and thus are given by Table 1 of [32] appearing above.

Observe that it is more likely for the 80,000 reinfections to be from older infections—say, those before April of 2021, than for more recent infections, say, from the period April–September 2021. Note that assuming reinfections are of older infections will result in G's having greater prior-infection protection as a group. We will assume, conservatively, that 40,000 of the reinfections come from infections April–September 2021 and 40,000 come from the period before April 2021. We will also assume, conservatively, that the 40,000 reinfections are distributed across the months from April to September of 2021 according to case totals (e.g., we assume that there were $\frac{\text{Number of cases April 2021}}{\text{Number of cases April through September 2021}} \times 40{,}000$ infections in April 2021 with corresponding re-infections occurring in October – December 2021).

Based on data from Table 1 of the preceding page, we anticipate for the period July–September 2021, a total of $4 \times (92/7) \times 4601 \approx 241881$ infections, for June 2021, $4 \times (30/7) \times 64 \approx 10971$ infections, and for April–May, $4 \times (61/7) \times 1638 \approx 57096$ infections. Of these 241881+10,971+ 57096 = 309,948 infections, we assume 269,948 (=309, 948-40,000) are among member of G different from those infected September–December. Thus, 4/1/21 through 12/31/2021, we know 248,804+ 269,948 = 519,752 of our group G will be infected. Because we are assuming 760000 different members of G are infected by 12/31/21, we see that 241248 = 760,000-517948 were infected before April 1, 2021.

To estimate the average effectiveness level over the evaluation period of prior-infection protection for previously infected members of G ,we take the average of (i) an overall effectiveness estimate EE_s for the beginning of the evaluation period (effectiveness as of 1/1/22) for those in G infected before 1/1/22 and (ii) an overall effectiveness estimate EE_e for the end of the evaluation period (effectiveness as of 5/31/22) for those in G infected before 1/1/22. We use the average of the 1/1/22 estimate EE_s and the 5/31/22 estimate EE_e to approximate average effectiveness over the evaluation period. To compute each of EE_s and EE_e, we estimate infections per month from January 2020 through December 2021 and compute a weighted average effectiveness estimate following the form of (†) on page 12 in Section S4 but replace waves with months: Recall that we are assuming 760,000 members of G were infected before the evaluation period. Enumerate the months January 2020 through December 2021 as "Month 1" (January 2020) through "Month 24" (December 2021), and let $PM_s(k)$ approximate the effectiveness of prior-infection protection, as of 1/1/21, for those infected in month k (and not re-infected before 1/1/22) while $PM_e(k)$ approximates the effectiveness of prior-infection protection, as of 5/31/22, for those infected in month k (and not re-infected before 5/31/22). We have

$$EE_s = \sum_{k=1}^{24} \frac{\text{Number infected in Month } k}{760{,}000} \times PM_s(k) \text{ and } EE_e = \sum_{k=1}^{24} \frac{\text{Number infected in Month } k}{760000} \times PM_e(k).$$

To complete the computation, we enumerate the days from January 1, 2020 (day 0 of a leap year) to December 31,2025 (day 730) and rely on the functions $PD_s(t) = PD(t-47)$ and $PD_e(t) = PD_s(t-150)$, where

$$PD(t) = \begin{cases} (13/172)(t-370) + 40, & t \leq 370 \\ (2/19)(t-560) + 60, & 370 < t < 623 \\ 66.6, & t \geq 623. \end{cases}$$

is the function defined on page 13 of Section S4. For t between 0 and 730, $PD_s(t)$ gives the effectiveness of prior-infection protection, as of 1/1/22, for a person infected on day t and $PD_e(t)$ gives the effectiveness of prior-infection protection, as of 5/31/22, for a person infected on day *t*. We obtain the monthly estimates $PM_s(k)$ and $PM_e(k)$ of effectiveness of prior-infection protection using the mid-point of each month. For months that have 31 days we take the 16th to be the midpoint while for those with 15 days, we take the 15th. For February 2020 (which has 29 days) we take the 15th as the midpoint and for February 2021, we take the 14th as the midpoint. Example: for March of 2020, we have $PM_s(3) = PD_s(75) \approx 14.2\%$ and $PM_e(3) = PD_e(75) \approx 2.8\%$. These effectiveness estimates appear in columns 5 and 7 of the spreadsheet on the following page. The weighted average estimates of effectiveness EE_s and EE_e appear in the final row, columns 6 and 8, with $EE_s \approx 52\%$ and $EE_e \approx 39\%$. We take the average of these, 45%. (rounded down) as our estimate of effectiveness of prior-infection protection E_{pi}.

Month and Year	Cumulative Cases[c] through Month in Column 1	Cases during Month in Column 1	Number Infected[d] during month in Column 1	Estimated protection level for month at start of evaluation period[e]	Month's contribution to weighted-average effectiveness estimate at start of evaluation period[f]	Estimated protection level for month at end of evaluation period[g]	Month's contribution to weighted-average effectiveness estimate at end of evaluation period[h]
Jan 2020[a]	7	7	0.06	9.6	0.00	0	0.00
Feb 2020[b]	69	62	0.49	11.9	0.00	0.5	0.00
Mar 2020	192078	192009	1514.80	14.2	0.03	2.8	0.01
Apr 2020	1,081,149	889,071	7,014.07	16.4	0.15	5.0	0.05
May 2020	1,791,546	710,397	5,604.47	18.8	0.14	7.4	0.05
Jun 2020	2,648,796	857,250	6,763.03	21.0	0.19	9.7	0.09
Jul 2020	4,548,496	1,899,700	14,987.13	23.4	0.46	12	0.24
Aug 2020	6,046,488	1,497,992	11,817.97	25.7	0.40	14.4	0.22
Sep 2020	7,246,199	1,199,711	9,464.77	28.0	0.35	16.6	0.21
Oct 2020	9176351	1,930,152	15,227.38	30.3	0.61	19.0	0.38
Nov 2020	13,667,914	4,491,563	35,434.89	32.6	1.52	21.3	0.99
Dec 2020	20,219,877	6,551,963	51,689.82	34.9	2.37	23.6	1.61
Jan 2021	26,357,106	6,137,229	48,417.89	37.3	2.38	25.9	1.65
Feb 2021	28,764,297	2,407,191	18,990.84	39.4	0.98	28.1	0.70
Mar 2021	30,579,484	1,815,187	14,320.39	42.4	0.80	30.4	0.57
April 2021	32,468,054	1,888,570	32,586.25	45.6	1.96	32.7	1.40
May 2021	33,386,446	918,392	15,846.36	48.8	1.02	35.0	0.73
June 2021	33,782,324	395,878	9749.16	52.0	0.67	37.3	0.48
July 2021	35,107,403	1,325,079	28758.76	55.3	2.09	39.6	1.50
August 2021	39,395,087	4,287,684	93057.44	58.5	7.16	42.7	5.23
Septembe r 2021	43,539,595	4,144,508	89950.03	61.7	7.30	45.9	5.43
October 2021	46,053,360	2,513,765	57120.48	64.9	4.88	49.2	3.70
November 2021	48,606,338	2,552,978	58011.52	66.6	5.08	52.3	3.99
December 2021	54,907,716	6,301,378	133672.00	66.6	11.71	55.6	9.78
Overall Effectiveness Level					52.25		39.00

[a] CDC Museum COVID-19 Timeline. Entry for 30 January 2020. David J. Sencer CDC Museum: In Association with the Smithsonian Institution. Available online: https://www.cdc.gov/museum/timeline/covid19.html

[b] February 29 coronavirus news. CNN. 29 February 2020. "There are 69 confirmed and presumptive positive cases of coronavirus in the US, CDC says." Available online: https://www.cnn.com/asia/live-news/coronavirus-outbreak-02-29-20-intl-a

[c] From Johns Hopkins Coronavirus Resource Center. United States. Data Timeline. Number of Daily Cases. Available online: https://coronavirus.jhu.edu/region/united-states

[d] For Jan 2020 through March 2021 computed as
(Cases for the corresponding month)/(Sum of cases Jan 2020 through March 2021) ⨉ 241248.
For April and May 2021 computed as
(Cases for the corresponding month)/(Sum of cases for April and May 2021) ⨉ 57096 –
(Cases for the corresponding month)/(Sum of cases April through September 2021) ⨉ 40000
For June 2021 computed as
Cases for June - (Cases for June)/(Sum of cases April through September 2021) ⨉ 40000
For July through September of 2021 computed as
(Cases for the corresponding month)/(Sum of cases for July through September 2021) ⨉ 241248 –
(Cases for the corresponding month)/(Sum of cases April through September 2021) ⨉ 40000
For October and November of 2021 computed as
(Cases for the corresponding month)/(Sum of cases for October and November 2021) ⨉ 115132
For December 2021, 133672.00

[e] Computed using $PD_s(t) = PD(t - 47)$

[f] Computed as (Number infected during Month)/760,000 X Estimated protection level for month at start of evaluation period

[g] Computed using $PD_e(t) = PD_s(t - 150)$,

[h] Computed as (Number infected during Month)/760,000 X Estimated protection level for month at end of evaluation period
