# Supplementary material for: A Reanalysis of the FDA’s Benefit–Risk Assessment of Moderna’s mRNA-1273 COVID Vaccine Based on a Model Incorporating Benefits Derived from Prior COVID Infection
Source: Vaccines (Basel). 2026 Feb 10;14(2):165. doi: 10.3390/vaccines14020165 (PMC12945011; doi:10.3390/vaccines14020165)
Supplement: Supplementary file 1 [file vaccines-14-00165-s001.zip › Supplement_S2_4071938.pdf]

Supplement S2: Validating the Model Using COVID-19 Hospitalization Data from Ontario[1]

S1. Introduction

In our paper "A reanalysis of the FDA's benefit-risk assessment of Moderna's mRNA-1273 COVID vaccine based on a model incorporating benefits derived from prior COVID infection," we examined the FDA's modeling of the hospitalizations-prevented benefit of mRNA-1273 vaccination and proposed an alternative model that accounts for prior-infection protection as well as incidental hospitalizations. The principal purpose of this supplement is to use real-world COVID-19 hospitalization data to test our predictions of the hospitalizations-prevented benefit of mRNA-1273 vaccination as well as corresponding predictions made by the FDA. We will also show that our hospitalizations modeling functions U and V provide estimates for Omicron-caused hospitalizations consistent with real-world hospitalizations data from Ontario for the period 1 January 2022 through 31 May 2022.

Applying our model to predict the hospitalizations-prevented benefit of mRNA-1273 vaccination over the evaluation period 1/1/22 – 5/31/22 to real-world COVID hospitalizations data requires several adjustments:

(i) For reasons explained in Section S3 below, we believe that real-world COVID-hospitalization data from Ontario is more reliable than that from the United States. In making comparisons between Ontario COVID-hospitalization rates and those for the U.S., we will need to account for the overall better health of Ontarians versus U.S. residents [1]. We also need to consider Pfizer BNT162b2 vaccination (and to a lesser extent AstraZeneca COVISHIELD vaccination) to be roughly equivalent to Moderna mRNA-1273 vaccination in terms of its effectiveness against COVID-19 hospitalization. According to the dataset used to produce Figure 3 of [2], the numbers of second doses of COVID-19 vaccines administered in Ontario through 1/1/2022 are as follows: Pfizer 7,428,264 (66.0%), Moderna 3,595,303 (32.0%), and AstraZeneca 223,674 (2.0%). Data from Table 3a of [3] indicates that Pfizer's and Moderna's mRNA vaccines offer very similar protection against Omicron BA.1 and BA.2 hospitalization, with AstraZeneca's vaccine offering slightly lower protection.

(ii) For a benefit-risk test population of 1 million unvaccinated persons with fraction having prior COVID-19 infection F_{pi}, recall that we project hospitalizations over the evaluation period (i) assuming the test population remains unvaccinated throughout the period and (ii) assuming the population is vaccinated throughout the period (with all members 14 days past dose 2 on the first day of the evaluation period). In either case, the fraction of the population—unvaccinated or vaccinated—having had a prior infection is the same: F_{pi}. However, in January 2022, we'd expect a representative population of 1 million *unvaccinated* 18–25 year-olds to have a higher fraction having experienced a prior infection than a corresponding representative population of *vaccinated* 18–25 year-olds. Thus, in this supplement, we let ${}_uF_{pi}$ be the fraction of a representative population of unvaccinated 18–25 year-olds having had a COVID-19 infection before 1 January 2022, and we let ${}_vF_{pi}$ be the fraction of a representative population of vaccinated 18–25 year-olds having had a COVID-19 infection before 1 January 2022. We assume ${}_vF_{pi} \leq {}_uF_{pi}$.

(iii) We expect that owing to waning of vaccine protection against infection (symptomatic and asymptomatic) the effectiveness estimate $E_v = 0.30$ (relative to the infection-naïve unvaccinated) used in our benefit-risk modeling likely overstates the real-world effectiveness of mRNA vaccination during 1 January 2022 through 31 May 2022. E.g., Figure 4 of [4] indicates that over 56% of those in the 18–29 age group in Ontario were fully vaccinated as of 7/31/2021 and thus on 1/1/22 were 5 months beyond the point of their full vaccination. Data from, e.g., the UK's Health Security Agency's Week 51 (23 December 2021) COVID-19 vaccine surveillance report [5] (see, e.g., Figure S2.1 below) suggests that mRNA vaccination provides little or no protection against symptomatic infection 5 months beyond dose 2 (and protection against all infection is not likely to be higher based on, say, Table 3 from the UK HSA's' 24 March 2022 COVID-19 vaccine surveillance report [6]). In fact, a study [7] titled "Real-world COVID-19 vaccine effectiveness against the Omicron BA.2 variant in a SARS-CoV-2 infection-naive population" provides the following estimates of protection provided by full vaccination (2 doses

[1] Supplement to "A reanalysis of the FDA's benefit-risk assessment of Moderna's mRNA-1273 COVID vaccine based on a model incorporating benefits derived from prior COVID infection" by P.S. Bourdon, R. Duriseti, H.C. Gromoll, D.K. Dalton, K. Bardosh, and A.E. Krug.

of BNT162b2) and full vaccination plus a booster (3 doses of BNT162b2) *relative to infection-naïve unvaccinated persons*: "Assuming VE took full effect 7 days after vaccination, we estimated: (1) VE for the second, third or fourth doses of BNT162b2 were 13% (95% credible interval: 2–39%), 48% (34–64%) and 69% (46–98%) 7 days following immunization, respectively, waning to 7% (1–21%), 26% (7–41%) and 35% (10–71%) 100 days after immunization." In fitting our model to Ontario hospitalization data, we will assume that over the evaluation period, owing to waning of protection and properties of Omicron variant, the effectiveness of mRNA vaccination against all COVID infection, relative to the infection-naïve unvaccinated is between 1% and 13%; that is $0.01 \leq E_v \leq 0.13$.

Figure S2.1: Vaccine Effectiveness Against Symptomatic Disease from Figure 6 of [4]

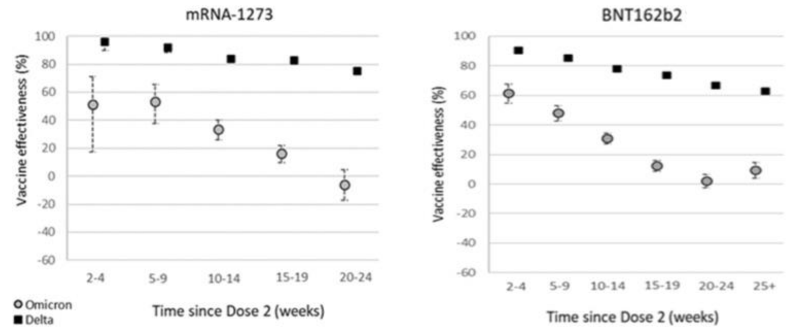

(iv) Based on data from [8], which found overall hybrid protection against hospitalization to be 97.4% at 12 months, we also adjust our value of the hospitalization-risk reduction for hybrid protection HRR_h from 0.88 to 0.94, which yields overall hybrid protection of $1 - (1 - 0.57)(1 - 0.94) \approx 97.4\%$, where 0.57 is the value we used in Scenarios A–E for E_h, the effectiveness against re-infection provided by hybrid protection.[2]

(v) Because Public Health Ontario does not provide sex-specific COVID-hospitalizations data, to test our model using its data, we compute model outputs assuming equivalent infection and hospitalization risk for males and females—see Table S2.2 below.

In the next section, we conduct sensitivity analyses, exploring how our model's benefit-risk outcomes change when input assumptions are changed, e.g., when no sex-based differences in infection and hospitalization rates are assumed or when we include the hospitalizations benefit of a single dose of mRNA-1273 during the period between dose 1 and 14 days past dose 2. In Section S3, we explain why we test our model using COVID-19 hospitalization data from Ontario rather than from the U.S. Then, in Section S4, we compute a "better-health adjustment" for Ontarians vs. U.S. residents, reducing our COVID-19 infection-hospitalization rate from 0.22% for those 18–25 in the U.S. (males & females combined) to 0.17% for those 18–25 in Ontario (a drop of about 23%). In Section S5, we present seroprevalence data suggesting that over the evaluation period 1/1/22 – 5/31/22 the increase in the COVID-19 infection level in Ontario substantially exceeded that in the U.S. In Section S6, we use data from Public Health Ontario to estimate the evaluation-period COVID-19 hospitalization rate per million population for the 18–29 age range and then use the IHR model [9] to estimate the corresponding rate for the 18–25 range. In Section S7, we use Ontario COVID-hospitalization rates to obtain estimates of corresponding rates for the U.S. using the better-health adjustment discussed in Section S4. We find that mRNA-1273 vaccinating 1 million 18–25 year-olds in the U.S. (roughly 50% males and 50% females), with all being 14 days past dose 2 on 1/1/22, likely would have prevented at most 214 evaluation-period COVID hospitalizations (non-incidental hospitalizations). We have asserted "at most 214 hospitalizations" because in obtaining estimates of U.S. COVID hospitalizations from Ontario hospitalizations, we ignore the greater rise in COVID-infections in Ontario vs. the U.S. over the evaluation period. Moreover, because young males are hospitalized with COVID at lower rates than young females ([10, Table 2], [11, Table 1]), we would expect fewer than 214 hospitalizations prevented per million vaccinated males 18–25. In Section S8, we show that there are inputs consistent with evaluation-period data from Ontario such that our modeling functions U and V yield outputs exactly matching Ontario-hospitalizations data for the evaluation period.

Appendix S1 provides tables of COVID-hospitalization data by age and vaccination status upon which we rely to estimate the number of "with COVID" hospitalizations in Ontario over the evaluation period among 18–29

[2] The 97.4% estimate for hybrid protection against hospitalization may be an overestimate owing to a number of biases, including, e.g., failure to make adequate adjustment for comorbidity status [8, p. 560].

year-olds. Appendix S2 describes our method of estimating the fraction of unvaccinated Ontarians ages 18–29 having had a COVID infection before 1/1/22. In Appendix S3, we use VAM/P data from Public Heath Ontario (6/1/21–9/4/21) and CDC VSD data (through 10/9/21) to show that among males and females ages 18–39, the number VAM/P cases per million second doses of mRNA-1273 detected by Public Health Ontario was 3.76 times the number that the CDC's VSD system detected (each case occurring during 7-day risk window after the dose). Finally, in Appendix S4, we present data suggesting that those having a COVID infection prior to COVID vaccination are at a greater risk of experiencing postvaccination myocarditis.

S2. **Sensitivity Analyses**

S2.1 Exploring Equipoise. For Scenario D, our most likely scenario, model-input values are as follows $\mathrm{I_r} = 0.456, \mathrm{H_r} = 0.0014, \mathrm{F_{pi}} = 0.69, \mathrm{E_{pi}} = 0.45, \mathrm{E_v} = 0.30, \mathrm{E_h} = 0.57, \mathrm{HRR_{pi}} = 0.79, \mathrm{HRR_v} = 0.67,$ and $\mathrm{HRR_h} = 0.88$, and $H_{VAM/P} = 268$. Recall that for Scenario D we based our assumption that 45.6% of the infection-naïve unvaccinated would contract Omicron over the evaluation period on the prediction that the infection-level rise in the general population over the period would be twice that of the second COVID wave in the U.S.

Benefit-risk analyses are quite sensitive to infection-prevalence assumptions. Thus, it is natural to consider the following question: if for Scenario D all input variables except for $\mathrm{I_r}$ are held constant how much can $\mathrm{I_r}$ increase before benefit-risk equipoise is reached? A computation shows that if $I_r = 0.678$ and values of other model inputs are as in Scenario D, then $U \approx 370,\ V \approx 102$, so that 370-102 = 268 per million vaccinations is the COVID-hospitalizations-prevented benefit of vaccination, which equals our estimated VAM/P hospitalization risk (per million full vaccinations). Thus, the projected Scenario D infection-level increase would have to rise from 0.456 to 0.678 to reach equipoise, a rise of nearly 50%.

Turning to our other scenarios, If all input variables except for $\mathrm{I_r}$ are held constant, then benefit-risk equipoise is reached when $I_r \approx 0.577$ for Scenarios A–C, and $I_r \approx 0.528$ for Scenario E.

Another natural equipoise question to raise is the following: if for Scenario D all input variables except for the IHR H_r are held constant, how much can H_r increase before benefit-risk equipoise is reached? A computation shows that $H_r \approx 0.00208$ yields equipoise, a value nearly 50% higher than our estimate $H_r \approx 0.0014$. We remark that our modeling work of Section S8 below suggests that for the general population of 18–25-year-old Ontarians IHR ≤ 0.0011 during the evaluation period. This supports the plausibility of 0.0014 for 18–25-year-old *males* in the U.S. (We'd expect to adjust the Ontario IHR upward to arrive at a U.S. IHR owing to Ontarians overall better health, but a downward adjustment would also be needed because we expect the IHR for 18–25-year-old *males* to be lower than the IHR for the general population of 18–25 year-olds.) We note that COVID-hospitalizations data from the State of Connecticut (see Appendix S1 of Supplement S1) suggests that our Omicron IHR value 0.0014 for 18–25-year-old males is a reasonable estimate even if we assume males and females in this age range are hospitalized for COVID at roughly equivalent rates.

So far, for Scenario D, we have assessed the extent to each of the inputs I_r and H_r can vary before equipoise is reached assuming all other variables are held constant at their Scenario D values. The graphic below provides similar extents for all the other inputs.

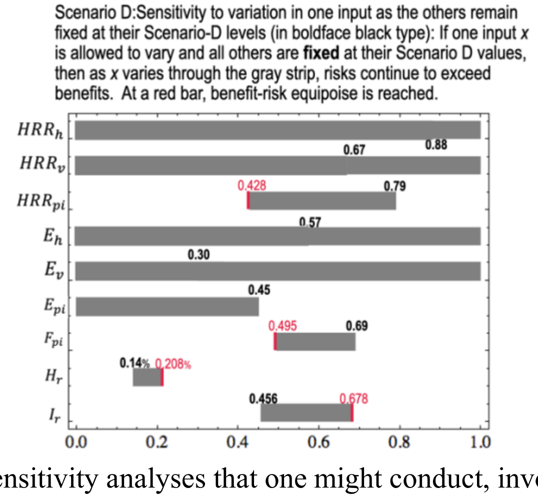

Of course, there are other sensitivity analyses that one might conduct, involving even combinations of variables. Such analyses are facilitated via slider-based plotting, an example of which is illustrated below.

Figure S2.2: Slider plot displaying equipoise curve such that if (F_{pi}, I_r) lies on the curve, with values of all variables other than F_{pi} and I_r set by the sliders on the left, then equipoise is achieved.

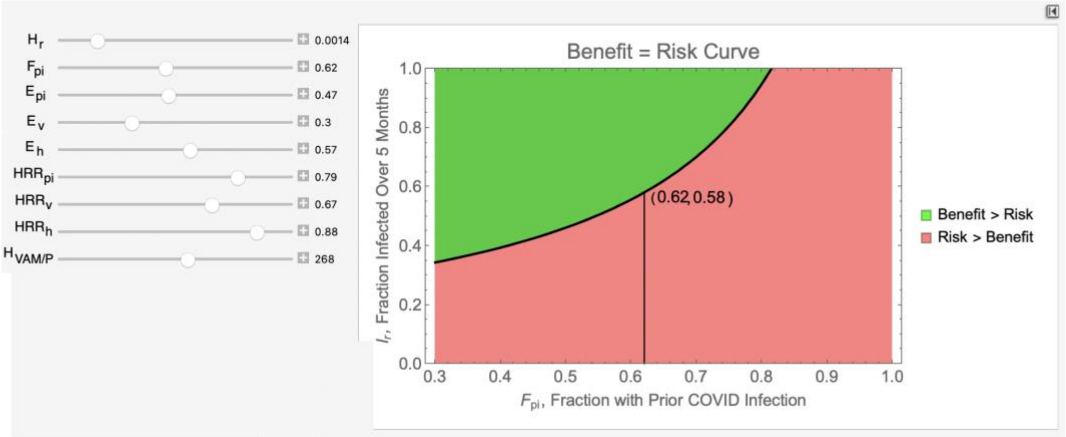

The preceding figure suggests that for plausible values of F_{pi} and I_r vaccination risks exceed benefits (assuming values for all other variables are set by the sliders on the left).

For the sensitivity analyses discussed above involving our most likely scenario (Scenario D), we see that equipoise is not reached for plausible values of I_r or H_r. Scenario E with I_r increased to 0.528 represents the most plausible equipoise situation considered, but Scenario E is our best-case scenario for vaccination. These sensitivity analyses, were they carried in early January of 2022, increase confidence that the benefit-risk ratio for mRNA-1273 vaccination would be less than 1.

S2.2 Including Dose 1 Benefits. To include in our modeling the hospitalizations-prevented benefit of mRNA-1273 vaccination during the period between the day dose 1 is administered and 13 days past dose 2, we make the following assumptions:

(a) The dose 1 benefit period extends from day 14 through day 41, with "day 0" being the day that dose 1 is administered and "day 41" being 13 days after dose 2 is administered, with dose 2 being administered on day 28. This assumption is consistent with Public-Health Ontario's description of the protection period provided by dose 1 appearing in Table 1 of [12]:

"Not yet protected: 0-13 days after dose 1"; "Partially vaccinated: 14+ days after dose 1 and 0-13 days after dose 2."

The "no-benefit" period, days 0 through 13, is also consistent with plots [13, Figure 3] and [14, Figure 3B] showing, respectively, for the BNT162b2 and mRNA-1273 phase-3 trials, divergence in COVID cases among the placebo and vaccinated groups starting at approximately day 12.

(b) For simplicity, we will take the 28-day benefit period for dose 1 (days 14–41) to be one month and analyze the hospitalizations-prevented benefit of mRNA-1273 over a 6-month evaluation period, with dose 1 providing partial protection over the first month and dose 2 providing additional protection starting at the beginning of month 2. We assume the six-month evaluation period is 1/1/22–6/30/22.

(c) We assume effectiveness factors for protection from vaccination are constant over the first month, reflecting the protection of partial vaccination, and constant (at a higher level) over months 2 through 6, reflecting the protection of both vaccine doses.

(d) We assess vaccination's benefits and risks via Scenarios A_1–E_1, which are analogues of our Scenarios A–E from the main exposition. In particular, in Scenarios A_1–D_1, we assume that during the period 2/1/22–6/30/22, $\mathrm{E_{pi}} = 0.45, \mathrm{E_v} = 0.30, \mathrm{E_h} = 0.57, \mathrm{HRR_{pi}} = 0.79, \mathrm{HRR_v} = 0.67,$ and $\mathrm{HRR_h} = 0.88$, which are the values for these model inputs used in our Scenarios A–D.

The principal challenge of assessing the benefits of dose 1 is that of using data available before 1/22/22 to arrive at plausible levels of protection against Omicron provided by a single dose of mRNA-1273. We begin with hospitalization-risk reduction and depend on data from Tables 2 and A1a from [12], which yields the following:

COVID case-hospitalization rates for 18–29 year-olds in Ontario during the period 12/10/20–12/12/21		
Partially Vaccinated	Fully Vaccinated	Unvaccinated
0.39%	0.23%	0.92%

The preceding rates suggest that for pre-Omicron variants present in Ontario during the period 12/10/20–12/12/21, the vaccines used in Ontario (Pfizer, 66.0%; Moderna, 32.0%; and AstraZeneca, 2.0%, as of 1/1/22), provided a hospitalization-risk reduction relative to the unvaccinated of about 75% for full vaccination and 57.6% for partial vaccination. These rates seem plausible given our estimate that mRNA-1273 vaccination (among the COVID-naïve) provided a hospitalization-risk reduction of 67% against Omicron. Note that 57.6 is 23.2% lower than 75; thus, we assume that one-dose of mRNA-1273 provides against Omicron, over the first month of the evaluation period, a hospitalization-risk reduction of $51.5\% \approx 0.768 \cdot 67\%$. Using lower-case letters in our identifiers for partial-vaccination model inputs, we have $hrr_v = 0.515$. To estimate hrr_h, hybrid protection against Omicron provided by prior infection and the first dose of mRNA-1273, we subtract 23.2% of the difference $HRR_h - HRR_{pi}$ from $HRR_h = 0.88$, obtaining $hrr_h = 0.859$ for Scenarios A_1–D_1 and $hrr_h = 0.831$ for Scenario E_1.

We now turn to the task of estimating e_v, the effectiveness of 1 dose of mRNA-1273 in preventing any COVID infection during the period beginning 14 days after dose 1 up to the point of full vaccination. Even though Moderna's phase-3 clinical trial had as its primary endpoint the prevention of *symptomatic* COVID-19 in the per-protocol population, data was collected allowing an estimate of the effectiveness of dose 1 against all infection (symptomatic as well as asymptomatic). We note that in its report on its phase-3 clinical trial [14], Moderna labels the day dose 1 is administered "day 1," so that day 29 is the day the second dose is administered. Here is the key passage from Moderna's clinical-trial report providing data on which we rely: "Common reasons for not receiving the second dose were withdrawal of consent (153 participants) and the detection of SARS-CoV-2 by PCR before the administration of the second dose on day 29 (114 participants: 69 in the placebo group and 45 in the mRNA-1273 group)" [14, p. 409].[3] We assume that the purpose of PCR testing prior to dose 2 was to ensure that no participant who had a potentially active COVID infection on day 29 received a second dose that day.

Other relevant data, which we compare to the 69 placebo-group cases and 45 treatment-group cases detected via PCR testing before the administration of the second dose, appears in Figure 3B of [14] displaying data from the Modified-Intention-to-Treat Population (which, e.g., excludes those having evidence of infection at baseline):

Covid-19 Onset	Placebo (N=14,598)	mRNA-1273 (N=14,550)
Randomization to 14 days after dose 1	11	5
14 Days after dose 1 to dose 2	35	2
Dose 2 to 14 days after dose 2	19	0
Starting 14 days after dose 2	204	12
Total (any time after randomization)	269	19

Note that before the administration of the second dose, there were 46 symptomatic cases among the placebo group and 7 among the vaccinated group. We conclude that among the 69 placebo-group cases detected via PCR testing before the administration of the second dose, 46 were symptomatic and among the 45 treatment-group cases 7 were symptomatic (suggesting that 33% of the unvaccinated cases and 84% of vaccinated cases were asymptomatic or slightly symptomatic). We also conclude that during the period starting 14 days past dose 2 and ending 13 days after dose 2, there were at least 69+19 = 88 COVID cases among the unvaccinated and at least 45 among the vaccinated, where we have said "at least" because there may have been undetected asymptomatic or slightly symptomatic cases during days 0 through 13 after the second dose. According to Figure 1 of [14], from the treatment and placebo groups, respectively, 15,181 and 15,170 received dose 1; thus, with our accounting of infections during the dose-1 protection period, the ratio of incidence-rates 45/15181 and 88/15170 is about 0.51, yielding an approximation of single-dose efficacy against any COVID infection during the dose-1 protection period of 49%.

An analysis of the efficacy of mRNA-1273 vaccination at the completion of the blinded portion of Moderna's phase-3 clinical trial found that primary-series vaccination provided 82% efficacy against any infection and 93.2% efficacy against symptomatic infection [15]. Because our single-dose efficacy estimate of 49% is about 40.2% lower than 82%, the corresponding efficacy of two doses, we assume that one-dose of mRNA-1273 provides against Omicron, over the first month of the evaluation period protection against any infection having effectiveness $17.9\% \approx 0.598 \cdot 30\%$. Hence, we have $e_v = 0.179$ compared to $E_v =$

[3] Because none of the participants having these 114 cases received a second dose, all these cases were excluded from the per-protocol population analysis of postdose 1 cases appearing Supplementary Table S16 of [14] even though some cases were symptomatic.

0.30. To estimate e_h, hybrid protection against Omicron provided by prior infection and the first dose of mRNA-1273, we subtract 40.2% of the difference $E_h - E_{pi}$ from $E_h = 0.57$, obtaining $e_h = 0.522$ for Scenarios A_1–D_1 and $e_h = 0.461$ for Scenario E_1.

Combining effectiveness against infection with hospitalization-risk reduction, we obtain the following estimates of overall single-dose effectiveness against Omicron hospitalization (during the 1st month of the 6-month evaluation period): $0.602 \approx 1-(1-e_v)(1-hrr_v) = 1-(1-0.179)(1-0.515)$ for vaccination alone and, for hybrid protection, $0.933 \approx 1-(1-e_h)(1-hrr_h) = 1-(1-0.522)(1-0.859)$ in Scenarios A_1–D_1, and $0.909 \approx 1-(1-e_h)(1-hrr_h) = 1-(1-0.461)(1-0.831)$ in Scenario E_1.

The preceding paragraph's VEH estimates of single-dose effectiveness during the first month of the evaluation period should be compared to the FDA's two-dose estimate of VEH = 0.72 and to our estimates of VEH ≈ 0.77 for two doses alone and 94.8% for hybrid protection for two doses.

We turn now to the computation of the prevention-of-hospitalizations benefit of vaccination over our 6-month evaluation period accounting for the benefits of dose 1 as well as dose 2. For Scenarios B_1–E_1, we assume that the fraction of infection-naïve unvaccinated who become infected over the first month of the evaluation period is 1/5 of the value of I_r from the corresponding scenario among Scenarios B–E. For Scenario A_1, we assume that 1/6 of the infection-naïve unvaccinated become infected during month 1 and assume all remaining infection-naïve unvaccinated become infected during months 2–6 by setting $I_r = 1$.

Table S2.1. Scenarios projecting the hospitalizations-prevented benefit of mRNA-1273 vaccination of a hypothetical test population of 1 million males ages 18–25, with the penultimate column providing an estimate of the corresponding hospitalization risk owing to VAM/P. Benefits are computed over a 6-month period with one-dose benefits estimated for the first month and two-dose benefits estimated for months 2–6.*

Scenario	Assumptions Month 1 One-Dose Protection	Assumptions Months 2–6 Two-Dose Protection	Hosp. Prevented Per Million Month 1 $U-V$	Hosp. Prevented Per Million Months 2–5 $U-V$	Hosp. Prevented Per Million Months 1–6	$H_{VAM/P}$	Benefit Risk Ratio
A_1	$I_r = \frac{1}{6}, H_r = 0.0014,$ $F_{pi} = 0.62, E_{pi} = 0.45,$ $e_v = 0.179, e_h = 0.522,$ $HRR_{pi} = 0.79, hrr_v = 0.515,$ $hrr_h = 0.859$	$I_r = 1, H_r = 0.0014,$ $F_{pi} = 0.683, E_{pi} = 0.45,$ $E_v = 0.30, E_h = 0.57,$ $HRR_{pi} = 0.79, HRR_v = 0.67,$ $HRR_h = 0.88$	105 – 45<br>60	554 – 152<br>402	462	268	1.72
B_1	$I_r = 0.071, H_r = 0.0014,$ $F_{pi} = 0.62, E_{pi} = 0.45,$ $e_v = 0.179, e_h = 0.522,$ $HRR_{pi} = 0.79, hrr_v = 0.515,$ $hrr_h = 0.859$	$I_r = 0.356, H_r = 0.0014,$ $F_{pi} = 0.647, E_{pi} = 0.45,$ $E_v = 0.30, E_h = 0.57,$ $HRR_{pi} = 0.79, HRR_v = 0.67,$ $HRR_h = 0.88$	45 – 19<br>26	213 – 57<br>156	182	268	0.68
C_1	$I_r = 0.01, H_r = 0.0014,$ $F_{pi} = 0.62, E_{pi} = 0.45,$ $e_v = 0.179, e_h = 0.522,$ $HRR_{pi} = 0.79, hrr_v = 0.515,$ $hrr_h = 0.859$	$I_r = 0.052, H_r = 0.0014,$ $F_{pi} = 0.624, E_{pi} = 0.45,$ $E_v = 0.30, E_h = 0.57,$ $HRR_{pi} = 0.79, HRR_v = 0.67,$ $HRR_h = 0.88$	6 – 3<br>3	33 – 9<br>24	27	268	0.10
D_1	$I_r = 0.091, H_r = 0.0014,$ $F_{pi} = 0.69, E_{pi} = 0.45,$ $e_v = 0.179, e_h = 0.522,$ $HRR_{pi} = 0.79, hrr_v = 0.515,$ $hrr_h = 0.859$	$I_r = 0.456, H_r = 0.0014,$ $F_{pi} = 0.718, E_{pi} = 0.45,$ $E_v = 0.30, E_h = 0.57,$ $HRR_{pi} = 0.79, HRR_v = 0.67,$ $HRR_h = 0.88$	50 – 22<br>28	233 – 65<br>168	196	268	0.73
E_1	$I_r = 0.091, H_r = 0.0014,$ $F_{pi} = 0.69, E_{pi} = 0.30,$ $e_v = 0.179, e_h = 0.461,$ $HRR_{pi} = 0.67, hrr_v = 0.515,$ $hrr_h = 0.831$	$I_r = 0.456, H_r = 0.0014,$ $F_{pi} = 0.718, E_{pi} = 0.30,$ $E_v = 0.30, E_h = 0.57,$ $HRR_{pi} = 0.67, HRR_v = 0.67,$ $HRR_h = 0.88$	60 – 24<br>36	286 – 65<br>221	257	268	0.96

* Remarks: In Scenarios D_1 and E_1, over 50% of the infection naïve unvaccinated become infected over the 6-month evaluation period.

Thus, we have validated a claim made in Section 1.4 of the main exposition: accounting for dose 1 benefits during the period leading up to the point of full vaccination does not substantially change our benefit-risk conclusions—in natural analogues of Scenarios B-E, vaccine risks still exceed benefits for 18–25-year-old males, with vaccination generating between 4% and 47% more hospitalizations from VAM/P alone compared to COVID hospitalizations prevented over a six-month period of vaccine protection assumed by the FDA.

S2.3 Predicting the Hospitalizations-Prevented Benefit of Vaccination Assuming No Sex-Related Differences in COVID-Hospitalization and Infection Rates

In applying our model in the main exposition's Section 3, we assumed, as did the FDA in its benefits-risk assessments [10] and [16], that COVID-hospitalization rates for males and females differ significantly for those in the age range 18–25. Table 2 of [10] indicates that the COVID-hospitalization rate per 100,000 population among females 18–24 is 2.53 times that of males 18–24, while Table 1 of [11] indicates that the confirmed-case hospitalization rate among females 20–29 is 2.32 times that of males 20–29. In addition,

Table 2 of [10] suggests the COVID-case rate per 100,000 population among females 18–24 is 1.21 times that of males 18–24, while Table 1 of [11] indicates that among 3,607,403 COVID tests taken by 20–29-year-old Israeli males 3/1/20–3/1/22, 223,659 were positive (6.2%) while among 3,584,826 COVID tests taken by 20-29-year-old females, 308,295 were positive (8.6%), yielding a positivity rate for females 1.39 times that for males.

In Section S2 of Supplement S1, we derived an IHR of 0.14% for 18–25-year-old males in the U.S. and of 0.29% for 18–25-year-old females, with 0.22% being the estimated rate for the general 18–25 U.S. population consisting of males and females (roughly 50% male). Our derivation of different IHRs for males versus females relies on the following: the sex-based hospitalization rates described in Table 2 of [10] , the assumption that sex-based differences for the 18–24 age range well approximate those for the 18–25 range, the IHR-model of [9], and estimates of incidental-hospitalization rates. With an IHR of 0.14% for males, we found in the main exposition that benefits of mRNA-1273 vaccination outweigh VAM/P risk only for those scenarios of [16] for which the FDA assumed the infection rate for the unvaccinated would be essentially 100% over 5 months. For our scenarios that are based on more modest infection increases, namely Scenario B (35.6% increase among the infection-naïve unvaccinated) and Scenarios D and E (45.6% increase among the infection-naïve unvaccinated), we found benefit-to-risk ratios to be, respectively, about 0.62, 0.68, and 0.87. See Table 5 of the main exposition.

We now explore how the risk-benefit picture changes if we assume no sex-based differences in COVID infection and hospitalization risk, in which case, our work of Supplement S1 suggests a COVID-IHR over the evaluation period of R = 0.22% for males 18–25 (with the same IHR for females) and $F_{pi} = 0.76$.[4] Table S2.2 below provides the corresponding hospitalizations-based benefit-risk assessment, which should be compared to Table 6 of the main exposition. For Scenario D_2, our most likely no-sex-differences scenario, we assume $F_{pi} = 0.76$. We also assume $F_{pi} = 0.76$ for Scenario E_2. For Scenarios A_2–C_2, we apply a reduced estimate for F_{pi} of 0.65, the approximate average of (a) the CDC's estimate that, as of the end of September 2021, 54.9% of those 18–49 years old had been infected by COVID-19 [17] with (b) the estimate 76% derived in Supplement S1.

Table S2.2. Scenarios projecting the hospitalizations-prevented benefit of mRNA-1273 vaccination of a hypothetical test population of 1 million males ages 18–25 ***assuming males and females are at equal risk of COVID infection and hospitalization.***

Scenario	Description	Assumptions	U	V	Hospitalizations Prevented Per Million Vaccinations $U-V$	VAM/P Hospitalizations Expected Per Million Full Vaccinations	Benefit Risk Ratio
FDA Scenario 1	Highest 2021 COVID incidence assumed. FDA's "most likely scenario"	$U = 6619$ $V = 6619(1-0.72) \approx 1853$	6619	1853	4766	110 (2nd-dose only)	43.33
A_2	Reanalysis of FDA's Scenario 1	$I_r = 1, H_r = 0.0022,$ $F_{pi} = 0.65, E_{pi} = 0.45,$ $E_v = 0.30, E_h = 0.57,$ $HRR_{pi} = 0.79, HRR_v = 0.67,$ $HRR_h = 0.88$	935	252	683	268	2.55
FDA Scenario 2	Average 2021 COVID incidence assumed	$U = 2900$ $V = 2900(1-0.72) \approx 812$	2900	812	2088	110 (2nd-dose only)	18.98
B_2	Reanalysis of FDA's Scenario 2	$I_r = 0.356, H_r = 0.0022,$ $F_{pi} = 0.65, E_{pi} = 0.45,$ $E_v = 0.30, E_h = 0.57,$ $HRR_{pi} = 0.79, HRR_v = 0.67,$ $HRR_h = 0.88$	333	90	243	268	0.91
FDA Scenario 3	Lowest incidence of pandemic assumed (6/5/21)	$U = 882$ $V = 882(1-0.72) \approx 247$	882	247	635	110 (2nd-dose only)	5.77
C_2	Reanalysis of FDA's Scenario 3	$I_r = 0.052, H_r = 0.0022,$ $F_{pi} = 0.65, E_{pi} = 0.45,$ $E_v = 0.30, E_h = 0.57,$ $HRR_{pi} = 0.79, HRR_v = 0.67,$ $HRR_h = 0.88$	49	13	36	268	0.13
D_2	I_r twice that for the 2nd COVID wave in the U.S. Our "most likely scenario"	$I_r = 0.456, H_r = 0.0022,$ $F_{pi} = 0.76, E_{pi} = 0.45,$ $E_v = 0.30, E_h = 0.57,$ $HRR_{pi} = 0.79, HRR_v = 0.67,$ $HRR_h = 0.88$	329	95	234	268	0.87
E_2	Equivalent protection for prior infected and COVID-naïve vaccinated; I_r as in Scenario D.	$I_r = 0.456, H_r = 0.0022,$ $F_{pi} = 0.76, E_{pi} = 0.30,$ $E_v = 0.30, E_h = 0.57,$ $HRR_{pi} = 0.67, HRR_v = 0.67,$ $HRR_h = 0.88$	417	95	322	268	1.20

[4] In Appendix S1 of Supplement S1, we point out that COVID-hospitalization data from Connecticut suggests that H_r = 0.14% may be a reasonable estimate for a sex-neutral Omicron IHR.

Thus, we see from the preceding table that under the assumption of equivalent COVID hospitalization and infection rates among males and females 18–25 that VAM/P hospitalization risk exceeds the hospitalizations-prevented benefit of mRNA-1273 vaccination in Scenarios $B_2 – D_2$, but for Scenario E_2 the benefit-risk ratio rises above 1. Relative to Scenario D_2, one can check using the formulas for U and V from the main exposition that benefit-risk equipoise is reached when I_r is increased from 0.456 to about 0.523. We note that the value $F_{pi} = 0.76$ is quite possibly an underestimate, being based on the assumption that as of the end of September 2021 the percentage of unvaccinated 18–25 year-olds having had a prior COVID infection is the same as the percentage of the general population of 18–25 year-olds having had a prior COVID infection (and some in general population had vaccine protection for a portion of the period preceding 1 October 2021). If, say, we increase F_{pi} in Scenario D_1 from 0.76 to 0.8, then benefit-risk equipoise is reached when I_r is increased from 0.456 to about 0.594.

We have noted that relative to Scenario D_2, benefit-risk equipoise is reached when $I_r \approx 0.523$. Based on the following reasoning we assert that $I_r \approx 0.5$ is a reasonable estimate for the actual evaluation-period rise in infection-level in the U.S. among the infection-naive unvaccinated 18–25 year-olds. In Section S8 of this supplement, we arrive at scenarios in which our model (adjusted as described in Section S1) fits Ontario COVID-hospitalization data perfectly with plausible values for inputs suggested by Ontario data. For the scenario we consider to be "most likely," the value for I_r, the increase in infection level among infection-naïve unvaccinated 18–25 year-old Ontarians over the evaluation period, is 0.567. As discussed in Section S5, the ratio of COVID anti-N seroprevalence increases over the evaluation period for the 18–25 population in the U.S. vs. Ontario is $34.5/40 \approx 0.86$. This, combined with our finding of $I_r = 0.567$ being a plausible value of I_r for Ontarians 18–25, suggests $I_r = 0.86 \cdot 0.567 \approx 0.488 \approx 0.5$ is a plausible value of I_r for Americans 18–25. For males 18–25, we expect I_r to be lower.[5] With all inputs other than I_r having Scenario D_2 values and $I_r = 0.5$, the corresponding hospitalization benefit/risk ratio for mRNA-1273 vaccination becomes $257/268 \approx 0.96$.

We conclude this section by justifying a claim made in Section 3.4 of the main exposition:

> Ontario data also supports the definitiveness of the results of Table 6 above given the male has no comorbidities, suggesting that 151 in 1 million is an overestimate of his evaluation-period COVID hospitalization risk [Supplement S2, Section S2.3].

Table 6 of the main exposition compares hospitalization risks for vaccination and not vaccinating for a typical 18–25-year-old male in the US having prior-infection protection. We will call this male "Bob" *and suppose in addition that Bob has no comorbidities.* Table 6 suggests that Bob's hospitalization risk for COVID, assuming he remains unvaccinated is at most 161.7 in million. Based on Ontario data from the evaluation period 1/1/22–5/31/22, we estimate (Table S2.6 below) that the COVID-hospitalization rate for 18–29 year-olds (general population mix of males and females.) in Ontario was 264 in 1 million over the evaluation period. We claim 264 in one million clearly overstates Bob's risk of COVID-19 hospitalization over the evaluation period. Bob's risk is lower owing to a number of factors, including (i) according to the IHR model [9] the COVID-hospitalization rate for 18–25 year-olds should be 0.843 times the rate for 18–29 year-olds (see Section S6 below), (ii) the rate 264 per million is for the general population of 18–29 Ontarians (males & females), and females have a significantly higher COVID hospitalization risk than do males in the age range 18–29 [10, 11], (iii) Bob's COVID-hospitalization risk is lower than average for 18–29-year-old Ontarians because some have COVID comorbidities (and Bob does not) and some Ontarians do not have prior-infection protection, and (iv) Bob's chance of contracting an evaluation-period Omicron infection is lower than that of an average Ontarian because Bob lives in the U.S. and seroprevalence studies (see Section S5 below) indicate that over the evaluation period, there was a greater COVID-infection-level rise in Ontario than in the U.S. If we apply the risk reduction from (i), then we obtain an upper bound on Bob's COVID-hospitalization risk of about $0.843 \cdot 264 \approx 223$ in 1 million, and if we then apply the risk reduction of (ii) above, then we obtain an upper bound on Bob's COVID-hospitalization risk of about 151 in 1 million, with the latter estimate being obtained as follows: According to Table 2 of [10] for the 18–24 age range, females are 2.53 times as likely as males to be hospitalized with COVID-19; similarly Table 1 of [11] indicates that, for the 20–29 age range, a female with a confirmed case of COVID is 2.32 times as likely to be hospitalized for COVID as male. If we assume that females in the age range 18–25 are only twice as likely as males to be hospitalized for COVID and assume that 52% of the 18–25-year-old Ontarians are males (suggested

[5] Table 2 of [10] suggests that females are more likely to have a COVID case, with female to male case-rate ratio of 1.21. Similarly, Table 1 of [11] reports positivity rates for COVID tests among young females exceed those among young males, e.g., 8.6% vs. 6.2% for 20-29 year-olds.

by 2021 estimates from Statistics Canada), then if r is the 18–25 male COVID-hospitalization rate over the evaluation period we have $520{,}000r + 480{,}000 \cdot 2r = 223$, so that $r \approx 0.000151$, corresponding to a risk of 151 in 1 million. Note that 151 in 1 million is likely to overestimate risk because we have not accounted for reduced-risk factors (iii) and (iv) above.

S3. Comparing COVID-19 Hospitalizations Data from Public Health Ontario versus that from the U.S. Centers for Disease Control and Prevention.

Here are the reasons why we decided to test our model as well as the FDA's using COVID-19 hospitalizations data from Ontario rather than from the U.S. (provided by the CDC).

- We are interested in COVID-19 hospitalizations by vaccination status for the age range 18–25, and Public Health Ontario provides such data for the age range 18–29, which has an approximately 67% overlap with the target range 18–25. However, publicly available data from the CDC reporting COVID-19 hospitalizations by age and vaccination status has an insufficiently fine age stratification—the range the CDC once provided that most closely matches our target range is 18–49 (see, e.g., the figure in the FactCheck.org article [18]), which has only a 25% overlap with the 18–25 target range. The CDC-tracker website [19] "https://covid.cdc.gov/covid-data-tracker/#covidnet-hospitalizations-vaccination" that once reported COVID-19 hospitalizations by age and vaccination status no longer exists.
- The CDC does not provide any data on incidental COVID-19 hospitalizations (and Public Health Ontario does).
- In the U.S., "profitability is important for both for-profit and not-for-profit hospitals" and there is a positive relationship between profitability and occupancy rate [20]. Thus, in the U.S., there is a profit-related incentive to hospitalize COVID-positive persons that isn't present in Canada.
- Electronic health records "EHRs" in the U.S. do not accurately reflect the COVID-19 vaccination status of many of those hospitalized with or for COVID-19 (see the following paragraph). Thus, it is possible that some COVID-19 hospitalizations that the CDC classifies to be of unvaccinated individuals are actually of vaccinated individuals. Such misclassification might impact Ontario COVID-hospitalization data as well.

Data from the National COVID Cohort Collaborative (N3C), which "provides one of the largest secure collections of harmonized clinical health data in the United States,"[6] illustrates how dramatically EHRs in the U.S. may fail to present accurately vaccination status of COVID-19 patients. For instance, an N3C dashboard reports that as of 3/9/23 the vaccination status of 63.59% of COVID-positive N3C patients is unknown:

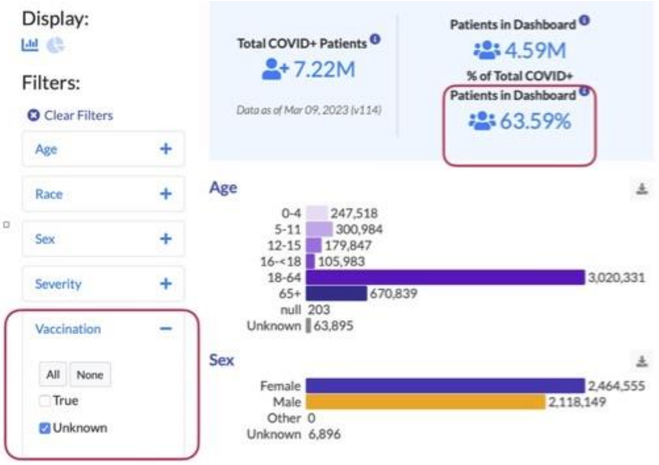

Here's the explanation N3C provides for EHRs inaccuracy:

> Vaccination data shown here is coming from EHR-recorded vaccination events at the sites which deposit data to N3C. If a patient was vaccinated at their local pharmacy, doctor's office, or state/federal vaccination site, that data will not be included here because these systems do not automatically link to a patient's EHR. Given that most vaccination events are not occurring at N3C sites, patients shown here as "Unknown" may be vaccinated, however, we do not have the records to verify this.

---

6 As of 3/9/23, 77 sites contributed data for 18.3 million persons

In contrast, our analysis of Public-Health-Ontario's reports of COVID-positive hospitalized patients over the period 1/1/22–5/31/22 suggests that under 10% of such patients (excluding those in the ICU) had unknown vaccination status.[7]

S4. Accounting for Ontarians Better Health

In this section, we will quantify the health difference between Ontarians and U.S. residents in such a way that the better health of Ontarians is more likely overstated than understated.

To account for Ontarians having better health, on average, than those in U.S., we will adjust downward, in two steps, the infection hospitalization rate H_r= 0.0022 we use in our modeling of COVID-19 hospitalizations in the U.S. for those 18–25 (general-population mix of males & females).

As discussed in the main exposition, obesity is a factor increasing the risk that a COVID-19 infected person will require hospitalization for COVID-19 treatment. In fact, Body Mass Index (BMI) is the single most potent modifier of COVID-19 risk for those under 60 years old [21, 22]. According to the CDC [23], "Having obesity may triple the risk of hospitalization due to a COVID-19 infection." We will assume this to be the case.

We will also assume that the health of Ontarians matches that of Canadians in general. The World Health Organization, in a Global Health Observatory data repository [24], reports the 2016 prevalence of obesity in the U.S. (BMI≥30) to be 36.2% (32.3, 40.1) and that in Canada to be 29.4% (25.7, 33.3). Citing more recent CDC and Statistics-Canada sources, Table 1 of [25] reports obesity prevalence to be 42.4% in the U.S. and 26.8% in Canada. We assume the latter measures of prevalence are accurate and that in the U.S. and Canada, 18–25 year-olds who are not obese share the same COVID infection-hospitalization rate r while $3r$ is the IHR for the obese. We have in the U.S.,

$$0.0022 \;=\; 0.576 \cdot r \;+\; 0.424 \cdot 3 \cdot r,$$

so that $r \;\approx 0.00119$. The IHR for 18–25 year-olds in Ontario would thus be

$$0.732 \cdot 0.00119 + 3 \cdot 0.00119 \cdot 0.268 \approx 0.00183.$$

Remark: our goal is to adjust the COVID-19 IHR for 18–25-year-old Ontarians downward by more than is justified in order to obtain evaluation-period COVID hospitalization rates (non-incidental) for the U.S. that are likely upper bounds for the actual rates. Because obesity rates decrease with age, the rate for the 18–25 age range is lower than 42.4% (U.S.) and 26.8% (Ontario). If we assume, based on CDC data, that obesity prevalence for the age range 18–25 in the U.S. is 20.5% and that for 18–25 year-olds in Ontario is 13% (≈ 26.8/42.4 · 20.5%*) and compute r as above, we obtain an IHR of 0.0021 for 18–25-year-old Ontarians.*

As detailed in Supplement S1, our IHR of 0.0022 for the 18–25 range is based on a model of Herrera-Esposito and de los Campos [9] yielding an IHR of 0.0037 for the 18–25 age range not accounting for incidental hospitalizations. We assumed an incidental hospitalization rate of 40% to obtain our estimate of $0.0022 \approx 0.6 \cdot 0.0037$. The Herrera-Esposito model is based on hospitalization and seroprevalence data for the ancestral strain of COVID-19. Studies suggest that the hospitalization risk of Omicron is a little less than that of the ancestral strain. E.g., a Johns Hopkins study [26] found a hospitalization hazard ratio for Omicron versus the ancestral strain of 0.94 while another study [27] based on data from the state of Washington found the hazard ratio to be 0.92. If we take the average of these two hazard ratios and apply it to our Ontario IHR 0.00183 from the preceding paragraph, we obtain an IHR for Ontario of $0.93 \cdot 0.00183 \approx 0.00170$. Even though this second reduction of the IHR 0.0022 used in our COVID hospitalizations modeling for the U.S. is not based on better health of Canadians, we assume the reduction from 0.0022 to 0.0017, one of about 23%, is based on better health, consistent with our intention to provide a better-health adjustment that is "more likely overstated than understated."

S5. Rise COVID-19 Infection Level in the United States and Ontario over the Evaluation Period 1/1/22–5/31/22

Seroprevalence data, presented below, suggests that over the evaluation period the rise in COVID-19 infection level in Ontario substantially exceeded that in the United States. Thus, to estimate U.S. COVID-19

---

[7] See reports provided at https://covid-19.ontario.ca/data/hospitalizations via the Wayback Machine. Subtract the number in the ICU who are COVID positive from the "total testing positive" in the Overview Section and compare to the total with known vaccination status "In hospital but not the ICU" (by adding the numbers to the right of the pie chart). For example, for the report "Last updated: April 23, 2022 at 10:31 a.m. (EST)," 1684 is the total testing positive 180 of whom were in the ICU, yielding 1504 testing positive "in hospital but not in the ICU." Adding the numbers with known vaccination status to the right of the pie chart "In hospital but not the ICU," we obtain 1368, indicating that 1504 – 1368 = 136 of the 1504 testing positive "in hospital but not the ICU" have unknown vaccination status; 136/1504 ≈ 0.09, so that about 9% have unknown vaccination status.

hospitalizations from those in Ontario we *should* proceed in two steps: (i) adjust Ontario rates downward based on an assumption of a smaller rise in infections over the evaluation period—a rise to equal that in the U.S., and then (ii) adjust the resulting lower rates upward by dividing by 0.77, implementing the better-health adjustment discussed in the preceding section. *We will make only the latter adjustment, aiming to obtain an upper-bound estimate of the hospitalizations-prevented benefit of mRNA-1273 vaccinating 1 million 18–25 year-olds in the U.S. immediately before the evaluation period* 1/1/22–5/31/22.

As we work with seroprevalence data, readers should keep the following from [28] in mind:

> [E]stimates of seroprevalence should not generally be interpreted as direct measures of cumulative infections over the course of a pandemic that has lasted more than 2 years. However, absolute increases in anti-N seroprevalence over short intervals (e.g., a few months) are likely to accurately represent increases in infections over the relevant interval.

U.S. Seroprevalence Data. For the United States, we estimate the increase in COVID anti-N seroprevalence[8] over the 5-month period January through May of 2022 via measuring its “absolute increase” from 1/1/22 to 2/15/22 and then from 2/15/22 through 5/31/22. Our age range of interest is 18–25. For the period 1/1/22 to 2/15/22, we rely on a CDC seroprevalence study [29] for which the age range 18–49 is closest to 18–25. For the period 2/15/22 through 5/31/22, we rely on a different CDC seroprevalence survey [30] for which the age range 18–29 is closest to 18–25.

As reported in [29],

> During December 2021–February 2022, overall U.S. seroprevalence increased from 33.5% (95% CI = 33.1–34.0) to 57.7% (95% CI = 57.1–58.3). Over the same period, … [s]eroprevalence increased from 36.5% (95% CI = 35.7–37.4) to 63.7% (95% CI = 62.5–64.8) among adults aged 18–49 years.

The preceding information for the age range 18–49 is reflected in the following figure from [29] in which the tick-marks on the horizontal axis should be interpreted as mid-month. We have added a vertical line representing Jan 1 and horizontal lines to aid in identifying seroprevalence levels, including that corresponding to Jan 1, approximately 42.5%. We see that the increase in COVID anti-N (infection-induced) seroprevalence over the period 1/1/22–2/15/22 is approximately (63.7 – 42.5)% = 21.2% for 18–49 year-olds.

FIGURE. Seroprevalence of infection-induced SARS-CoV-2 antibodies,* by age group — United States, September 2021–February 2022

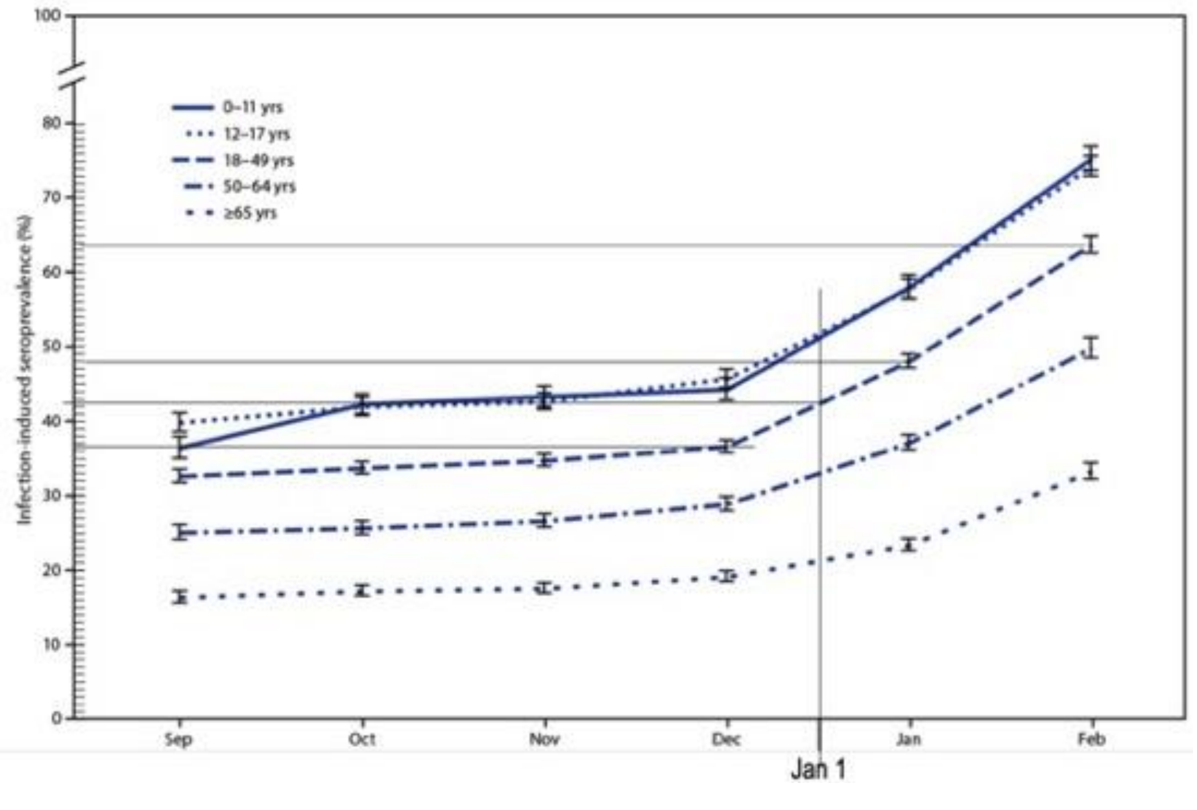

---

8 “anti-N” is short for antibodies against the SARS-Cov-2 nucleocapsid protein; such antibodies are produced in response to SARS-CoV-2 infection, not from SARS-Cov-2 vaccination (which elicits antibodies against the SARS-CoV-2 spike protein).

A different seroprevalence survey [30] whose results appear at the CDC webpage "2022 Nationwide COVID-19 Infection- and Vaccination-Induced Antibody Seroprevalence (Blood donations)" shows infection-induced seroprevalence in the 18–29 range increased from 60.7% (mid-February) to 72.1% (mid-May). Extending linearly the endpoint estimates 60.7% (Feb 15) and 72.1% (May 15) for the 96-day period from February 15 to May 15 to the end of May, 16 days after May 15, we obtain an approximation of seroprevalence for the 18–29 age group on 31 May 2022 of $74\% = \left(72.1 + \frac{72.1-60.7}{96} \cdot 16\right)\%$. We see that according to the CDC survey [30] infection-induced seroprevalence for the 18–29 age group in the U.S. increased from about 60.7% on 2/15/22 to about 74% on 5/31/22, a rise of 13.3%. We add our estimated rise (of 21.2%) from 1/1/22 to 2/15/22 to that from 2/15/22 to 5/31/22 to obtain an approximation of the rise in seroprevalence over the evaluation period of 34.5%, which we take as an approximation for the rise in infections over the 5-month evaluation period (January through May of 2022) for the 18–25 age group in the U.S.

Ontario Seroprevalence Data. The plots below from Figure 2 on page 5 and Figure 3 on page 6 of [28] suggest, respectively, that infection-acquired seropositivity in Ontario (all ages) rose about 40% over the evaluation period from about 9% on January 1st to about 49% on May 31st and likely increased by an even greater amount among the young, with Figure 3 suggesting a seroprevalence rise of about 53% in Canada over the evaluation period in the 17–24-year-old age-group.

Figure 2. Infection-induced seropositivity by province.

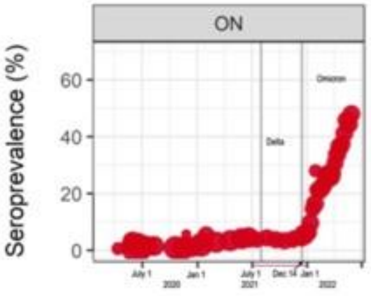

Figure 3. Infection-induced seropositivity by median age

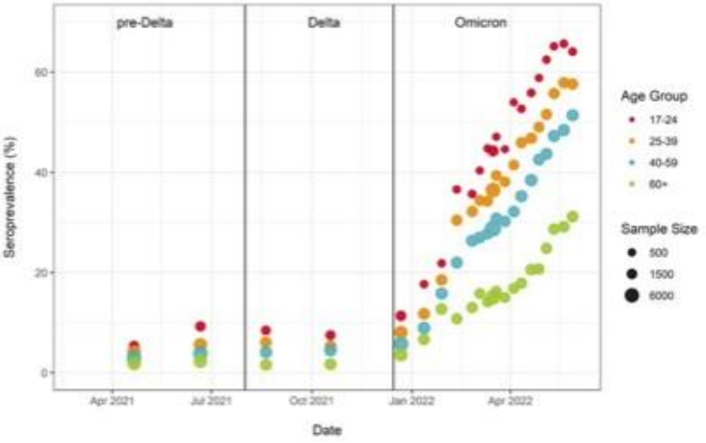

Two important observations relating to the preceding plots:
(i) The rise of about 40% in seropositivity over the evaluation period in Ontario is substantially greater than the 34.5% rise in seroprevalence, computed above, over the evaluation period for those 18–25 in the U.S. (Remark: the seroprevalence rise in the U.S., all ages, over the evaluation period is even lower—33.4%, based on the seroprevalence studies [29] and [30].) Moreover, as noted above, we would expect the rise in seroprevalence among Ontarians in age range 18–25 to exceed 40%.
(ii) The dip in seropositivity during the Delta-dominant period illustrates the point made in the reminder from [28] that "[E]stimates of seroprevalence should not generally be interpreted as direct measures of cumulative infections over the course of a pandemic that has lasted more than 2 years."

Because the rise in COVID N-antibody seroprevalence in Ontario over the evaluation period 1 January 2022 through 31 May 2022 is substantially greater than that in the U.S., the corresponding percentage rise in COVID-19 infections in Ontario should substantially exceed that in the U.S. As noted at the beginning of this section, we'd thus expect that over the evaluation period there would be more COVID hospitalizations per million population in Ontario than in the U.S. if COVID-hospitalization-risk profiles for 18–25 year-olds were identical in the two areas.

In the next section, we estimate the COVID hospitalization rate per million for 18-29 year-olds in Ontario over the evaluation period and adjust downward using the Herrera-Esposito model [9] to obtain an estimated rate for the 18–25 age group.

We remark that the greater rise in seroprevalence in Ontario vs. the U.S. over the evaluation period was likely at least partially due to there being a lower rate of prior COVID infection in Canada vs. the U.S. at the start of the

period. (The seroprevalence data of this section suggests a lower prior-infection rate 1/1/22 in Canada vs. the United States; see also Section S3 of Supplement S1 and Appendix S2 of this supplement.}

S6. COVID-19 Hospitalizations in Ontario, 1 January 2022 through 31 May 2022

We rely on the following hospitalizations data from Public Health Ontario:

Cumulative Hospitalizations "with COVID-19" in Ontario, ages 18–29, From 1/3/2022–6/5/2022			
Source	Through	Unvaccinated	Fully Vaccinated (no booster)
Table 1b of (i)	1/3/2022	1,024	39
Table 1a of (ii)	1/16/2022	1,060	87
Table 3a of (iii)	1/30/22	1,094	137
Table 3a of (iv)	2/13/22	1,114	155
Table 3a of (v)	2/27/22	1,124	166
Table 3 of (vi)	3/13/22	1,130	175
Table 3 of (vii)	3/27/22	1,136	187
Table 3 of (viii)	4/10/22	1,147	205
Table 3 of (ix)	4/24/22	1,158	221
Table 3 of (x)	5/8/22	1,165	234
Table 3 of (xi)	5/22/2022	1,175	245
Table 3 of (xii)	6/5/2022	1,175	254

(i) "Confirmed Cases of COVID-19 Following Vaccination in Ontario: December 14, 2020 to January 3, 2022,"
(ii) "Confirmed Cases of COVID-19 Following Vaccination in Ontario: December 14, 2020 to January 16, 2022,"
(iii) "Confirmed Cases of COVID-19 Following Vaccination in Ontario: December 14, 2020 to January 30, 2022,"
(iv) "Confirmed Cases of COVID-19 Following Vaccination in Ontario: December 14, 2020 to February 13, 2022,"
(v) "Confirmed Cases of COVID-19 Following Vaccination in Ontario: December 14, 2020 to February 27, 2022,"
(vi) "Confirmed Cases of COVID-19 Following Vaccination in Ontario: December 14, 2020 to March 13, 2022,"
(vii) "Confirmed Cases of COVID-19 Following Vaccination in Ontario: December 14, 2020 to March 27, 2022,"
(viii) "Confirmed Cases of COVID-19 Following Vaccination in Ontario: December 14, 2020 to April 10, 2022,"
(ix) "Confirmed Cases of COVID-19 Following Vaccination in Ontario: December 14, 2020 to April 24, 2022,"
(x) "Confirmed Cases of COVID-19 Following Vaccination in Ontario: December 14, 2020 to May 8, 2022,"
(xi) "Confirmed Cases of COVID-19 Following Vaccination in Ontario: December 14, 2020 to May 23, 2022," and
(xii) "Confirmed Cases of COVID-19 Following Vaccination in Ontario: December 14, 2020 to June 5, 2022."

Remark: (i)–(xi) are available at Public Health Ontario's "Archive of Severe Outcomes among Confirmed Cases of COVID-19 Following Vaccination in Ontario" that omits links to a few reports such as that for (xii). The tables from (i), (iii), (xi), and (xii) from which we have drawn our data appear in Appendix SI.

From the preceding table, we find that the number of reported COVID hospitalizations among the unvaccinated of ages 18–29 in Ontario from 1/4/22 through 5/22/22 is 151 = 1,175 – 1,024, and among the much larger number of the fully vaccinated, 206 = 245 – 39. Also note that the table above suggests that from 1/4/22 through 1/30/22 there were 70 = 1,094-1,024 hospitalizations among the unvaccinated and 98= 137-39 hospitalizations among the fully vaccinated. However Table 3b of "Confirmed Cases of COVID-19 Following Vaccination in Ontario: December 14, 2020 to January 30, 2022" reports that from 1/1/22 through 1/30/22 there were 68 hospitalizations among the unvaccinated and 96 among the vaccinated, while we would expect these counts would be 70 or greater for the unvaccinated and 98 or greater for the fully vaccinated. At any rate, it seems reasonable to assume there were very few hospitalizations of the unvaccinated or fully vaccinated from 1/1/22 through 1/3/22. Thus, we will assume that for those 18-29 years old, 1,024 well approximates cumulative hospitalizations among the unvaccinated through 12/31/21 and 39 well approximates cumulative hospitalizations among the vaccinated through 12/31/21. Finally, because the number of hospitalizations among the unvaccinated on 6/5/2022 is the same as that on 5/22/22, we assert 151 = 1,175 – 1,024 well approximates the number of COVID hospitalizations among the unvaccinated 18–29 over the evaluation period.

Note that the table above indicates there were 9 hospitalizations from 5/23/22 through 6/5/22 among the fully vaccinated. Because the fraction of the 14-day period from 5/23/22 through 6/5/22 belonging to May is $\frac{9}{14}$, we will assume that $9/14 \cdot 9 \approx 6$ of the hospitalizations occurring from 5/23/22 through 6/5/22 occurred on or before 5/31/22, bringing the cumulative count of hospitalizations among the fully vaccinated

18–29 year-olds in Ontario to 251 as of 5/31/22. We obtain 251 - 39 = 212 as an approximation of the number of COVID hospitalizations among the fully vaccinated 18–29 over the evaluation period.

The hospitalization counts discussed above include a substantial number of incidental COVID admissions (with COVID, not for COVID). In the table S2.3 below, we approximate the number of for-COVID hospitalizations using incidental-hospitalization data from [31] as well as the with-COVID hospitalization counts tabulated above. Incidental-hospitalizations data provided by Public Health Ontario is not stratified by sex, age, or vaccination status. In this section, our primary goal is to obtain estimates of *for-COVID* hospitalizations per million 18–29-year-old Ontarians (general population mix of males and females) stratified by vaccination status.[9] To accomplish this, we assume incidental rates are essentially uniform over all age ranges (even though there is evidence that incidental hospitalization rates are higher among the young—see Section S2 and Appendix S1 of Supplement S1); we also assume incidental rates are similar among the vaccinated and unvaccinated. There is evidence that incidental rates may be higher among the unvaccinated: a study [32] of 151 adult Omicron patients found that those patients *hospitalized primarily for COVID* had "*higher COVID-19 vaccination rates* compared with patients with incidental COVID-19".[10] For the 55 patients in the study known to have completed the primary vaccination series, 37 were deemed hospitalized primarily for COVID, while 7 had COVID as a factor contributing to admission, and 11 (20%) were deemed incidental [32, Table 2]. Moreover, if we assume that Ontario would also have classified as incidental the 7 patients for whom COVID was considered a contributing factor to hospitalization, then we arrive at an incidental rate for the vaccinated of 32%, well below incidental rates provided by Public Health Ontario (see below) for all hospitalized patients—unvaccinated and vaccinated. Another factor supporting our assumption that incidental rates are not higher among the vaccinated is that our assumption of equivalent incidental rates yields Omicron-caused hospitalization rates for the evaluation period of 264 per million unvaccinated 18–29 year-olds and 84 per million vaccinated 18–29 year-olds. This suggests a vaccination effectiveness against hospitalization (VEH) of 68.2%, which exceeds the estimate 60.9% of mRNA-vaccine effectiveness against Omicron in the meta-analysis [33] and is consistent with Omicron VEH estimates for BNT162b2 of 73.2% (14–174 days postdose 2) and 65.1% (175+ days after dose 2) [34, Table S13], where these VEH estimates require strong evidence that COVID is the primary reason for hospitalization (length of stay ≥ two days, respiratory coded). We now adjust our evaluation-period hospitalization counts using incidental-hospitalization data (from [31]).

Table S2.3.

Hospitalizations for COVID-19 in Ontario, ages 18–29, From 1/1/2022–5/31/2022					
Period	Average percentage of "for COVID" hospitalizations during period*	With-COVID hospitalizations among the unvaccinated during period	For-COVID hospitalizations among the unvaccinated during period	With-COVID hospitalizations among the vaccinated during period	For-COVID hospitalizations among the vaccinated during period
1/1-1/16	53.30	36	19.19	48	25.58
1/17-1/30	55.25	34	18.79	50	27.63
1/31-2/13	55.11	20	11.02	18	9.92
2/14-2/27	48.97	10	4.90	11	5.39
2/28-3/14	45.58	6	2.73	9	4.10
3/15-3/27	46.78	6	2.81	12	5.61
3/28-4/10	46.60	11	5.13	18	8.39
4/11-4/24	45.14	11	4.97	16	7.22
4/25-5/8	42.33	7	2.96	13	5.50
5/8-5/22	39.26	10	3.93	11	4.32
5/23-5/31	40.93	0	0	6	2.46
1/1-5/31	47.21**	151	76	212	106

* from [31]; ** The average of the averages in column 2.

[9] We then adjust these rates downward to approximate rates for 18–25 year-olds using the Herrera-Esposito model.

[10] Observe that higher incidental rates among the unvaccinated will decrease the number of evaluation-period for-COVID hospitalizations among the unvaccinated and decrease the hospitalizations benefit of vaccination.

We need to use Ontario population and vaccination data to convert these hospitalization counts over the evaluation period, 76 for the unvaccinated and 106 for the fully vaccinated (no boosters), into expected hospitalizations per million, over the evaluation period.

Recall from the main exposition that U represents the projected the number of Omicron-caused hospitalizations in a test population of 1 million representative unvaccinated males 18–25, the fraction of whom have had a prior COVID-19 infection at the beginning of the five-month evaluation period (January–May 2022) is F_{pi}. To test U using data from Ontario, we replace the hypothetical test population with a real-world test population consisting of unvaccinated persons in Ontario ages 18–29. We choose this age range because it's one for which Ontario provides hospitalization data and has significant overlap with the range 18–25 of interest. Because Ontario age-stratified data is not sex specific, our real-world test population consists of males and females. This real-world test population varies in size from about 319,418 on 1 January 2022 to about 277,532 on 31 May 2022. In addition, some persons 29 years of age at the beginning of the evaluation period leave the test population during the period by turning 30 and some enter by turning 18. The preceding estimates of numbers of unvaccinated in Ontario over time are found in a spreadsheet "COVID-19 Vaccine data by age" available at a government of Ontario website [35] and are obtained from spreadsheet data by subtracting, from the "total population" of Ontarians 18–29 in column G, the number having received at least one dose by a given date in column C.

Here is a graph based on the data in the vaccine-data-by-age spreadsheet [35], along with a portion of the data:

Table S2.4.

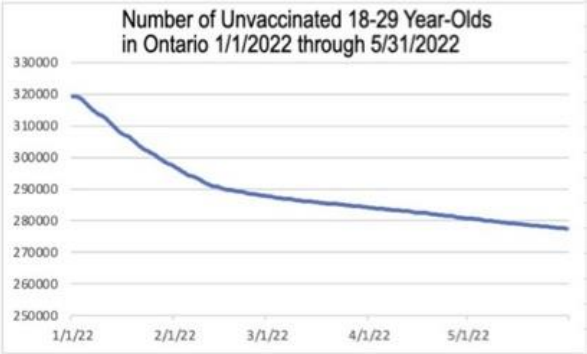

1/1/22	319418		5/18/22	278792
1/2/22	319335		5/19/22	278695
1/3/22	318964		5/20/22	278559
1/4/22	318226		5/21/22	278430
1/5/22	317253		5/22/22	278352
1/6/22	316306		5/23/22	278322
1/7/22	315297	•••	5/24/22	278300
1/8/22	314351		5/25/22	278162
1/9/22	313666		5/26/22	278063
1/10/22	313180		5/27/22	277922
1/11/22	312388		5/28/22	277802
1/12/22	311411		5/29/22	277702
1/13/22	310366		5/30/22	277649
1/14/22	309204		5/31/22	277532

If the number of unvaccinated had remained at a constant level of say 277,532 (May 31 level) throughout the period 1/1/22–5/31/2022, then the for-COVID hospitalization rate per million for unvaccinated 18–29 year-olds would be $76/0.277532 \approx 274$ per million. However, the actual rate is smaller because, during, say, the period 1/1/22 through 1/14/22, at a minimum, 309,204 unvaccinated 18–29 year-olds were exposed to the risk of possible COVID-19 hospitalization (not just the 277,532 mentioned earlier). To account for the decreasing level of unvaccinated persons over the period in question, we express the hospitalization rate in terms of hospitalizations per 100,000 person days at risk (which will facilitate comparison with data Public Health Ontario provides). Based on Table S2.4 above, we can see that on 5/31/2022 a total of 277,532 unvaccinated 18–29 year-olds were exposed to one day (May 31) of hospitalization risk: 277,532 person-days of risk. On 5/30/2022, we see that 277,649 were exposed to 1 day of risk, so that the 30th and 31st of May account for 277,549 + 277,532 person-days of risk. Adding all of numbers of unvaccinated persons on each of the days from 1/1/2022 through 5/31/2022 (and dividing by 100,000), we obtain $76/437.04401 \approx 0.1739$ hospitalizations per 100,000 person-days as the for-COVID hospitalization rate for unvaccinated 18–29 year-olds in Ontario for the period 1 January 2022 through 31 May 2022. (The with-COVID rate stands at 151/437.04401 ≈ 0.3455.)

One million unvaccinated persons over the course of 5 months, 152 days, would be exposed to 1 million times 152 days = 152,000,000 person days of risk. With the rate of 0.1739 hospitalizations per 100,000 person days, Ontario's for-COVID hospitalization rate for unvaccinated 18–29 year-olds is $0.1739 \cdot 1520 \approx 264$ hospitalizations (for COVID) per million population.

We remark that in some of its enhanced epidemiological summaries, Public Health Ontario provides with-COVID hospitalization rates in terms of 100,000 person-days. For example, Table 1 of "Confirmed Cases of COVID-19 Following Vaccination in Ontario: December 14, 2020 to January 30, 2022"[11] provides a rate of 0.57 per 100,000 person-days for unvaccinated 18–29 year-olds for the 30-day period ending January 30, 2022 and a rate of 0.18 per 100,000 person days for vaccinated (unboosted) 18–29 year-olds. We have computed above 0.3455 as the corresponding unvaccinated rate for the entire evaluation period 1/1/22–5/31/22, and below we find the corresponding vaccinated rate for the entire evaluation period to be 0.1107.

We now analyze for-COVID hospitalizations over the period 1 January 2022 through 31 May 2022, of those 18–29 year-olds in Ontario who had completed the primary vaccination series (with no booster doses). We are assuming there were 106 such hospitalizations among this group of Ontarians. Here is a plot, based on data from the spreadsheet [35] cited earlier of the number of those 18–29 who had primary-vaccination protection by the given date, along with some of the data used to produce the plot.[12]

Table S2.5.

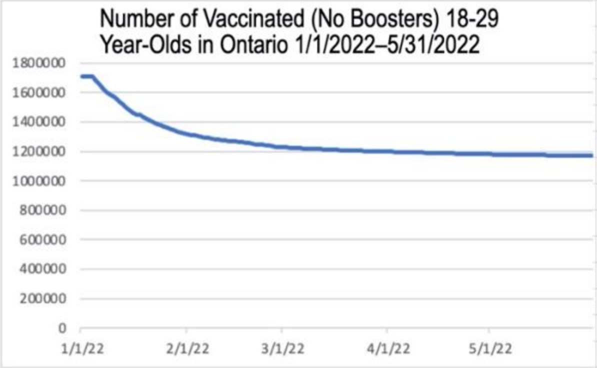

1/1/22	1708464
1/2/22	1708587
1/3/22	1709122
1/4/22	1710072
1/5/22	1679699
1/6/22	1655257
1/7/22	1630208
1/8/22	1605907
1/9/22	1587776
1/10/22	1573775
1/11/22	1556115
1/12/22	1537051
1/13/22	1517036
1/14/22	1497523

•••

5/18/22	1174184
5/19/22	1173892
5/20/22	1173583
5/21/22	1173262
5/22/22	1173056
5/23/22	1172924
5/24/22	1172848
5/25/22	1172549
5/26/22	1172307
5/27/22	1172035
5/28/22	1171763
5/29/22	1171510
5/30/22	1171391
5/31/22	1171138

Adding the reported numbers of the fully vaccinated for 1/1/2022 through 5/31/2022, we obtain the total number of person-days at risk of COVID-19 hospitalization for those 18–29 having completed the primary vaccination series (but unboosted): 191,578,037. Thus, the rate of for-COVID hospitalizations per 100,000 person-days, for fully vaccinated Ontarians 18–29 years old, is $106/1915.78037 \approx 0.05533$. (The with-COVID rate stands at $212/1915.78037 \approx 0.1107$.) Hence, in a group of 1 million fully vaccinated (but not boosted) 18-29 year-olds at risk of for-COVID hospitalization for 5 months, 152 days, we would expect $1520 \cdot 0.05533 \approx 84$ hospitalizations.

We conclude that over the five-month period 1 January 2022 through 31 May 2022, full vaccination provided Ontarians 18–29 an approximate hospitalizations-prevented benefit of 264 – 84 = 180 per million; that is, the full vaccination of one million persons in the age range prevented about 180 COVID-19 hospitalizations. If incidental COVID-19 hospitalizations are more common among the young, then 180 is likely an overestimate of the hospitalizations-prevented benefit.

Recall our goal is to evaluate COVID-19 hospitalizations for the age range 18–25, not 18–29. As we have discussed, the model of Herrera-Esposito and de los Campos [9] suggests an IHR of 0.37% for the age range 18–25 (including incidental hospitalizations). For the age range 18–29, it suggests 0.439%:

$$\frac{1}{12}\int_{18}^{30} M(t)\ dt \approx 0.439\%,$$

where $M(t) = 0.07014\, e^{0.0750t}$ approximates Herrera and de los Campos's model—see Supplement S1 for details. Because 0.37 is about 15.7% less than 0.439, we might adjust our hospitalizations data from the age range 18–29 to the range 18–25 as follows:

[11] See Appendix S1 for a copy of the table.

[12] The spreadsheet [35] fails to record numbers of boosters administered (in any age range) for 1/1/2022 through 1/4/2022. For this range of dates, we have used 329,050 as an approximation of the number of boosters given to those 18–29, which is the number reported for 1/1/2022 in a spreadsheet [36] providing weekly information. E.g., the number of fully vaccinated but not boosted on 1/1/2022 is approximately 2,037,514-329,050 = 1,708,464, where 2,037,514 is from [35] (and 329,050 is from [36]).

*Table S2.6: Estimated Hospitalizations-Prevented Benefit of Full Vaccination, Ontario, 1/1/22–5/31/22**

Age Range	COVID Hospitalizations Per Million Unvaccinated Persons	COVID Hospitalizations Per Million Vaccinated, Unboosted Persons	Hospitalizations Prevented Benefit of Full Vaccination Per Million Persons
18–29	264	84	180
18–25	223	71	152

*264 and 84 are rates computed directly from Ontario data while 223 and 71 result from decreasing the rates for the age range 18–29 by a factor of 0.843, suggested by the IHR model of Herrera-Esposito and de los Campos [9] to obtain corresponding rates for the age range 18–25.

S7. COVID-19 Hospitalizations in the United States 1 January 2022 through 31 May 2022

We seek to obtain estimates for U.S. COVID-19 hospitalizations over the evaluation period based on those in Ontario. We will assume that incidental COVID-19 hospitalization rates in Ontario are similar to those in the U.S.[13] In order to more likely overstate than understate the COVID-19 hospitalizations-prevented benefit for 18–25 year-olds in the U.S., we will ignore the seroprevalence-based evidence presented in Section S5 that the COVID-19 infection-level increase in Ontario over the evaluation period substantially exceeded that in the U.S. Rather, we will assume that the infection-rise was comparable so that hospitalizations per million would be approximately the same in the U.S. and in Ontario were health-related hospitalization-risks equivalent. As we discussed in Section S4, health-related risks are not comparable—they are higher in the U.S. and we adjust Ontario rates upward to account for this.

Recall that our computation in Section S4 (more likely to overestimate the better health of Ontarians vs. U.S. residents than to underestimate it) yields a COVID infection-hospitalization rate for Ontarians 18–25 that is 77% of that in the U.S. Thus, we might anticipate the hospitalizations data in Table S2.6 of the preceding section would represent about 77% of corresponding values for the U.S. In Table S2.7 below, we record these projected U.S. hospitalizations, and, in the 3rd column, we estimate parenthetically a reduction in vaccinated hospitalizations resulting from an assumption that all 1 million vaccinated persons were 14 days past their second dose on 1 January 2022.

*Table S2.7: Estimated Hospitalizations-Prevented Benefit for Full Vaccination, USA, 1 January 2022-31 May 2022 Assuming a COVID-Infection-Level Increase in Ontario Approximately Equal to that in the U.S.**

Age Range	COVID Hospitalizations Per Million Unvaccinated Persons	COVID Hospitalizations Per Million Vaccinated, Unboosted Persons (Assuming Recent Vaccination)	Hospitalizations Prevented Benefit of full vaccination per million persons (Assuming Recent Vaccination)
18–29	343	109 (89)	234 (254)
18–25	290	92 (76)	198 (214)

* The larger rise in infection level in Ontario vs. the U.S. over the evaluation period (discussed in Section 5) suggests the predicted hospitalization counts are more likely overestimates than underestimates

To obtain the third-column parenthetical rates of hospitalizations per million vaccinated persons, we assume that if the million had been fully vaccinated as of 1/1/2022, vaccine effectiveness would have been 77% against hospitalization instead of 72%. For example, with 109 hospitalizations based on 72% VEH (relative to the unvaccinated), we'd expect $109/0.28 \approx 389$ hospitalizations among the unvaccinated, and if VEH were 77%, we'd expect $389 \cdot (1 - 0.77) \approx 89$ hospitalizations for among those "recently vaccinated."

We have arrived at an estimate that fully COVID vaccinating (with all vaccinations 14 days past dose 2 on 1/1/22) a representative group of 1 million 18–25 year-olds (males and females) in the U.S would prevent approximately 214 COVID-hospitalizations over the evaluation period. We would expect considerably fewer than 214 hospitalizations if the vaccinated group consisted of males only ([10, Table 2], [11, Table 1).

[13] The incidental rates from Ontario influencing our COVID-hospitalization projections for the U.S. are, roughly 50% on average (see Table S2.3 above). There is evidence that among the young in the U.S., the incidental rate exceeds 50%. In the final paragraph of the Appendix S1 to Supplement S1, we estimate an incidental rate of 72.2% for 15–24 year-olds in Connecticut for calendar year 2020. Figure 2 of an Epic Health study suggests that for 0–18 year-olds in the U.S. an incidental rate typically well above 50% during the study period 3/1/2020–1/31/22 and for those 19–64 an incidental rate above 40% for 1/1/2022–1/31/2022.

Recall that in its most likely scenario the FDA projected 6619 COVID hospitalizations would occur among 1 million unvaccinated 18–25 year-old males in the U.S. during the evaluation period 1 January 2022 through 31 May 2022 [12]. Given the evidence in [10] and [11] that females 18–25 are hospitalized for COVID at higher rates than males, the FDA's projection of 6619 would be higher for a mixed population of unvaccinated males and females. We have presented evidence in Table S2.7 above that the actual hospitalization rate (for COVID not with COVID) among the unvaccinated 18–25 year-olds in the U.S. (males and females) over the evaluation period is approximately 290 per million, likely an overestimate because we are ignoring evidence that more COVID infections occurred in Ontario over the evaluation period than in the U.S. In addition, we would expect a lower rate of hospitalizations per million males 18–25.

Recall that in its mostly likely scenario, the FDA projected that 4,766 hospitalizations would be prevented through the full mRNA-1273 vaccination of one million 18–25-year-old males during the evaluation period 1 January 2022 through 31 May 2022 [10]. Real-world data from Ontario contributing to Table S2.7 above suggests the actual hospitalizations-prevented benefit of mRNA-1273 vaccinating 1 million 18-25-year-old males to be at most 214 per million ("at most" because *we have ignored the greater rise in COVID-infections in Ontario over the evaluation period than in the U.S. and 214 represents hospitalizations of a mixed population of males and females and females are likely hospitalized at higher rates); finally, we have also ignored evidence that incidental rates are likely higher among the young. Thus, in its most likely scenario, our work suggests that FDA's projected number of evaluation-period COVID hospitalizations prevented by mRNA-1273 vaccinating 1 million 18–25-year-old males is at least 22 (*$4766/214 = 22.27$*) times the actual number of COVID hospitalizations (non-incidental) prevented.*

This overestimation resulted from the FDA's (i) not accounting for prior-infection protection, (ii) not accounting for the high rates of incidental COVID-19 hospitalizations in the U.S. (which we have assumed to be comparable to those in Ontario), (iii) assuming 18–25 year-olds are hospitalized for COVID at the same rate as 26–35 and 36–45 year-olds, and (iv) in its most likely scenario, essentially assuming that every unvaccinated person would be infected over the 5-month evaluation period. The following table compares our estimated actual evaluation-period hospitalizations-prevented benefit of mRNA-1273 vaccinating 1 million 18–25-year-old males to the FDA's estimates from each of its Omicron-based scenarios:

Table S2.8: The FDA's Estimates of the Evaluation-Period Hospitalizations-Prevented Benefit of mRNA-Vaccination of 18–25 Year-Old Males in the U.S. Compared to the Estimated Actual Benefit.

Scenario	1 "Most Likely" High Infection Level $I_r = 1$	2 Moderate Infection Level $I_r = 0.356$	3 Low Infection Level $I_r = 0.052$	4 Delta Dominant High Infection Level $I_r = 1$	5 High Infection Level $I_r = 1$	6 High Infection Level $I_r = 1$
FDA's projected hospitalizations-prevented benefit per million vaccinated	4,766	2,088	635	5,957	4,766	4,766
Factor by which the benefit differs from the benefit of 214 from Table S2.7 derived from Ontario data	22.3	9.8	3.0	N/A	22.3	22.3

Remarks: (i) In all scenarios, except the 4th, Omicron is assumed to be dominant and (ii) the factors in row 3 are likely lower bounds because we have assumed the COVID infection-level increase in Ontario over the evaluation to be approximately equal to that in the U.S. and the benefit of 214 per million vaccinated from Table S2.7 is for a mixed population of males and females and would likely be lower were the million all males.

In contrast our model's scenarios—modified in Section S2 above to reflect the assumption that there are no significant sex-based differences in COVID infection and hospitalization rates—produce estimates closer to the estimated actual benefit.

Table S2.9: Our Model's Estimates of the Evaluation-Period Hospitalizations-Prevented Benefit of mRNA-Vaccination of 18–25 Year-Old Males in the U.S. Over the Evaluation Period Compared to the Estimated Actual Benefit, Assuming No Sex-Based Differences in COVID-Hospitalization Risk (See Table S2.2).

Scenario	A<br>High Infection Level<br>$I_r = 1$<br>$F_{pi} = 0.65$	B<br>Moderate Infection Level<br>$I_r = 0.356$<br>$F_{pi} = 0.65$	C<br>Low Infection Level<br>$I_r = 0.052$<br>$F_{pi} = 0.65$	D<br>Most Likely<br>$I_r = 0.456$ (twice that of 2nd COVID wave in U.S.)<br>$F_{pi} = 0.76$	E<br>Equal Protection: Prior Infection & Vaccination<br>$I_r = 0.456$<br>$F_{pi} = 0.76$
Our Model's projected hospitalizations-prevented benefit per million vaccinated	683	243	36	234	322
Factor by which the benefit differs from the benefit of 214 from Table S2.7	3.2	1.1	0.17	1.09	1.50

S8. Testing Our Modeling Functions U and V

We show that there are inputs consistent with evaluation-period data such that our modeling functions U and V yield outputs exactly matching Ontario-hospitalizations data from Table S2.6 above. Our strategy is to identify plausible ranges of values of key-input variables, and then to use the computer-algebra system *Mathematica* to find input-values lying in the identified plausible ranges such that with these *Mathematica*-generated input-values our functions U and V output, respectively, the hospitalization values 223 and 71 from Table S2.6.

We make a number of simplifying assumptions that might be construed as over-simplifying. However, our process fits our model perfectly to COVID-hospitalization data from Ontario with input values consistent with infection and vaccination data from Ontario, as well as effectiveness data for protection provided by prior-infection or vaccination (or both).

We begin by using currently available data pertaining to the evaluation period to derive reasonable ranges for the key variables that determine the values of U and V, the key variables being I_r, ${}_uF_{pi}$, ${}_vF_{pi}$,E_{pi}, E_v, E_h, and E_b, where

- I_r is the fraction of the infection-naïve unvaccinated population of 18–25-year-old Ontarians that becomes infected with COVID-19 over the evaluation period 1/1/22–5/31/22,
- ${}_uF_{pi}$ is the fraction of a representative population of unvaccinated 18–25-year-old Ontarians having had a COVID-19 infection before 1 January 2022,
- ${}_vF_{pi}$ is the fraction of a representative population of vaccinated 18–25-year-old Ontarians having had a COVID-19 infection before 1 January 2022,
- E_{pi} is the effectiveness of prior-infection protection against re-infection (symptomatic and asymptomatic) for 18–25-year-old Ontarians over the evaluation period,
- E_v is the effectiveness of primary -series vaccination (no boosters) against all infection (symptomatic and asymptomatic) for 18–25-year-old Ontarians over the evaluation period,
- E_h is the effectiveness of hybrid protection against re-infection (symptomatic and asymptomatic) for 18–25-year-old Ontarians over the evaluation period, and
- E_b is the effectiveness of primary-series vaccination and one booster against all infection (symptomatic and asymptomatic) for 18–25-year-old Ontarians over the evaluation period.

Another key variable is the Omicron infection-hospitalization rate H_r for 18–25-year-old Ontarians during the evaluation period. In Section S3 above, we derived an estimate that over the evaluation period we might expect H_r to be approximately 0.0017. However, this estimate was derived assuming an incidental COVID-19 hospitalization rate of 40%, but we have seen that, on average, over the evaluation period, this incidental rate is about 52.8%. Hence, we will assume that $H_r \approx 0.0017 \cdot 0.472/0.6 \approx 0.0013$. If the incidental rate

is higher (as it may be for the 18–25 age group), then H_r should be lower. As we will show, with H_r = 0.0013 or H_r = 0.0012, *Mathematica's* "`NMinimize`" command cannot find values of other key input variables, in ranges suggested by real-world data, such that U and V output exactly the data in Table S2.6 for the 18–25-year-old age-group. However, when H_r = 0.0011, *Mathematica* can fit our model perfectly to the data with plausible values for all other inputs. With H_r = 0.0010, *Mathematica*'s input values yielding a perfect modeling fit are even more plausible in that most values are closer to the middle of their plausible ranges.

Plausible Ranges for Key Variables (Highlighted). In Appendix S2, we establish that a plausible range of values for ${}_uF_{pi}$ for an Ontario test population of 18–29 year-olds is $0.61 \le {}_uF_{pi} \le 0.73$. We assume ${}_vF_{pi} \le {}_uF_{pi}$, and we assume that ${}_uF_{pi}$ for the age range 18–29 well approximates ${}_uF_{pi}$ for 18–25 year-olds.

Based on our discussion of (iii) in Section S1 above, we assume $0.01 \le E_v \le 0.13$ and $0.26 \le E_b \le 0.48$.

As for the key variables $E_{pi}$and E_h, we consider a finding of a population-based cohort study [37], which monitored all persons previously infected with SARS-CoV-2 for reinfection during the Omicron wave in Iceland, from December 1, 2021 (first diagnosed case of Omicron in Iceland) to the end of the study period on February 13, 2022. The study [37] found, "Surprisingly, 2 or more doses of vaccine were associated with a slightly higher probability of reinfection compared with 1 dose or less." Similarly, a study [38], based on early Omicron cases among previously infected employees of the Cleveland Clinic, found that employees receiving two or three doses of an mRNA vaccine following prior COVID-19 were at a higher risk of reinfection than those receiving only a single dose. Based on the studies [37] and [38] and to simplify our modeling by reducing the number of key input variables, we assume that (i) $E_{pi} = E_h$, that (ii) the common value of E_{pi} *and* E_h also provides the effectiveness of prior infection in preventing reinfection for a person who has received a primary series vaccination plus one booster, and that (iii) $0.3 \le E_{pi} = E_h \le 0.57$.

Expressing the key variable I_r in terms of other key variables. Based on the Government of Ontario's vaccine-data-by-age spreadsheet [35], we see that the percentage of Ontarians, 18–29 years old, having received at lease one dose of a COVID-19 vaccine by 1/1/22 stands at 86.99%, reaching 88.0% by 2/5/22 and 88.69 by 5/31/22. The average of all one-dose percentages for the evaluation period stands at about 88%, which we'll take as an approximation for the vaccinated percentage of the 18–25-year-old population in Ontario over the evaluation period. The spreadsheet [35] also shows that the percentage of 18–29-year-old Ontarians having completed a primary series plus booster dose by 1/1/22 stands at 13.4%, reaching 31.4% by 2/5/22 and 37.6% by 5/31/22. We will take the average boosted percentage for 18–29 year-olds during the period, about 33%, as an approximation for the boosted percentage of the 18–25-year-old population in Ontario over the evaluation period. Assuming that 33% of the 18–25 population is boosted over the evaluation period and 88% is vaccinated implies that, on average, 55% are vaccinated, most with two doses (only) but some with one. We assume that for the vaccinated (one or two doses, no prior infection), the effectiveness of vaccine protection against infection is E_v.

Let N be the number of 18–25 year-olds in Ontario over the evaluation period, which we assume to be essentially constant and let ΔI be the fraction of this population to be COVID-19 infected over the evaluation period (which will assume to be 0.40 based on seroprevalence data discussed in Section S4). Using the notation for key variables described above and making the additional simplifying assumption that the probability a vaccinated 18–25 year-old has had a prior COVID infection is independent of the number of vaccine doses received, we can express the number $\Delta I \cdot N$ of COVID-infections in the 18–25 year-old population in Ontario over the evaluation period as follows:

$$\Delta I \cdot N = 0.12 \cdot N \cdot \left(1 - {}_uF_{pi}\right) \cdot I_r + 0.12 \cdot N \cdot {}_uF_{pi} \cdot I_r \cdot \left(1 - E_{pi}\right) + 0.55 \cdot N \cdot \left(1 - {}_vF_{pi}\right) \cdot I_r \cdot (1 - E_v)$$
$$+0.55 \cdot N \cdot {}_vF_{pi} \cdot I_r \cdot \left(1 - E_{pi}\right) \cdot N + 0.33 \cdot N \cdot \left(1 - {}_vF_{pi}\right) \cdot I_r \cdot (1 - E_b) + 0.33 \cdot N \cdot {}_vF_{pi} \cdot I_r \cdot \left(1 - E_{pi}\right),$$

where the first summand on the right is the number of infections among the infection-naïve unvaccinated, the second is the number (of infections) among the unvaccinated having prior infection, the third is the number among the infection-naïve vaccinated, the fourth is the number among the vaccinated having prior infection, the fifth the number among the infection-naïve boosted, and the sixth is the number among the

boosted having prior infection. Canceling N from both sides of the preceding equation, letting ΔI = 0.4, ${}_uF_{pi} = x$, ${}_vF_{pi} = y$, $E_{pi} = E_h = p$, $E_v = v$, $E_b = b$, and then solving for I_r yields

$$I_r = \frac{0.40}{0.12(1-x) + 0.12x(1-p) + 0.55(1-y)(1-v) + 0.55y(1-p) + 0.33(1-y)(1-b) + 0.33y(1-p)}.$$

Fitting our Model to Ontario Hospitalization Data From Table S2.6 (for those 18–25 years old): With the function I_r defined above and variable labels x, y, p, v, and b introduced in the preceding subsection, as well as the hospitalization-risk reduction values $HRR_{pi} = 0.79$, HRR_v= 0.67, $HRR_h = 0.94$ (where the first two values are as in our Scenarios A-E and the latter value was derived in (iv) of Section S1 of this supplement), our hospitalizations functions U and V are given by

$$U(H_r, x, y, p, v, b) = (1\text{m}) \cdot H_r \cdot I_r(x, y, p, v, b) \cdot \big((1-x) + x \cdot (1-p) \cdot (1-0.79)\big);$$

$$V(H_r, x, y, p, v, b) = (1\text{m}) \cdot H_r \cdot I_r(x, y, p, v, b) \cdot \big((1-y) \cdot (1-v) \cdot (1-0.67) + y \cdot (1-p) \cdot (1-0.94)\big).$$

We seek values of the variables $H_r, x, y, p, v,$ and b that will minimize the function

$$F(H_r, x, y, p, v, b) = (U(H_r, x, y, p, v, b) - 223)^2 + (V(H_r, x, y, p, v, b) - 71)^2$$

subject to the constraints $0.0010 \leq H_r \leq 0.0013$, $0.61 \leq x \leq 0.73$, $y \leq x$, $0.3 \leq p \leq 0.57$, $0.1 \leq v \leq 0.13$, $0.26 \leq b \leq 0.48$.

Note that if U and V do not output the exact target values of 223 and 71 hospitalizations, respectively, then F measures the sum of squares of errors in achieving the target values.

Here are excerpts from our *Mathematica* notebook accomplishing the task of minimizing F:

```
Function Definitions

Ir[x_, y_, p_, v_, b_] :=
 .4 / (.12 (1 - x) + .12 x (1 - p) + .55 (1 - y) (1 - v) + .55 y (1 - p) + .33 (1 - y) (1 - b) + .33 y (1 - p))

U[Hr_, x_, y_, p_, v_, b_] := 1000000 Hr Ir[x, y, p, v, b] ((1 - x) + x (1 - p) (1 - 0.79))

V[Hr_, x_, y_, p_, v_, b_] :=
 1000000 Hr Ir[x, y, p, v, b] ((1 - y) (1 - v) (1 - 0.67) + y (1 - p) (1 - 0.94))

F[Hr_, x_, y_, p_, v_, b_] := (U[Hr, x, y, p, v, b] - 223) ^2 + (V[Hr, x, y, p, v, b] - 71) ^2

Minimuizing the Sum of Squared Errors

NMinimize[{F[0.0013, x, y, p, v, b], x < 0.73, x > 0.61, y < x, p < 0.57, p > 0.3, v < .13,
  v > .01, b < 0.48, b > 0.26}, {x, y, p, v, b}]
{1438.48, {x → 0.73, y → 0.722605, p → 0.3, v → 0.01, b → 0.26}}
```

With Hr = 0.0013 *Mathematica's*, error-minimizing values x = 0.73, y ≈ 0.723, p = 0.3, v = 0.01, and b = 0.26 yield a sum of squared errors ≈ 1438.

```
NMinimize[{F[0.0012, x, y, p, v, b], x < 0.73, x > 0.61, y < x, p < 0.57, p > 0.3, v < .13,
  v > .01, b < 0.48, b > 0.26}, {x, y, p, v, b}]
{289.318, {x → 0.73, y → 0.72413, p → 0.3, v → 0.01, b → 0.26}}
```

With Hr = 0.0012 Mathematica indentifies inputs for which the sum of squared errors ≈ 289.

```
NMinimize[{F[0.0011, x, y, p, v, b], x < 0.73, x > 0.61, y < x, p < 0.57, p > 0.3, v < .13,
  v > .01, b < 0.48, b > 0.26}, {x, y, p, v, b}]
{4.00152 × 10^-16, {x → 0.728554, y → 0.706268, p → 0.304465, v → 0.0629342, b → 0.293315}}
```

With Hr = 0.0011 *Mathematica indentifies inputs for which the sum* of squared errors ≈ 0; inputs yield an essentially perfect fit

```
NMinimize[{F[0.0010, x, y, p, v, b], x < 0.73, x > 0.61, y < x, p < 0.57, p > 0.3, v < .13,
  v > .01, b < 0.48, b > 0.26}, {x, y, p, v, b}]
{2.01948 × 10^-28, {x → 0.701745, y → 0.668187, p → 0.356717, v → 0.0926556, b → 0.349519}}
```

With Hr = 0.001 *Mathematica finds inputs for which the sum* of squared errors ≈ 0, inputs yield an essentially perfect fit.

The output of the final instance above of *Mathematica*'s "`NMinimize`" command: ${}_uF_{pi} = x$ = 0.701745, ${}_vF_{pi} = y$, = 0.668187, $E_{pi} = E_h = p$ = 0.356717, $E_v = v$ = 0.0926556, and $E_b = b$ = 0.349519, together with the corresponding value of I_r:

```
Ir[0.701745, 0.668187, 0.356717, 0.0926556, 0.349519]
0.567353
```

as well as $H_r = 0.0010$, $HRR_{pi} = 0.79$, $HRR_v = 0.67$, and $HRR_h = 0.94$ can be entered into our original formulations of our functions U and V:

$$U = (1 - F_{pi}) \cdot (1\text{m}) \cdot I_r \cdot H_r + F_{pi} \cdot (1\text{m}) \cdot I_r \cdot (1 - E_{pi}) \cdot H_r \cdot (1 - HRR_{pi})\ [F_{pi} = {}_uF_{pi}],$$

$$V = (1 - F_{pi}) \cdot (1\text{m}) \cdot I_r \cdot (1 - E_v) \cdot H_r \cdot (1 - HRR_v) + F_{pi} \cdot (1\text{m}) \cdot I_r \cdot (1 - E_h) \cdot H_r \cdot (1 - HRR_h)\ [F_{pi} = {}_vF_{pi}];$$

the result is $U \approx 222.9999$ and $V \approx 71.0001$. Note that the preceding outputs are produced by inputs lying in our identified plausible ranges and that $I_r \approx 0.567$ is a plausible value for the fraction of the infection-naïve unvaccinated population of 18–25 year-olds in Ontario that becomes infected with COVID-19 over the evaluation period 1/1/22–5/31/22 (given our assumption of a 40% increase in infection level in the general population of 18–25 year-olds). Also keep in mind that the fraction of the unvaccinated and vaccinated having infections prior to 1/1/22 includes those who were infected before vaccination was available to 18–25 year-olds and includes those having asymptomatic infections. We note that the IHR value $H_r = 0.0010$ is consistent with Connecticut COVID Hospitalization data for 2020. This data, together with the Herrera-Esposito model [9], suggests $H_r \approx 0.00103$ for 18–25-year-olds in Connecticut [Supplement S1, Appendix S1]. Given that the state of Connecticut is considered to be one of the "healthiest states in the U.S." [39, 40], we might assume that those in Connecticut and Ontario have comparable health and that age-range-specific COVID IHRs would be approximately the same. Recalling that we would expect the Omicron IHR to be slightly less than that for the ancestral strain [26, 27], the values $H_r \approx 0.00103$ derived from Connecticut data and $H_r = 0.0010$ derived from our modeling are certainly consistent.

We remark that when $H_r = 0.0010$ (or 0.0011), there are many different combinations of key input variables in their plausible ranges yielding an essentially perfect modeling fit. We also remark that when $H_r = 0.0010$, the variable values $E_{pi} = 0.356717$ and $E_b = 0.349519$ contributing to the essentially perfect fit described above are such that $E_b < E_{pi}$, a relationship consistent with evaluation-period data for booster-protection and prior-infection-protection discussed in Section S7 of Supplement S1.

References

1. Feeny, D., Kaplan, M.S., Huguet, N. et al. Comparing population health in the United States and Canada. Popul. Health Metr. 2010;8:8. doi: 10.1186/1478-7954-8-8

2. COVID-19 vaccination in Canada. Government of Canada. Available online: https://health-infobase.canada.ca/covid-19/vaccine-administration/

3. COVID-19 vaccine surveillance report Week 31. UK Health Security Agency. 4 August 2022. Available online: https://assets.publishing.service.gov.uk/government/uploads/system/uploads/attachment_data/file/1096327/Vaccine_surveillance_report_week_31_2022.pdf

4. COVID-19 vaccination in Canada. Figure 4: Cumulative percent of people who have completed the primary series with a COVID-19 vaccine in Ontario by age group and report week. Government of Canada. Available online: https://health-infobase.canada.ca/covid-19/vaccination-coverage/.

5. UK Health Security Agency. COVID-19 vaccine surveillance report Week 51, published 23 December 2021. Available online: https://assets.publishing.service.gov.uk/government/uploads/system/uploads/attachment_data/file/1043608/Vaccine_surveillance_report_-_week_51.pdf

6. UK Health Security Agency. COVID-19 vaccine surveillance report 24 March 2022. Available online: https://assets.publishing.service.gov.uk/government/uploads/system/uploads/attachment_data/file/1063023/Vaccine-surveillance-report-week-12.pdf

7. Lau, J.J., Cheng, S.M.S., Leung, K. et al. Real-world COVID-19 vaccine effectiveness against the Omicron BA.2 variant in a SARS-CoV-2 infection-naive population. Nat. Med. 2023;29:348–357. doi: 10.1038/s41591-023-02219-5.

8. Bobrovitz, N., Ware, H., Ma, X., Li, Z., Hosseini, R., Cao, C., et al. Protective effectiveness of previous SARS-CoV-2 infection and hybrid immunity against the omicron variant and severe disease: a systematic review and meta-regression. Lancet Infect. Dis. 2023;23:556–567. doi: 10.1016/S1473-3099(22)00801-5

9. Herrera-Esposito, D., de los Campos, G. Age-specific rate of severe and critical SARS-CoV-2 Infections estimated with multi-country seroprevalence studies. BMC Infect.Dis. 2022;22:311–325. doi: 10.1186/s12879-022-07262-0

10. Funk, P.R., Yogurtcu, O.N., Forshee, R.A., Anderson, S.A., Marks, P.W., Yang, H. Benefit-risk assessment of COVID-19 vaccine, mRNA (Comirnaty) for age 16–29 years. Vaccine 2022;40: 2781–2789. doi: 10.1016/j.vaccine.2022.03.03

11. Kaim, A., Shetrit, S.B., Saban M. Women Are More Infected and Seek Care Faster but Are Less Severely Ill: Gender Gaps in COVID-19 Morbidity and Mortality during Two Years of a Pandemic in Israel. Healthcare (Basel) 2022;10:2355. doi: 10.3390/healthcare10122355

12. Public Health Ontario. Confirmed Cases of COVID-19 Following Vaccination in Ontario: December 14, 2020 to December 12, 2021. Available online: https://www.publichealthontario.ca/-/media/Documents/nCoV/Archives/Severe-Outcomes/2021/covid-19-cases-post-vaccination-dose-3-2021-12-20.pdf?rev=522e725c2f614397a88e926a61ed19d1&la=fr

13. Polack, F.P., Thomas, S.J., Kitchin, N., Absalon, J., Gurtman, A., et al. Safety and Efficacy of the BNT162b2 mRNA Covid-19 Vaccine. N. Engl. J. Med. 2020;383:2603–2615. doi: 10.1056/NEJMoa2034577.

14. Baden, L.R., El Sahly, H.M., Essink, B., Kotloff, K., Frey, S., et al. Efficacy and Safety of the mRNA-1273 SARS-CoV-2 Vaccine. N. Engl. J. Med. 2021;384:403–16. doi: 10.1056/NEJMoa2035389.

15. El Sahly, H.M., Baden, L.R., Essink, B., Doblecki-Lewis, S., Martin, J.M., et al. Efficacy of the mRNA-1273 SARS-CoV-2 Vaccine at Completion of Blinded Phase. N. Engl. J. Med 2021;385:1774–1785. doi: 10.1056/NEJMoa2113017.

16. Yogurtcu, O.N., Funk, P.R., Forshee, R.A., Anderson, S.A., Marks, P.W., Yang, H. Benefit-risk assessment of COVID-19 vaccine mRNA (mRNA-1273) for males age 18–64 years. Vaccin*e* 2023; 14:100325. doi: 10.1016/j.jvacx.2023.100325.

17. Estimated COVID-19 Burden. Updated 8 November 2021. United States Centers for Disease Control and Prevention. Available online: https://web.archive.org/web/20211110062857/https://www.cdc.gov/coronavirus/2019-ncov/cases-updates/burden.html

18. COVID-19 Data Comparing Vaccinated vs. Unvaccinated Continue to Be Available, Contrary to Viral Posts. FactCheck.org. Accessed 5/27/2023. Available online: https://www.factcheck.org/2022/04/scicheck-covid-19-data-comparing-vaccinated-vs-unvaccinated-continues-to-be-available-contrary-to-viral-posts/

19. Discontinued COVID Data Tracker: Rates of laboratory-confirmed COVID-19 hospitalizations by vaccination status. United States Centers for Disease Control and Prevention. Former URL: https://covid.cdc.gov/covid-data-tracker/#covidnet-hospitalizations-vaccination

20. Rosko, M., Al-Amin, M., Tavakoli, M. Efficiency and profitability in US not-for-profit hospitals. Int J Health Econ Manag. 2020;20:359–379. doi: 10.1007/s10754-020-09284-0

21. Tartof, S.Y., Qian, L., Hong, V., Wei, R., Nadjafi, R.F., Fischer, H., Li, Z., Shaw, S.F., et al. Obesity and Mortality Among Patients Diagnosed With COVID-19: Results From an Integrated Health Care Organization. Ann. Intern. Med. 2020;173:773–781. doi: 10.7326/M20-3742

22. Nagy, É., Cseh, V., Barcs, I., Ludwig, E. The Impact of Comorbidities and Obesity on the Severity and Outcome of COVID-19 in Hospitalized Patients-A Retrospective Study in a Hungarian Hospital. Int. J. Environ. Res. Public Health. 2023; 20:1372. doi: 10.3390/ijerph20021372

23. Obesity, Race/Ethnicity, and COVID-19. United States Centers for Disease Control and Prevention https://www.cdc.gov/obesity/data/obesity-and-covid-19.html.

24. Prevalence of obesity among adults, BMI ≥ 30, age-standardized. Estimates by country. Global Health Observatory data repository. World Health Organization. https://apps.who.int/gho/data/node.main.A900A?lang=en

25. Combden, S., Forward, A., Sarkar, A. COVID-19 pandemic responses of Canada and United States in first 6 months: A comparative analysis. Int. J. Health Plann Manage. 2022;37:50-65. doi: 10.1002/hpm.3323

26. Robinson, M.L., Morris, C.P., Betz, J.F., Zhang, Y., Bollinger, R., Wang, N., Thiemann, D.R., et al. Impact of Severe Acute Respiratory Syndrome Coronavirus 2 (SARS-CoV-2) Variants on Inpatient Clinical Outcome, Clin. Inf. Dis. 2023;76:1539–1549. doi: 10.1093/cid/ciac957

27. Paredes, M.I., Lunn, S.M., Famulare, M., Frisbie, L.A., Painter, I., Burstein, R., Roychoudhury, P., Xie, H., Mohamed Bakhash, S.A., Perez, R., et al. Associations Between Severe Acute Respiratory Syndrome Coronavirus 2 (SARS-CoV-2) Variants and Risk of Coronavirus Disease 2019 (COVID-19) Hospitalization Among Confirmed Cases in Washington State: A Retrospective Cohort Study. Clin. Infect. Dis. 2022;75:e536–e544. doi: 10.1093/cid/ciac279

28. Seroprevalence against SARS-CoV-2 due to infection in Canada, Results from the Government of Canada's COVID-19 Immunity Task Force and other partners' funded studies through to May 31, 2022. 5 July 2022. Available online: https://www.covid19immunitytaskforce.ca/wp-content/uploads/2022/07/CITF_Bespoke-report_Omicron-tsunami_2022_FINAL_ENG.pdf

29. Clarke, K., Jones, J., Deng, Y., Nycz, E., Lee, A., et al. Seroprevalence of Infection-Induced SARS-CoV-2 Antibodies — United States, September 2021–February 2022. MMWR Morb. Morta.l Wkly. Rep. 2022;71:606-608. doi: 10.15585/mmwr.mm7117e3

30. 2022 Nationwide COVID-19 Infection- and Vaccination-Induced Antibody Seroprevalence (Blood donations). Centers for Disease Control and Prevention. Available online: https://covid.cdc.gov/covid-data-tracker/#nationwide-blood-donor-seroprevalence-2022

31. Breakdown of COVID-19 positive hospital admissions. Government of Ontario. Available online: https://data.ontario.ca/dataset/breakdown-of-covid-19-positive-hospital-admissions

32. Voor In 't Holt, A.F., Haanappel C.P., Rahamat-Langendoen, J., Molenkamp, R., van Nood, E., van den Toorn, L.M., Peeters, R.P., van Rossum, A.M.C., Severin, J.A. Admissions to a large tertiary care hospital and Omicron BA.1 and BA.2 SARS-CoV-2 polymerase chain reaction positivity: primary, contributing, or incidental COVID-19. Int. J. Infect. Dis. 2022;22:665–668. doi: 10.1016/j.ijid.2022.07.030

33. Song, S., Madewell, Z.J., Liu, M., Longini, I.M., Yang, Y. Effectiveness of SARS-CoV-2 vaccines against Omicron infection and severe events: a systematic review and meta-analysis of test-negative design studies. Front. Public Health. 2023;11:1195908. doi: 10.3389/fpubh.2023.1195908

34. Stowe, J., Andrews, N., Kirsebom, F. et al. Effectiveness of COVID-19 vaccines against Omicron and Delta hospitalisation, a test negative case-control study. Nat. Commun. 2022;13:5736. doi:10.1038/s41467-022-33378-7

35. COVID-19 Vaccine data by age. Government of Ontario. https://data.ontario.ca/en/dataset/covid-19-vaccine-data-in-ontario/resource/775ca815-5028-4e9b-9dd4-6975ff1be021

36. COVID-19 Vaccination: Vaccination Coverage. Data for Figure 3. Government of Canada. https://health-infobase.canada.ca/covid-19/vaccination-coverage/.

37. Eythorsson, E., Runolfsdottir, H.L., Ingvarsson, R.F., Sigurdsson, M.I., Palsson, R. Rate of SARS-CoV-2 Reinfection During an Omicron Wave in Iceland. JAMA Netw. Open. 2022;5:e2225320. doi:10.1001/jamanetworkopen.2022.25320

38. Shrestha, N.K., Shrestha, P., Burke, P.C., Nowacki, A.S., Terpeluk, P., Gordon, S.M. Coronavirus Disease 2019 Vaccine Boosting in Previously Infected or Vaccinated Individuals. Clin. Infect. Dis. 2022;75:2169–2177. doi: 10.1093/cid/ciac327

39. Sharecare's Community Well-Being Index 2020 State Rankings Report. Available online: https://wellbeingindex.sharecare.com/wp-content/uploads/2021/05/Sharecare-Community-Well-Being-Index_2020-State-Rankings-vFINAL.pdf

40. Johnson, S.R. The 10 Healthiest States in the U.S. U.S. News & World Report. 6 May 2025. Available online: https://www.usnews.com/news/best-states/slideshows/10-healthiest-states-in-the-us

41. Brown, P., Hang, F., Bansal, A., Newcombe, L., Colwell, K., et al. Omicron BA.1/1.1 SARS-CoV-2 Infection among Vaccinated Canadian Adults. N. Eng. .J Med. 2022;386: 2337–2339. doi: 10.1056/NEJMc2202879

42. Alfego, D., Sullivan, A., Poirier, B., Williams, J., Grover, A., et al. A population-based analysis of the longevity of SARS-CoV-2 antibody seropositivity in the United States. EClinicalMedicine 2021; doi: DOI: https://doi.org/10.1016/j.eclinm.2021.100902

43. Public Health Ontario. Confirmed Cases of COVID-19 Following Vaccination in Ontario: December 14, 2020 to October 3, 2021. Available online: https://www.publichealthontario.ca/-/media/Documents/nCoV/Archives/Severe-Outcomes/2021/covid-19-cases-post-vaccination-2021-10-13.pdf?rev=458161407c084530b4aff901f2bbb615&sc_lang=en

44. Updated Eligibility for PCR Testing and Case and Contact Management Guidance in Ontario. Backgrounder. Government of Ontario. 30 December 2021. Available online: https://news.ontario.ca/en/backgrounder/1001387/updated-eligibility-for-pcr-testing-and-case-and-contact-management-guidance-in-ontario. Accessed 4/28/2023.

45. Public Health Ontario. Archive of COVID-19 Daily Epidemiological Summary. Available online: https://www.publichealthontario.ca/en/Data-and-Analysis/Infectious-Disease/COVID-19-Data-Surveillance/Archives/Daily-Epi-Summary

46. Klein, N. Myocarditis analyses in the vaccine safety datalink: rapid cycle analyses and "Head-to-Head" product comparisons. CDC ACIP meeting on COVID-19 vaccines, October 2021. Available online: https://www.cdc.gov/vaccines/acip/meetings/downloads/slides-2021-10-20-21/08-COVID-Klein-508.pdf.

47. Buchan, S.A., Seo, C.Y., Johnson, C., et al. Epidemiology of myocarditis and pericarditis following mRNA vaccines in Ontario, Canada: by vaccine product, schedule and interval. Preprint. medRxiv 2021.12.02.21267156; doi: 10.1101/2021.12.02.21267156

48. Buchan, S.A.; Seo, C.Y.; Johnson, C.; et al. Epidemiology of Myocarditis and Pericarditis Following mRNA Vaccination by Vaccine Product, Schedule, and Interdose Interval Among Adolescents and Adults in Ontario, Canada. JAMA Netw. Open 2022;5: e2218505. doi:10.1001/jamanetworkopen.2022.18505

49. Patone, M., Mei, X.W., Handunnetthi, L., Dixon, S., Zaccardi, F., Shankar-Hari, M., Watkinson, P., Khunti, K., Harnden, A., Coupland, C.A.C., Channon, K.M., Mills, N.L., Sheikh, A., Hippisley-Cox, J. Risk of Myocarditis After Sequential Doses of COVID-19 Vaccine and SARS-CoV-2 Infection by Age and Sex. Preprint. medRxiv 2021.12.23.21268276; doi: 10.1101/2021.12.23.21268276

50. Patone, M., Mei, X.W., Handunnetthi, L., Dixon, S., Zaccardi, F., Shankar-Hari, M., Watkinson, P., Khunti, K., Harnden, A., Coupland, C.A.C., Channon, K.M., Mills, N.L., Sheikh, A., Hippisley-Cox, J. Risk of Myocarditis After Sequential Doses of COVID-19 Vaccine and SARS-CoV-2 Infection by Age and Sex. Circulation 2022;146:743–754. doi: 10.1161/CIRCULATIONAHA.122.059970

51. Bourdon, P.S., Pantazatos, S.P. Why a major study on myocarditis risk following COVID vaccination should not influence public-health policy. Front. Med. 2023;10:1126945. doi: 10.3389/fmed.2023.1126945

Appendix S1: COVID Hospitalization Data from Public Health Ontario

Table 1b. Hospitalizations (including intensive care unit admissions) confirmed cases of COVID-19 by vaccination status: Ontario, December 14, 2020 to January 3, 2022

Age (years)	Hospitalized partially vaccinated cases: Number	Hospitalized post-dose 2 cases: Number	Hospitalized post-dose 3 cases: Number	Hospitalized unvaccinated cases: Number
5-11	4	0	0	82
12-17	6	8	0	130
18-29	18	39	1	1,024
30-39	34	47	3	1,679
40-49	56	71	3	2,295
50-59	131	101	13	3,628
60-69	253	250	13	4,061
70-79	349	313	38	3,855
80+	668	564	55	3,963

Notes:

1. Individuals with unknown age are excluded.
2. Post-dose 2 cases include a small number of individuals that completed their primary series with a vaccine product with a 1-dose schedule (i.e. Janssen).
3. Age groups are informed by vaccine product recommendations (i.e. no vaccine currently authorized or recommended in individuals <5 years of age) and vaccine program eligibility.
4. Trends in hospitalizations in the most recent weeks should be interpreted with caution due to delays in reporting.

Table 3a. Hospitalized confirmed cases of COVID-19 by vaccination status: Ontario, December 14, 2020 to January 30, 2022

Age (years)	Hospitalized cases post-series initiation: Number	Hospitalized cases post-series completion: Number	Hospitalized cases post-booster dose: Number	Hospitalized cases post-two booster doses: Number	Hospitalized unvaccinated cases: Number
**5-11**	11	0	0	0	125
**12-17**	10	33	0	0	149
**18-29**	29	137	8	0	1,094
**30-39**	52	143	15	0	1,773
**40-49**	76	217	35	0	2,413
**50-59**	155	355	73	0	3,869
**60-69**	295	744	156	0	4,444
**70-79**	389	1,029	301	0	4,315
**80+**	713	1,563	561	1	4,506
**Total**	**1,730**	**4,221**	**1,149**	**1**	**22,691**

Table 3. Hospitalized Confirmed Cases of COVID-19 by Vaccination Status: Ontario, December 14, 2020 to May 22, 2022

Age (years)	Hospitalized unvaccinated cases: Number	Hospitalized cases post-series initiation: Number	Hospitalized cases post-series completion: Number	Hospitalized cases post-booster dose: Number	Hospitalized cases post-two booster doses: Number
**5-11**	173	41	17	1	0
**12-17**	175	11	83	7	0
**18-29**	1,175	42	245	74	2
**30-39**	1,917	70	274	81	3
**40-49**	2,549	105	358	142	2
**50-59**	4,146	175	591	280	19
**60+**	15,013	1,566	4,950	3,979	338
**Total**	**25,151**	**2,010**	**6,518**	**4,564**	**364**

Table 3. Hospitalized Confirmed Cases of COVID-19 by Vaccination Status: Ontario, December 14, 2020 to June 5, 2022

Age (years)	Hospitalized unvaccinated cases: Number	Hospitalized cases post-series initiation: Number	Hospitalized cases post-series completion: Number	Hospitalized cases post-booster dose: Number	Hospitalized cases post-two booster doses: Number
**5-11**	174	43	17	1	0
**12-17**	177	11	87	8	1
**18-29**	1,175	45	254	76	2
**30-39**	1,919	70	280	84	3
**40-49**	2,553	105	360	150	2
**50-59**	4,154	175	600	303	20
**60+**	15,091	1,572	5,014	4,176	414
**Total**	**25,246**	**2,021**	**6,612**	**4,798**	**442**

x

Table 1. Rate of COVID-19 hospitalizations per 100,000 person days by vaccination status and age group in the previous 30 days: Ontario Jan 1-30,2022

Age (years)	Rate per 100,000 person days: Unvaccinated	Rate per 100,000 person days: Post-series completion	Rate per 100,000 person days: Post-series completion and one booster dose	Rate ratio: Unvaccinated/Post-series completion	Rate ratio: Unvaccinated/Post-series completion and one booster dose
5-11	0.23	0.00	0.00	N/A	N/A
12-17	0.43	0.11	0.00	3.91	N/A
18-29	0.57	0.18	0.06	3.17	9.50
30-39	1.13	0.21	0.10	5.38	11.30
40-49	1.73	0.36	0.21	4.81	8.24
50-59	3.22	0.69	0.25	4.67	12.88
60-69	18.51	1.74	0.55	10.64	33.65
70-79	148.83	5.37	1.13	27.72	131.71
80+	103.93	15.99	3.64	6.50	28.55

Appendix S2: Estimating the Fraction of Unvaccinated Ontarians 18–25 Having Been COVID-19 Infected Before 1 January 2022

To test our COVID-hospitalizations model using data from Ontario over the evaluation period 1/1/22–5/31/22, we seek a range of plausible values for ${}_uF_{pi}$, the fraction of unvaccinated Ontarians 18–29 having been COVID-19 infected before 1 January 2022 (and we assume that the fraction infected in the age range 18–25 is approximately the same).

To find a range of plausible values of ${}_uF_{pi}$, we rely on findings of a study [41] (reference list above), which are based on both survey and seroprevalence data for all of Canada. The discussion of [41] focuses on data from phases 3 and 4 of the study, with phase 3 conducted between 15 August 2021 and 15 October 2021, and phase 4, between 24 January 2022 and 15 March 2022. Table S1 in the supplementary appendix of [41] reports that 42.3% and 41.5% of dried-blood-spot "DBS" samples for phases 3 and 4, respectively, were provided by Ontarians. The appendix also reports in Table S1 that 5.6% of the 5155 persons providing DBS's for phase 3 were unvaccinated.

We will use information about the unvaccinated participants in phase 3 to estimate their infection level at the end of phase 3. We emphasize that our goal is to arrive at a range of plausible infection levels. We've already noted that the number of unvaccinated participants in Phase 3 is about $0.056 \cdot 5155 \approx 289$. Figure 1B of [41] indicates that 5% of phase-3 participants of ages 18–59 were both unvaccinated infection-naïve (based on phase-3 surveys and antibody testing), while 2% of those 60 or greater were both unvaccinated and infection-naïve. Using age-group percentages from Table S1 of [41] for those providing phase-3 DBS samples, we find for phase 3 there were approximately $0.05 \cdot 0.545 \cdot 5155 \approx 140$ infection-naïve unvaccinated persons 18–59 and approximately $0.02 \cdot 0.455 \cdot 5155 \approx 47$ infection-naïve unvaccinated persons of ages 60 and above. With 140 + 47 = 187 infection naïve unvaccinated persons and a total of 289 unvaccinated persons, we arrive at a phase-3 infection-incidence among the unvaccinated, ages ≥ 18, of $102/289 \approx 0.35$.

The supplementary appendix of [41] describes the phase-3 criteria for classifying unvaccinated study participants as having been infected (with COVID-19):

> The age-specific "immunity wall" in Figure 1B defines infection as either having tested positive on polymerase chain reaction or antigen rapid test or with antibodies to the NP antigen (which is appropriate among the largely vaccinated cohort). NP antibodies reflect infection and would not arise from Canadian-approved vaccines that only contain the spike protein. In unvaccinated participants, spike and RBD positivity would be indicative of infection and would capture additional cases where a person did not seroconvert for NP antibodies. Hence, spike or RBD seropositivity with NP seronegativity were also considered as infection among the unvaccinated (this does not apply to the vaccinated since vaccination would induce spike and RBD seropositivity). For the pre-Omicron period [including all of phase 3], we used cumulative prevalence of infection, which is defined as any positive COVID test within two months of DBS testing and any NP seropositive.

With the preceding definition of infection for study-group participants in mind, consider the following from the third paragraph of the "Discussion" section of [42], which suggests several ways that infections in the phase-3 population of [41] may be under-ascertained:

> Interestingly, both antibodies [spike and NP] achieve peak [following a linear trend] of about 90% seropositivity by three weeks post-index [PCR-positive test]. The remaining 10% could reflect immunocompromised individuals with no antibody response, possible false negative antibody testing, false positive PCR testing, or those asymptomatic or with low viral load.[14]

Assuming a possible under-ascertainment of infections of 10% among the unvaccinated phase-3 population, would mean that about 19 of the approximately 187 identified as infection naïve, ages $\geq$ 18, would actually be infected yielding a phase-3 infection-incidence among the unvaccinated, ages $\geq$ 18, of $121/289 \approx 0.42$.

Our *possible* fraction 0.42 of phase-3 infected is for the entire age range 18 and over. Infection is much more likely in the age group of interest 18–29 than in older age groups. Consider the "All cases" data in the final column of the following table from [43], reflecting data through 3 October 2021 (and note 10/3/21 lies in the phase-3 period):

Table 2. Demographic characteristics of partially vaccinated and breakthrough confirmed cases of COVID-19: Ontario, December 14, 2020 to October 3, 2021

Characteristic	Symptomatic and asymptomatic partially vaccinated cases: Number (% of all cases)	Symptomatic and asymptomatic breakthrough cases: Number (% of all cases)	Symptomatic and asymptomatic post-dose 3 cases: Number (% of all cases)	All cases*: Number
**Gender**				
**Female**	10,932 (5.0%)	6,055 (2.8%)	7 (<0.1%)	218,581
**Male**	8,922 (4.0%)	4,409 (2.0%)	2 (<0.1%)	222,472
**Age (years)**				
**12-17**	437 (1.6%)	281 (1.0%)	0 (0.0%)	26,957
**18-29**	3,176 (2.9%)	2,221 (2.0%)	0 (0.0%)	110,359
**30-39**	2,507 (3.4%)	2,077 (2.8%)	0 (0.0%)	74,240
**40-49**	2,587 (4.1%)	1,823 (2.9%)	0 (0.0%)	63,422
**50-59**	3,244 (5.4%)	1,490 (2.5%)	0 (0.0%)	60,476
**60-69**	3,648 (9.7%)	1,267 (3.4%)	2 (<0.1%)	37,462
**70-79**	2,272 (12.6%)	630 (3.5%)	1 (<0.1%)	18,025
**80+**	2,090 (14.2%)	780 (5.3%)	6 (<0.1%)	14,694
**Total**	**19,974 (4.5%)**	**10,569 (2.4%)**	**9 (0<0.1%)**	**444,714**

*Includes all confirmed COVID-19 cases (both vaccinated and unvaccinated, as well as both symptomatic and asymptomatic cases) since December 14, 2020.

Below, we have reproduced reported cases from the preceding table, added population data to arrive at case rates per 100,000, and appended, on the right, age-group percentages of phase 3 DBS participants [41, Table S1].

Table SA2: Case Rates by Age

(A)

Age (years)	All cases: Number	Population*	Cases per 100,000	Fraction of 18–29 Rate
18–29	110,359	2454802	4496	1
30–39	74,240	2077137	3574	0.795
40–49	63,422	1855340	3418	0.760
50–59	60,476	2019276	2995	0.666
60–69	37,462	1813493	2066	0.459
70–79	18,025	1181151	1526	0.339
80+	14,694	674976	2177	0.484

(B)

Age range	Percentages of Phase 3 Participants**	Approximate Percentages of Phase 3 Participants***
18–39	20.4	18–29: 11.05<br>30–39: 9.35
40–59	34.1	40–49: 16.33<br>50-59: 17.77
60–69	30.0	60–69: 30.0
70-+	15.5	70–79: 9.86<br>80+: 5.64

* Population data obtained through [35]

** Table S1 of [41]

*** Assuming population within the larger range (e.g., 18-39) is distributed into the smaller ranges (e.g., 18–29, 30–39) as in the whole population (3rd column of the table (A))

---

[14] Regarding false positive PCR-test results: Public Health England reports The RT-PCR assays used for the UK's COVID-19 testing programme have been verified by PHE, and show over 95% sensitivity and specificity. This means that under laboratory conditions, these RT-PCR tests should never show more than 5% false positives or 5% false negatives.

Assume that (i) the unvaccinated phase 3 DBS population has an age distribution following the percentages in the rightmost column of the Table SA2(B); (ii) the rates of infection among age groups in the unvaccinated phase 3 DBS population are related the way that case rates are in the rightmost column of Table SA2(A), and (iii) r is the infection incidence-rate among the unvaccinated the 18–29 range (so that, e.g., $0.795r$ would be the infection rate for the 30–39 range). We conclude that the overall incidence of infection in the unvaccinated phase 3 DBS population, 42%, is represented as follows:

(†) $42 = 11.05r + 9.35 \cdot 0.795r + 16.33 \cdot 0.760r + 17.77 \cdot 0.666r + 30 \cdot 0.459r + 9.86 \cdot 0.339r + 5.64 \cdot 0.484r,$

which reduces to $42 = 62.5712\, r$, so that $r \approx 0.67$. That is, assuming a 10% under-ascertainment of infections among unvaccinated participants in phase 3 of [41], we might expect the percentage of unvaccinated persons, 18–29 in Ontario, at end of phase 3 (15 October 2021) to be approximately 67%. Had we not assumed under-ascertainment of infections of 10%, replacing 42 with 35 in equation (†), we would have arrived at $r \approx 0.56$. Thus, we assume that as of 15 October 2021, the fraction of unvaccinated 18–29 year-olds in Ontario having had a COVID-19 infection lies in the range [0.56, 0.67].

To support the plausibility of this range for *unvaccinated* 18–29 year-olds in Ontario as of 15 October 2021, we note that if we take the CDC's infected-rate estimate of $r = 0.549$ for a mixed population of vaccinated and unvaccinated 18–49 year-olds in the U.S. as of 30 September 2021 [17], and then assume the 18–49 population is distributed into the age ranges 18–29, 30–39, and 40–49 as in Ontario with the same corresponding case-rates per 100,000, we'd obtain $r = 0.65$ for the 18–29 age group (and would expect r would be larger if we restricted to *unvaccinated* persons 18–29).

To estimate the increase in infections among the unvaccinated, infection naïve 18–29 year-olds in Ontario between 15 October 2021 to 31 December 2021, we use the following finding from [41]:

> *Among 91 unvaccinated, uninfected participants in phase 3 of the study [who also participated in phase 4], 36 had a positive result in phase 4 (education-weighted between-phase incidence, 40% (95% CI, 25 to 54).*

Thus, we will assume that during the "between-phase" period (16 October 2021–23 January 2022), 40% of the infection-naïve unvaccinated 18–29 year-olds became infected with COVID-19. Assuming the lower bound of 56% having had prior infection by 10/15/21, then, we have 40% of the 44% (namely, 17.6%) of the infection-naive becoming infected, so that as of 24 January 2022, the fraction of unvaccinated persons having had at least one episode of COVID-19 infection would have risen to 0.736 (from 0.56 at the end of phase 3). A similar computation starting with the upper bound 0.67 would yield a corresponding fraction having had an infection by 24 January 2022 of 0.802.

We seek to estimate the fraction of the unvaccinated 18–29 year-olds in Ontario having had a prior infection by 1/1/22. Thus, we need to estimate what portion of the 40% rise in infections over 10/16/21–1/23/22 among the infection-naïve unvaccinated takes place during the period 10/16/21-12/31/21. We attempt to obtain this estimate using confirmed-Covid-case data from Ontario. Our task is made more difficult owing to changes in Ontario COVID testing policies

On 30 December 2021, the Ontario government updated its COVID-19 testing and isolation guidelines [44], announcing "key changes" including

- Symptomatic testing will be available for high-risk individuals, and individuals who work in high-risk settings.
- Individuals with symptoms consistent with COVID-19 are presumed positive and they should follow isolation and/or self-monitoring guidelines.
- Testing for asymptomatic contacts of cases is generally no longer recommended, except for high-risk contacts/individuals that are part of confirmed or suspected outbreaks in high-risk settings, as recommended by public health.

Thus, in particular, we see that over the evaluation period even many symptomatic cases would not be confirmed by testing, and Public Health Ontario reports only confirmed cases

With the preceding in mind, we consult Daily Epidemiological Summaries [45], which provide case data by age, but in age groups 5–11, 12–19, 20–39, 40–59, 60–79, and 80+. We'll use cases in the 20–39 age range to approximate infection-level changes in the age range 18–29. According to the daily summary "COVID-19 in Ontario: January 15, 2020 to October 15, 2021", cumulative cases in the 20-39 range total 223,927 through 10/15/21; according to the daily summary "COVID-19 in Ontario: January 15, 2020 to December 31, 2021", cumulative cases in the 20-39 range total 297,148 through 12/31/21; and, according to the daily summary "COVID-19 in Ontario: January 15, 2020 to January 23, 2022", cumulative cases in the 20-39 range total 385,844 through 1/23/22. We see there were 297,148-223,927 = 73,221 cases reported for the period 10/16/21 through 12/31/21 and 385,844-297,148 = 88,696 cases reported for the period 1/1/22 through 1/23/22. Given the changes to Ontario testing policies discussed above, it seems safe to assume that case totals suggest that at most $73{,}221/(73{,}221 + 88{,}696) \times 100\% \approx 45\%$ of the infections occurring between 10/16/21 and 1/23/22 occurred before 1/1/22. Assuming that 45% of the "between phases" rises from 0.56 to 0.736 and 0.67 to 0.802 occur before 1/1/22, we'd arrive at the following range for ${}_{u}F_{pi}$ after rounding to two decimal places: [0.64, 0.73]. If we assume new testing policies cut confirmed cases by ½, then we might assume that $73{,}221/(73{,}221 + 177{,}392) \times 100\% \approx 29\%$ of the infections occurring between 10/16/21 and 1/23/22 occurred before 1/1/22 and arrive at the following range for ${}_{u}F_{pi}$ after rounding to two decimal places: [0.61, 0.71]. *Thus, we will assume that* $0.61 \leq {}_{u}F_{pi} \leq 0.73$.

Appendix 3: Underascertainment of Myo/pericarditis Cases After mRNA-1273 Dose 2 by the CDC's VSD System

We present here evidence of the shortcomings of the CDC's VSD System.

Review the following figures from the 21 October 2021 ACIP-meeting presentation [46] (orange text and boxes added):

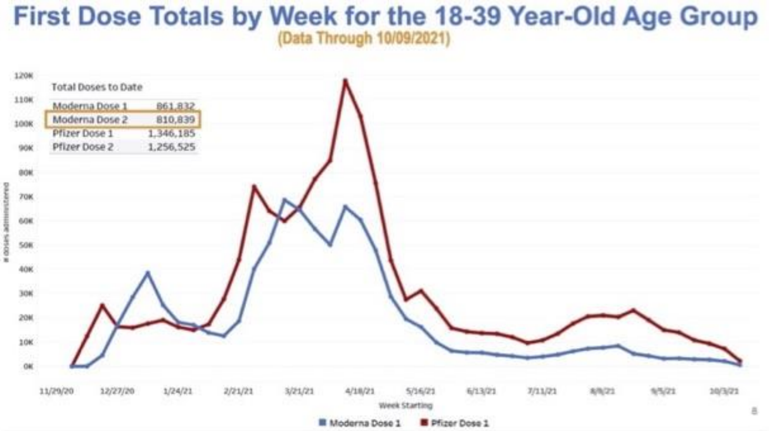

Confirmed Myocarditis/Pericarditis in the 0-7 Day Risk Interval, among 12–39-year-olds by Product and Dose

Compared with Outcome Events in Vaccinated Comparators on the Same Calendar Days

Vaccine	Dose	Events in Risk Interval	Events in Comparison Interval[1]	Adjusted Rate Ratio[2]	95% Confidence Interval	2-Sided P-value	Excess Cases in Risk Period per 1 Million Doses
Both mRNA	Both Doses	66	6	21.73	9.53 - 57.34	<0.001	11.8
	Dose 1	10	6	10.18	3.01 - 36.36	<0.001	3.3
	Dose 2	52	5	31.28	12.83 - 91.40	<0.001	21.1
Pfizer Ages 12-39	Both Doses	44	5	19.31	7.66 - 57.79	<0.001	11.5
	Dose 1	5	5	8.65	2.01 - 36.99	0.005	2.3
	Dose 2	35	4	30.27	11.01 - 104.40	<0.001	21.5
Moderna Ages 18-39	Both Doses	22	1	37.51	6.69 - 803.00	<0.001	12.8
	Dose 1	5	1	10.47	1.38 - 258.35	0.020	5.2
	Dose 2	17	0	very high[3]	11.75 -∞	<0.001	21.0

[1]Comparison interval is 22–42 days after either dose.
[2]Adjusted for VSD site, 5-year age group, sex, race/ethnicity, and calendar date.
[3]The focus should be on the lower bound of the confidence interval.

Results in this slide cannot be directly compared between Pfizer and Moderna because of the different age groups who are authorized to receive each vaccine

16

Focus only on data from the preceding two slides for Moderna dose 2. Based on data through 10/9/21, we see that the CDC's VSD system has identified for 18–39 year-olds 17 confirmed myo/pericarditis cases in 7-day risk windows following 810,939 second doses of Moderna's mRNA-1273. Observe that the corresponding incidence rate is 17/0.810939 ≈ 21 myo/pericarditis cases per million 2nd doses administered to 18–39 year-olds (males & females).

Now focus on corresponding Moderna dose 2 data from Table 4 (below) of Buchan et al.'s study [47]:

Table 4. Observed vs. expected episodes of myocarditis/pericarditis using a 7-day risk window following dose 2 of COVID-19 mRNA vaccines among individuals receiving dose 2 on or after June 1, 2021, by age group, sex, and vaccine product

**Age group (years)**	**Females**			**Males**		
	Individuals with 2 doses	Expected*	Observed	Individuals with 2 doses	Expected*	Observed
			**BNT162b2 – Dose 2**			
12-17	331,016	0.1-0.1	**4**	338,234	0.4-0.5	**31**
18-24	255,580	0.3-0.3	**2**	245,430	0.9-1.0	**10**
25-29	196,378	0.2-0.3	**3**	190,586	0.5-0.6	**2**
30-39	404,704	0.5-0.6	**2**	369,721	1.1-1.3	**6**
40-49	404,785	0.5-0.7	0	350,902	1.0-1.1	1
50-59	460,742	0.8-1.0	0	420,927	1.2-1.4	1
60-69	441,965	1.0-1.2	0	392,472	1.3-1.5	**3**
70-79	368,666	1.0-1.3	1	319,305	1.2-1.5	**3**
≥80	193,578	0.5-0.6	0	148,837	0.5-0.7	0
			**mRNA-1273 – Dose 2**			
12-17**	-	-	-	-	-	-
18-24	170,317	0.2-0.2	**7**	179,866	0.6-0.7	**55**
25-29	133,420	0.1-0.2	0	151,079	0.4-0.5	**12**
30-39	266,347	0.3-0.4	**5**	292,548	0.9-1.0	**15**
40-49	261,699	0.4-0.4	**2**	274,340	0.8-0.9	**5**
50-59	292,890	0.5-0.6	**1**	311,910	0.9-1.0	**2**
60-69	247,723	0.6-0.7	0	249,489	0.8-0.9	**2**
70-79	139,124	0.4-0.5	0	128,971	0.5-0.6	**1**
≥80	66,729	0.2-0.2	0	47,684	0.2-0.2	0

*The expected range is estimated from the confidence intervals around the mean background rate from 2015-2019.

**Estimates were not provided for individuals aged 12-17 for mRNA-1273 because this product was not used for this age group in Ontario.

Bold results indicate where the observed number was greater than the upper confidence limit of the expected number.

Add the observed myo/pericarditis cases for the 18–24, 25–29, and 30–39 age ranges, female and male occurring in a 7-day risk window following dose 2 of mRNA-1273: 7+0+5+55+12+15=94. Now add the numbers of corresponding doses that yield the 94 cases: 170,317+ 133,420 + 266,347+179,866+151,079+292,548 = 1,193,577. We obtain the following incidence rate of 94/1.193577 ≈ 79 myo/pericarditis cases per million 2nd doses of mRNA-1273 administered to 18–39 year-olds (males & females). Remarks: all cases meet the Brighton Collaborations case definitions of myocarditis and pericarditis, levels 1–3, and the data reported in Table 4 of above reflects a period of "enhanced surveillance" for postvaccination myo/pericarditis by Public Health Ontario starting in "early June 2021" [47, p. 3].

Compare the VSD myo/pericarditis incidence rate of 21 per million second doses of mRNA-1273 administered to a mixed population of male and female 18–39 year-olds to the corresponding incidence rate of 79 per million based on Public Health Ontario data in [47, 48], noting both rates use risk windows having the same length. We see that Public Health Ontario's adverse event reporting system with "enhanced surveillance" detected 79/21 ≈ 3.76 times the number of confirmed myo/pericarditis events post Moderna dose 2 than did the CDC's VSD system.

Appendix S4: Evidence from a Study by Patone et al. that Prior Infection Increases the Risk of Vaccination-Associated Myocarditis

We find evidence that prior infection increases the risk of vaccine-associated myocarditis in Supplementary Tables 1 and 2 of a preprint [49] (available 12/25/21) reporting on COVID-vaccine-associated myocarditis, involving hospitalization or death, in a study population of over 42 million. Myocarditis incidence post dose 1 of BNT162b2 is about 7.9 ≈ 12/1.513590 cases per million among those having had a positive COVID test prior to receiving dose 1 and about 5.7 ≈ 108/18.878010 cases per million among those not having a positive COVID-test prior to receiving dose 1. Broader evidence may be found in the published version [50] of [49]. According to Table S2 of [50] and the discussion of results on p. 745 of [50], among 2,958,026 study-population members having a positive COVID-test before vaccination there were 229 myocarditis cases occurring after vaccination (not necessarily in the 1–28-day period following dose reception) yielding a myocarditis incidence rate (during the study period 12/1/20 – 12/15/21) of about 77.4 per million. Among the 39,884,319 study-population members not having had a positive test before vaccination there were 2861-229-114 = 2518 cases, yielding a postvaccination incidence of myocarditis (not necessarily in the 1–28-day period following dose reception) of about 63.1 per million. Infections in the study-population of [50] were under-ascertained [51], and thus, prior-infection's impact on risk of VAM/P might be greater than these computations indicate.
